# An Intelligent System for Classifying Patient Complaints Using Machine Learning and Natural Language Processing: Development and Validation Study

**DOI:** 10.2196/55721

**Published:** 2025-01-08

**Authors:** Xiadong Li, Qiang Shu, Canhong Kong, Jinhu Wang, Gang Li, Xin Fang, Xiaomin Lou, Gang Yu

**Affiliations:** 1 Children's Hospital Zhejiang University School of Medicine National Clinical Research Center For Child Health Hang Zhou China; 2 Patient Service Surveillance Office Medical Information Department Hangzhou Red Cross Hospital Hang Zhou China; 3 Department of Radiation Oncology Zhe Jiang Xiaoshan hospital Hangzhou Normal University Hang Zhou China; 4 Hospital Management Office Hangzhou Cancer Hospital Hang Zhou China; 5 Patient Service Surveillance Office Hangzhou Red Cross Hospital Hang Zhou China

**Keywords:** complaint analysis, text classification, natural language processing, NLP, machine learning, ML, patient complaints

## Abstract

**Background:**

Accurate classification of patient complaints is crucial for enhancing patient satisfaction management in health care settings. Traditional manual methods for categorizing complaints often lack efficiency and precision. Thus, there is a growing demand for advanced and automated approaches to streamline the classification process.

**Objective:**

This study aimed to develop and validate an intelligent system for automatically classifying patient complaints using machine learning (ML) and natural language processing (NLP) techniques.

**Methods:**

An ML-based NLP technology was proposed to extract frequently occurring dissatisfactory words related to departments, staff, and key treatment procedures. A dataset containing 1465 complaint records from 2019 to 2023 was used for training and validation, with an additional 376 complaints from Hangzhou Cancer Hospital serving as an external test set. Complaints were categorized into 4 types—communication problems, diagnosis and treatment issues, management problems, and sense of responsibility concerns. The imbalanced data were balanced using the Synthetic Minority Oversampling Technique (SMOTE) algorithm to ensure equal representation across all categories. A total of 3 ML algorithms (Multifactor Logistic Regression, Multinomial Naive Bayes, and Support Vector Machines [SVM]) were used for model training and validation. The best-performing model was tested using a 5-fold cross-validation on external data.

**Results:**

The original dataset consisted of 719, 376, 260, and 86 records for communication problems, diagnosis and treatment issues, management problems, and sense of responsibility concerns, respectively. The Multifactor Logistic Regression and SVM models achieved weighted average accuracies of 0.89 and 0.93 in the training set, and 0.83 and 0.87 in the internal test set, respectively. Ngram-level term frequency–inverse document frequency did not significantly improve classification performance, with only a marginal 1% increase in precision, recall, and *F*_1_-score when implementing Ngram-level term frequency–inverse document frequency (n=2) from 0.91 to 0.92. The SVM algorithm performed best in prediction, achieving an average accuracy of 0.91 on the external test set with a 95% CI of 0.87-0.97.

**Conclusions:**

The NLP-driven SVM algorithm demonstrates effective classification performance in automatically categorizing patient complaint texts. It showed superior performance in both internal and external test sets for communication and management problems. However, caution is advised when using it for classifying sense of responsibility complaints. This approach holds promises for implementation in medical institutions with high complaint volumes and limited resources for addressing patient feedback.

## Introduction

### Background

Patient complaints refer to patients feeling dissatisfied or having opinions regarding medical services, treatment processes, medical staff attitudes, or health care institution management during their participation in medical care [1]. These complaints are expressed through written or verbal feedback or grievances to relevant organizations or individuals. Complaints can involve various aspects, including medical errors, poor treatment outcomes, unfriendly service attitudes, and excessive waiting times among other issues.

Handling patient complaints is an essential part of health care institution management, as it can help improve the quality of medical services and enhance patient satisfaction. Patient complaints also serve as valuable sources for gaining insights into safety-related issues within health care institutions. Patients are often more sensitive to a range of issues within medical services compared with health care professionals within the institution. Some of these issues may not be identified by traditional medical surveillance systems [2], such as adverse event reporting systems or mortality case reviews. Therefore, patient complaints can provide crucial information for health care institutions on how to improve patient safety.

Currently, patient satisfaction surveys are conducted through SMS text messages or online methods (such as through a WeChat [Tencent] official account) in our hospital. If a patient responds negatively, a staff member from the outpatient office follows up with a phone call. Under traditional management methods, satisfaction survey personnel meticulously document specific complaints from patients regarding dissatisfaction with the hospital’s medical management. These complaints are then summarized and regularly sent to the hospital’s Patient Advocacy Center (PAC). They are manually categorized and reported every 2 weeks during hospital meetings and also distributed to the email addresses of relevant clinical and medical technology department heads for corrective action and improvement. Only if the patient receives feedback from the relevant department, it will be considered as closing the loop on the complaint-handling process.

On average, about 25 new complaints are submitted through various channels each day, while the PAC can only analyze and process approximately 10 complaints loops per day. At this rate, it can be estimated that the hospital’s PAC will still have over 5400 patient complaints waiting to be analyzed and addressed within a year. Clearly, without significant changes to the screening and categorization process, the hospital’s PAC will be unable to analyze all submitted complaints within a reasonable timeframe to provide adequate feedback and resolution. The crux of the issue lies in the inability to classify patient complaints in a timely manner, which consequently hampers targeted communication between subsequent departments and patients. This delay leads to patients lodging secondary or even multiple complaints due to prolonged waiting times. In addition, departments with the capability to address specific issues remain idle as they do not receive timely patient complaints. Experience with several hundred such interventions at community and academic medical centers shows fewer subsequent complaints associated with most of those receiving timely and effective feedback [3]. Therefore, tardily and ineffectively addressing patient complaints has become a significant bottleneck restricting the enhancement of hospital management quality and medical safety.

Automatic classification tools play a crucial role in streamlining the triage process, enabling health care providers to efficiently prioritize and address patient concerns based on the severity and nature of their complaints. By automating this classification process, clinicians can allocate resources more effectively, leading to improved patient outcomes and satisfaction. In addition, such tools facilitate the organization and analysis of large volumes of patient data, ultimately contributing to evidence-based decision-making and enhanced health care delivery. The problem is that there are hardly any studies, articles, or systems focusing on the automatic classification of patient complaints. The need for automatic classification of patient complaints is crucial, as manual identification of complaints is inefficient, time-consuming, and highly prone to errors.

The main contributions of this study are by automating the patient complaint triage process, PAC can allocate resources more efficiently, thereby improving patient care experience and satisfaction. In addition, such tools help organize and analyze large amounts of patient complaint data, ultimately contributing to evidence-based medical decisions and improved medical services to ensure patient treatment safety.

The structure of the article is as follows. “Existing Approaches to Text Classification” introduces previous studies on existing approaches to text classification and the patients complain auto-classification methodology. “Methods” and “Results” introduce machine learning (ML) models and experimental results. “Discussion” presents an analysis and discussion of selected results and offers some suggestions for future research. Finally, “Conclusion” summarizes our research.

### Existing Approaches to Text Classification

The classification of patient complaint texts falls within the realm of text auto-classification. Therefore, many previous studies in text classification provide valuable insights for us (Multimedia Appendix 1) [4-9]. Especially, auto-classification of texts based on patients’ chief complaints (CC) during the diagnostic process is the most relevant to our research on auto-classifying patient complaint texts.

CC text classification refers to the task of automatically categorizing or classifying text data based on the main concerns or symptoms expressed by patients when they seek medical attention [10]. In principle, each patient complaint should be classified into 1 category, but typically 1 patient complaint may be classified into a single or multiple categories because it contains more than 1 category of disease. A recorded free-text CC is assigned to a category either manually by emergency medicine physicians or other emergency department (ED) professionals, or by using an automated mapping algorithm [11-14]. Automated CC categorization, on the other hand, are more suitable for a wide range of (ED) applications. Traditional automated CC classification algorithms typically use a linear mapping algorithm (keyword search and category matching algorithm [15]), or a semantic model based on Bayesian network [12].

The study by Arnaud et al [16] leveraged natural language processing (NLP) solutions to predict medical specialties at hospital admission by integrating structured data with unstructured textual notes. An MLP model and a convolutional neural network (CNN) were independently used to achieve promising accuracy in the analysis of over 260,000 ED records. Although the classifier model could only achieve about 68% accuracy of prediction, this research contributes to the growing use of NLP methods in health care analytics. NLP models achieved high accuracy in predicting the need for admission, triage score, critical illness, and mapping free-text CC to structured fields. Incorporating both structured data and free-text data improved results when compared with models that used only structured data. However, the majority of studies (16/20, 80%) were assessed to have a high risk of bias, and only 1 study reported the deployment of an NLP model into clinical practice [17].

These traditional linear classification methods are all based on the bag-of-words (BOW) representation, which treats text as an unordered collection of words, ignoring the position and grammatical structure of words, and only focusing on the frequency of word occurrences. In the BOW model, text is represented as a vector, where each dimension corresponds to a word, and each element of the vector represents the frequency or occurrence of the corresponding word in the text. However, it is known that BOW technique suffers from issues such as loss of word order information, curse of dimensionality, sparsity, and semantic loss. These limitations restrict the performance of the BOW model in NLP tasks [18,19]. Therefore, researchers have proposed various improvement methods to overcome these issues, such as using word embedding techniques and models that consider contextual information [20]. A representative work was proposed by Alhazzani et al [21], who used pretrained static word embeddings and pretrained vector word embeddings to train deep learning (DL)–based BiLSTM (Bidirectional Long Short-Term Memory) and BiGRU (Bidirectional Gated Recurrent Unit) classifiers. Lee et al [13] developed a recurrent neural network (RNN)–based long short-term memory (LSTM) and gated recurrent unit (GRU) cells for text classification [13], and they reported that in all instances, the RNN models outperformed the BOW classifiers suggesting DL models could substantially improve the automatic classification of unstructured text for syndromic surveillance.

Currently, 1 significant innovation using the transformer architecture [22] and using bidirectional encoding to effectively capture dependency relationships from both directions of context simultaneously, has significantly propelled technological advancements across various CC text classification tasks [23,24]. The performance of those models (BERT [Bidirectional Encoder Representations from Transformers; Google], Bio_BERT, Clinical BERT, KP_BERT, CC_BERT) was compared with that of the term frequency–inverse document frequency (TF-IDF) [25] model serving as the baseline. However, the results revealed that the TF-IDF model outperforms a robust BERT-based model on the test dataset and exhibits statistical comparability in terms of misspelling sets.

Despite the application of DL methods, including CNNs, RNNs, and BERT, in text categorization—particularly in handling large-scale, high-dimensional data and significantly enhancing text classification performance for complex tasks [26,27]—traditional ML approaches continue to offer advantages in terms of computational resource requirements and the interpretability of results compared with DL, CNNs, and RNNs. Modern RNNs can have millions of free parameters and thus require huge datasets [28]. Training models of this size require significant computing resources. In the task of patient complaints classifying, besides pursuing classification accuracy, the interpretability of ML models should be prioritized. This is because our goal was to identify the root causes of patient complaints through a correct interpretation of classification results, and the interpretability of models such as DL, CNNs, and RNNs is notably low. This significantly undermines the credibility of clinical interpretations of the aforementioned analysis results and their subsequent applicability. Therefore, 3 interpretable common ML classification models (Multifactor Logistic Regression [MLR], Multinomial Naive Bayes [MNB], and Support Vector Machines [SVMs]) are used in our research work.

By training ML models on large datasets of labeled complaint data, these models can learn to accurately categorize complaints based on their content and context. This automated classification approach can significantly improve the efficiency and accuracy of complaint management systems, reducing reliance on subjective human intervention and enabling faster processing and resolution of patient complaints. In addition, it can help to identify patterns and trends in complaints so that PAC can proactively address common issues and improve overall patient satisfaction.

In this study, we explore the automatic text classification of patient complaints using 2 types of TF-IDF techniques and trained an ML tool for automatic classification of patient complaint texts written in Chinese based on the existing mature English text automatic classification models.

We posit that an automated classification architecture for the categorization of patient complaints can be created, which consists of 2 modules.

First, the NLP module is based on the TF-IDF technique. The function of this module is to perform word frequency and extract features from the patient complaint texts. By using the TF-IDF technique (Word-level TF-IDF and Ngram-level TF-IDF), the importance of each word and semantics in the text can be calculated and keywords representing the core meaning of the complaint can be extracted. This helps the model to better understand and classify the complaint texts.

Second, the ML module is to classify complaint texts. ML algorithms have been widely adopted for text classification [29-33]. However, existing research in the literature has primarily focused on the English language. Therefore, patient comments written in Chinese are currently difficult to automatically analyze using traditional ML or statistical approaches. While several classification models based on Chinese text have emerged, their efficacy, algorithmic intricacy, and substantial hardware requirements have impeded the broad implementation of this approach in scholarly discourse [34,35]. The challenge of dealing with Chinese text lies in its complex linguistic features, including character-based writing, tonal variations, and the absence of explicit word boundaries. In addition, Chinese exhibits rich contextual meanings and cultural nuances, which can pose difficulties for NLP tasks such as machine translation, sentiment analysis, and text summarization. By using translation tools (such as ChatGPT 3.5 [OpenAI] or other professional translation tools) to translate Chinese text into English, and then having English professionals review and correct the translated results, these reviewed English texts were used as input data for ML models for classification training. This approach can overcome the challenges of directly processing Chinese text and provides an effective way to use Chinese text data in the test classification process.

## Methods

### Data Acquisition and Preprocessing Flow

A retrospective study of patient complaints was conducted at Hangzhou Red Cross Hospital and Hangzhou Cancer Hospital from 2015 to 2019. A comprehensive compilation of 1817 documented complaints were carefully collated. Complaints involving the same patient and the same incident were grouped into 1 independent complaint case. This study was approved by the ethics committee, which granted a waiver of review and exempted it from the informed consent requirement. The data from Hangzhou Red Cross Hospital were used for model training and internal validation, while the data from Hangzhou Cancer Hospital were used for external testing of the model with the best performance in the ML model. All data collection and analysis methods in this study strictly adhere to the “Guidelines for Developing and Reporting Machine Learning Predictive Models in Biomedical Research” [36].

Due to the current difficulty of directly applying ML and classification training to Chinese text, coupled with the availability of mature software and tools for translating Chinese into English, along with the review of translation results by English-proficient graduate students, converting the Chinese written text into English for training purposes is a feasible and efficient approach for automatic text classification. All the texts recorded in Chinese were translated into English by ChatGPT 3.5. Furthermore, 2 postgraduates with English language skills were responsible for reviewing the machine-translated texts; if there were obvious translation errors, the texts were corrected after the 2 individuals proposed unanimous modification suggestions.

If 2 individuals had a disagreement regarding the translation of the same Chinese word, we would escalate the matter to native English-speaking professors in the International Education Department of the hospital. They would make the final decision to ensure that each identical Chinese word in all Chinese complaint texts was translated into English with complete consistency. To ensure the efficiency of the expert correction mechanism, we have established a reward system. For every flaw or error found in the machine-translated text, a certain amount of funds would be allocated from our project budget for rewards.

Once sufficient data have been collected, preprocessing is divided into the 3 steps.

In the first step, the complaint texts are tokenized. After comparing jieba (Multimedia Appendices 2 and 3), THULAC [37], and HanLP [38], the more mature tool jieba is used in this study. To achieve the best balance between speed and accuracy in tokenization, the Hidden Markov Model Bigram (HMM-Bigram) [39] algorithm is used. Part-of-speech tagging is performed at the same time as tokenization. This involves assigning labels to words based on their respective parts of speech, such as adjectives, verbs, nouns, and so on. Part-of-speech tagging is based on maximum entropy and maximum likelihood approaches.

The second step is to eliminate duplicate parts in the tokenized results and ensure that each word appears only once. In this way, a total vocabulary is generated.

In the third step, all complaint texts are searched. If a word from the total vocabulary occurs in a particular complaint text, the corresponding position is marked with 1. If it does not appear, it is marked with 0. This creates a matrix with words and tokens as horizontal and vertical axes. Each complaint text can be regarded as a vector of 1s and 0s.

### Feature Extraction

After preprocessing, feature extraction was adopted before model training. The features are presented in text form, whereby words and phrases with a strong semantic meaning are included as a feature set. The feature set contains most of the information from the entire text, which is beneficial for document classification. Content that is not included in the feature set may result in the loss of some semantic information but has minimal impact on the classification task. Complaint texts usually have a length of 10-100 words and are therefore relatively short. To achieve a relatively accurate classification, the TF-IDF technique was used for the extraction of text features.

After the preprocessing step, there are still many semantically meaningless particles or signs in the text. For example, “doctor” appears frequently in the text, but its contribution to text classification is relatively small. In addition to the common 841 Chinese stop words, we included an additional 17 frequently occurring but semantically insignificant words from the medical complaint domain into our stop words list based on unanimous recommendations from the staff of our collaborating hospital’s PAC. This decision was made based on our previous experience, as detailed in Multimedia Appendix 4. By including them as stop words, we can focus on more specific and meaningful keywords and thus improve the accuracy and relevance of extracting high-frequency words.

### Semantic Analysis

Due to the singularity of the environment in which patients complain, the content of the text has a high similarity in terms of scenes and semantics. Therefore, in addition to using Word-level TF-IDF, we also used Ngram-level TF-IDF to examine the impact of consecutive words on the importance of text. Given our dataset’s modest size of approximately 2600 cases and the need for higher granularity in data analysis, the decision was made to use n=2 for Ngram-level TF-IDF.

After TF-IDF analysis and extension of existing stop words, the problem of particles such as “doctor” can be alleviated, and the feature text is more suitable for the following ML text classification training. For example, the complaint text “Doctor issued unnecessary examination orders for financial reasons” was changed to “unnecessary examination orders financial reasons.” The top 10 high-probability words and word clouds for each type of complaint are calculated and displayed. This step not only allows for an understanding of the basic structure of existing complaint text data but also enables the examination of the correctness of manually labeled complaint texts, preparing necessary groundwork for subsequent model training and verification.

### Unbalanced Data Handling

The degree of imbalance of dataset is based on the proportion of a minority class in the whole dataset and could range from mild (20%-40%), moderate (1%-20%) to vigorous (<1%) imbalances [40]. Previous studies showed that the resampling approach is a useful preprocessing step to handle the imbalanced dataset [41]. This method modifies the imbalanced distribution of the majority and minority classes at the data level before training with classifiers. Before training the datasets, we used the Synthetic Minority Oversampling Technique (SMOTE) as an imbalanced adjustment strategy [42]. The decision was made due to a moderate dataset imbalance (sense of responsibility/communication problem=11.9%). The datasets of the minority class, including diagnosis and treatment, management problem, and sense of responsibility, were all up sampled to 719. Unlike regular SMOTE, we matched the data volume of minority classes to the majority class through up sampling, while keeping the actual amount of data in the majority class unchanged. This maneuvering minimized the impact on the raw data distribution. One important point to emphasize is that SMOTE only oversamples the internal data used for training and validation, while the independent external data used for testing remains untouched.

### Classification Model Training and Validation

In order to ensure the consistency of the labeling by the 2 experts, the complaint data were independently labeled by the 2 experts. Secondary annotations for the data of different categories were made by PAC staff from our cooperative hospital, comprising 3 people in total. Finally, the classification results were counted from 5 people for this annotation, and the category that received the most votes was determined as the final complaint classification annotation. We implemented a comparison of several well-known ML classifiers to determine if a supervised ML algorithm can achieve a reasonable classification accuracy compared to the expert rules. The complaint types are “communication problem,” “diagnosis and treatment,” “management problem,” and “sense of responsibility.” The sample counts for each type are shown in Table 1.

**Figure 1:**
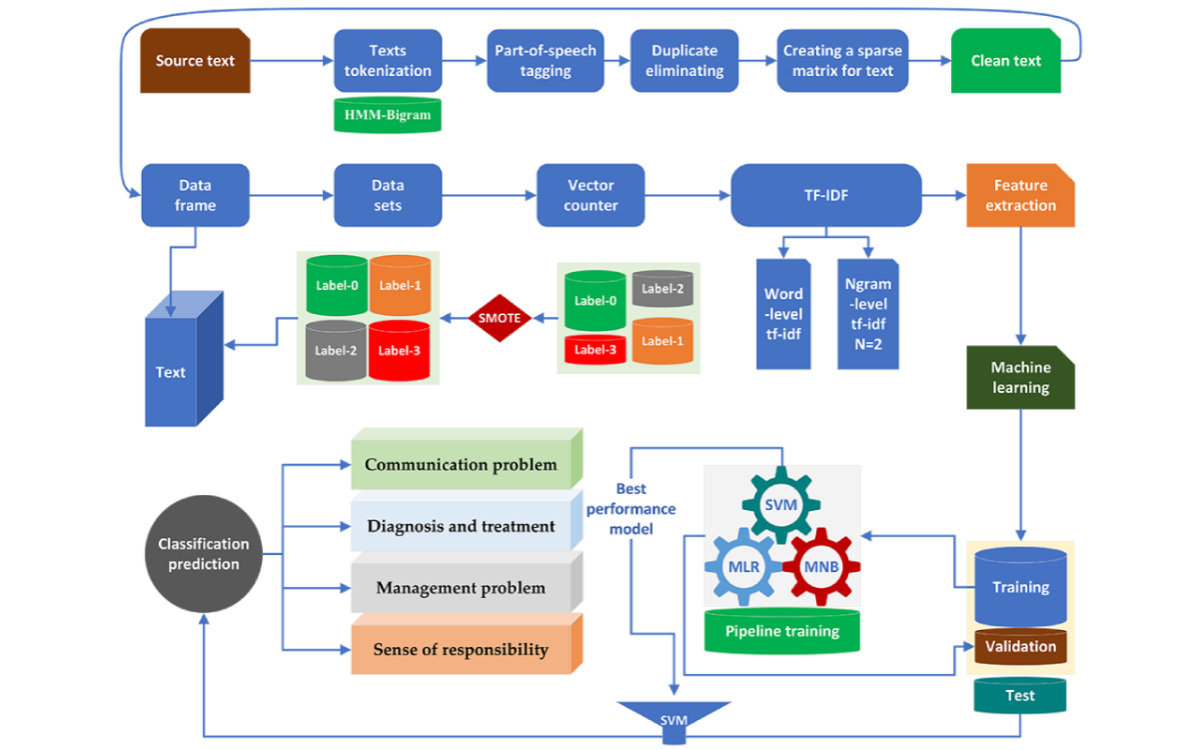
Illustration of the text preprocessing workflow and patient complaint classification model development and validation process. HMM-Bigram: Hidden Markov Model Bigram; MLR: Multifactor Logistic Regression; MNB: Multinomial Naive Bayes; SMOTE: Synthetic Minority Oversampling Technique; SVM: Support Vector Machine; TF-IDF: term frequency–inverse document frequency.

In total, 3 ML methods (MLR, MNB, and SVMs) were used to train and validate the model. In the training process, 75% (539/719) of the data in each category were used for training, the rest of 25% (180/719) was used for verification. Another 376 patient complaint records from Hangzhou Cancer Hospital were used for external testing of the trained model with the best performance in the training and verification section. The composition of the 376 patient complaint records is communication problem (n=122), diagnosis and treatment (n=89), management problem (n=85), and sense of responsibility (n=80). The performance of ML algorithms in the training and verification sets is shown in Table 2. To assess the generalization ability of the trained model in a more objective manner, a 5-fold cross-validation (CV) was applied to evaluate the model performance during external testing. Figure 1 summarizes the entire process of preprocessing and ML method for patient complaint classification.

**Table 1:** Sample number of complaints for each type in the training and verification sets.

Category	Sample size of raw data	Sample size after SMOTE^a^
Communication problem	719	719
Diagnosis and treatment	376	719
Management problem	260	719
Sense of responsibility	86	719
Total	1441	2876

^a^SMOTE: Synthetic Minority Oversampling Technique.

**Table 2:** Comparison of the performance of 3 machine learning algorithms.

Machine learning models			Communication problem				Diagnosis and treatment				Management problem					Sense of responsibility		
			Training		Validation		Training		Validation		Training		Validation		Training			Validation
**MLR^a^**																		
	Precision	0.83		0.71		0.89		0.83		0.99		0.97		1.00			0.99	
	Recall	0.95		0.91		0.88		0.81		0.94		0.88		0.96			0.89	
	*F*_1_-score	0.89		0.80		0.88		0.82		0.96		0.92		0.98			0.94	
	Support	160		140		195		183		209		197		210			200	
**SVM^b^**																		
	Precision	0.75		0.67		1.00		0.95		1.00		0.99		1.00			1.00	
	Recall	1.00		0.98		0.96		0.81		0.98		0.92		0.96			0.92	
	*F*_1_-score	0.86		0.80		0.98		0.87		0.99		0.95		0.98			0.96	
	Support	167		122		219		210		220		193		209			195	
**MNB^c^**																		
	Precision	0.73		0.67		0.98		0.95		1.00		0.99		1.00			1.00	
	Recall	1.00		0.98		0.94		0.81		0.96		0.92		0.97			0.92	
	*F*_1_-score	0.84		0.80		0.96		0.87		0.98		0.95		0.98			0.96	
	Support	134		122		219		210		220		193		219			195	

^a^MLR: Multifactor Logistic Regression.

^b^SVM: Support Vector Machine.

^c^MNB: Multinomial Naive Bayes.

### Ethical Considerations

From an ethical standpoint, the present study focused on the examination of public behavior in human subjects. The information collection methods used by the researchers were designed to ensure that subjects could not be directly identified, nor could they be indirectly identified through associated identifiers. To facilitate the effective handling of patient complaints, informed consent was obtained for telephone recordings or written complaints, with access to this information restricted to designated hospital departments. In the context of this research, only the specific content of patient complaints was analyzed, without any extraction or linkage to personal identifiable information. Furthermore, the identities of hospital staff members who were the subjects of complaints were anonymized, with only their departmental affiliation being retained. This approach served to mitigate potential conflicts between complainants and the staff involved. In addition, the potential for harm or discomfort anticipated in the study did not surpass that which individuals might have experienced in daily life or during standard physical and psychological examinations. Consequently, the hospital’s ethics review committee granted a waiver of review for this research.

The study was carried out in compliance with the Declaration of Helsinki and received ethical approval as a waiver of review from the Ethics Committee of Hangzhou Red Cross Hospital.

## Results

### Overview

In our investigation, we discovered that out of 1817 patient complaints, approximately 23 cases, accounting for 1.3% of the total, required modification post translation by ChatGPT 3.5 to avoid potential ambiguity. Among these cases, communication problem accounted for 7 instances, diagnosis and treatment only had 1, management problem had 4, while sense of responsibility constituted 11 cases. Most ambiguities were concentrated within communication problem and sense of responsibility categories, with relatively fewer occurrences in the more objective diagnosis and treatment and Management Problem categories. This observation may be attributed to the inherent difficulty patients encounter in articulating issues related to communication problem and sense of responsibility, compared with the relatively straightforward nature of diagnosis and treatment and management problem categories.

The HMM-Bigram algorithm was used for part-of-speech tagging. For the input text: “General surgery patients complained that unnecessary examination items were applied by the doctor,” the tagging results are as follows: [(‘General’, ‘JJ-TL’), (‘surgery’, ‘NN’), (‘patients’, ‘NNS’), (‘complained’, ‘VBD’), (‘that’, ‘DT’), (‘doctor’, ‘NN’), (‘applied’, ‘VBN’), (‘unnecessary’, ‘JJ’), (‘examination’, ‘NN’), (‘items’, ‘NNS’), (‘performance’, ‘NN’), (‘commissions’, ‘NNS’)]. Each tag consists of 2 parts, the first being the word itself, and the second being the part-of-speech tag for that word. For example, in the first tag of the results (‘General’, ‘JJ-TL’), “General” is the word, and “JJ-TL” is its part-of-speech tag. In this tag, “JJ” indicates an adjective, while “TL” represents the word’s tag in the Brown Corpus. Similarly, (‘surgery’, ‘NN’) indicates that “surgery” is a noun.

**Figure 2:**
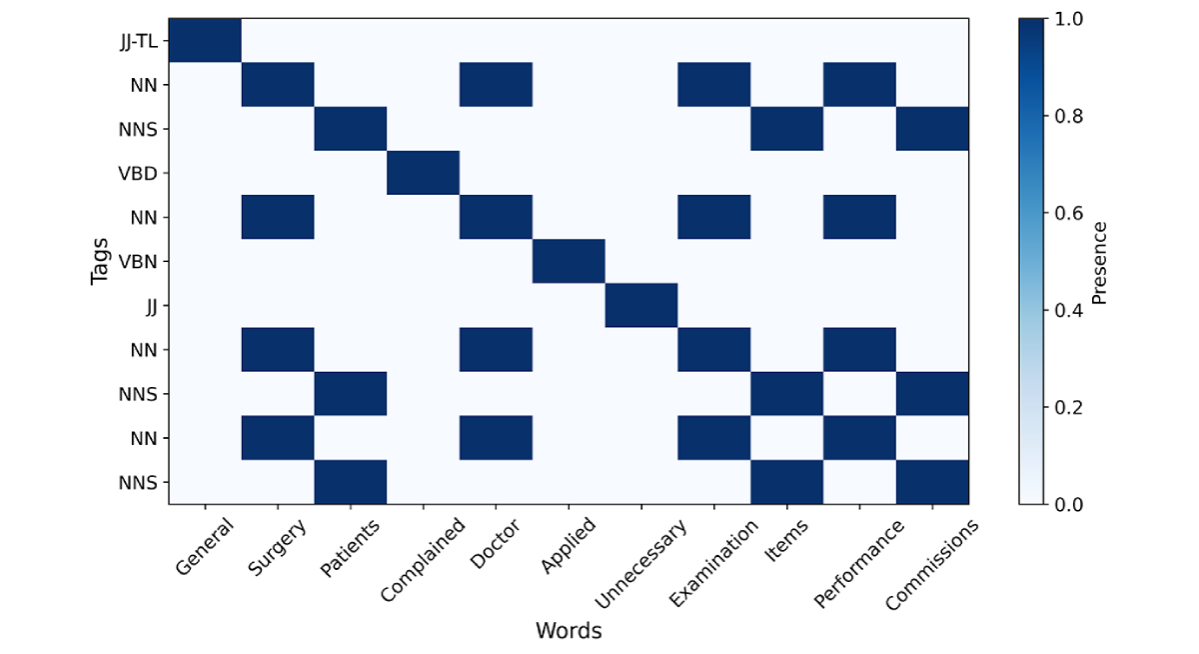
Illustration of a matrix with words and tokens as horizontal and vertical axes with the Hidden Markov Model Bigram token algorithm.

The following image (Figure 2) illustrates a matrix with words and tokens as horizontal and vertical axes with the HMM-Bigram algorithm.

**Figure 3:**
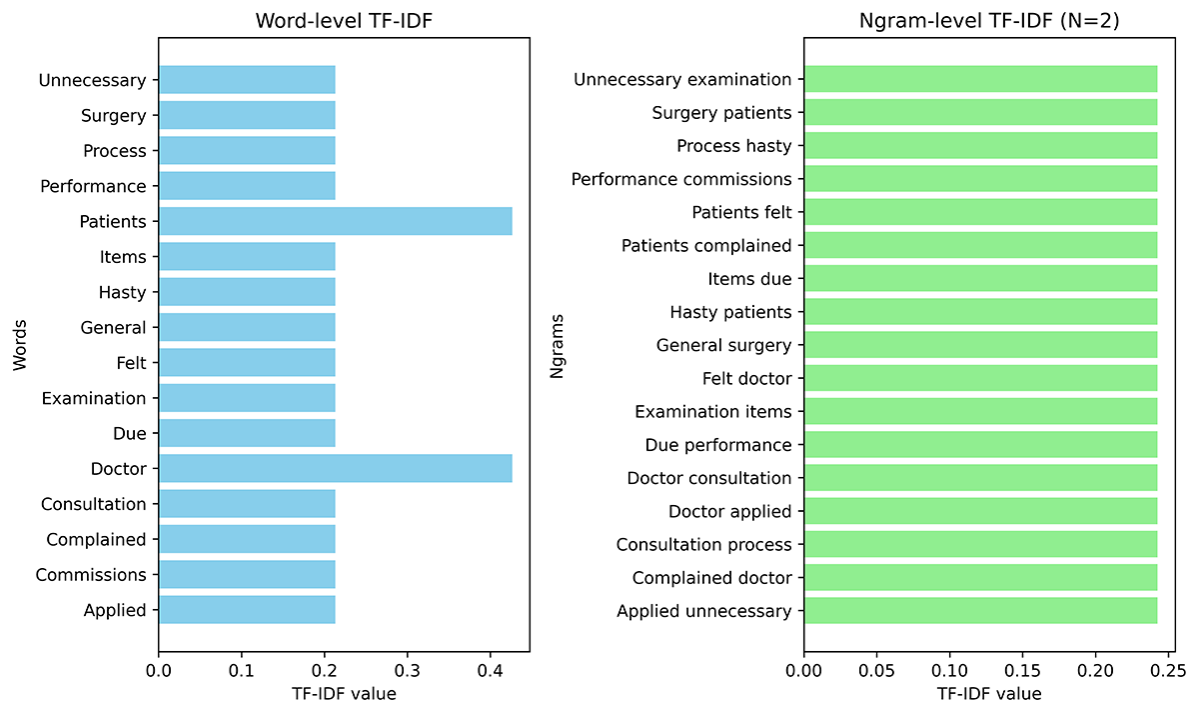
Comparison of the term frequency–inverse document frequency values between Word-level and Ngram-level term frequency–inverse document frequency (with n=2). TF-IDF: term frequency–inverse document frequency.

In the methodology section, 2 methods, Word-level TF-IDF and Ngram-level TF-IDF (with n=2), were used for feature extraction from the text. The TF-IDF values and their performance on the training and verification sets are shown in Figure 3 and Table 3, respectively.

**Table 3:** Classification results comparison of Word-level term frequency–inverse document frequency and Ngram-level term frequency–inverse document frequency (n=2).

	Precision			Recall			*F*_1_-score			Support	
	WL^a^	NL^b^	WL		NL	WL		NL	WL		NL
Communication problem	0.73	0.74	0.95		0.95	0.82		0.83	138		140
Management problem	0.93	0.93	0.89		0.88	0.91		0.91	189		189
Diagnosis and treatment	0.99	0.99	0.89		0.9	0.94		0.94	201		200
Sense of responsibility	1	1	0.94		0.94	0.97		0.97	192		191
Accuracy	—^c^	—	—		—	0.91		0.92	720		720
Macro average	0.91	0.92	0.92		0.92	0.91		0.91	720		720
Weighted average	0.93	0.93	0.91		0.92	0.92		0.92	720		720

^a^Word-level term frequency–inverse document frequency.

^b^Ngram-level term frequency–inverse document frequency.

^c^Not applicable.

### Semantic Analysis

A total of 4 complaint categories underwent preprocessing after incorporating expended stop words to enhance the specificity of medical complaints. This process generated high-frequency word histograms, and the word frequency analysis results for the 4 types of complaints will be uploaded as supplementary files (Multimedia Appendices). The top 3 vocabulary words in the category of “communication problem” are “explanation,” “enough,” and “believes.” In the category of “diagnosis and treatment” complaints, apart from “diagnosis and treatment” itself, the main concerns were “effect,” “pediatric,” and “emergency.” In the category of “management problem” complaints, the primary content revolves around “clinic,” “waiting,” and “time.” As for the category of “sense of responsibility,” the prominent content includes “medicine,” “back,” and “went.”

### Performance of Patient Complaint Classification Models

**Figure 4:**
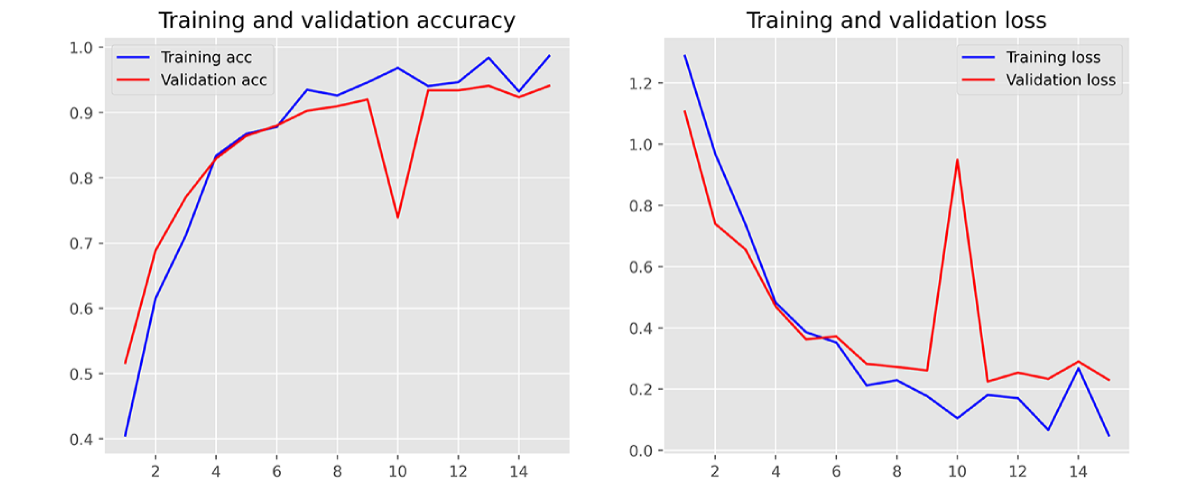
Demonstration of training and validation performance in machine learning iteration process. Training and validation performance of Support Vector Machine. acc: accuracy.

**Figure 5:**
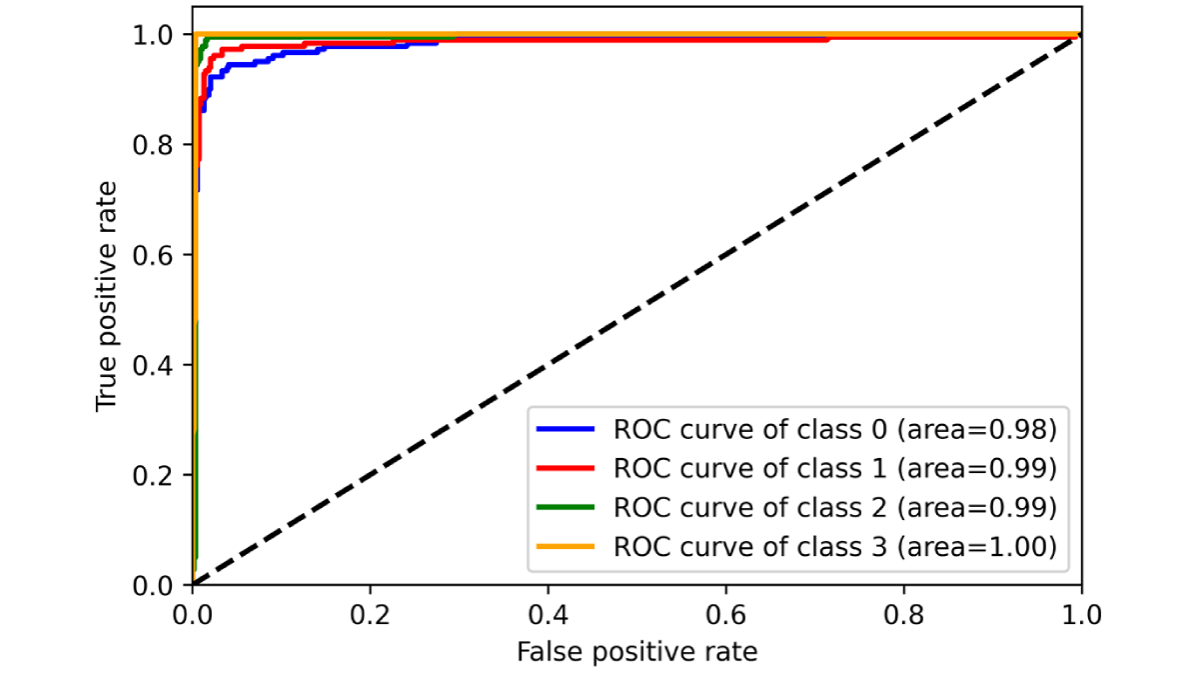
Training and validation performance of Support Vector Machine (receiver operating characteristic curve). ROC: receiver operating characteristic.

The performance of 3 ML models in the training and validation sets was demonstrated in Table 2 (for more detailed data including the 95% CIs for each metric, please refer to Multimedia Appendix 5). It was found that MLR achieved the best performance in classifying the communication problem, as assessed by *F*_1_-score. However, in the classification tasks of the remaining 3 categories, SVM outperformed MLR and MNB, comprehensively. Furthermore, from the training and validation iteration process, it can be observed that the model began to converge well by the eighth epoch (Figures 4 and 5). In addition, the best model achieved very high receiver operating characteristic (ROC) values (ROC>0.97) in classifying the 4 types on the test set. Hence, the SVM algorithm was adopted for the subsequent external data validation section with a 5-fold CV method applied. A more detailed comparison of the point estimate and 95% CIs for each model’s performance metrics has been included in Multimedia Appendix 6.

### Stability of Model Iteration Process

**Figure 6:**
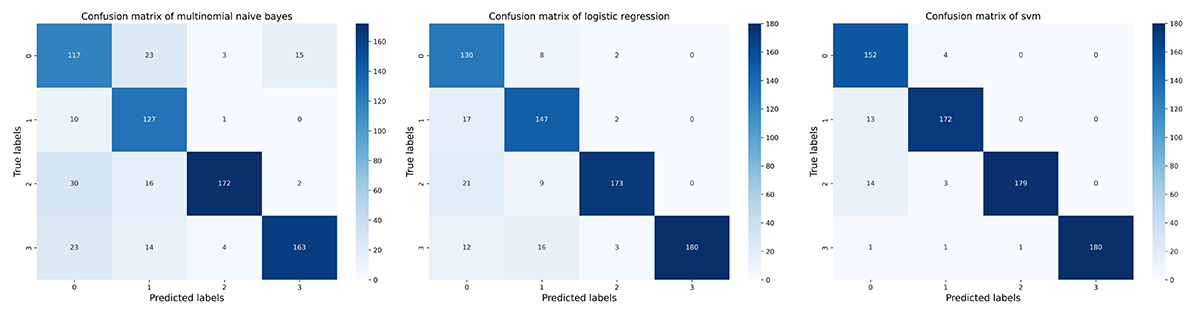
Demonstration of training and validation performance in machine learning iteration process. Confusion matrix of Multinomial Naive Bayes, Logistic Regression, and Support Vector Machine in testing set. svm: Support Vector Machine.

**Figure 7:**
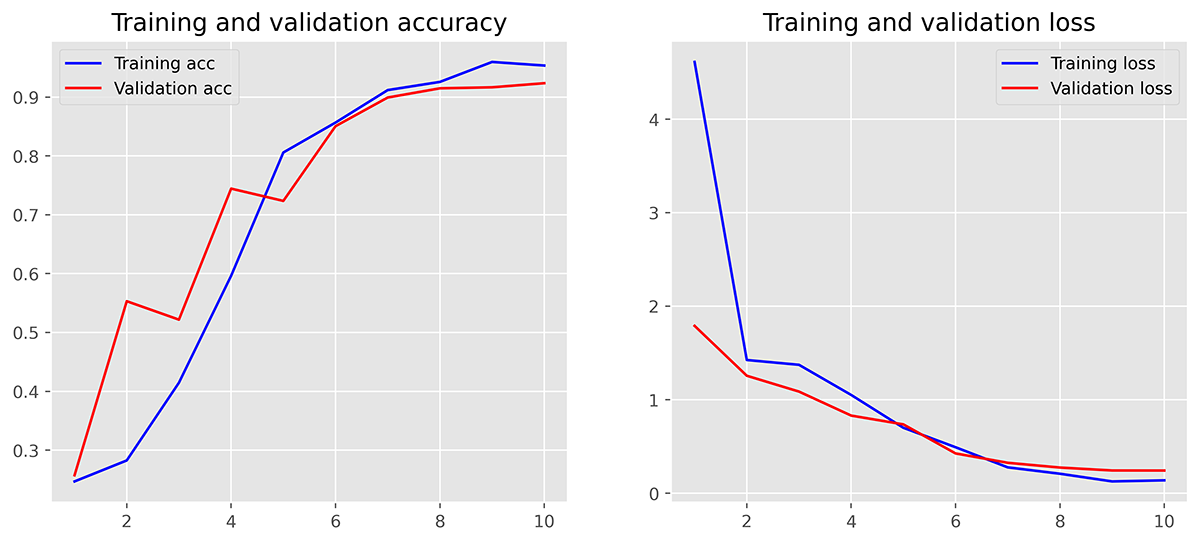
Demonstration of training and validation performance in machine learning iteration process. Training and validation performance of Multinomial Naive Bayes. acc: accuracy.

**Figure 8:**
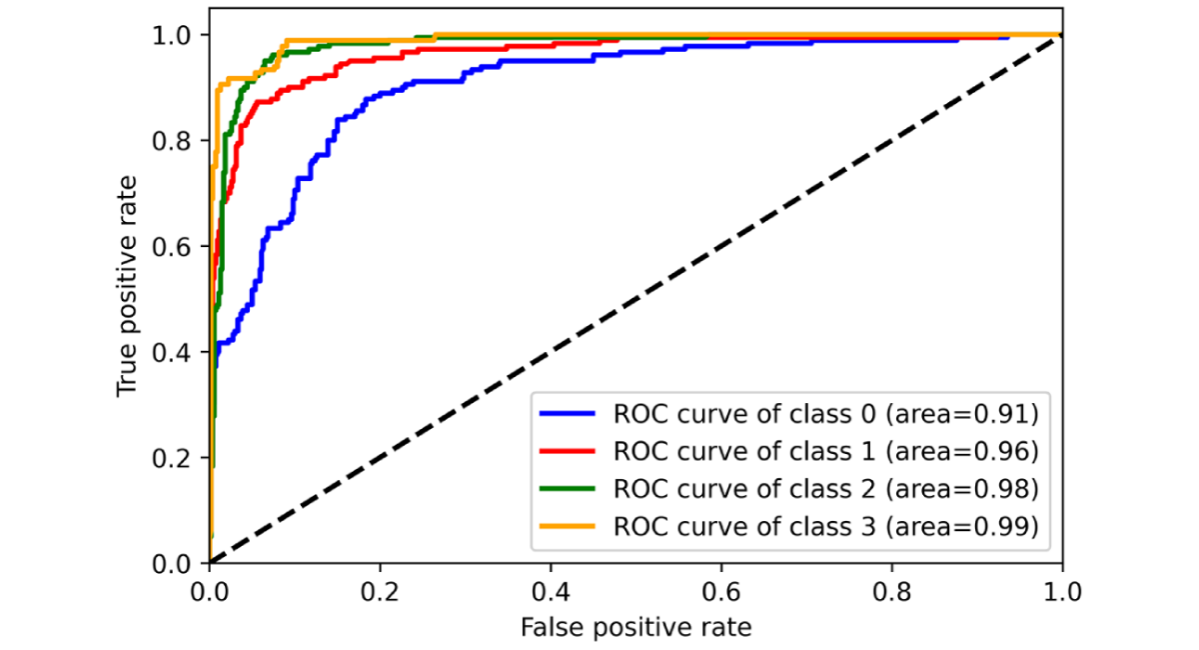
Training and validation performance of Multinomial Naive Bayes (receiver operating characteristic curve). ROC: receiver operating characteristic.

**Figure 9:**
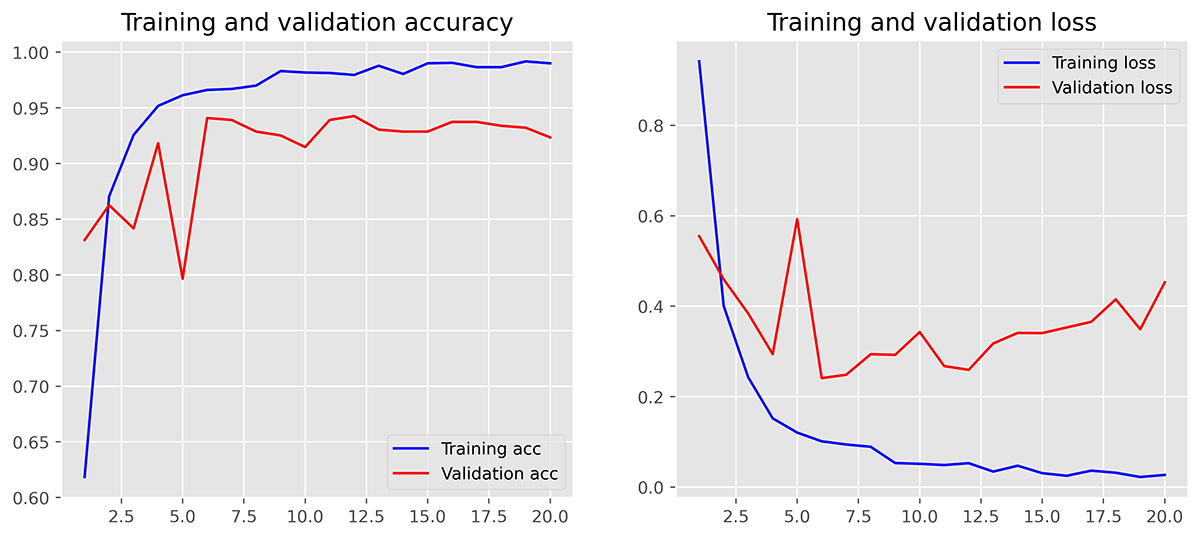
Demonstration of training and validation performance in machine learning iteration process. Training and validation performance of Multifactor Logistic Regression. acc: accuracy.

**Figure 10:**
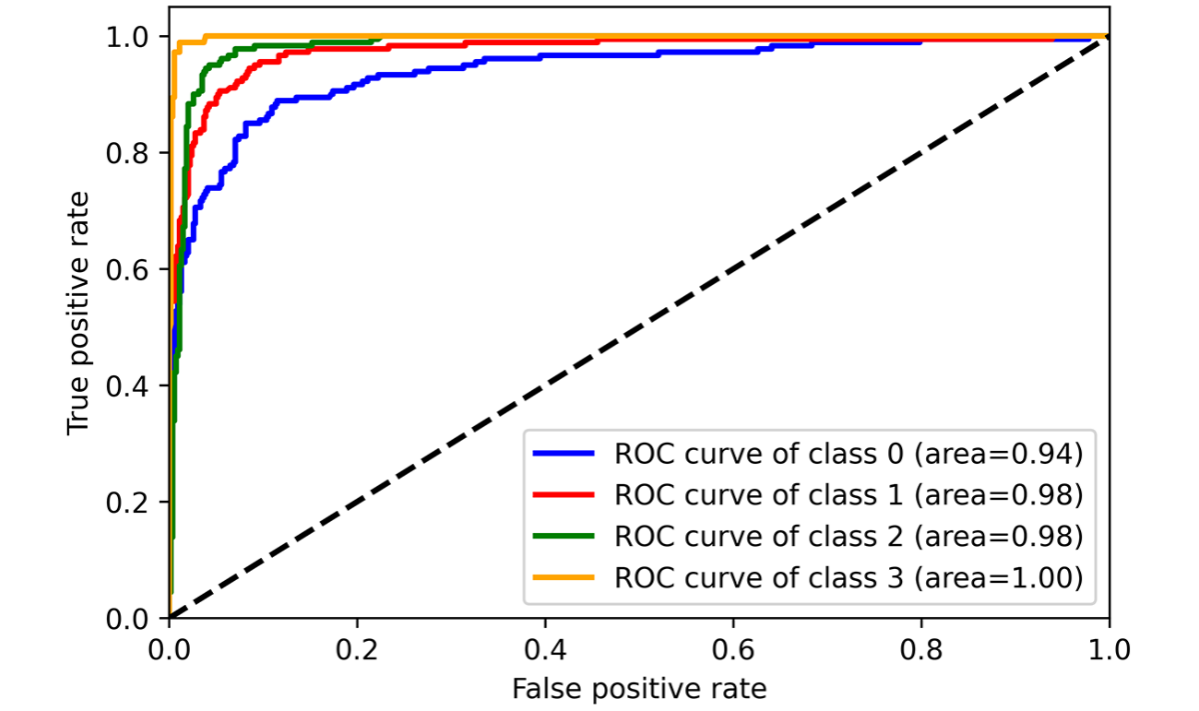
Training and validation performance of Multifactor Logistic Regression (receiver operating characteristic curve). ROC: receiver operating characteristic.

To monitor the stability of the model’s training effects during the machine-learning process, we set a maximum iteration number of 30. It was found that all ML algorithms perfectly converged in the first 20 iterations, and the MLR had the most stable output and the best generalization performance during the training and validation iterations (Figures 6-8). However, due to the use of the SGD (Stochastic Gradient Descent) algorithm, the SVM algorithm tended to output significantly fluctuating results during the training process (Figures 4 and 5). Although the MNB algorithm performed well on the training set, it exhibited overfitting and mediocre generalization ability on the test set (Figures 9 and 10).

### Performance of the 5-Fold SVMs Algorithm on the External Test Dataset

**Figure 11:**
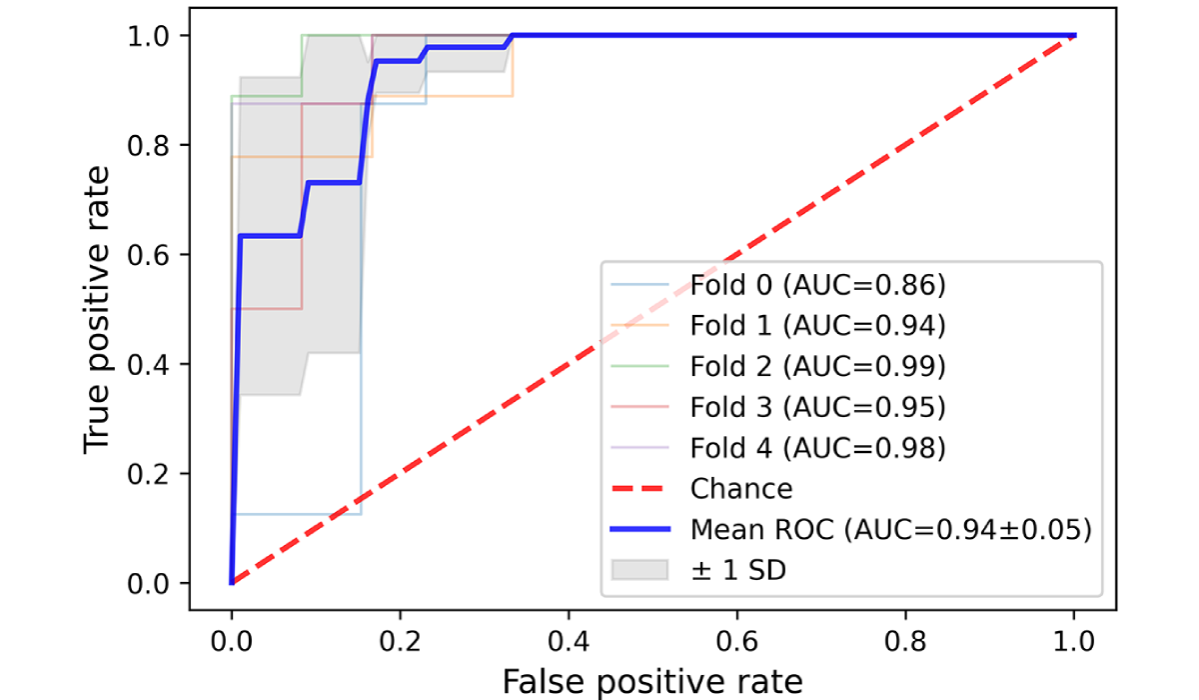
A 5-fold cross-validation curve of receiver operating characteristic of Support Vector Machine on the external test data set. Receiver operating characteristic for communication problem classification. AUC: area under the curve; ROC: receiver operating characteristic.

**Figure 12:**
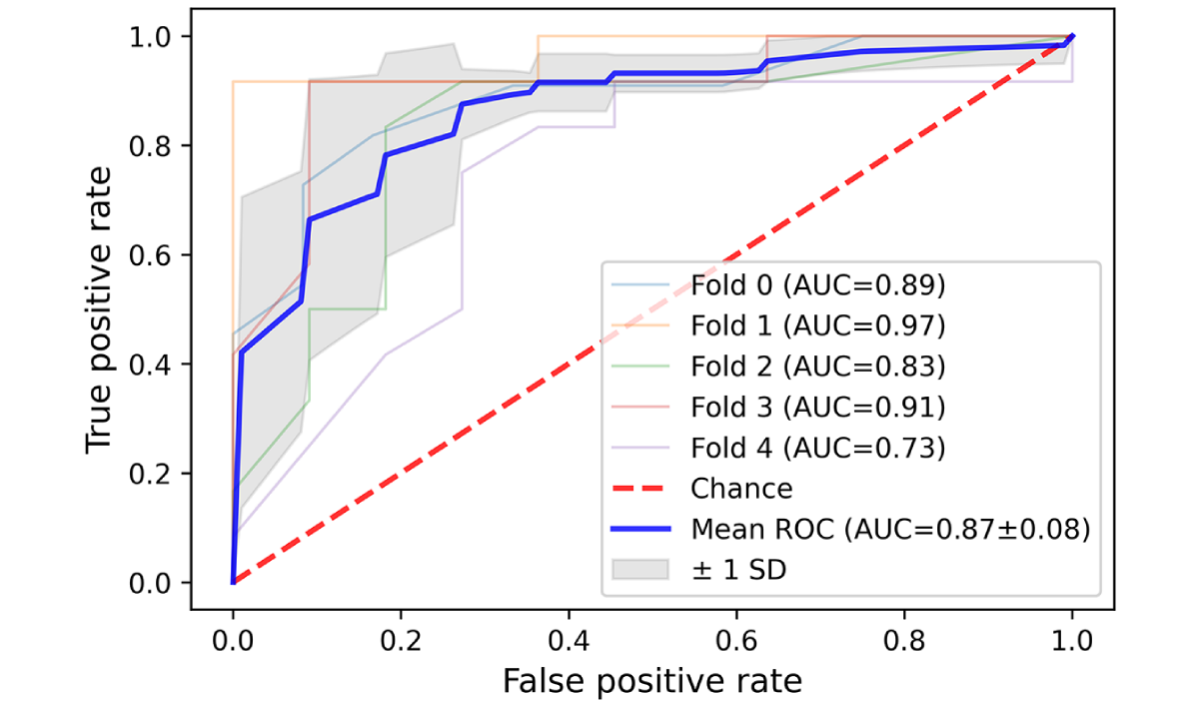
A 5-fold cross-validation curve of receiver operating characteristic of Support Vector Machine on the external test data set. Receiver operating characteristic for diagnosis and treatment problem classification. AUC: area under the curve; ROC: receiver operating characteristic.

**Figure 13:**
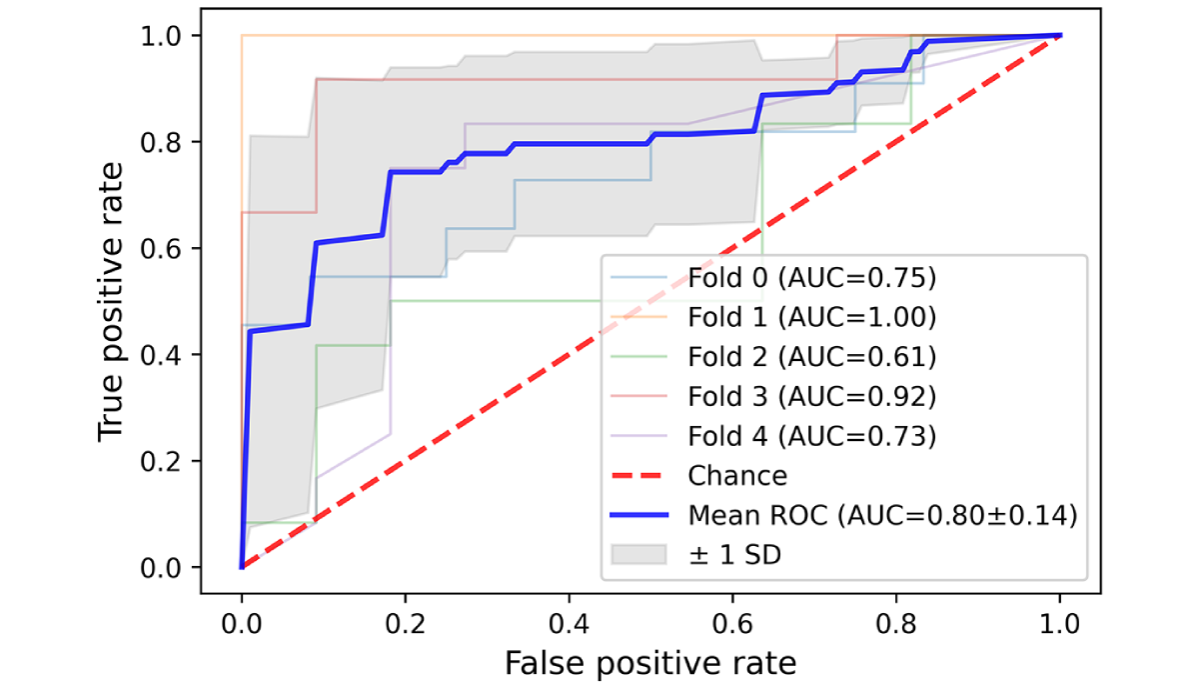
A 5-fold cross-validation curve of receiver operating characteristic of Support Vector Machine on the external test data set. Receiver operating characteristic for management problem classification. AUC: area under the curve; ROC: receiver operating characteristic.

**Figure 14:**
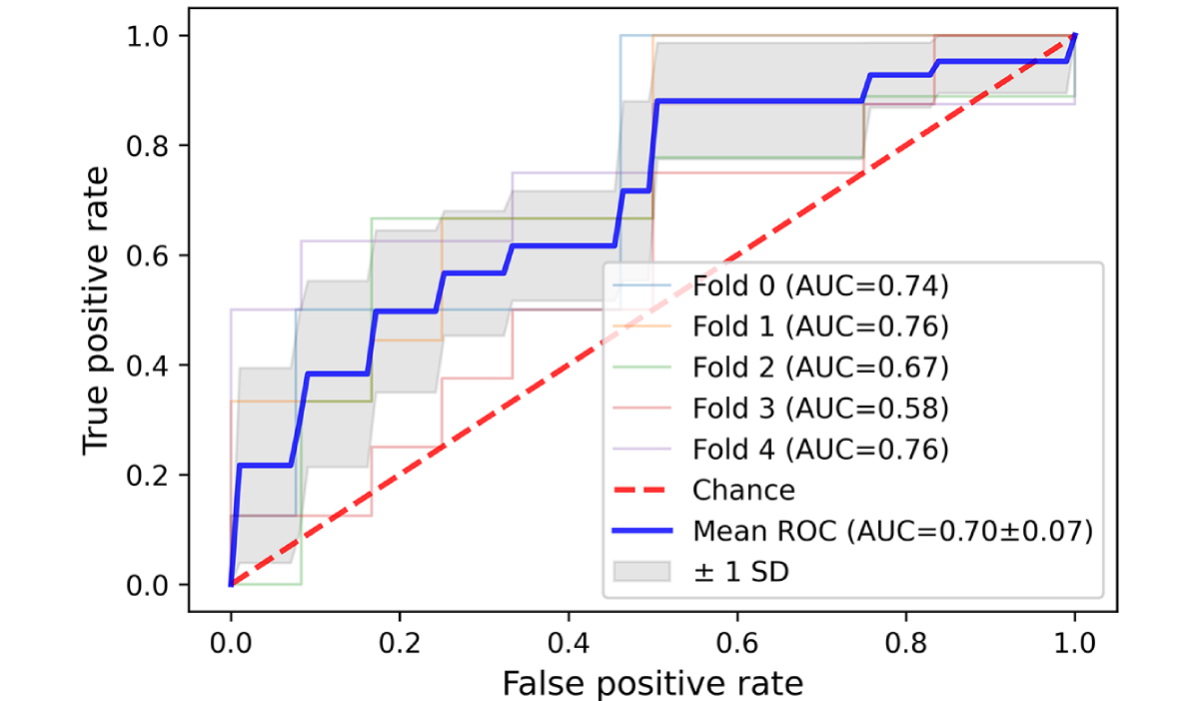
A 5-fold cross-validation curve of receiver operating characteristic of Support Vector Machine on the external test data set. Receiver operating characteristic for sense of responsibility problem classification. AUC: area under the curve; ROC: receiver operating characteristic.

It was inspiring to see that the SVM classification algorithm achieved a commendable result in accurately categorizing 3 distinct types of patient complaints (communication problem, diagnosis, treatment, and management problem) on the external test dataset. The average area under the curve (AUC) for these 3 types was 0.94, 0.86, and 0.81, respectively, with an average SD below 0.1 in all 3 types of patient complaints (Figures 11-13). However, the average AUC for the sense of responsibility category was only 0.7 (in the 5-fold testing, the minimum AUC is 0.48, and the maximum AUC is only 0.82; Figure 14).

## Discussion

### Principal Findings

Ambiguities arise more frequently in areas where patients may struggle to clearly articulate their concerns, particularly in subjective categories like communication and responsibility. Word-level TF-IDF proved sufficient for our needs. The result also revealed that Ngram-level methods did not significantly enhance model performance, even after optimal tuning of N. The NLP-driven SVM algorithm effectively classifies patient complaint texts, demonstrating superior performance in both internal and external test sets for communication and management problems.

### Related Systems for Patient Complaints Text Classification

Traditional text classification relies on hand-coded keyword-based methods suffer from low efficiency and accuracy. The manual creation of these classifiers (often in the form of expert rules) is a difficult and expensive process [43] that requires the coordinated work of medical experts and knowledge engineers. To the best of our knowledge, there are currently 3 manual approaches to classifying patient complaint texts: The first one is Patient Advocacy Reporting System (PARS), the second mainly focused on Healthcare Complaint Analysis Tool (HCAT [44]), developed by UK researchers Alex Gillespie and Tom Reader, and the last one is based on the use of the qualitative analysis software NVivo [45,46], launched by the Australian company QSR (now Lumivero). However, both HCAT and NVivo require human classification at various stages of the classification process. Therefore, these methods are at best semiautomatic means for text classification. They have not yet fully solved the problem of manual intervention in the classification process [47].

### Ngram-Level TF-IDF Failed to Enhance Classification Performance

As gleaned from Table 3, the increment in precision, recall, and *F*_1_-score with Ngram-level TF-IDF (n=2) merely amounted to 1%, rising from 0.91 to 0.92. The reason analysis for why Ngram-level TF-IDF (n=2) did not improve the classification performance of Word-level TF-IDF could be as described further. First, the primary reason might be Ngram-level TF-IDF with n=2 considers pairs of words, capturing some contextual information. However, this may not always lead to significant improvements, especially when text prone to ambiguity undergoes review by language experts, the additional information obtainable from conjunctions becomes relatively limited. The second possible reason was Ngram-level TF-IDF, particularly with smaller datasets (only 2157 cases for training), which could be prone to overfitting. It might capture too much noise or specific patterns from the training data that did not generalize well to unseen data, resulting in worse performance on the test set. For tuning the parameter of Ngram-level TF-IDF, we used the MNB method and set N to values ranging from 1 to 5. The results were depicted in Multimedia Appendix 7. It can be observed that when N=1, the model achieved its maximum values for precision, recall, and *F*_1_-score. Consequently, N=1 for Ngram-level TF-IDF corresponds to using Word-level TF-IDF.

### The Feasibility of Using ChatGPT as a Bulk Translation Tool

The process of translating patient complaints into English through ChatGPT could potentially introduce several issues into the classification, especially concerning the accuracy of translation. Common knowledge holds by European and American litterateurs that translating ancient Chinese poetry into English is extremely challenging. However, in a study reported by Gao et al [48], they compared the performances of ChatGPT with Google Translate and DeepL Translator in translating Chinese classical poetry in terms of fidelity, fluency, language style, and machine translation style. The results revealed that ChatGPT outperformed Google Translate and DeepL Translator in all evaluation criteria. Therefore, in our research, the difficulty level of the Chinese text of patient complaints was significantly lower compared with classical poetry. The texts were generally very clear and complete, containing specific elements and structures such as precise time, location, individuals involved, and events. As the complainants were patients and their family members, the language was relatively common, devoid of esoteric medical terminology. Consequently, for ChatGPT 3.5, the translation results were very close to the outcomes of human translations. In addition, 2 postgraduates with English language skills were responsible for reviewing the machine-translated texts. If there were obvious translation errors, the texts were corrected after the 2 individuals proposed unanimous modification suggestions. Therefore, we believe that at least using ChatGPT 3.5 for translating bulk of simple complaint texts into English was acceptable and efficient, as we did not have to struggle with balancing the accuracy and efficiency of the translation.

### Appropriate Number and Types of Categories

Reader et al [49] developed a coding taxonomy for analyzing patient complaints. The subcategories were thematically grouped into 7 categories and then 3 conceptually distinct domains. Their “3-domain” classification system is similar to our “4-category” one. For instance, “the safety and quality of clinical care” corresponds to our “diagnosis and treatment,” “management of health care organizations” corresponds to our “management problem,” and “healthcare staff-patient relationships” corresponds to our “communication problem” and “sense of responsibility.”

We subdivided “healthcare staff-patient relationships” into 2 categories because it was considered that communication issues were skill-related problems that can be addressed through targeted training. At the same time, the sense of responsibility is a matter of the staff’s character that requires long-term education and may even necessitate stricter rewards and penalties to improve. They also reported that the most common issues complained about were “treatment” (113,738/88,069, 15.6%) and “communication” (12,065/88,069, 13.7%). However, our research shows that complaints mainly stem from “communication” (719/1465, 49.9%) and “diagnosis and treatment” (376/1465, 26.1%), respectively. However, when focusing on categorizing complaints, we must analyze the data sources, as there can be significant differences between patient complaint data from specialized hospitals and data from comprehensive hospitals. Even within the same hospital, there can be significant variations in complaint data between different departments.

Another similar research carried out by HaCohen-Kerner et al [50], showed that their study attempted to reduce the number of categories for automatic text classification from 7 to 4 across 2073 samples. The predictive model’s performance reached its peak accuracy (93.8%) on the test set when the number of categories was reduced to 4. Therefore, it was reasonable in our research to limit the predicted categories to 4 in similar sample sizes.

### Optimal Number of Folds to Use in a K-Fold Cross-Validation in External Dataset

It is well-accepted that larger k means less bias toward overestimating the true expected error but higher variance and higher running time. In both of these cases, selecting k depends on the same thing. We must ensure that the training set and testing set are drawn from the same distribution and that both sets contain sufficient variation such that the underlining distribution is represented. The problem we encountered involved using training and verification data sourced from a general hospital and external test data from a specialized cancer hospital. Discrepancies in patient treatment expectations and compliance between the 2 hospitals may introduce biases in the distribution of complaint data. Consequently, we opted to use CV testing during external testing to mitigate the potential impact of these factors on data representation.

Selecting k is not an exact science because it is challenging to find a balance in a fold of data that effectively represents the overall dataset while also minimizing data bias. We try to use a 5-fold cross validation, in this case, with approximately 75 (376×0.2) instances per fold, conducting 10-folds CV would result in only about 37 instances per fold. As a result, all 4 types of complaints may not be encompassed within a single fold. However, if our dataset size increases dramatically, like if we have over 10,000 instances, a 10-fold cross validation would lead in folds of 1000 instances. In a related work carried by Oyedele [51], a bigger independent testing set of size 10,000 (independently sampled from the same distribution and using the same parameters with 5000 samples per class) was created in order to estimate the performance (ie, error probability) of the network. Therefore, for a task with 2 classifications and 10,000 samples, they adopt a 10-fold CV, which poses much less risk of bias in the results compared with our 5-fold CV approach.

### Advantages of Using TF-IDF and SVM for Patient Complaint Text Classification

Elmessiry et al [52] compared a text classification method using 6 ML classifiers and suggested that the TF-IDF–based random forest classification algorithm has advantages in text classification both in accuracy and *F*_1_-score. However, based on their results, the achieved accuracy, *F*_1_-score, sensitivity, and specificity in correctly classifying patient complaints were relatively low, with values of 82%, 81%, 0.76, and 0.87 respectively.

Previous studies have used ML algorithms such as SVMs [53], Logistic Regression [54], and Naive Bayes classifiers [55] for classification purposes [56]. Compared with other ML algorithms, the SVM algorithm has demonstrated superior performance in text classification, which aligns closely with our research findings. The strong generalization capability of SVM in high-dimensional feature spaces can be credited for their effectiveness in text classification tasks. SVMs eliminate the need for feature selection, simplifying the classification process. Their regularization parameters help prevent overfitting, making them robust in handling noisy data and datasets with many features. In addition, SVMs are efficient in processing small to medium-sized datasets, producing reliable performance even with limited training data. Our research also confirmed that, even with very limited sample sizes, such as in the cases of “management problem” and “sense of responsibility” with raw data sizes of only 260 and 86, the SVM algorithm achieved average AUC scores of 0.81 and 0.7, respectively, in external test sets.

In the text preprocessing and ML section, we used the pipeline training architecture, which can be seen as a tool to connect multiple data processing and model training steps. It allows users to combine multiple operations in a specific order to form a complete flow and ensures data flows between each operation. Through pipeline, data preprocessing, feature engineering, model training, model evaluation, and other steps can be organically combined into a complete ML process. The advantage of doing this is improving code maintainability and reusability, making it easier to perform the same workflow on different datasets and better managing dependencies between each step.

We initialized SVM parameters as listed in Multimedia Appendix 8 and adopted a fine-tuning with genetic algorithm–SVM as proposed by Ali et al [57] and Nair et al [58], which has the best model performance while keeping a reasonable optimization time. The detailed tuning process and prediction results before and after tuning have been illustrated in Multimedia Appendices 9 and 10).

### ML Model Selection Strategy

A critical consideration is the balance between model accuracy and model simplicity or interpretability. High accuracy is essential for a model’s effectiveness, especially in scenarios that rely on making correct predictions. While complex models (such as DL or ensemble methods) can achieve higher accuracy due to their ability to capture intricate patterns in the data, they often require large amounts of data and substantial computational resources. Models with high interpretability foster trust and accountability, as stakeholders can see and understand how decisions are made. However, there is a trade-off here; complex models may exhibit lower bias but higher variance, meaning they might fit the training data very well but perform poorly on unseen data due to overfitting. Conversely, simpler models tend to have higher bias, potentially missing significant patterns in the data, but they perform more consistently across different datasets.

In this study, a hybrid strategy was adopted in ML model selection. In the first stage, 3 ML algorithms (MLR, MNB, and SVM) were used for model training and validation. In the second stage, the best-performing model was tested using 5-fold CV on external data. Ultimately, the choice of model should be guided by the specific context of the problem, including the importance of accuracy versus interpretability, the stakes involved, and the audience for the model outputs. In the specific task of patient complaint classification, we prioritize the generalization ability of the model, which means achieving the best prediction accuracy on unseen data. This preference is crucial because if the model’s generalization is insufficient, misclassified complaint cases will require more human effort to resolve, and the resulting delay in complaint resolution might exacerbate patient-doctor conflicts and disputes.

### Limitations and Further Research Directions

In this study, the original data were initially in Microsoft Excel format, and each row of complaint content was separately saved as a text file before subsequent data cleaning and analysis were performed. Consequently, similar processing was required during application to ensure compatibility with the model. Failure to align the actual input data format with that of the model could lead to the inability of the model to predict outcomes correctly. Therefore, in subsequent work, a complaint text organization code will be developed to support both individual complaint text prediction through copy-and-paste operations and batch text processing.

It is noteworthy that despite ultimately selecting the SVM algorithm for validating external data, it displayed consistent fluctuations in learning accuracy and verification loss throughout in training iteration. We attempted to improve the situation by reducing the learning rate from 0.5 to 0.1, but this did not yield positive results. After ruling out factors such as randomness and model complexity, we speculate that it is likely due to the SVM algorithm using SGD optimization, which can cause the model to converge to local minimum or exhibit more significant fluctuations in results. Another possibility may be the imbalance in our raw data distribution, with the third class, “management problem,” and the fourth class, “sense of responsibility,” having too few data samples.

The primary challenge addressed in this study pertains to the imbalanced distribution of raw data and the limited dataset size. Although we used SMOTE to adjust for imbalanced data, the synthetic samples generated by SMOTE may introduce noise or distortions into the dataset, potentially leading to information loss and affecting the classifier’s performance on the minority class. To address this issue, our future work will involve collaborative efforts with multiple hospitals to enhance data volume. Furthermore, as is well-known, more advanced NLP models (eg, BERT) provide contextual understanding, enabling them to grasp complex relationships within text and improve analysis accuracy and depth. As a future direction for our work, we plan to develop a user-friendly graphical user software interface powered by BERT to facilitate the classification and management of complaints by PAC personnel.

Another issue that must be stated is that the semantic features extracted using NLP technology are quite different from the common scenarios based on explicit independent and dependent variables. In ML problems with clear relationships between independent and dependent variables, it is possible to rank the importance of independent variables, as these features have stable meanings themselves. However, semantic features are influenced by the linguistic context, meaning that the same word can have different meanings in different language environments and contexts. Therefore, our results cannot pinpoint which specific words significantly influence the classification of complaint text types.

### Conclusion

The NLP combined SVM algorithm performs well in automatically categorizing patient complaint texts. Furthermore, it has the best performance in both internal and external test sets for communication and management problems. However, caution is necessary when used for the classification of sense of responsibility. It has excellent prospects for application in medical institutions with many complaints and a shortage of medical-patient processing specialists.

## Appendix

Multimedia Appendix 1 Review and Prospect of Multi-Label Text Classification Research.

Multimedia Appendix 2 A method and system for classifying short text of product names based on attention mechanism: Part a.

Multimedia Appendix 3 A method and system for classifying short text of product names based on attention mechanism: Part b.

Multimedia Appendix 4 High frequency but meaningless stop words related to medical complaints.

Multimedia Appendix 5 Comparison of the Performance of Three Machine Learning Algorithms: Point Estimate and 95% Confidence Intervals.

Multimedia Appendix 6 Point Estimate and 95% CIs of each model’s performance metrics in distinguishing the four categories.

Multimedia Appendix 7 Precision, Recall, and F1-score on different N in Ngram-level TF-IDF (term frequency–inverse document frequency).

Multimedia Appendix 8 Initialized Support Vector Machine parameters.

Multimedia Appendix 9 The fine tuning hyperparameters.

Multimedia Appendix 10 The fine tuning hyperparameters.

Multimedia Appendix 11 Core Python code used in this study.

## Abbreviations

AUC: area under the curve
BERT: Bidirectional Encoder Representations from Transformers
BiGRU: Bidirectional Gated Recurrent Unit
BiLSTM: Bidirectional Long Short-Term Memory
BOW: bag-of-words
CC: chief complaints
CNN: convolutional neural network
CV: cross-validation
DL: deep learning
ED: emergency department
GRU: gated recurrent unit
HMM-Bigram: Hidden Markov Model Bigram
HCAT: Healthcare Complaint Analysis Tool
LSTM: long short-term memory
ML: machine learning
MLR: Multifactor Logistic Regression
MNB: Multinomial Naive Bayes
NLP: natural language processing
PAC: Patient Advocacy Center
PARS: Patient Advocacy Reporting System
RNN: recurrent neural networks
ROC: receiver operating characteristic
SGD: Stochastic Gradient Descent
SMOTE: Synthetic Minority Oversampling Technique
SVM: Support Vector Machine
TF-IDF: term frequency–inverse document frequency

## Data Availability

Data that support the findings of the study are available upon request from the corresponding author.

In addition, we have provided the important Python code related to this study in Multimedia Appendix 11 for interested readers to download and use.
