# Supplementary material for: An Intelligent System for Classifying Patient Complaints Using Machine Learning and Natural Language Processing: Development and Validation Study
Source: J Med Internet Res. 2025 Jan 8;27:e55721. doi: 10.2196/55721 (PMC11754990; doi:10.2196/55721)
Supplement: Multimedia Appendix 1 [file jmir_v27i1e55721_app1.pdf]

多标签文本分类研究回顾与展望

张文峰[1,2]，奚雪峰[1,2,3]，崔志明[1,2,3]，邹逸晨[1,2]，栾进权[1,2]
1.苏州科技大学 电子与信息工程学院，江苏 苏州 215000
2.苏州市虚拟现实智能交互及应用技术重点实验室，江苏 苏州 215000
3.苏州智慧城市研究院，江苏 苏州 215000

摘 要：文本分类（TC）是自然语言处理（NLP）领域的重要基础任务，多标签文本分类（MLTC）是TC的重要分支。为了对多标签文本分类领域进行深入了解，介绍了多标签文本分类的概念和流程。将近年来多标签文本分类方法划分为基于传统机器学习方法和基于深度学习方法，梳理了多标签文本分类领域常用的数据集和评价指标，分析了部分多标签文本分类模型的优势和存在问题。介绍了多标签文本分类的研究方向：标签相关性、特定标签特性、类别不平衡、标签丢失和标签压缩。对多标签文本分类的难点和未来的发展方向进行了总结展望。

关键词：多标签文本分类；深度学习；标签相关性；特定标签特性；类别不平衡
文献标志码：A **中图分类号：**TP391 doi：10.3778/j.issn.1002-8331.2210-0446

Review and Prospect of Multi-Label Text Classification Research

ZHANG Wenfeng[1,2], XI Xuefeng[1,2,3], CUI Zhiming[1,2,3], ZOU Yichen[1,2], LUAN Jinquan[1,2]
1.School of Electronic & Information Engineering, Suzhou University of Science and Technology, Suzhou, Jiangsu 215000, China
2.Suzhou Key Laboratory of Virtual Reality Intelligent Interaction and Application Technology, Suzhou, Jiangsu 215000, China
3.China Suzhou Smart City Research Institute, Suzhou, Jiangsu 215000, China

Abstract： Text classification（TC）is an important basic task in the field of natural language processing（NLP）, and multi-label text classification（MLTC）is an important branch of TC. In order to have a deep understanding of the field of multi-label text classification, the concept and process of multi-label text classification are introduced. In recent years, multi-label text classification methods are divided into traditional machine learning methods and deep learning methods. The commonly used data sets and evaluation indexes in the field of multi-label text classification are sorted out, and the advantages and problems of some multi-label text classification models are analyzed. The research directions of multi-label text classification：label correlation, specific label characteristics, category imbalance, label loss and label compression. Finally, the difficulties of multi-label text classification are summarized and the future development direction is prospected.

Key words： multi-label text classification; deep learning; label correlation; features label-specific; class imbalance

文本分类是将文本内容划分为一个或多个类别的过程，是NLP中的一个基础任务。在现实世界中，由于文本数据环境复杂多变以及多义对象的存在，文本分类面临诸多严峻挑战。传统的单标签文本分类方法并不能完全满足用户的需求，多标签学习方法应运而生[1]。多标签学习是指从标签集中将最相关的类标签分配给每个文本的过程，从而直观地反映模糊对象的各种语义信息内容。例如，一篇关于2019冠状病毒病（“COVID-19”）的新闻报道会属于“医疗卫生”类别和“经济危机”或“国家安全”类别等多个类别。

多标签文本分类问题是多标签学习的重要研究方向，主要应用于情感分析[2]、主题标注[3]、问答[4]和对话行

基金项目：国家自然科学基金（61876217，62176175）；江苏省“六大人才高峰”高层次人才项目（XYDXX-086）；苏州市科技计划项目（SGC2021078）。

作者简介：张文峰（1999—），男，硕士研究生，CCF学生会员，研究方向为自然语言处理、文本分类，E-mail：2906213721@qq.com；奚雪峰（1978—），通信作者，男，博士，教授，CCF会员，研究方向为自然语言处理、多模态机器学习、软件工程；崔志明（1961—），男，博士，教授，CCF会员，研究方向为知识挖掘、机器学习；邹逸晨（2001—），男，CCF学生会员，研究方向为自然语言处理；栾进权（2001—），男，研究方向为软件工程。

收稿日期：2022-10-27 **修回日期：**2023-02-20 **文章编号：**1002-8331（2023）18-0028-21

计算机工程与应用 www.ceaj.org

为分类[5]。其文本数据具有以下特点：一个文本可属于多个标签，因此需要捕获语义特征的不同层次和方面；文档较长时，语义信息会隐藏在冗余的内容中；大多数文本只属于少量标签[6]；文本数据不平衡、标签丢失以及标签集过于庞大。基于上述问题，研究人员主要关注几个方面：如何充分应用标签的相关性；如何捕获有效信息并提取相关的特征信息；以及如何减缓类别不平衡、标签丢失、标签压缩的问题。

本文主要的主要贡献归纳如下：

（1）对多标签文本分类概念和流程进行阐述。

（2）对近年多标签文本分类方法进行回顾，梳理多标签文本分类常用数据集和评估指标；以及对部分模型或方法分析其优势和存在问题。

（3）对多标签文本分类领域的研究方向进行整理回顾。

（4）对多标签文本分类当前的难点和未来研究方向进行总结和展望。

1　多标签文本分类

1.1　多标签文本分类概念

给定一个 d 维输入空间 $\mathfrak{X}=X_1\times X_2\times\cdots\times X_d$ 和一个输出 q 标签的空间 $\mathcal{Y}=\{\lambda_1,\lambda_2,\cdots,\lambda_q\}, q>1$ 。每个标签 $|\lambda_i|=2$ 的基数，一个多标签示例可以定义为一对 (x,Y) ，其中 $x=(x_1,x_2,\cdots,x_d)\in\mathfrak{X}, Y\in\mathcal{Y}$ 被称为标签集。 $\mathfrak{D}=\{(x_i,Y_i)|1\leqslant i\leqslant m\}$ 是由一组 m 个文本构成的多标签数据集。

多标签文本分类是构建一个预测模型 $h:\mathfrak{X}\to 2^{\mathcal{Y}}$ ，该模型将为文本提供一组相关标签。每个文本可能具有来自先前定义的标签集的与它相关联的几个标签。因此，对于每个 $x\in\mathfrak{X}$，有一个标签空间 $Y\in\mathcal{Y}$ 的二分区 $(Y,\bar{Y}), Y=h(x)$ 是相关标签集合， $\bar{Y}$ 是不相关标签集合。多标签文本分类如图1所示。

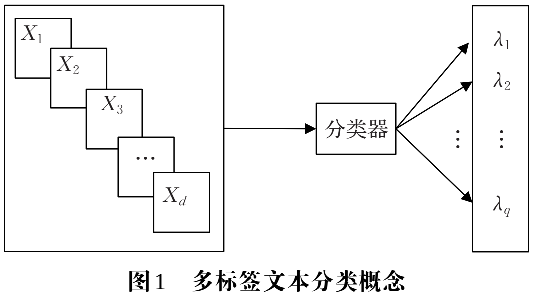

图1　多标签文本分类概念

Fig.1　Multi-label text classification concept

1.2　多标签文本分类流程

多标签文本分类的流程如图2所示。

（1）数据集

多标签文本分类领域常用数据集在第3章会有详细介绍。

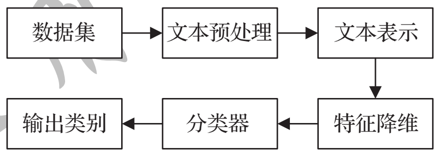

图2　多标签文本分类流程

Fig.2　Multi-label text classification process

（2）文本预处理

文本预处理是指对原始数据集进行去除停止词、分词、词性恢复等一系列操作，但目前对于上述处理已有非常成熟的技术。如果需要分词，可以直接使用jieba、HanLP[7]等现成的工具，研究人员不需要在这项研究上花费太多精力。

（3）文本表示

文本表示是自然语言处理领域的基石。由于机器无法直接识别自然语言，因此将自然文本转换为机器可以理解的表达式是文本表示的工作。文本表示的生成可以理解为按照一定的模型对文本数据进行编码，其发展大致经历了几个阶段，如One-hot、词袋（bag of words，BOW）、语言模型（LM）、Word2Vec[8]、Glove[9]等。One-hot表示通过二进制编码生成单词向量，每个维度仅指示字典中对应的单词是否在该位置取，该方法不仅会带来维数灾难，导致数据稀疏，还会造成文本语义的特征提取不足；BOW在一元热的基础上用词频数据替代二进制数据，但仍未能解决维数灾难和语义丢失的问题；LM模型使用条件概率来表达文本序列中单词之间的关联，但LM模型的语义表示方法比较原始，因此已经发展到Word2Vec模型。Word2Vec模型由连续袋词模型（CBOW）和Skip-Gram模型组成。Word2Vec模型使用类似于神经网络的结构来建立词之间关系的过程。近年来兴起的文本表示方法专注于上下文的词嵌入，如语言模型嵌入（ELMo），生成式预训练（GPT）[10]方法和BERT[11]的用双向编码器进行文本表示。ELMo首先通过语言模型学习每个单词的单词嵌入，对上下文动态调整嵌入，解决了一词多义问题，同时实现语义关系判断的功能。在特征提取方面，ELMo采用了LSTM，但后来提出了一种新的特征提取器Transformer，并且特征提取能力被证明优于LSTM。因此，基于Transformer作为特征处理提出了GPT。GPT模型通过语言模型预训练，然后进行微调进行文本表示；但是，GPT是一种单向语言模型，只注意词的上文，而不考虑词的下文，所以它在语义理解上并不全面。为了同时考虑两个词序方向上的语义信息，提出了BERT针对大型数据集的训练，从而可以学习更合理的词表征，包括了上下文信息[12]。

（4）特征降维

向量化处理后文本特征较为稀疏，维度比较高。特征降维常用方式有TF-IDF[13]和互信息等。而在Transformer提出后，大多数都采用Transformer用作特征降维模块。

计算机工程与应用 www.ceaj.org

（5）分类器和输出类别

将特征降维后的数据送入分类器中进行模型训练，然后用测试集对模型的输出类别进行预测，用验证集和评估指标来评判模型的优劣。

2　多标签文本分类方法

多标签文本分类方法主要可以分为：基于传统机器学习和基于深度学习。传统的机器学习方法根据解决策略角度可以划分为问题转换方法和算法自适应方法。问题转换方法是将多标签问题转化为多个单标签子问题，然后这些子问题直接利用成熟的单标签算法来解决，所以问题转换方法独立于具体的算法，可以根据实际选择合适的算法。算法自适应方法是将现有的单标签算法进行拓展，使其能够直接应用到多标签数据上。多标签文本分类方法详细的分类如图3所示。

2.1　问题转换方法

目前，问题转换算法主要基于以下三种方法：（1）二进制相关性（BR）转换方法，（2）标签幂集（LP）转换方法，以及（3）成对方法（PW）。

2.1.1　BR转换方法

BR是最具代表性的问题转换模型，它为每个标签建立了二元分类模型，但基本的BR模型[14]忽略了标签相关性。为了利用BR框架中标签之间的这种相关性，常见的方法是将标签作为额外的特征添加到原始特征中，然后构建相应的分类器，这些改进的BR模型可以分为两种类型。第一种类型是构造两层BR，第一层显示了与原始BR相同的做法，在第二层中，第一层的输出作为额外特征添加到原始特征中；然后，基于这些增强的特征，学习每个标签的二进制分类器。第二层的输出用作预测标签，使用这种方式的BR模型称为基于堆叠的BR模型。第二种类型是将所有二进制分类器连接成一个链，链上的一个分类器将所有先前分类器的输出作为额外的特征添加到原始特征中，这样的框架称为分类器链模型。下面详细介绍了BR模型、基于堆叠的BR模型和分类器链模型。

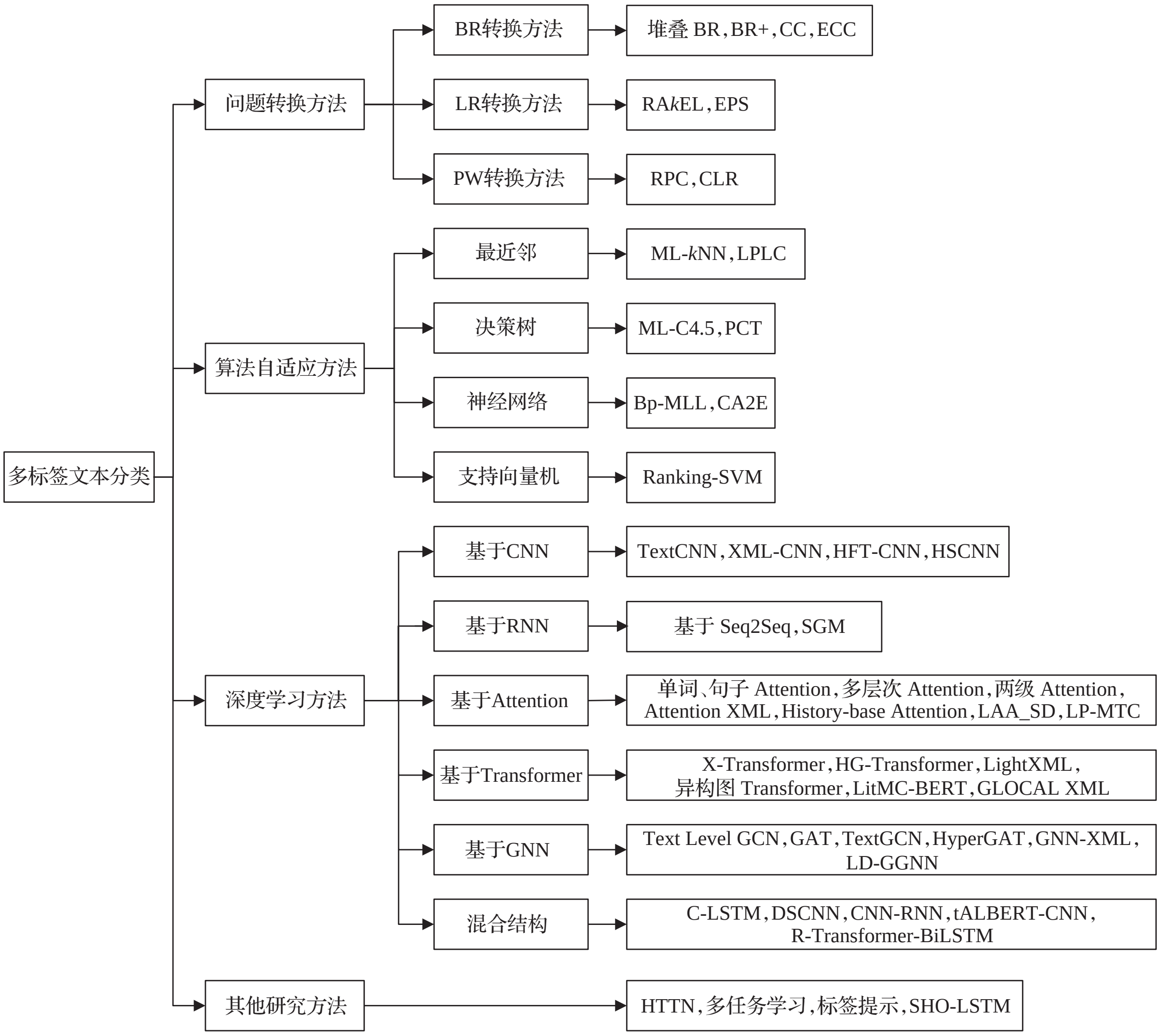

图3　多标签文本分类方法

Fig.3　Multi-label text classification method

计算机工程与应用 www.ceaj.org

（1）BR模型

2004年，Boutell等人[14]首先提出了基本的BR模型。它将多标签问题转换为几个二进制分类子问题，所有子问题共享相同的特征空间，但是标签空间不同。尽管BR算法简单，直观且应用广泛，但由于它忽略了标签之间的关系，因此预测性能较差。

（2）基于堆叠的BR模型

Godbole等人[15]将集成学习中的基于堆叠的学习策略引入到BR算法中，考虑了标签相关性。在训练过程中，基于堆叠的BR模型建立了两层BR模型：第一层是基础层，与传统BR学习过程相同，并为每个标签分配相应的二进制分类模型。在元层次的第二层中，将基础层次中所有二元分类模型的预测标签添加到原始特征空间中，在这些扩展的特征上再次学习每个标签，得到相应的二元分类模型。这种基于堆叠的BR算法假定任何标签都与所有标签相关，然而，这在大多数情况下是无效的。相关研究有算法BR+[16]和基于剪枝与堆叠的算法BR[17]。

（3）分类器链模型

Read等人[18]首次提出了分类器链（CC）模型。与BR算法不同，链中的标签将所有先前标签的二进制分类器输出添加到原始特征空间中，作为新的特征进行训练。CC模型有两个明显的缺点：一是分类器的效果受标签序列的影响很大，不同的序列带来明显不同的分类效果；另一个是当前标签可能与序列的前一部分中的标签无关，因此它可能通过使用所有先前标签的输出来引入噪声。在同一文献中，Read提出了CC的集成框架（ECC）。在ECC中，采用多个随机标签序列的CC模型的均值预测结果，可以在一定程度上解决随机标签序列对分类的影响，然而，这种计算费用成倍增加。Cheng等人提出了概率分类器链（PCC）模型，并指出可以从标签的条件联合分布中获得基于汉明或秩等损失函数的分类器链。除了改善基于概率推导的CC算法的效果之外，还有一些其他方法可以找到最佳或更好的标签序列。例如，GA-PratCC通过使用遗传算法搜索最佳标签序列；OOCC从k近邻中找到每个样本的最佳标签序列。

2.1.2 LR转换方法

LP变换方法[19]是将训练集中所有标签的组合视为一个类，然后将多标签问题转化为单标签多类问题。从学习中获得分类器后，看不见的实例是输入，类是输出，该类对应于一个标签集，该标签集涵盖了实例所属的所有标签。

LP变换方法只能预测出现在训练集中的标签集。此外，当有很多标签时，可能会有很多标签集。因此，许多集合可能具有一些相同的实例，从而导致类不平衡。这些问题不仅增加了学习的时间成本，而且降低了模型效果。为了解决这些问题，提出了两个著名的算法RAkEL[19]和EPS[20]。

随机k个标签集（RAkEL）算法通过LP变换方法训练每个标签集。RAkEL可以获得比BR和LP更准确的性能。此外，RAkEL还减少了必须学习的模型数量。在预测过程中，使用LP方法获得的所有学习者来预测看不见的实例，并对所有预测结果进行平均。因此，RAkEL是一种集成学习方法。

EPS算法计算与标签集(R_i)相关的实例数$(Count(R_i))$。如果$Count(R_i)$高于指定的阈值，则将与R_i相关的所有实例添加到训练集中。如果计数(R_i)低于阈值，则继续对与R_i的子集相关的实例进行计数。如果子集的频率高于指定阈值，则将与该子集相对应的样本添加到与R_i相对应的训练集中。这样，LP方法就在R_i的相应训练集上进行了训练。同时，EPS还通过集成学习减少了过拟合问题。

2.1.3 PW转换方法

2008年，Hüllermeier等人[21]提出了成对比较排序（RPC）算法，并将其应用于多标签分类。该算法将包含q个标签的多标签问题转换为$q(q-1)/2$个二进制分类子问题，每个子问题对应一对标签。标签对(y_i, y_j)的子问题包含原始问题中与标签y_i或y_j相关的所有实例，但同时与这两个标签相关的文本被排除在外。这样，与标签y_i相关的实例是正例，而其余的则被视为负实例。因此，可以通过传统的单标签算法解决此子问题。

显然，RPC算法的规模受标签数量的影响很大。当标签数量很大时，RPC算法对于高复杂度是不切实际的。此外，RPC算法无法区分与测试一下实例相关的标签。换句话说，它缺乏阈值或划分点来区分标签的哪一部分属于实例。

Fürnkranz等人[22]提出了校准标签排名（CLR）算法来解决上述划分点问题。在CLR算法中，添加了校准标签y_0作为相关标签和不相关标签的边界。与RPC算法相比，每个标签y_j只需添加一个子问题(y_j, y_0)，其中数据涵盖与标签y_j相关的所有实例（被视为与y_0无关）和与标签y_j无关的实例（被视为与y_0相关）。

2.2 算法自适应方法

算法自适应是对现有的单标签算法进行拓展以轻松应用于多标签文本数据。在本节中，介绍了几种具有代表性和广泛使用的算法自适应方法。

2.2.1 最近邻

ML-kNN[23]是第一个使用最近邻的多标签算法。其基本思想是计算k个最近邻中标签的出现，然后计算每个标签在不同出现时间下的概率，根据最大后验原理给出预测结果。为了估计相应的概率，ML-kNN必须实现大量的计算和距离比较，时间复杂度相对较高。如果训练集中存在噪声，ML-kNN算法的效果容易受到影响，并且ML-kNN不考虑标签相关性。为了解决这个问题，

计算机工程与应用 www.ceaj.org

LPLC[24]利用了最近邻范围内的一对标签中的相关性。LPLC与ML-*k*NN的概率估计差异很小，关键差异在于LPLC致力于为预测的标签找到相关标签集。LPLC假设强相关性仅存在于与训练实例相关的标签之间，对于具有 n 个实例和 q 个标签的训练集，需要定义一个 $n \times q$ 矩阵 M 来记录与每个实例相关的相关标签。然后，基于 M 计算每个标签的概率。

2.2.2　决策树

Clare等人[25]提出了一种基于决策树的多标签算法ML-C4.5。它从上到下构建决策树，树根包括所有训练样本。对于非叶节点中的实例，逐一调查每个特征，以找到合适的划分点。此划分点用于划分此节点的实例，从而获得最大的信息增益。

基于预测聚类树（PCT）[26]实现了分层多标签分类学习。与其他决策树类似，PCT也根据最大程度地减少簇内方差的原则，从上到下将当前簇划分为较小的簇。对于当前群集中的实例，方差度 S 定义为标签向量 (c_i) 与平均标签向量 $(\bar{c})$ 之间的距离的平方和。

2.2.3　神经网络

Bp-MLL[27]是第一个将传统神经网络转换为多标签分类的算法。它构造了一个简单的三层网络，输入层有 d 个输入单元，每个单元对应于训练集的一个特征；隐藏层包含 M 个单元；输出层具有 q 个单元，每个单元对应一个标签。在Bp-MLL中，全局损失函数参与区分实例的相关标签和不相关标签，并指导学习系统输出相对较大的相关标签值以及相对较小的不相关标签值。与传统的直接比较输出层各标签预测值和实际值的损失评估方法相比，Bp-MLL考虑了不同标签之间的关系，取得了更好的效果。CA2E是一种基于深度神经网络（DNN）提出的多标签分类算法。CA2E算法的目标函数可以分为两部分。第一部分是利用DNN模型求解目标函数，得到嵌入特征和标签空间。第二部分旨在使整个模型的输出恢复标签空间，其中使用类似于Bp-MLL的方法来解决该问题。

2.2.4　支持向量机

Elisseeff提出Ranking-SVM算法[28]。首先，Ranking-SVM算法将高效率单标签方法支持向量机（SVM）转化为直接用于多标签分类的方法。Ranking-SVM首先为每个标签定义线性分类器 $\{h_j(X) = <\boldsymbol{\omega}_j, X> + b_j = \boldsymbol{\omega}_j^{\mathrm{T}} + b_j | 1 \leqslant j \leqslant q\}$，然后最大化所有相关和无关标签对之间的距离。

2.3　深度学习方法

与传统的机器学习方法不同，深度学习方法更为复杂，但极其促进了多标签文本分类的发展，本节将深度学习模型根据其结构主要划分为基于卷积神经网络（convolutional neural network，CNN）、基于循环神经网络（recurrent neural network，RNN）、基于注意力机制（attention）、基于Transformer、基于图神经网络（graph neural network，GNN）和混合结构。

2.3.1　基于CNN

CNN包括卷积层、池化层和全连接层。典型CNN结构如图4所示。

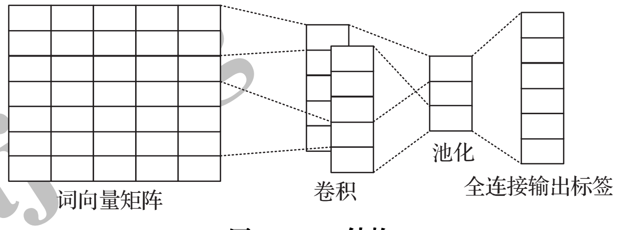

图4　CNN结构

Fig.4　CNN structure

CNN在检测局部和位置不变模式很重要的情况下性能良好。2014年，Kim等人[29]提出了TextCNN模型，在预先训练的词向量上训练CNN，用于句子级分类任务，用一个具有少量超参数调优和静态向量的简单CNN测试，通过微调学习特定于任务的向量。但因为CNN中需要利用固定窗口的问题，因此不可以对长文本信息进行建模。Liu等人[30]改进了TextCNN的结构，提出XML-CNN。该模型与TextCNN不同之处是通过将文档通过各种卷积过滤器传递来学习大量的特征表示，采用动态最大池化，从文档的不同区域捕获更多细粒度的特征；利用输出上的二进制交叉熵损失，在池化和输出层之间插入了一个额外的隐藏瓶颈层，以学习紧凑的文档表示形式。Shimura等人[31]提出了一种利用CNN的微调技术，一种分层卷积神经网络结构（HFT-CNN），有效利用上层数据为下层分类做出贡献。Yang等人[32]提出了一种双高光谱CNN（HSCNN）来处理不平衡数据，是一种混合-暹罗卷积神经网络（HSCNN），即基于单网和暹罗网络的多任务结构，对头部分类采用通用网络，对尾部分类采用少射技术。

尽管基于CNN的多标签文本分类不需要花费大量的计算成本，但是由于CNN的需要利用固定窗口的缺点以及池化操作会造成语义的丢失，所以当上下文文本过长时，基于CNN的模型不利于捕捉上下文之间的标签关系，因此不利于多标签文本分类。

2.3.2　基于RNN

RNN是一种用于从时间序列数据中捕获信息的网络。RNN结构如图5所示，其中，x_t 为 t 时刻输入；S_t 为 t 时刻隐层单元；y_t 为 t 时刻输出；O 为权重矩阵；U 为输入变换矩阵；V 为输出变换矩阵，与 U 在序列的不同时间点上共享，可以视为学习序列中固定的状态转移矩阵。

基于RNN的模型将文本视为一个单词序列，旨在为TC捕获单词依赖关系和文本结构。对于多标签文本

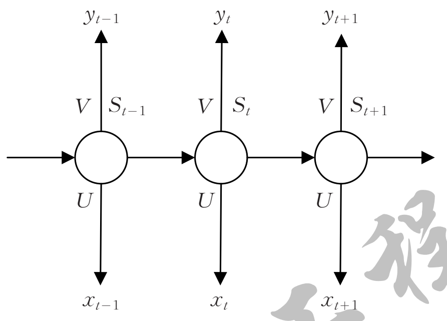

图5　RNN结构

Fig.5　RNN structure

分类,基于CNN的方法通常不能捕获多个标签之间的复杂关联,导致查全率低。为了解决这一问题,RNN被广泛应用于探索标签的相关性,用于多标签文本分类[33-34],它是一种逐条预测标签的递归神经网络。Nam等人[33]使用RNN代替的分类器链,这是一种序列到序列的预测算法,最近已成功应用于许多领域的序列预测任务。这种方法的关键优势在于,它允许仅专注于正标签的预测,其集合比可能标签的完整集合小得多。此外,所有分类器之间的参数共享可以更好地利用先前决策的信息。后续研究中,Yang等人[35]进一步将深度强化学习纳入Seq2Seq模型,以减少标签排列对性能的影响。Lin等人[36]提出了基于Seq2Seq模型的多级扩展卷积,扩张型卷积有效地降低了维数,支持接受域的指数扩展而不丢失局部信息。

但这些模型忽略标签之间的相关性或者不考虑文本内容关键信息,因此,不能得到较好的预测结果。Yang等人[37]把多标签文本分类问题当作序列生成,考虑标签间相关性,对解码部分进行改造,并且自动获取文本的关键信息,提升标签预测的有效性,这一改进在一定程度上改善了模型效果,但还有待提高。

2.3.3　基于Attention

注意力(Attention)机制由Bengio团队2014年提出并应用于自然语言处理。Yang等人[38]将单词级和句子级的注意力纳入模型,处理大规模文本分类。Hong等人[39]提出了注意力池化,增加了2D max池化操作,以维护更重要的语义信息,降低噪声的影响。此外,为了更好地突出不同情境下的重要文本信息,提出了多层次的注意力机制。Li等人[40]采用两级注意力来提高文本分类的性能,第一级注意力旨在同时捕获局部和长距离相关的特征,第二级注意力通过双向循环注意网络对生成的特征进行注意。You等人[41]提出Attention XML捕获文本与每个标签最相关的部分,Attention XML的出现超越所有传统机器学习方法,并证明原始文本与稀疏特征相比的优越性。与在XML-CNN中使用简单全连接层进行标签评分不同,Attention XML采用了可以处理数百万个标签的概率标签树(PLT),通过其上层模型初始化当前层模型的权重,可以帮助模型快速收敛。但是,这仍然使Attention XML在预测和从大型整体模型尺寸中获取数据方面的速度非常慢。Du等人[42]首先使用卷积运算来捕捉注意力信号,每个信号代表一个词在其上下文中的局部信息;然后这些注意信号再被进行预测。Xiao等人[43]第一个试图在Seq2Seq模型中提出了基于历史的注意力机制,以增强多标签文本分类中标签的预测能力。基于历史的注意机制考虑了历史上下文信息以避免陷入标签陷阱,同时考虑了历史标签信息以缓解错误传播问题。Liu等人[44]提出了LAA_SD方法,从冗余内容中选择歧视性特征,考虑了语义标签,并基于注意力机制建立了标签与文本之间的关系,该方法将增强的文本特征表示与标签语义依赖相结合,以执行文本多标签学习。Song等人[45]提出了一种标签提示多标签文本分类模型(LP-MTC),设计了一套多标签文本分类的模板,将标签集成到预先训练好的语言模型的输入中,并通过蒙面语言模型(MLM)联合优化。通过这种方式,在自我注意力机制下捕获标签之间的相关性以及标签与文本之间的语义信息,从而有效地提高了模型性能。

2.3.4　基于Transformer

RNN遇到的计算瓶颈之一是文本的顺序处理。尽管CNN的顺序性不如RNN,但捕获句子中单词之间关系的计算成本也会随着句子长度的增加而增加,这与RNN类似,Transformer克服了这些问题,其结构只保留了Attention,如图6所示,不需要与RNN或CNN相结合。应用自我注意力来并行计算句子或文档中每个单词的"注意力得分",以模拟每个单词对另一个单词的影响。由于这一特性,Transformer允许比CNN和RNN更多地并行化,这使得在GPU上对大量数据高效地训练非常大的模型成为可能。

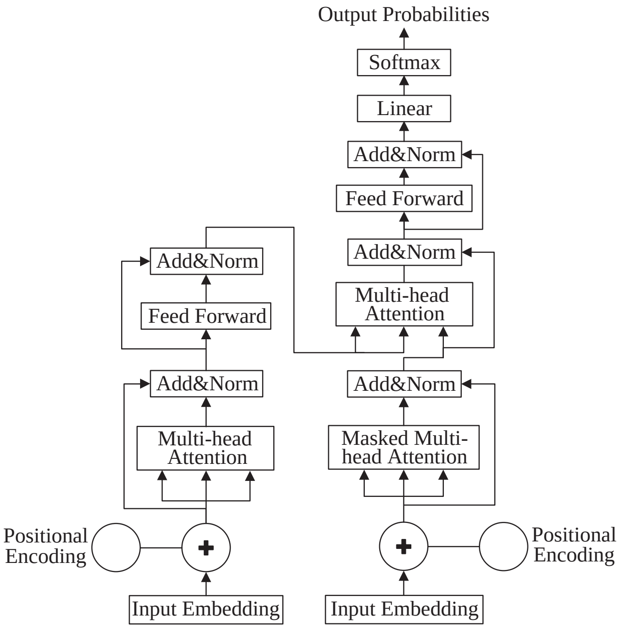

图6　Transformer结构

Fig.6　Transformer structure

计算机工程与应用 www.ceaj.org

Chang等人[46]提出了X-Transformer模型,它仅使用深度学习模型来匹配给定原始文本的标签簇,并通过具有深度学习模型的稀疏特征和文本表示的高维线性分类对这些标签进行排序。但由于Transformer模型的计算复杂度高,因此仅对Transformer模型进行微调作为标签聚类匹配器,无法充分利用Transformer模型的功能。尽管X-Transformer可以以较高的计算复杂度和模型大小为代价,达到比AttentionXML更高的精度,但因为通过使用更多的集成模型,AttentionXML可以在相同的X-Transformer计算复杂度下达到更好的精度。Gong等人[47]提出了HG-transformer,该模型将输入文本建模为图结构;然后在词、句、图三个层次采用多层转换结构,充分捕捉文本特征;最后利用标签之间的层次关系生成t个标签表示。Jiang等人[48]提出LightXML,采用端到端训练和动态负标签采样。在LightXML中,使用生成式合作网络对标签进行调用和排序,其中标签调用部分生成负标签和正标签,而标签排序部分将正标签与这些标签区分开来。通过这些网络,在标签排名部分训练期间,通过馈送相同的文本表示来动态采样负标签,提升模型的效果。Ye等人[49]提出了一种新颖的基于神经网络的多标签文档分类方法,其中使用异构图Transformer构造和学习两个异构图。一种是元数据异构图,它对各种类型的元数据及其拓扑关系进行建模;另一个是标签异构图,它是根据标签的层次结构及其统计依赖性构建的。Chen等人[50]提出LitMC-BERT模型使用共享Transformer主干,同时还捕获特定于标签的特征和标签对之间的相关性,来进行多标签分类。Zhang等人[51]认为全局特征向量可能不足以表示文档中语义的不同粒度级别,因此结合了Transformer模型产生的局部和全局特征,以提高分类器的预测能力。

2.3.5 基于GNN

虽然自然语言文本表现出连续的顺序,但是它们也包含内部的图结构,例如语法和语义分析树,其定义句子中的词之间的语法和语义关系。为NLP开发得最早的基于图的模型之一是TextRank。作者提出将自然语言文本表示为图形(V,E),其中V表示一组节点,E表示节点之间的一组边。根据具体的应用,节点可以表示各种类型的文本单位,例如单词、搭配、整句话等。同样,边缘可以表示任何节点之间的不同类型的关系,例如词汇或语义关系、上下文重叠关系等。

在各种类型的GNN中,图卷积网络(GCN)[52]及其变体,是最流行的一种,因为它们有效且方便地与其他神经网络组合,并且在许多应用中已经实现了最先进的结果。GCN将卷积操作从网格数据推广到图数据。主要思想是通过聚合它自己的特征和相邻的特征来生成节点的表示,GCN堆叠多个图卷积层以提取高级的节点表示。Liu等人[53]提出的Text Level GCN模型为每个输入的文本建构独立,但具有全局参数共享的图而不是为整个训练、测试语料库建立一个巨大的单图,并通过滑动窗口来构建图形,当中可以设定n元语法的数量,用于提取更多的局部特征,并减少大量的计算资源,这也使图神经网络能够从已有的资料中归纳出模式,并应用于新的任务。Velikovi等人[54]提出的图注意力网络(graph attention network,GAT)是GCN的一个变体,它操作图形结构数据,利用隐藏的自我注意力层来解决了先前基于图卷积缺点。通过层层叠加,节点能够参与到它们邻近节点的特性,支持(隐式地)指定不同的权重一个邻域中的不同节点,不需要任何昂贵的矩阵运算(如反转)或依赖于预先了解图的结构。Yao等人[3]提出了TextGCN模型,使用GCN为整个资料集建立一个基于文本和词的异构图,可以用来得到全局图的共现信息使GCN能够对文本进行半监督分类。Pal等人[55]提出了一种基于图注意力网络的模型来捕捉标签之间的注意依赖结构。图注意力网络使用特征矩阵和相关矩阵来捕获和探索标签之间的关键依赖关系,并为任务生成分类器。所生成的分类器被应用于从文本特征提取网络(BiLSTM)获得的句子特征向量以实现端到端训练。Ding等人[56]提出了一个原则性模型——超图注意网络(HyperGAT),它可以在文本表示学习中以更少的计算消耗获得更强的表达能力。Zong等人[57]提出了GNN-XML,这是一个可扩展图神经网络框架。通过挖掘它们的共现模式来利用标签相关性,并基于相关矩阵构建标签图;然后,通过使用低通图滤波器进行图卷积来联合建模标签依赖关系和标签特征,从而进行属性图聚类,诱导语义标签聚类。Zheng等人[58]提出了一种标签划分门控图神经网络(LD-GGNN),可以更好地区分同级标签,实现文本与标签之间的自适应交互,优化了门控图神经网络(GGNN),以准确捕获标签层次结构的结构特征,并深入探索标签依赖性,利用GGNN更强的非线性特性来解决过平滑问题。

2.3.6 混合模型

许多混合模型已经被开发出来应用于多标签文本分类。

Zhou等人[59]提出了一种卷积LSTM(C-LSTM)网络。C-LSTM利用CNN来提取较高级短语(n元语法)表示的序列,该序列被馈送到LSTM网络以获得句子表示。类似地,Zhang等人[60]提出了用于文档建模的依赖性敏感CNN(DSCNN)。DSCNN是一个分层模型,其中LSTM学习句子向量,这些句子向量被馈送到卷积和最大池层以生成文档表示。Chen等人[34]提出了一种CNN-RNN模型,以准确获得全局和局部文本语义信息,建模高阶标签相关性。Transformer的提出对自然语言处理领域产生了巨大的影响,但Transformer模型所需的模型参数往往较多,网络结构复杂,在实际应用过程中有

一定的局限性。Liu等人[61]提出了一种tALBERT-CNN的多标签文本分类方法,使用LDA主题模型和ALBERT模型获取每个词(文档)的主题向量和语义上下文向量,采用一定的融合机制获得文档的深度主题和语义表示,通过TextCNN模型提取文本的多标签特征,训练多标签分类器,减少了模型参数。Yan等人[62]本文提出了一种基于标签嵌入和注意力机制的R-Transformer_BiLSTM模型用于多标签文本分类。首次将实体识别模型引入文本分类利用R-Transformer模型结合部分语音嵌入来获取文本序列的全局和局部信息;同时使用BiLSTM+CRF获取文本的实体信息,使用自我注意力机制获取实体信息的关键词;然后,使用双向注意力和标签嵌入进一步生成文本表示和标签表示;最后,分类器根据标签表示和文本表示进行文本分类。

2.3.7　其他研究方法

Xiao等人[63]提出了一种头对尾网络(HTTN),将元知识从数据丰富的头部标签转移到数据贫乏的尾部标签。Zhang等人[64]引入了一种具有多任务学习的新颖方法,以增强标签相关性反馈。首先利用联合嵌入(JE)机制同时获得文本和标签表示,在MLTC任务中,采用了文档标签交叉注意力机制(CA)来生成更具歧视性的文档表示。此外,提出了两个辅助标签共现预测任务来增强标签相关性学习:(1)成对标签共现预测(PLCP)和(2)条件标签共现预测(CLCP)。Khataei等人[65]提出了一种基于LSTM网络和SHO算法的MLTC的斑点鬣狗优化器-长短期记忆(SHO-LSTM)模型。在LSTM网络中,将单词嵌入到向量空间中,采用SHO算法优化LSTM网络的初始权值。调整LSTM中的权重矩阵是一个重大挑战,如果神经元的权重准确,那么输出的精度会更高。表1列举了部分深度学习模型或方法。

3　多标签文本分类数据集

总结了13个多标签文本分类领域的主流数据集,涵盖中英文、长文本、短文本、极端多标签和普通多标签。并按照样本的平均标签数进行排列并在表2中显示了相关数据。

(1)IMDB:包含117 196个电影介绍(英文),共有27个电影类别,每个电影介绍都有一种或多种可能的类型。数据集根据电影是否属于特定类型,为每个电影提供多标签二进制掩码。

表1　深度学习模型及方法

Table 1　Deep learning models and methods

类别	模型/方法	文献	简要介绍	年份
基于CNN	TextCNN	[29]	利用基本CNN结构来进行分类,该模型将词向量组成的句子矩阵作为CNN的输入,利用CNN卷积器来提取特征	2014
	XML-CNN	[30]	将文档通过各种卷积过滤器传递来学习大量的特征表示。采用动态最大池化,从文档的不同区域捕获更多细粒度的特征。利用输出上的二进制交叉熵损失。在池化和输出层之间插入了一个额外的隐藏瓶颈层,以学习紧凑的文档表示形式	2017
	HFT-CNN	[31]	一种利用CNN的微调技术,一种分层卷积神经网络结构(HFT-CNN),有效利用上层数据为下层分类做出贡献	2018
	HSCNN	[32]	一种混合-暹罗卷积神经网络,即基于单网和暹罗网络的多任务,对头部分类采用通用网络,对尾部分类采用少射技术	2020
基于RNN	深度强化学习	[35]	将深度强化学习纳入Seq2Seq模型,以减少标签排列对性能的影响	2018
	多级扩展卷积	[36]	基于Seq2Seq模型的多级扩展卷积,扩张型卷积有效地降低了维数,支持接受域的指数扩展而不丢失局部信息	2018
	SGM	[37]	把多标签文本分类问题当作序列生成,考虑标签间相关性,对解码部分进行改造,并且自动获取文本的关键信息,提升标签预测的有效性	2018
基于Attention	单词、句子Attention	[38]	将单词级和句子级的注意力纳入模型,处理大规模文本分类	2016
	多层次Attention	[39]	增加了2Dmax池化操作,以维护更重要的语义信息,降低噪声的影响。此外,为了更好地突出不同情境下的重要文本信息,提出了多层次的注意力机制	2018
	两级Attention	[40]	第一级注意力旨在同时捕获局部和长距离相关的特征,第二级注意力通过双向循环注意网络对生成的特征进行注意	2018
	Attention XML	[41]	使用注意力机制来处理具有不同模型的原始文本,通过其上层模型初始化当前层模型的权重,可以帮助模型快速收敛	2019
	History-base Attention	[43]	考虑了历史上下文信息以避免陷入标签陷阱,同时考虑了历史标签信息以缓解错误传播问题	2021
	LAA_SD	[44]	从冗余内容中选择歧视性特征,考虑语义标签,基于注意力机制建立了标签与文本之间的关系	2022
	LP-MTC	[45]	将标签集成到预先训练好的语言模型的输入中,并通过蒙面语言模型(MLM)联合优化。通过这种方式,可以在自我注意的帮助下捕获标签之间的相关性以及标签与文本之间的语义信息	2022

计算机工程与应用 www.ceaj.org

计算机工程与应用 www.ceaj.org

表1 （续）

类别	模型/方法	文献	简要介绍	年份
基于Transformer	X-Transformer	[46]	模型包含了三部分，包括语义标签序列组件（SLI）、深度神经匹配组件和整体排名组件	2020
	HG-Transfomer	[47]	模型将文本建模为一个结构，在单词、句子引入了具有多头注意机制的多层Transformer结构来捕获特征，利用标签的层次关系来生成标签的表示形式	2020
	LightXML	[48]	用生成式合作网络对标签进行调用和排序，其中标签调用部分生成负标签和正标签，而标签排序部分将正标签与这些标签区分开来。通过这些网络，在标签排名部分训练期间，通过馈送相同的文本表示来动态采样负标签	2021
	异构图	[49]	使用异构图Transformer构造和学习两个异构图。一种是元数据异构图，它对各种类型的元数据及其拓扑关系进行建模。另一个是标签异构图，它是根据标签的层次结构及其统计依赖性构建的	2021
	LitMC-BERT	[50]	使用共享Transformer主干，同时还捕获特定于标签的特征和标签对之间的相关性，来进行多标签分类	2022
	GLOCAL XML	[51]	结合了Transformer模型产生的局部和全局特征，以提高分类器的预测能力	2022
基于GNN	Text Level GCN	[53]	为每个输入的文本建构独立，但具有全局参数共享的图而不是为整个训练、测试语料库建立一个巨大的单图，并通过滑动窗口来构建图形，提取更多的局部特征，并减少大量的计算资源	2016
	GAT	[54]	操作图形结构数据，利用隐藏的自我注意力层来解决了先前基于图卷积或它们的方法的缺点近似。通过层层叠加，节点能够参与到它们的邻居的特性，支持指定不同的权重一个邻域中的不同节点，不需要任何昂贵的矩阵运算运算（如反转）或依赖于预先了解图的结构	2017
	TextGCN	[55]	使用GCN为整个资料集建立一个基于文本和词的异构图，可以用来得到全局图的共现信息使GCN能够对文本进行半监督分类	2020
	HyperGAT	[56]	可以在文本表示学习中以更少的计算消耗获得更强的表达能力	2020
	GNN-XML	[57]	通过挖掘它们的共现模式来利用标签相关性，并基于相关矩阵构建标签图。然后，通过使用低通图滤波器进行图卷积来联合建模标签依赖关系和标签特征，从而进行属性图聚类，从而诱导语义标签聚类	2020
	LD-GGNN	[58]	更好地区分同级标签，实现文本与标签之间的自适应交互，优化了门控图神经网络（GGNN），以准确捕获标签层次结构的结构特征，并深入探索标签依赖性。利用GGNN更强的非线性特性来解决过平滑问题	2022
混合模型	C-LSTM	[59]	利用CNN来提取较高级短语（n元语法）表示的序列，该序列被馈送到LSTM网络以获得句子表示	2015
	DSCNN	[60]	一个分层模型，其中LSTM学习句子向量，这些句子向量被馈送到卷积和最大池层以生成文档表示	2016
	CNN-RNN	[34]	该模型将CNN和RNN进行融合，准确获得全局和局部文本语义信息，建模高阶标签相关性	2017
	tALBERT-CNN	[61]	使用LDA主题模型和ALBERT模型获取每个词（文档）的主题向量和语义上下文向量，采用一定的融合机制获得文档的深度主题和语义表示，通过TextCNN模型提取文本的多标签特征，训练多标签分类器	2021
	R-Transformer_BiLSTM	[62]	首次将实体识别模型引入文本分类，利用R-Transformer模型结合部分语音嵌入来获取文本序列的全局和局部信息。同时使用BiLSTM+CRF获取文本的实体信息，使用自我注意力机制获取实体信息的关键词，然后使用双向注意和标签嵌入进一步生成文本表示和标签表示。最后，分类器根据标签表示和文本表示进行文本分类	2022
其他研究方法	HTTN	[63]	一种头对尾网络（HTTN），将元知识从数据丰富的头部标签转移到数据贫乏的尾部标签	2021
	多任务学习	[64]	利用联合嵌入（JE）机制同时获得文本和标签表示。采用了文档标签交叉注意（CA）机制来生成更具歧视性的文档表示。提出了两个辅助标签共现预测任务来增强标签相关性学习：（1）成对标签共现预测（PLCP）和（2）条件标签共现预测（CLCP）	2021
	SHO-LSTM	[65]	使用Skip-Gram方法将单词嵌入到向量空间中。采用SHO算法优化LSTM网络的初始权值，调整LSTM中的权重矩阵	2022

（2）Ren-CECps1.0：该数据集是中文情感语料库，包含37 678个中文博客的句子和11种情感标签。

（3）Reuters-21578：数据集包含22个文档，总共10 788篇来自路透社的新闻文章，总共90个标签。

（4）AAPD：该数据集收集了55 840篇论文的摘要和相应学科类别，一篇学术论文属于一个或者多个学科。

（5）RCV1：共有804 414篇新闻报道，涉及103个类别。每个报告可能包含一个或多个类别。平均而言，每个新闻报道包含3.2个类别标签。

（6）RCV1-V2：该数据集共有804 414篇新闻，每篇新闻故事分配有多个主题，共有103个主题。

（7）ToutiaoNews：该数据集为今日头条统计新闻的中文数据集。

（8）Wiki-500K：数据也包含自维基百科，但与Wiki10-

表2　多标签文本分类数据集

Table 2　Multi-label text classification dataset

数据集	样本总数	标签数	样本平均单词数	样本平均标签数
IMDB	117 196	27	98.40	2.20
Ren-CECps1.0	37 678	11	24.71	2.40
Reuters-21578	10 788	90	—	—
AAPD	55 840	54	163.42	2.40
RCV1	804 414	103	268.90	3.20
RCV1-V2	804 414	103	123.94	3.20
ToutiaoNews	28 938	1 070	—	—
Wiki-500K	2 549 302	501 008	808.66	4.75
AmazonCat-13K	1 493 021	13 330	448.57	5.00
EUR-Lex	19 314	3 956	1 239.49	5.30
Amazon-670K	643 474	670 091	244.27	5.50
Wiki10-31K	20 762	30 938	2 484.30	18.64
Amazon-3M	2 460 406	2 812 281	104.13	36.00

31K数据集相比，样本数量更大，标签数量更大。

（9）AmazonCat-13K：该数据集来自亚马逊，包含用户评论和产品信息等数据。

（10）EUR-Lex：数据集组织来自各种欧盟法律、条约等的文件，并包含15 449个训练文档和3 865个测试文档，整个数据集总共有3 956个标签。

（11）Amazon-670K：该数据集是亚马逊商品的评论，有643 474条样本数据。

（12）Wiki10-31K：数据集包含维基百科上的20 762篇文章，但标签数量达到了30 938个。

（13）Amazon-3M：该数据集是亚马逊商品的产品信息、链接和评论，标签数量达到2 812 281个。

4　多标签文本分类评估指标与模型分析

4.1　评估指标

多标签文本分类（MLTC）模型的评估不同于单标签分类模型的评估。因此，根据文献[66]，已经提出了几种多标签文本分类评估指标，它们分为两种主要方法：基于实例的指标和基于标签的指标。第一种方法是测试每个实例，然后对所有测试实例进行平均计算。第二种方法是对每个标签计算，然后在所有标签上取平均值。

4.1.1　基于实例的指标

下面介绍了用于评估多标签文本分类模型的最常见的基于实例的指标。假设：m 指数据集中的实例总数，i 表示数据集中的实例（其中 $1\leqslant i\leqslant m$），n 为标签总数，Z_i 和 Y_i 分别指预测的和实际的标签。

（1）汉明损失（Hamming loss）：计算在实例标签对中发现的平均错误数，并在所有实例上平均。此度量的表达式如公式（1）所示：

$$Hamming\ loss=\frac{1}{m}\sum_{i=1}^{m}\frac{1}{n}\left|Z_i\Delta Y_i\right| \tag{1}$$

其中 Δ 定义了预测标签和实际标签之间的对称差，因子 $1/n$ 用于获得[0，1]中的归一化值。

（2）多标签精度（ML-accuracy）：计算正确预测的标签与标签总数的比率，计算如公式（2）所示：

$$ML\text{-}accuracy=\frac{1}{m}\sum_{i=1}^{m}\frac{\left|Z_i\cap Y_i\right|}{\left|Z_i\cup Y_i\right|} \tag{2}$$

（3）子集精度（Subset-accuracy）：称为精确匹配比或分类精度。这是一个非常严格的指标，用于测量预测标签的比率，该比率与它们相应的实际标签集完全匹配，计算如公式（3）所示：

$$Subset\ accuracy=\frac{1}{m}\sum_{i=1}^{m}I(Z_i=Y_i) \tag{3}$$

（4）精度（Precision）：该指标提供了正确分类的标签与预测标签的比率，计算如公式（4）所示：

$$Precision=\frac{1}{m}\sum_{i=1}^{m}\frac{\left|Z_i\cap Y_i\right|}{\left|Z_i\right|} \tag{4}$$

（5）召回率（Recall）：计算实际标签的正确预测标签的比率，计算如公式（5）所示：

$$Recall=\frac{1}{m}\sum_{i=1}^{m}\frac{\left|Z_i\cap Y_i\right|}{\left|Y_i\right|} \tag{5}$$

（6）F-度量（F-measure）：精度和召回率的调和平均，计算如公式（6）所示：

$$F\text{-}measure=\frac{1}{m}\sum_{i=1}^{m}\frac{2\left|Z_i\cap Y_i\right|}{\left|Z_i\right|+\left|Y_i\right|} \tag{6}$$

除了汉明损失度量之外，本小节中描述的所有基于示例的度量都表明具有越高值的度量具有更好的性能，汉明损失的值越低表示性能越好。

4.1.2　基于标签的指标

基于两种计算平均值的方法为所有标签计算二进制评估指标（例如召回率、精度和 F -度量）：宏观或微观平均方法。这些指标被广泛用于测量召回率、精度和 F -度量的平均值。设 B 为一种用于计算这些指标的二进制评估度量，该度量基于真正类（t_p）、假正类（f_p）、真负类（t_n）和假负类（f_n）的数量进行计算。如公式（7）、（8）中说明了 $B(t_p,f_p,t_n,f_n)$ 的宏观平均和微观平均指标的表达式。

$$B_{\text{macro}}=\frac{1}{n}\sum_{i=1}^{n}B(t_{p_i},f_{p_i},t_{n_i},f_{n_i}) \tag{7}$$

$$B_{\text{micro}}=B\left(\sum_{i=1}^{n}t_{p_i},\sum_{i=1}^{n}f_{p_i},\sum_{i=1}^{n}t_{n_i},\sum_{i=1}^{n}f_{n_i}\right) \tag{8}$$

4.2　模型分析

表3为对相关多标签文本分类方法在部分数据集上的结果分析。

从模型结果可以看出，随着深度学习的发展，基于深度学习方法的模型在不同数据集上的F-measure值都有显著的提升。在APPD数据集上，F-measure值从BR模型的0.641 2提升到LP-MTC模型的0.745 8，提升的

计算机工程与应用 www.ceaj.org

计算机工程与应用 www.ceaj.org

表3　模型方法结果分析

Table 3　Analysis of model method results

类别	模型/方法	文献	数据集	Precision	Recall	F-measure	年份
传统机器学习方法	BR	[18]	AAPD	0.664 1	0.648 3	0.646 1	2004
			RCV1-V2	0.904 2	0.816 1	0.858 1	
	CC	[14]	AAPD	0.657 4	0.651 4	0.654 2	2011
			RCV1-V2	0.887 1	0.828 1	0.857 3	
基于CNN	TextCNN	[29]	IMDB	0.720 2	0.615 2	0.671 4	2014
			AAPD	0.631 2	0.550 6	0.573 2	
	XML-CNN	[30]	IMDB	0.728 7	0.647 5	0.690 5	2017
			AAPD	0.651 8	0.588 4	0.623 5	
基于RNN	SGM	[37]	RCV1-V2	0.887 0	0.850 1	0.869 1	2018
			AAPD	0.746 1	0.659 2	0.699 3	
	多级扩展卷积	[36]	RCV1-V2	0.891 0	0.873 3	0.882 1	2018
			Ren-CECps	0.593 1	0.585 1	0.590 2	
基于Attention	Attention XML	[41]	AAPD	0.757 1	0.685 7	0.715 5	2019
			RCV1-V2	0.898 7	0.866 5	0.881 7	
	LAA_SD	[44]	APPD	0.764 1	0.693 2	0.721 1	2022
基于Transformer	X-Transformer	[46]	EURLex-4K	0.751 2	0.684 0	0.717 9	2020
			Wiki10-31K	0.787 1	0.714 2	0.750 4	
			AmazonCat-13K	0.838 5	0.770 1	0.804 1	
	LightXML	[48]	EURLex-4K	0.758 9	0.691 1	0.725 1	2021
			Wiki10-31K	0.789 6	0.713 2	0.751 4	
			AmazonCat-13K	0.840 2	0.782 4	0.811 6	
	GLOCAL XML	[51]	EURLex-4K	0.789 0	0.718 5	0.753 8	2022
			Wiki10-31K	0.809 5	0.732 1	0.771 0	
			AmazonCat-13K	0.839 7	0.768 4	0.762 3	
基于GNN	GNN-XML	[57]	EURLex-4K	0.761 4	0..691 2	0.726 3	2020
			Wiki10-31K	0.811 3	0.748 5	0.780 1	
	LD-GGNN	[58]	RCV1-V2	0.892 4	0.886 4	0.889 4	2022
混合模型	CNN-RNN	[34]	AAPD	0.718 1	0.618 7	0.664 7	2017
			RCV1-V2	0.889 8	0.825 8	0.856 4	
	tALBERT-CNN	[61]	IMDB	0.843 1	0.758 5	0.802 3	2021
			AAPD	0.751 9	0.669 5	0.731 7	
	R-Transformer_BiLSTM	[62]	AAPD	0.762 1	0.689 3	0.718 1	2022
			RCV1-V2	0.910 1	0.890 4	0.893 2	
其他研究模型	SHO-LSTM	[65]	RCV1-V2	0.896 8	0.861 7	0.880 3	2022
			Reuter-21578	0.643 4	0.639 4	0.641 4	
			Bookmarks	0.422 4	0.425 1	0.423 3	

效果十分明显。在RCV1-V2数据集上，F-measure从BR模型的0.851 8提升到R-Transformer_BiLSTM模型的0.893 2。在EURLex-4K数据集上模型的效果从0.717 9提升到0.753 8。在其他的数据集上，随着深度学习的发展，模型都有显著的提升。R-Transformer_BiLSTM模型在RCV1-V2数据集上的表现完全优于在AAPD数据集的表现，说明此模型对大规模数据标签的文本分类依然具有良好的性能。

传统的机器学习方法，如BR和CC等，因为其不考虑标签的相关性，并且当前标签可能与序列的前一部分中的标签无关，从而在使用先前标签的输入来引入了噪声，导致分类器性能的降低，因此在部分数据集上的表现较差。

基于CNN的深度学习网络模型，如TextCNN和XML-CNN等，由于CNN固定窗口的缺点以及其池化操作导致语义的丢失，影响了它的分类器的性能。虽然XML-CNN模型对这一缺点有一定程度上的改进，但是由于根本上CNN结构简单，因此在不同数据集的表现一般。

基于RNN的深度学习网络模型，如SGM等，对模型进一步改善，提高了分类器的性能，但其在预测标签时，基于序列的模型因为后一个标签往往依赖于前一个标签，所以前一个错误标签的影响往往是叠加的，导致分类器性能下降。基于RNN的模型虽然考虑了标签的相关性，但是模型的效果提升不明显。

基于Attention的深度学习网络模型，如Attention

XML和LAA_SD等，应用动态最大池化来学习文本表示，运用多层次Attention来捕捉特征，Attention XML使用双向长短时记忆（BiLSTM）网络从原始文本输入中提取嵌入，在模型的性能上提升的效果明显，但是因为不充分考虑局部或者全局的标签相关性，在一定程度上影响了模型性能。

基于Transformer的深度学习网络模型，如X-Transformer等，克服了文本的顺序处理的问题以及减少了捕获句子中单词之间关系的计算成本，从而提升了模型的性能，但是在实际的应用场景中，Transformer模型所需的模型参数往往较多，网络结构复杂，所以还是一定程度上影响了模型性能。

基于图神经网络的深度学习网络模型，如GNN-XML和LD-GGNN等，挖掘文本内部的图结构，从网络数据推广到图数据，提取更多的局部特征，减少大量的计算资源，不断挖掘标签之间的相关性，提高了模型的性能，在不同的数据集上表现明显。

混合的深度学习网络模型，如tALBERT-CNN和R-Transformer_BiLSTM模型利用注意力机制考虑上下文信息，考虑标签之间的相关性，抽取出文本的关键信息，用于多标签文本分类，其分类器效果显著，但是分类效果依然有提升的空间。

近年来，其他研究模型在不同的数据集上的分类效果都有明显提高，但是在面对大规模标签数据集以及层次多标签数据集时的分类效果并不明显，未来研究需要进一步提升。

5　多标签文本分类研究方向

5.1　标签相关性

5.1.1　标签相关性的类型

在多标签问题中，标签不是独立的，而是存在一些相关性。标签相关性的使用有利于学习更有效和稳健的分类模型。在多标签文本分类问题中，某些标签的正样本很少，在这种情况下，使用标签相关性是极其重要的。充分利用标签相关性已成为目前多标签分类的主要研究方向之一，它是许多算法的重要组成部分[67-68]。现有的标签相关使用策略可以分为三种类型[69]：一阶、二阶和高阶。

（1）一阶算法

一阶算法如BR、ML-C4.5和ML-*k*NN[23]。BR为每个标签构建二进制分类方法，目的是学习相应的分类器$h_i:X\rightarrow\{0,1\}$。在学习过程中，输入是原始特征空间，而输出是标签y_i的值。因此，不同的标签具有相同的输入，但具有不同的输出。ML-C4.5通过决策树将训练数据集逐层分成几个小子集，树根覆盖所有训练数据。对于非叶节点，应用信息熵和基尼指数等指标进一步将非叶节点划分为子节点，使得子节点数据的“纯度”高于父节点。ML-*k*NN是一种惰性学习方法。在预测时，ML-*k*NN模型根据预测数据在训练数据的最近邻中各标记的分布情况，采用最大化后验概率的原则决定测试样例是否与某一标记相关。但这些一阶算法完全忽略标签相关性。

（2）二阶算法

二阶算法如Rank-SVM[28]、CLR[22]、MLPP[70]、CPNL[71]、PCT[72]和GBRAML[73]考虑了的标签相关性。Rank-SVM定义了在相关性-非相关性标签对中最大化距离的优化目标，并利用SVM技术解决了多标签分类问题。CLR算法通过成对比较方法将常见的学习扩展到多标签文本分类中，引入一个人工校准标签，在每个实例中，将相关标签从不相关标签中分离出来。MLPP通过每对标签对分类器进行训练，并通过投票结合各种分类器的预测结果来确定标签相关性的序列。CPNL利用了标签的正负相关性，扩展了BR算法。PCT根据排序损失的定义，提出了一种标签两两比较变换方法，将每个原始的多标签样本转化为特征向量相同、标签向量不同的多个样本。GBRAML是一种基于颗粒批处理模式的多标签排序活动模型。从自下而上的角度来看，依次构造了三个造粒算子，以形成三个颗粒结构。在低级造粒算子中，引入辅助标签以增强每个标签的信息性和代表性。表4列举了标签相关性研究的一阶算法和二阶算法。

（3）高阶算法

高阶算法如RA*k*EL[19]、CC[18]、BNCC[74]和MLMF[75]考虑几个或所有标签之间的相关性。作为一种集成学习方法，RA*k*EL算法通过考虑一个小的标签随机子对于任何标签，序列前部的标签都会作为新特征添加到原始特征中，以这种方式利用了多个标签之间的关系。BNCC利用贝叶斯网络对标签相关性进行建模，用条件熵描述标签之间的依赖关系，以节点为标签，以边的权重为依赖关系，并引入了一种启发式算法来优化BN结构，通过对优化后的BN节点进行拓扑排序，得到构建CC模型的标签顺序。MLMF设计一个有效的多标签分类器，自动学习高阶非对称标签相关性，降低特征空间的维数，处理完整标签和缺失标签的情况。从关系提取或使用角度来看，高阶算法可以分为全局关系算法、局部关系算法和全局-局部组合关系算法。

① 全局关系算法

全局关系算法认为标签相关性是全局的，换句话说，标签之间的相关性存在于所有训练数据中。全局关系算法如CC、MLLS[76]、ML-LPC[77]、CLSF[78]和A-GCN[79]。CC将所有标签放在一个随机序列中。先前标签的二进制分类器输出作为新特征添加到标签的原始特征空间中。MLLS是一个通用的框架来提取多标签分类中的共享结构，在这个框架一个公共子空间被多个标签共享，从而阐明了它们的内在关系。ML-LPC学习标签之

计算机工程与应用 www.ceaj.org

表4　一阶算法和二阶算法

Table 4　First-order algorithms and second-order algorithms

类别	文献	框架	简要介绍	年份
一阶算法	[18]	BR	为每个标签构建二进制分类方法,目的是学习相应的分类器 $h_i:X\rightarrow\{0,1\}$。在学习过程中,输入是原始特征空间,而输出是标签 y_i 的值。因此,不同的标签具有相同的输入,但具有不同的输出	2004
	[25]	ML-C4.5	通过决策树将训练数据集逐层分成几个小子集,树根覆盖所有训练数据。对于非叶节点,应用信息熵和基尼指数等指标进一步将非叶节点划分为子节点,使得子节点数据的"纯度"高于父节点	2007
	[23]	ML-*k*NN	根据预测数据在训练数据的最近邻中个标记的分布情况,采用最大化后验概率的原则决定测试样例是否与某一标记相关	2016
二阶算法	[28]	Rank-SVM	定义了在相关性-非相关性标签对中最大化距离的优化目标,并利用SVM技术解决了多标签分类问题	2003
	[22]	CLR	通过成对比较方法将常见的学习扩展到多标签场景中,引入一个人工校准标签,在每个示例中,将相关标签从不相关标签中分离出来	2008
	[70]	MLPP	通过每对标签对分类器进行训练,并通过投票结合各种分类器的预测结果来确定标签相关性的序列	2008
	[72]	PCT	根据排序损失的定义,提出了一种标签两两比较变换方法,将每个原始的多标签样本转化为特征向量相同、标签向量不同的多个样本	2017
	[71]	CPNL	利用了标签的正负相关性,扩展了BR算法	2018
	[73]	GBRAML	从自下而上的角度来看,依次构造了三个造粒算子,以形成三个颗粒结构。在低级造粒算子中,引入辅助标签以增强每个标签的信息性和代表性	2022

间的相关性同时训练多标签模型。采用低秩结构来捕获标签之间复杂的相关性,利用标签相关性得到不完整的标签矩阵。CLSF首先定义每个标签的基本要素和特征反映了内部特性和标签之间的联系。此外,还提供了计算单个标签的基本元素的过程,其次,通过考虑不同标签所确定的基本元素集合的重叠,描述了标签的相关性以及与标签集对应的相关性判断矩阵,因此,几个具有强关系的标签被分配到一个相关的标签组中,同时,可以计算局部和全局标签相关性。A-GCN使用标签图来学习带有单词嵌入的全局标签相关性。

② 局部关系算法

在局部关系算法中,标签相关性存在于训练数据的一部分中。在这种情况下,标签的依赖关系仅存在于某些数据中。如果从全局角度提取或使用此类标签相关性,则将对所有实例施加不必要甚至误导的约束,这将降低分类模型的性能。这些局部考虑标签相关性的算法LPLC[80]。LPLC在局部考虑标签相关性,为所有训练实例找到每个标签的正负标签相关性。然后,对于每个测试实例,基于其 k 近邻的局部正负标签相关性,使用最大后验概率进行预测。Ma等人[81]提出了一个具有局部特征选择和局部标签相关性的新框架,在该框架中,假设实例可以聚到不同的组中,且特征选择权重和标签相关性只能由同一组中的实例共享。该框架包括一个特定于组的特征选择过程和一个特定于标签组选择过程。前者通过提取实例组-相关性将实例投射到不同的组中,后一个过程通过提取组-标签相关性,基于相关组为每个实例选择标签集,并学习一个单标签分类器来预测这个子集的幂集中的每个元素来构造集合的每个元素。CC算法将标签随机放入一个序列中,为每个标签构建二进制分类器。

③ 全局-局部关系算法

全局-局部组合关系算法同时考虑全局和局部标签相关性,建立高效分类模型。例如,GLOCAL[82]通过流形正则化学习全局和局部标签相关性。GL*k*EL[83]通过近似联合互信息从标签空间中选择最相关的 k 标签集,以评估全局标签相关性。然后,它将训练数据聚类为不同的组,并评估每个组中的局部标签相关性。LFGLC[84]集成了全局和局部标签相关性,以提取每个标签的标签特定特征。Liu等人[85]在整个标签空间中计算一个全局标签相关矩阵,根据簇内标签的余弦相似度,为每个实例子集分配一个局部标签相关矩阵,基于标签相关性可以从原始类别空间转移到数值标签空间的假设,添加了全局和局部标签相关性正则化项,将重要性估计和模型训练整合到一个统一的框架中。表5列举了标签相关性的高阶算法。

5.1.2　标签相关性的研究

(1)基于标签相关性的特征压缩

为了消除冗余和不相关的特征,研究人员提出了许多方法来压缩多标签数据特征。这些方法中的许多方法通过使用标签相关性来选择特征或实现特征转换。在参考文献[86]中,提出过滤特征选择方法;最小冗余和最大相关性(mRMR);用互信息来衡量标签的重要性;通过多种权重策略估计特征和标签之间的关系。

(2)基于标签相关性的特征扩展

上述方法基于标签相关性对原始特征空间进行压缩。一些算法通过使用标签相关性来扩展特征,基于堆叠的BR算法[17],其中BR的第二层中二进制分类器从第一层中选择强相关的输出来扩展原始特征空间。在参考文献[87]中,提出了一种分类器链模型来处理多标签问题,主要创新在于它选择了一个有向无环图来对标签相关性进行建模,并通过条件熵来测量标签相关性,从而最大化了图中表示的所有标签之间的相关性之和;再

计算机工程与应用 www.ceaj.org

表5　高阶算法

Table 5　High-order algorithms

类别	文献	模型/方法	简要介绍	年份
全局关系算法	[18]	CC	将所有标签放在一个随机序列中。先前标签的二进制分类器输出作为新特征添加到标签的原始特征空间中，并根据此顺序学习相应的二进制分类器，标签相关性是在所有训练数据上构建的	2011
	[76]	MLLS	在这个框架一个公共子空间被多个标签共享，包括了几个众所周知的算法作为特殊情况，从而阐明了它们的内在关系	2008
	[77]	ML-LPC	学习标签之间的相关性同时训练多标签模型。采用低秩结构来捕获标签之间复杂的相关性，利用标签相关性得到不完整的标签矩阵	2014
	[78]	CLSF	定义每个标签的基本要素和特征反映了内部特性和标签之间的联系。还提供了计算单个标签的基本元素的过程。通过考虑不同标签所确定的基本元素集合的重叠，描述了标签的相关性以及与标签集对应的相关性判断矩阵	2020
	[79]	A-GCN	使用标签图来学习带有单词嵌入的全局标签相关性	2020
	[80]	LPLC	在局部考虑标签相关性，为所有训练实例找到文章标题每个标签的正负标签相关性。然后，对于每个测试实例，基于其 k 近邻的局部正负标签相关性，使用最大后验概率进行预测	2017
	[81]	局部特征局部标签	假设实例可以聚到不同的组中，且特征选择权重和标签相关性只能由同一组中的实例共享。该框架包括一个特定于组的特征选择过程和一个特定于标签的组选择过程。前者通过提取实例－组相关性将实例投射到不同的组中。后一个过程通过提取组-标签相关性，基于相关组为每个实例选择标签	2020
全局-局部组合关系算法	[82]	GLOCAL	通过流形正则化学习全局和局部标签相关性	2018
	[83]	GLkEL	通过近似联合互信息从标签空间中选择最相关的 k 标签集，以评估全局标签相关性。然后，它将训练数据聚类为不同的组，并评估每个组中的局部标签相关性	2018
	[84]	标签重要性	在整个标签空间中计算一个全局标签相关矩阵。其次，根据簇内标签的余弦相似度，为每个实例子集分配一个局部标签相关矩阵；基于标签相关性可以从原始类别空间转移到数值标签空间的假设，添加了全局和局部标签相关性正则化项。最后，将重要性估计和模型训练整合到一个统一的框架中	2022

根据这个有向无环图和预测扩展了原始特征空间，即将与原有标签相对应的二进制分类器的结果添加进去；最后，基于扩展特征训练二进制分类器。ML-LOC[88]也是一种基于标签相关性的特征扩展算法。

（3）基于标签相关性的标签嵌入

标签嵌入是一种重要的多标签分类算法，它可以联合提取所有标签的信息，从而获得更好的性能。Chen等人[89]提出了使用图卷积网络学习标签嵌入和标签之间的相关性，使用融合层将标签信息与文本的上下文语义信息结合起来。Wang等人[90]提出了一个新的基于跨视图的模型，具有多标签分类鉴别结构的鲁棒交叉视图嵌入（RCEDS）。该方法实现了一种鲁棒和鉴别嵌入，在RCEDS中，设计了一种新的超图融合技术，利用特征空间和标签空间之间的互补性，同时利用双边度量学习挖掘特征空间和标签空间的一致性。

5.2　特定标签特性

2014年，Zhang等人[91]提出了一种提升算法，首次提出了标签特定特征的概念。大多数现有的多标签方法通常使用相同的实例表达式来为不同的标签构建分类模型。换句话说，不同的标签在学习过程中使用相同的特征矩阵。尽管如此，提升算法认为标签应具有其唯一的表达式，因此，不同的标签应使用适当的特征表达。只有利用这些特征，才能进一步提高分类模型的效果，这些功能被称为“特定于标签的功能”。标签特定特征的概念与传统特征压缩的概念有明显的区别。在传统特征压缩的概念中，一般通过特征提取或特征选择向所有标签提供统一的特征表达。标签特定功能是指与特定标签相关的功能，而不是与所有标签相关的功能。

目前，构建标签特定特征的方法主要有两种：特征提取和特征选择。前者用LIFT表示[91]，后者用LLSF表示[92]。表6列举了特定标签特性的研究。

5.2.1　基于特定标签特性的特征提取

LIFT通过特征提取为每个标签提取特定于标签的特征。具体来说，与任何标签相关的样本被视为正样本，而其余样品被视为负样本。k 均值分别用于对正样本集和负样本集进行聚类，计算从样本到聚类中心的距离，这些距离形成了新的样本特征。接下来，在这些新的标签特定功能的空间上学习二进制分类器。对于不同的标签，正样本和负样本的分布是不同的，因此构建的标签特定特征彼此不同。在大量实验的基础上，证明其性能的非凡效果。此后，特定于标签的功能引起了学术界的广泛兴趣，并相继提出了一系列算法。

基于LIFT、LF-LPLCl[93]集成了标签特定特征和局部成对标签相关性，其中每个标签的特定特征通过将相关标签中的相关特征结合在一起来扩展，这丰富了标签的语义信息，并在一定程度上解决了类别不平衡问题。LETTER[94]从实例和特征级别中提取标签特定的特征。从实例层面，使用稀疏和原型约束来查找更具歧视性的实例中心；从特征层面，利用聚类从正负实例的原始特征中找到特征中心，最终的标签特定特征由从上述两个级别中提取的中心组成。Fan等人[95]提出了一种新的基于标签相关性和特征冗余度的LFFS方法。首先利用岭

计算机工程与应用 www.ceaj.org

表6　特定标签特性

Table 6　Features of lable-specific

类别	文献	模型/方法	简要介绍	年份
基于特定标签特性的特征提取	[91]	LIFT	通过特征提取为每个标签提取特定于标签的特征	2015
	[92]	LF-LPLCl	集成了标签特定特征和局部成对标签相关性，其中每个标签的特定特征通过将相关标签中的相关特征结合在一起来扩展。这丰富了标签的语义信息，并在一定程度上解决了类别不平衡问题	2018
	[94]	LETTER	从实例和特征级别中提取标签特定的特征。从实例级别上，使用稀疏和原型约束来查找更具歧视性的实例中心。从特征层面，利用聚类从正负实例的原始特征中找到特征中心。最终的标签特定特征由从上述两个级别中提取的中心组成	2020
	[95]	LFFS	利用岭回归建立特征选择矩阵和低维嵌入；然后，采用低维嵌入挖掘标签相关性，保持原始标签空间的全局和局部结构；最后，利用余弦相似度分析特征冗余度，生成低冗余度特征子集	2022
	[96]	LC领域集	通过计算标签之间的相似关系来探索LC，并将相关标签划分为多个标签子集。然后，提出了一种新的邻域关系，利用相关标签下实例的最近邻信息分布，解决了邻域粒度选择问题	2022
基于特定标签特性的特征选择	[92]	LLSF	目标函数假设强相关标签比弱相关标签具有更多的标签特定特征。通过线性回归实现了特征选择，它可以根据选择的特征学习二进制分类模型	2015
	[97]	NSLSF	将逻辑标签转换为数字标签，以传达更多的语义信息，并嵌入标签相关性	2018
	[98]	基于MFNRS和MRMR	针对标签缺失的多标签数据，构造样本关系系数、标签互补矩阵和标签特定特征矩阵，并在线性回归模型中实现对缺失标签的恢复；其次，建立了基于边缘的模糊邻域半径、模糊邻域相似关系和模糊邻域信息颗粒；多标签邻域粗糙集与模糊邻域粗糙集相结合，建立了多标签邻域粗糙集模型	2022
	[99]	RWFS	基于两种类型的变化比率，通过同时考虑两种类型的特征相关性评估比率来提供更可靠的特征排序	2022

回归建立特征选择矩阵和低维嵌入；然后，采用低维嵌入挖掘标签相关性，保持原始标签空间的全局和局部结构；最后，利用余弦相似度分析特征冗余度，生成低冗余度特征子集。Wu等人[96]引入了一个新的考虑LC的领域集模型。首先，通过计算标签之间的相似关系来探索LC，并将相关标签划分为多个标签子集；然后，提出了一种新的邻域关系，利用相关标签下实例的最近邻信息分布，解决了邻域粒度选择问题。

5.2.2　基于特定标签特性的特征选择

上述算法均采用特征变换提取标签特有的特征，但是，Huang等人[92]提出的LLSF算法通过特征选择技术来学习标签特定的特征。LLSF假设每个标签仅与一些原始特征相关，并且它在具有约束的线性回归中表达了这种稀疏性，非零回归参数表示相应的特征是特定于标签的，而其他特征则不是。

LLSF的目标函数假设强相关标签比弱相关标签具有更多的标签特定特征。由于LLSF通过线性回归实现了特征选择，它可以根据选择的特征学习二进制分类模型。NSLSF[97]认为稀疏性假设在某些应用中不成立，提出了一种基于特征选择的方法来选择标签特定的特征。它将逻辑标签转换为数字标签，以传达更多的语义信息，并嵌入标签相关性。MCUL还利用系数矩阵上的范数正则化来学习稀疏标签特定特征，从而处理缺失和完全未观察到的标签。Sun等人[98]提出了一种基于多标签模糊邻域粗糙集（MFNRS）和最大相关最小冗余（MRMR）的特征选择方法，可用于缺少标签的多标签数据。首先，针对标签缺失的多标签数据，构造样本关系系数、标签互补矩阵和标签特定特征矩阵，并在线性回归模型中实现对缺失标签的恢复；其次，建立了基于边缘的模糊邻域半径、模糊邻域相似关系和模糊邻域信息颗粒；多标签邻域粗糙集与模糊邻域粗糙集相结合，建立了多标签邻域粗糙集模型。基于代数和信息视图，提出了基于模糊邻域熵的MFNRS不确定度测度，改进了基于模糊邻域互信息的标签相关MRMR模型，用于评价候选特征的性能。Hu等人[99]提出一个基于权重特征选择的方法（RWFS），基于两种类型的变化比率，通过同时考虑两种类型的特征相关性评估比率来提供更可靠的特征排序。

5.3　其他研究方向

5.3.1　类别不平衡

类别不平衡问题已成为许多标签数据集的固有特征，其中样本及其对应的标签在数据空间上分布不均匀。多标签文本分类中的不平衡问题给多标签数据分析带来了挑战，可以从三个角度来看：标签内部、标签之间和标签集之间的不平衡。

在传统的二元类和多类问题中也普遍存在类不平衡问题。因此，该问题解决思路可以为处理多标签不平衡问题提供一些启示。Zhang等人[100]提出了一种COCOA算法来解决多标签应用中的类不平衡问题。Charte等人[101]提出了多标签不平衡度的几种复杂测量标准，同时Charte等人还提出了用于多标签数据不平衡预处理的欠采样和过采样算法（LP-RUS和LP-ROS）。Pereira等人[102]提出MLTL，是一种类似的基于启发式的方法。该方法采用经典的Tomek Link算法来解决不平衡问题，可以用作欠采样或清洗技术。MLSMOTE算法[103]讨论了一种用于多标签学习得过采样技术，该技术与SMOTE使用了类似的策略。首先，MLSMOTE算法通过MeanIR和CVIR识别少数标签；然后，为这些标签合成新样本。

计算机工程与应用 www.ceaj.org

另一个提出的方法是MLSOL[104]。该方法主要通过观察少数群体样本的局部特征来分析不平衡，而不是整个数据集的不平衡。在文献[105]中提出了一种适应方法来解决MLC中的不平衡问题，它基于非对称阶段损失函数来动态调整正负样本的损失成本。Rastogi等人[106]通过构建标签权重矩阵来处理类不平衡问题，权重估计由标签出现、缺席和未观察的频率来指导，利用类不平衡敏感权值和辅助标签相关性，引入带有歧视性标签权值的加权平方损失函数，指导缺失标签补全。

总之，通过采样实现了多标签问题种类不平衡的常见直观处理，需要进一步研究如何在抽样中使用标签相关性。此外，通过集成学习和成本敏感性，可以尽可能消除类别不平衡的不利影响。

5.3.2 标签丢失

在许多实际应用中，由于以下两个主要原因，获得训练集中所有样本的所有真实相关标签是不切实际的[107-108]。一方面，许多应用程序包含许多需要大量标签类；另一方面，不同标签的含义可能会重叠，因此很难完全区分。因此，基于此类部分标签的数据的学习模型可能无法准确捕获标签相关性以及标签与特征之间的关系。Taha等人[109]提出了基于聚合特征和标签图的缺失标签处理方法（GB-AS）和基于统一图的缺失标签传播方法（UG-MLP）。一方面，GB-AS算法根据基于特征的加权表示和基于标签的加权表示两种文档级别的相似度，获得初始标签矩阵。另一方面，引入UG-MLP构造一个混合图，将GB-AS和标签相关结合到一个单一的基础上，从不完整的训练数据中获取高阶标签相关性，并将其用于补充缺失的标签矩阵，指导多标签分类模型的建立。Ai等人[110]提出了一种改进的MLTSVM（LSFML-MLTSVM），利用标签缺失的标签特定特征。LSFML-MLTSVM首先通过半监督聚类分析提取标签特异性特征，然后获得样本的结构信息和边缘分布的几何信息。Sun等人[111]提出了一种基于两阶段邻域的多标签分类方法，用于邻域决策系统中缺失标签的不完全数据分类。首先，为了解决人工选取邻域半径的问题，以及在邻域内平衡样本，定义了基于特征分布函数的邻域半径，分别通过可识别矩阵和不可识别矩阵计算样本间的异同，在此基础上，提出了一种缺失特征值的恢复方法；其次，考虑到特征间的非线性关系，基于高斯核函数研究了样本间基于邻域的模糊相似关系；在模糊相似关系矩阵、标签特定特征矩阵和标签相关矩阵的综合基础上，提出了基于回归模型的目标函数，给出了基于梯度下降策略的标签特定特征矩阵和标签相关矩阵的最优解，并在第二阶段提出了一种新的缺少标签的多标签分类方法；最后，设计了两阶段多标签分类算法。

5.3.3 标签压缩

由于许多实际的多标签问题中的标签数量可以达到数万个，因此许多研究已经将注意力转移到涉及大量标签的多标签分类上，过多的标签可能会给算法带来相当大的时间和空间成本。此外，许多成熟和常见的算法，如BR[14]和ECC[18]不适用于处理这些过多的标签。为了解决这个问题，研究人员提出了标签的空间尺寸压缩技术，将高维标签压缩到低维标签空间中，并在低维标签空间中训练分类模型以减少计算负担。当然，低维标签空间中的预测结果必须恢复到原始特征空间中。根据现有研究，标签压缩不仅可以缩短算法的运行时间，而且可以提高分类效果。Cao等人[112]提出了一种新的标签压缩编码方法，同时考虑特征和标签信息。基于标签变换的标签压缩算法具有理论基础强、易于实现等优点。但是，转换后的标签缺乏原始标签的含义，因此它们很难相互连接。基于标签子集的标签压缩算法经常使用群稀疏学习、随机抽样、布尔矩阵分解等。这些算法可以获取低维标签，或者标签直接来自原始标签，或者可以完全恢复原始特征空间。Yu等人[113]介绍了一种PML方法（PML-LCom），使用标签压缩来有效地从部分多标签数据中学习。PML-LCom首先将观察到的标签数据矩阵分解为潜在的相关标签矩阵和不相关的标签矩阵，然后将相关的标签矩阵分解为两个低秩矩阵，一个对样本的压缩标签进行编码，另一个对潜在的标签相关性进行探究；然后，对压缩后的标签矩阵对多标签预测器的系数矩阵进行优化。Yang等人[114]提出一种多同步压缩变换方法（MSST），对处理后的数据进行变换。在同步压缩变换的基础上，采用迭代重分布代替原始数据，完成模式识别。此外，针对样本的特征相似度，对压缩后的标签矩阵进行正则化，并对标签矩阵和预测器进行了一致性优化。因此，标签压缩算法具有很强的解释力。表7列举了其他研究方向上的部分模型分析。

6 总结与展望

多标签文本分类的研究随着深度学习的到来取得了丰硕的成果，尤其是在BERT的到来之后，大大提高了相关研究的准确性。尽管该领域已经有相当成熟和实用的技术，但仍有一些棘手的问题值得研究人员共同探索：

缺乏数据集和低质量数据集问题。多标签文本分类比单标签文本分类要复杂得多，因此数据集资源的缺乏极大地限制了研究人员对模型的开发，并且特定领域如医疗、法律、金融和建筑的数据集十分匮乏；其次，由于该领域的当前数据集普遍存在数据分布不均匀的问题，因此主要表现为长尾问题，即同一数据集中的大多数文档仅与一个或极少数的标签相关。因此，创建更多高质量的数据集是一个值得长期讨论的问题。

文本相关标签的动态划分问题。目前，多标签文本

计算机工程与应用 www.ceaj.org

表7 其他研究方向

Table 7 Other research directions

类别	文献	模型/方法	简要介绍	年份
类别不平衡	[103]	MLSMOTE	通过MeanIR和CVIR识别少数标签,然后为这些标签合成新样本	2015
	[105]	自适应方法	基于非对称阶段损失函数来动态调整正负样本的损失成本	2019
	[104]	MLSOL	通过观察少数群体样本的局部特征来分析不平衡,而不是整个数据集的不平衡	2019
	[102]	MLTL	采用经典的Tomek Link算法来解决不平衡问题,可以用作欠采样或清洗技术	2020
	[106]	标签权重矩阵	权重估计由标签出现、缺席和未观察的频率来指导。利用类不平衡敏感权值和辅助标签相关性,引入带有歧视性标签权值的加权平方损失函数,指导缺失标签补全	2022
标签丢失	[109]	GB-AS	采用GB-AS算法,根据基于特征的加权表示和基于标签的加权表示两种文档级别的相似度,获得初始标签矩阵。引入UG-MLP构造一个混合图,将GB-AS和标签相关结合到一个单一的基础上,从不完整的训练数据中获取高阶标签相关性,并将其用于补充缺失的标签矩阵,指导多标签分类模型的建立	2022
	[110]	LSFML-MLTSVM	首先通过半监督聚类分析提取标签特异性特征;然后获得样本的结构信息和边缘分布的几何信息	2022
	[111]	两阶段邻域	用于邻域决策系统中缺失标签的不完全数据分类	2022
标签压缩	[113]	PML-LCom	使用标签压缩来有效地从部分多标签数据中学习	2015
	[112]	标签压缩编码	利用Hilbert-Schmidt独立性准则最大化特征和标签之间的依赖性,从而同时考虑特征和标签信息	2020
	[114]	MSST	对处理后的数据进行变换。在同步压缩变换的基础上,采用迭代重分布代替原始数据,完成模式识别	2021

分类主要依靠监督学习,标签发生变化,就需要对模型进行重新训练,适应变化,但重新标记数据集或训练模型都需要很高的成本。因此,如何低成本、快速地使训练好的模型适应标签的变化是一个值得考虑的问题。

极端多标签文本分类问题。极端多标签文本分类(XMC)目的是从一个极大的标签集合中为给定的文本找到相关的标签。XMC的主要难点是文本标签的数目非常多。目前提出模型的内存占用随着标签空间的变大而变大。因此,如何减小极端多标签文本分类模型的大小是未来主要研究方向之一。

层级多标签文本分类问题。许多现实世界的文本分类任务通常处理以层次结构或分类法组织的大量紧密相关的类别。当需要处理大量紧密相关的类别时,层级多标签文本分类(HMTC)变得非常具有挑战性。层次标签概念:一级标签包含二级标签,二级标签包含三级标签。HTMC的难点在于考虑垂直类别相关性的平面多标签以及同一级别类别之间的水平相关性;充分地建模层级依赖关系,提高各层级标签,尤其是下层长尾标签的预测性能,此外,整个层次结构中所有类别的结构特征及其类别标签的单词语义对于提高大量紧密相关类别的文本分类准确性非常有帮助。因此,如何设计这样一个模型去解决这些问题是未来亟待解决的一个难点。

小样本多标签文本分类也是当前和未来研究热点,在实际的应用场景中,得到文本数据可能会面临分类类别多、样本数据小和文本短等问题。小样本数据集的构建,也更利于模型可以应用于不同的领域。

7 结语

本文对近年来多标签文本分类概念、流程、方法和研究方向的文献进行了综述。将多标签文本分类方法分为传统机器学习方法和深度学习方法;研究方向划分为标签相关性、特定标签特性、类别不平衡、标签丢失和标签压缩;最后对多标签文本分类的挑战和未来方向进行了讨论。

参考文献:

[1] TSOUMAKAS G,KATAKIS I.Multi-label classification: an overview[J].International Journal of Data Warehousing and Mining(IJDWM),2007,3:1-13.

[2] ZENG Q,ZHAO X,HU X,et al.Learning emotional word embeddings for sentiment analysis[J].Journal of Intelligent & Fuzzy Systems,2021,40:9515-9527.

[3] YAO L,MAO C,LUO Y.Graph convolutional networks for text classification[C]//Proceedings of the AAAI Conference on Artificial Intelligence,2019,33:7370-7377.

[4] KALCHBRENNER N,GREFENSTETTE E,BLUNSOM P.A convolutional neural network for modelling sentences[J].arXiv:1404.2188,2014.

[5] LEE J Y,DERNONCOURT F.Sequential short-text classification with recurrent and convolutional neural networks[J].arXiv:1603.03827,2016.

[6] XIAO L,HUANG X,CHEN B,et al.Label-specific document representation for multi-label text classification[C]//Proceedings of the 2019 Conference on Empirical Methods in Natural Language Processing and the 9th International Joint Conference on Natural Language Processing(EMNLP-IJCNLP),2019:466-475.

[7] YANG Y,REN G.HanLP-based technology function matrix construction on Chinese process patents[J].International Journal of Mobile Computing and Multimedia Communica-

计算机工程与应用 www.ceaj.org

tions（IJMCMC），2020，11：48-64.
[8] MIKOLOV T，CHEN K，CORRADO G，et al.Efficient estimation of word representations in vector space[J].arXiv：1301.3781，2013.
[9] PENNINGTON J，SOCHER R，MANNING C D.Glove：global vectors for word representation[C]//Proceedings of the 2014 Conference on Empirical Methods in Natural Language Processing（EMNLP），2014：1532-1543.
[10] RADFORD A，NARASIMHAN K，SALIMANS T，et al. Improving language understanding by generative pretraining[EB/OL].（2018）[2020-11-30].https：//s3-us-west-2.amazonaws.com/openaiassets/researchcovers/languageunsupervised/languageunderstandingpaper.pdf.
[11] DEVLIN J，CHANG M W，LEE K，et al.Bert：pre-training of deep bidirectional transformers for language understanding[J].arXiv：1810.04805，2018.
[12] QIU X，SUN T，XU Y，et al.Pre-trained models for natural language processing：a survey[J].Science China Technological Sciences，2020，63：1872-1897.
[13] GHOSH S，DESARKAR M S.Class specific TF-IDF boosting for short-text classification：application to short-texts generated during disasters[C]//Companion Proceedings of the The Web Conference 2018，2018：1629-1637.
[14] BOUTELL M R，LUO J，SHEN X，et al.Learning multi-label scene classification[J].Pattern Recognition，2004，37：1757-1771.
[15] GODBOLE S，SARAWAGI S.Discriminative methods for multi-labeled classification[C]//Pacific-Asia Conference on Knowledge Discovery and Data Mining，2004：22-30.
[16] ALVARES-CHERMAN E，METZ J，MONARD M C.Incorporating label dependency into the binary relevance framework for multi-label classification[J].Expert Systems with Applications，2012，39：1647-1655.
[17] TSOUMAKAS G，DIMOU A，SPYROMITROS E，et al. Correlation-based pruning of stacked binary relevance models for multi-label learning[C]//Proceedings of the 1st International Workshop on Learning from Multi-Label Data，2009：101-116.
[18] READ J，PFAHRINGER B，HOLMES G，et al.Classifier chains for multi-label classification[C]//Joint European Conference on Machine Learning and Knowledge Discovery in Databases，2009：254-269.
[19] TSOUMAKAS G，VLAHAVAS I.Random k-labelsets：an ensemble method for multilabel classification[C]//European Conference on Machine Learning，2007：406-417.
[20] READ J，PFAHRINGER B，HOLMES G.Multi-label classification using ensembles of pruned sets[C]//2008 Eighth IEEE International Conference on Data Mining，2008：995-1000.
[21] HüLLERMEIER E，FüRNKRANZ J，CHENG W，et al. Label ranking by learning pairwise preferences[J].Artificial Intelligence，2008，172：1897-1916.
[22] FüRNKRANZ J，HüLLERMEIER E，LOZA MENCíA E，et al.Multilabel classification via calibrated label ranking[J].Machine Learning，2008，73：133-153.
[23] ZHANG M L，ZHOU Z H.ML-KNN：a lazy learning approach to multi-label learning[J].Pattern Recognition，2007，40：2038-2048.
[24] HUANG J，LI G，WANG S，et al.Categorizing social multimedia by neighborhood decision using local pairwise label correlation[C]//2014 IEEE International Conference on Data Mining Workshop，2014：913-920.
[25] CLARE A，KING R D.Knowledge discovery in multi-label phenotype data[C]//European Conference on Principles of Data Mining and Knowledge Discovery，2001：42-53.
[26] BLOCKEEL H，DE RAEDT L，RAMON J.Top-down induction of clustering trees[J].arXiv：cs/0011032，2000.
[27] ZHANG M L，ZHOU Z H.Multilabel neural networks with applications to functional genomics and text categorization[J].IEEE Transactions on Knowledge and Data Engineering，2006，18：1338-1351.
[28] ELISSEEFF A，WESTON J.A kernel method for multilabelled classification[C]//Advances in Neural Information Processing Systems，2001.
[29] KIM Y.Convolutional neural networks for sentence classification[J].arXiv：1408.5882，2014.
[30] LIU J，CHANG W C，WU Y，et al.Deep learning for extreme multi-label text classification[C]//Proceedings of the 40th International ACM SIGIR Conference on Research and Development in Information Retrieval，2017：115-124.
[31] SHIMURA K，LI J，FUKUMOTO F.HFT-CNN：learning hierarchical category structure for multi-label short text categorization[C]//Proceedings of the 2018 Conference on Empirical Methods in Natural Language Processing，2018：811-816.
[32] YANG W，LI J，FUKUMOTO F，et al.HSCNN：a hybrid-siamese convolutional neural network for extremely imbalanced multi-label text classification[C]//Proceedings of the 2020 Conference on Empirical Methods in Natural Language Processing（EMNLP），2020：6716-6722.
[33] NAM J，LOZA MENCíA E，KIM H J，et al.Maximizing subset accuracy with recurrent neural networks in multi-label classification[C]//Advances in Neural Information Processing Systems，2017.
[34] CHEN G，YE D，XING Z，et al.Ensemble application of convolutional and recurrent neural networks for multi-label text categorization[C]//2017 International Joint Conference on Neural Networks（IJCNN），2017：2377-2383.
[35] YANG P，LUO F，MA S，et al.A deep reinforced sequence-to-set model for multi-label classification[C]//Proceedings

计算机工程与应用 www.ceaj.org

of the 57th Annual Meeting of the Association for Computational Linguistics，2019：5252-5258.

[36] LIN J，SU Q，YANG P，et al.Semantic-unit-based dilated convolution for multi-label text classification[J].arXiv：1808.08561，2018.

[37] YANG P，SUN X，LI W，et al.SGM：sequence generation model for multi-label classification[J].arXiv：1806.04822，2018.

[38] YANG Z，YANG D，DYER C，et al.Hierarchical attention networks for document classification[C]//Proceedings of the 2016 Conference of the North American Chapter of the Association for Computational Linguistics：Human Language Technologies，2016：1480-1489.

[39] HONG M，WANG M，LUO L，et al.Combining gated recurrent unit and attention pooling for sentimental classification[C]//Proceedings of the 2018 2nd International Conference on Computer Science and Artificial Intelligence，2018：99-104.

[40] LI Y，CAI Y，LEUNG H F，et al.Improving short text modeling by two-level attention networks for sentiment classification[C]//International Conference on Database Systems for Advanced Applications，2018：878-890.

[41] YOU R，ZHANG Z，WANG Z，et al.Attentionxml：label tree-based attention-aware deep model for high-performance extreme multi-label text classification[C]//Advances in Neural Information Processing Systems，2019.

[42] YAO C，CAI M.A novel optimized convolutional neural network based on attention pooling for text classification[C]//Journal of Physics：Conference Series，2021.

[43] XIAO Y，LI Y，YUAN J，et al.History-based attention in Seq2Seq model for multi-label text classification[J].Knowledge-Based Systems，2021，224：107094.

[44] LIU B，LIU X，REN H，et al.Text multi-label learning method based on label-aware attention and semantic dependency[J].Multimedia Tools and Applications，2022，81：7219-7237.

[45] SONG R，LIU Z，CHEN X，et al.Label prompt for multi-label text classification[J].Applied Intelligence，2022，53：8761-8775.

[46] CHANG W C，YU H F，ZHONG K，et al.Taming pretrained transformers for extreme multi-label text classification[C]//Proceedings of the 26th ACM SIGKDD International Conference on Knowledge Discovery & Data Mining，2020：3163-3171.

[47] GONG J，TENG Z，TENG Q，et al.Hierarchical graph transformer-based deep learning model for large-scale multi-label text classification[J].IEEE Access，2020，8：30885-30896.

[48] JIANG T，WANG D，SUN L，et al.Lightxml：transformer with dynamic negative sampling for high-performance extreme multi-label text classification[C]//Proceedings of the AAAI Conference on Artificial Intelligence，2021：7987-7994.

[49] YE C，ZHANG L，HE Y，et al.Beyond text：incorporating metadata and label structure for multi-label document classification using heterogeneous graphs[C]//Proceedings of the 2021 Conference on Empirical Methods in Natural Language Processing，2021：3162-3171.

[50] CHEN Q，DU J，ALLOT A，et al.LitMC-BERT：transformer-based multi-label classification of biomedical literature with an application on COVID-19 literature curation[J].IEEE/ACM Transactions on Computational Biology and Bioinformatics，2022，19（5）：2584-2595.

[51] ZHANG R，WANG Y S，YANG Y，et al.Exploiting local and global features in transformer-based extreme multi-label text classification[J].arXiv：2204.00933，2022.

[52] KIPF T N，WELLING M.Semi-supervised classification with graph convolutional networks[J].arXiv：1609.02907，2016.

[53] LIU P，QIU X，HUANG X.Recurrent neural network for text classification with multi-task learning[J].arXiv：1605.05101，2016.

[54] VELIKOVI P，CUCURULL G，CASANOVA A，et al.Graph attention networks[J].arXiv：1710.10903，2017.

[55] PAL A，SANKARASUBBU M，SELVAKUMAR M.Multi-label text classification using attention-based graph neural network[J].arXiv：2003.11644，2020.

[56] DING K，WANG J，LI J，et al.Be more with less：hypergraph attention networks for inductive text classification[C]//Proceedings of the 2020 Conference on Empirical Methods in Natural Language Processing（EMNLP），2020：4927-4936.

[57] ZONG D，SUN S.GNN-XML：graph neural networks for extreme multi-label text classification[J].arXiv：2012.05860，2020.

[58] ZHENG S，ZHOU J，MENG K，et al.Label-dividing gated graph neural network for hierarchical text classification[C]//2022 International Joint Conference on Neural Networks（IJCNN），2022：1-8.

[59] ZHOU C，SUN C，LIU Z，et al.A C-LSTM neural network for text classification[J].arXiv：1511.08630，2015.

[60] ZHANG R，LEE H，RADEV D.Dependency sensitive convolutional neural networks for modeling sentences and documents[C]//Proceedings of NAACL-HLT，2016：1512-1521.

[61] LIU W，PANG J，LI N，et al.Research on multi-label text classification method based on tALBERT-CNN[J].International Journal of Computational Intelligence Systems，2021，14：1-12.

[62] YAN Y，LIU F A，ZHUANG X，et al.An R-Transformer_BiLSTM model based on attention for multi-label text classification[J].Neural Processing Letters，2022：1-24.

计算机工程与应用 www.ceaj.org

[63] XIAO L,ZHANG X,JING L,et al.Does head label help for long-tailed multi-label text classification[C]//Proceedings of the AAAI Conference on Artificial Intelligence, 2021:14103-14111.

[64] ZHANG X,ZHANG Q W,YAN Z,et al.Enhancing label correlation feedback in multi-label text classification via multi-task learning[J].arXiv:2106.03103,2021.

[65] KHATAEI MARAGHEH H,GHAREHCHOPOGH F S, MAJIDZADEH K,et al.A new hybrid based on long short-term memory network with spotted hyena optimization algorithm for multi-label text classification[J].Mathematics,2022,10:488.

[66] GIBAJA E,VENTURA S.A tutorial on multilabel learning[J].ACM Computing Surveys(CSUR),2015,47:1-38.

[67] BAO J,WANG Y,CHENG Y.Asymmetry label correlation for multi-label learning[J].Applied Intelligence,2022,52: 6093-6105.

[68] HUANG R,KANG L.Local positive and negative label correlation analysis with label awareness for multi-label classification[J].International Journal of Machine Learning and Cybernetics,2021,12:2659-2672.

[69] LI Y K,ZHANG M L,GENG X.Leveraging implicit relative labeling-importance information for effective multi-label learning[C]//2015 IEEE International Conference on Data Mining,2015:251-260.

[70] MENCíA E L,FURNKRANZ J.Pairwise learning of multilabel classifications with perceptrons[C]//2008 IEEE International Joint Conference on Neural Networks(IEEE World Congress on Computational Intelligence),2008: 2899-2906.

[71] WU G,TIAN Y,LIU D.Cost-sensitive multi-label learning with positive and negative label pairwise correlations[J]. Neural Networks,2018,108:411-423.

[72] XU H,XU L.Multi-label feature selection algorithm based on label pairwise ranking comparison transformation[C]// 2017 International Joint Conference on Neural Networks (IJCNN),2017:1210-1217.

[73] ZHANG Y,ZHAO T,MIAO D,et al.Granular multilabel batch active learning with pairwise label correlation[J]. IEEE Transactions on Systems,Man,and Cybernetics: Systems,2021,52:3079-3091.

[74] WANG R,YE S,LI K,et al.Bayesian network based label correlation analysis for multi-label classifier chain[J]. Information Sciences,2021,554:256-275.

[75] HE Z F,YANG M,GAO Y,et al.Joint multi-label classification and label correlations with missing labels and feature selection[J].Knowledge-Based Systems,2019,163: 145-158.

[76] JI S,TANG L,YU S,et al.Extracting shared subspace for multi-label classification[C]//Proceedings of the 14th ACM SIGKDD International Conference on Knowledge Discovery and Data Mining,2008:381-389.

[77] XU L,WANG Z,SHEN Z,et al.Learning low-rank label correlations for multi-label classification with missing labels[C]//2014 IEEE International Conference on Data Mining,2014:1067-1072.

[78] CHE X,CHEN D,MI J.A novel approach for learning label correlation with application to feature selection of multi-label data[J].Information Sciences,2020,512:795-812.

[79] LI Q,PENG X,QIAO Y,et al.Learning label correlations for multi-label image recognition with graph networks[J].Pattern Recognition Letters,2020,138:378-384.

[80] HUANG J,LI G,WANG S,et al.Multi-label classification by exploiting local positive and negative pairwise label correlation[J].Neurocomputing,2017,257:164-174.

[81] MA J,CHIU B C Y,CHOW T W.Multilabel classification with group-based mapping:a framework with local feature selection and local label correlation[J].IEEE Transactions on Cybernetics,2020,52(6):4596-4610.

[82] ZHU Y,KWOK J T,ZHOU Z H.Multi-label learning with global and local label correlation[J].IEEE Transactions on Knowledge and Data Engineering,2017,30:1081-1094.

[83] YAN Y,LI S,ZHANG X,et al.k-Labelsets for Multimedia classification with global and local label correlation[C]//International Conference on Multimedia Modeling,2018:177-188.

[84] WENG W,WEI B,KE W,et al.Learning label-specific features with global and local label correlation for multilabel classification[J].Applied Intelligence,2022:1-17.

[85] LIU Y,CAO F.A relative labeling importance estimation algorithm based on global-local label correlations for multi-label learning[J].Applied Intelligence,2022:1-19.

[86] LIU L,ZHANG J,LI P,et al.A label correlation based weighting feature selection approach for multi-label data[C]// International Conference on Web-Age Information Management,2016:369-379.

[87] LEE J,KIM H,KIM N R,et al.An approach for multilabel classification by directed acyclic graph with label correlation maximization[J].Information Sciences,2016, 351:101-114.

[88] HU Q,PEDRYCZ W,YU D,et al.Selecting discrete and continuous features based on neighborhood decision error minimization[J].IEEE Transactions on Systems,Man,and Cybernetics,Part B(Cybernetics),2009,40:137-150.

[89] CHEN Z,LI S,YE L,et al.Multi-label classification of legal text based on label embedding and capsule network[J].Applied Intelligence,2022,53:6873-6886.

[90] WANG K.Robust cross-view embedding with discriminant structure for multi-label classification[J].IEEE Access, 2021,9:117596-117607.

计算机工程与应用 www.ceaj.org

计算机工程与应用 www.ceaj.org

[91] ZHANG M L，WU L.Lift：multi-label learning with label-specific features[J].IEEE Transactions on Pattern Analysis and Machine Intelligence，2014，37：107-120.

[92] HUANG J，LI G，HUANG Q，et al.Learning label specific features for multi-label classification[C]//2015 IEEE International Conference on Data Mining，2015：181-190.

[93] WENG W，LIN Y，WU S，et al.Multi-label learning based on label-specific features and local pairwise label correlation[J].Neurocomputing，2018，273：385-394.

[94] GUAN Y，LI W，ZHANG B，et al.Multi-label classification by formulating label-specific features from simultaneous instance level and feature level[J].Applied Intelligence，2021，51：3375-3390.

[95] FAN Y，CHEN B，HUANG W，et al.Multi-label feature selection based on label correlations and feature redundancy[J].Knowledge-Based Systems，2022，241：108256.

[96] WU Y，LIU J，YU X，et al.Neighborhood rough set based multi-label feature selection with label correlation[J].Concurrency and Computation：Practice and Experience，2022，34：e7162.

[97] WENG W，CHEN Y N，CHEN C L，et al.Non-sparse label specific features selection for multi-label classification[J].Neurocomputing，2020，377：85-94.

[98] SUN L，YIN T，DING W，et al.Feature selection with missing labels using multilabel fuzzy neighborhood rough sets and maximum relevance minimum redundancy[J].IEEE Transactions on Fuzzy Systems，2021，30：1197-1211.

[99] HU L，GAO L，LI Y，et al.Feature-specific mutual information variation for multi-label feature selection[J].Information Sciences，2022，593：449-471.

[100] ZHANG M L，LI Y K，YANG H，et al.Towards class-imbalance aware multi-label learning[J].IEEE Transactions on Cybernetics，2020，52（6）：4459-4471.

[101] CHARTE F，RIVERA A，JESUS M J D，et al.A first approach to deal with imbalance in multi-label datasets[C]//International Conference on Hybrid Artificial Intelligence Systems，2013：150-160.

[102] PEREIRA R M，COSTA Y M，SILLA JR C N.MLTL：a multi-label approach for the tomek link undersampling algorithm[J].Neurocomputing，2020，383：95-105.

[103] CHARTE F，RIVERA A J，DEL JESUS M J，et al. MLSMOTE：approaching imbalanced multilabel learning through synthetic instance generation[J].Knowledge-Based Systems，2015，89：385-397.

[104] LIU B，TSOUMAKAS G.Synthetic oversampling of multi-label data based on local label distribution[C]//Joint European Conference on Machine Learning and Knowledge Discovery in Databases，2019：180-193.

[105] LUO F F，GUO W Z，CHEN G L.Addressing imbalance in weakly supervised multi-label learning[J].IEEE Access，2019，7：37463-37472.

[106] RASTOGI R，KUMAR S.Discriminatory label-specific weights for multi-label learning with missing labels[J]. Neural Processing Letters，2022，55：1397-1431.

[107] DENDAMRONGVIT S，VATEEKUL P，KUBAT M.Irrelevant attributes and imbalanced classes in multi-label text-categorization domains[J].Intelligent Data Analysis，2011，15：843-859.

[108] WU B，JIA F，LIU W，et al.Multi-label learning with missing labels using mixed dependency graphs[J].International Journal of Computer Vision，2018，126：875-896.

[109] TAHA A Y，TIUN S，RAHMAN A H A，et al.Unified graph-based missing label propagation method for multilabel text classification[J].Symmetry，2022，14：286.

[110] AI Q，LI F，LI X，et al.An improved MLTSVM using label-specific features with missing labels[J].Applied Intelligence，2022，53：8039-8060.

[111] SUN L，WANG T，DING W，et al.Two-stage-neighborhood-based multilabel classification for incompletedata with missing labels[J].International Journal of Intelligent Systems，2022，37（10）：6773-6810.

[112] CAO L，XU J.A label compression coding approach through maximizing dependence between features and labels for multi-label classification[C]//2015 International Joint Conference on Neural Networks（IJCNN），2015：1-8.

[113] YU T，YU G，WANG J，et al.Partial multi-label learning using label compression[C]//2020 IEEE International Conference on Data Mining（ICDM），2020：761-770.

[114] YANG Y，ZHOU J，LIU J，et al.Epileptic seizure detection based on multi-synchrosqueezing transform and multi-label classification[C]//Signal and Information Processing，Networking and Computers，2023：1017-1024.

Research Review and Prospect of Multi-label Text Classification

Zhang Wenfeng [1, 2], Xi Xuefeng [1, 2, 3], Cui Zhiming [1, 2, 3], Zou Yichen [1, 2], Luan Jinkuan [1, 2]

1. School of Electronic and Information Engineering, Suzhou University of Science and Technology, Suzhou, Jiangsu 215000

2. Suzhou Virtual Reality Intelligent Interaction and Application Technology Key Laboratory 215000, Suzhou, Jiangsu Province

3. Suzhou Smart City Research Institute, Suzhou, Jiangsu 215000

Abstract: Text classification (TC) is an important fundamental task in the field of natural language processing (NLP), and multi-label text classification (MLTC) is an important branch of TC. In order to have an in-depth understanding of the multi-label text classification field, the concept and process of multi-label text classification are introduced. The multi-label text classification methods in recent years are divided into those based on traditional machine learning methods and those based on deep learning methods. The commonly used datasets and evaluation indicators in the multi-label text classification field are sorted out, and the advantages and existing problems of some multi-label text classification models are analyzed. The research directions of multi-label text classification are introduced: label correlation, specific label characteristics, class imbalance, label loss and label compression. The difficulties and future development directions of multi-label text classification are summarized and prospected.

Key words: Multi-label text classification; Deep learning; Label correlation; Specific label characteristics Category imbalance

Literature Mark Code: A Chinese Library Classification: TP391 doi: 10.3778/j.issn.1002-8331.2210-0446

Review and Prospect of Multi-Label Text Classification Research

ZHANG Wenfeng [1, 2], XI Xuefeng [1, 2, 3], CUI Zhiming [1, 2, 3], ZOU Yichen [1, 2], LUAN Jinquan [1, 2]

1.School of Electronic & Information Engineering, Suzhou University of Science and Technology, Suzhou, Jiangsu 215000, China

2.Suzhou Key Laboratory of Virtual Reality Intelligent Interaction and Application Technology, Suzhou, Jiangsu 215000, China

3.China Suzhou Smart City Research Institute, Suzhou, Jiangsu 215000, China

Abstract: Text classification (TC) is an important basic task in the field of natural language processing (NLP), and multi-label text classification (MLTC) is an important branch of TC. In order to have a deep understanding of the field of multi-label text classification, the concept and process of multi-label text classification are introduced. In recent years, multi-label text classification methods are divided into traditional machine learning methods and deep learning methods. The commonly used datasets and evaluation indicators in the field of multi-label text classification are sorted out, and the advantages and problems of some multi-label text classification models are analyzed. The research directions of multi-label text classification: label correlation, specific label characteristics, category imbalance, label loss and label compression. Finally, the difficulties of multi-label text classification are summarized and the future development direction is prospected.

Key words: Multi-label Text Classification deep learning; label correlation; features label-specific; class imbalance

Text classification is the process of dividing the content of a text into one or more categories and is a fundamental task in NLP. In the real world, due to the complex and changeable environment of text data and the existence of polysemous objects, text classification faces many severe challenges. Traditional single-label text classification methods cannot fully meet the needs of users, and multi-label learning methods have emerged [1]. Multi-label learning refers to assigning the most relevant class labels from the label set to each The process of this text can visually reflect various semantic information contents of the ambiguous object. For example, a news report about the Coronavirus Disease 2019 ("COVID-19") would belong to multiple categories such as "healthcare" and "economic crisis" or "national security".

The multi-label text classification problem is an important research direction in multi-label learning, which is mainly applied in sentiment analysis [2], topic annotation [3], question answering [4], and dialogue rows

基金项目：国家自然科学基金（61876217， 62176175）；江苏省“六大人才高峰”高层次人才项目（XYDXX-086）；苏州市科技计划项 目（SGC2021078）。

作者简介：张文峰（1999—），男，硕士研究生，CCF 学生会员，研究方向为自然语言处理、文本分类，E-mail：2906213721@qq.com；奚雪峰（1978—），通信作者，男，博士，教授，CCF 会员，研究方向为自然语言处理、多模态机器学习、软件工程；崔志明（1961—），男，博士，教授，CCF会员，研究方向为知识挖掘、机器学习；邹逸晨（2001—），男，CCF学生会员，研究方向为 自然语言处理；栾进权（2001—），男，研究方向为软件工程。

收稿日期：2022-10-27 修回日期：2023-02-20 文章编号：1002-8331（2023）18-0028-21

For classification [5]. Its text data has the following characteristics: One text can belong to multiple labels, so it is necessary to capture different levels and aspects of semantic features; when the document is long, semantic information will be hidden in redundant content; most texts only belong to a small number of labels [6]; the text data is unbalanced, labels are lost, and the label set is too large. Based on the above problems, researchers mainly focus on several aspects: how to fully apply the relevance of labels; how to capture effective information and extract relevant feature information; and how to alleviate the problems of category imbalance, label loss, and label compression.

The main contributions of this article are summarized as follows:

(1) Elaborate on the concepts and processes of multi-label text classification.

(2) Review the multi-label text classification methods in recent years, sort out the common datasets and evaluation indicators for multi-label text classification; and analyze the advantages and existing problems of some models or methods.

(3) Review and sort out the research directions in the field of multi-label text classification.

(4) Summarize and prospect the current difficulties and future research directions of multi-label text classification.

1 Multi-label text classification

1.1 Multi-label Text Classification Concept

Given an input space X = $X1 \times X2 \times \ldots \times Xd$ of dimension d *and an* output space Y = {λ1, λ2, ..., λq} with $q > 1$. *E*ach label |λi| = 2 is the cardinality. A multi-label example can be defined as a pair (x, Y), where x = (x1, x2, ..., xd) ∈ X and Y ∈ Y is called the label set. D = {(xi, Yi) | 1 ⩽ i ⩽ m} is a multi-label dataset composed of a group of m texts.

Multi-label text classification is to construct a predictive model *h*: X → 2Y, which will provide a set of relevant labels for the text. Each text may have several labels associated with it from the previously defined label set. Therefore, for each *x* ∈ *X*, there is a dichotomy (Y, *Y*') of the label space *Y* ∈ *Y*, where *Y* = *h (x)* is the set of relevant labels and *Y*' is the set of irrelevant labels. Multi-label text classification is shown in Figure 1.

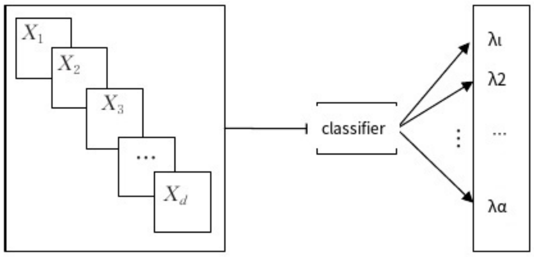

Figure 1 The concept of multi-label text classification

Fig.1 Multi-label text classification concept

1.2 Multi-label text classification process

The process of multi-label text classification is shown in Figure 2.

(1) Dataset

The commonly used datasets in the field of multi-label text classification will be introduced in detail in Chapter 3.

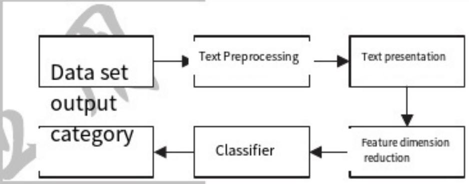

Figure 2 Multi-label Text Classification Process

Fig.2 Multi-label text classification process

(2) Text preprocessing

Text preprocessing refers to a series of operations such as removing stop words, word segmentation, and part-of-speech restoration on the original data set. However, there are already very mature technologies for the above processing at present. If word segmentation is required, ready-made tools such as jieba and HanLP [7] can be directly used, and researchers do not need to spend too much effort on this research.

(3) Text Representation

Text representation is the cornerstone of the field of natural language processing. Since machines cannot directly recognize natural language, converting natural texts into expressions that machines can understand is the task of text representation. The generation of text representation can be understood as encoding text data according to a certain model. Its development has roughly gone through several stages, such as One-hot, Bag of Words (BOW), Language Model (LM), Word2Vec [8], Glove [9], etc. One-hot representation generates word vectors through binary encoding, with each dimension indicating only whether the corresponding word in the dictionary is present at that position. This method not only leads to the curse of dimensionality, causing data sparsity, but also results in insufficient feature extraction of text semantics. BOW representation replaces binary data with word frequency data based on One-hot, but still fails to solve the problems of the curse of dimensionality and semantic loss. The LM model uses conditional probabilities to express the associations between words in a text sequence, but the semantic representation method of the LM model is rather primitive, thus leading to the development of the Word2Vec model. The Word2Vec model consists of the Continuous Bag of Words (CBOW) model and the Skip-Gram model. The Word2Vec model uses a structure similar to a neural network to establish the process of relationships between words. In recent years, emerging text representation methods have focused on context-based word embeddings, such as Language Model Embeddings (ELMo), Generative Pre-training (GPT) [10] methods, and the use of bidirectional encoders for text representation in BERT [11]. ELMo first learns word embeddings for each word through a language model and dynamically adjusts the embeddings for the context, solving the problem of polysemy and simultaneously achieving the function of semantic relation judgment. In terms of feature extraction, ELMo adopts LSTM, but later a new feature extractor, Transformer, was proposed, and the feature extraction ability of Transformer has been proven to be superior to that of LSTM. Therefore, GPT was proposed based on Transformer as the feature handler. The GPT model is pre-trained through a language model and then fine-tuned for text representation; however, GPT is a unidirectional language model that only pays attention to the context of a word and does not consider the subsequent context, so its semantic understanding is not comprehensive. To simultaneously consider semantic information in both word order directions, BERT was proposed for training on large datasets, thereby enabling the learning of more reasonable word representations, including context information [12].

(4) Feature dimension reduction

After vectorization processing, the text features are relatively sparse and the dimension is relatively high. Common methods for feature dimension reduction include TF-IDF [13] and mutual information, etc. After the proposal of Transformer, most of them have adopted Transformer as the feature dimension reduction module.

(5) Classifier and Output Categories

The data after dimension reduction of the features is sent into the classifier for model training. Then, the output categories of the model are predicted using the test set, and the validation set and evaluation indicators are used to judge the quality of the model.

2 Multi-label text classification methods

Multi-label text classification methods can be mainly divided into: traditional machine learning-based and deep learning-based. Traditional machine learning methods can be classified into problem transformation methods and algorithm adaptation methods based on the perspective of solution strategies. Problem transformation methods transform multi-label problems into multiple single-label sub-problems, and then these sub-problems are directly solved using mature single-label algorithms. Therefore, problem transformation methods are independent of specific algorithms and can select the appropriate algorithm according to the actual situation. Algorithm adaptation methods expand the existing single-label algorithms to enable them to be directly applied to multi-label data. The detailed classification of multi-label text classification methods is shown in Figure 3.

2.1 Problem Transformation Method

At present, the problem transformation algorithms are mainly based on the following three methods: (1) Binary relevance (BR) transformation method, (2) label power set (LP) transformation method, and (3) pairwise method (PW).

2.1.1 BR Conversion Method

BR is the most representative problem transformation model, which establishes binary classification models for each label. However, the basic BR model [14] ignores label correlations. To utilize this correlation between labels in the BR framework, a common method is to add the labels as additional features to the original features and then construct the corresponding classifier. These improved BR models can be divided into two types. The first type constructs a two-layer BR, where the first layer shows the same approach as the original BR. In the second layer, the output of the first layer is added as an additional feature to the original features. Then, based on these augmented features, a binary classifier for each label is learned. The output of the second layer is used as the predicted label. A BR model using this approach is called a stack-based BR model. The second type connects all binary classifiers into a chain, where a classifier on the chain adds the outputs of all previous classifiers as additional features to the original features. Such a framework is called a classifier chain model. The BR model, stack-based BR model, and classifier chain model are detailed below.

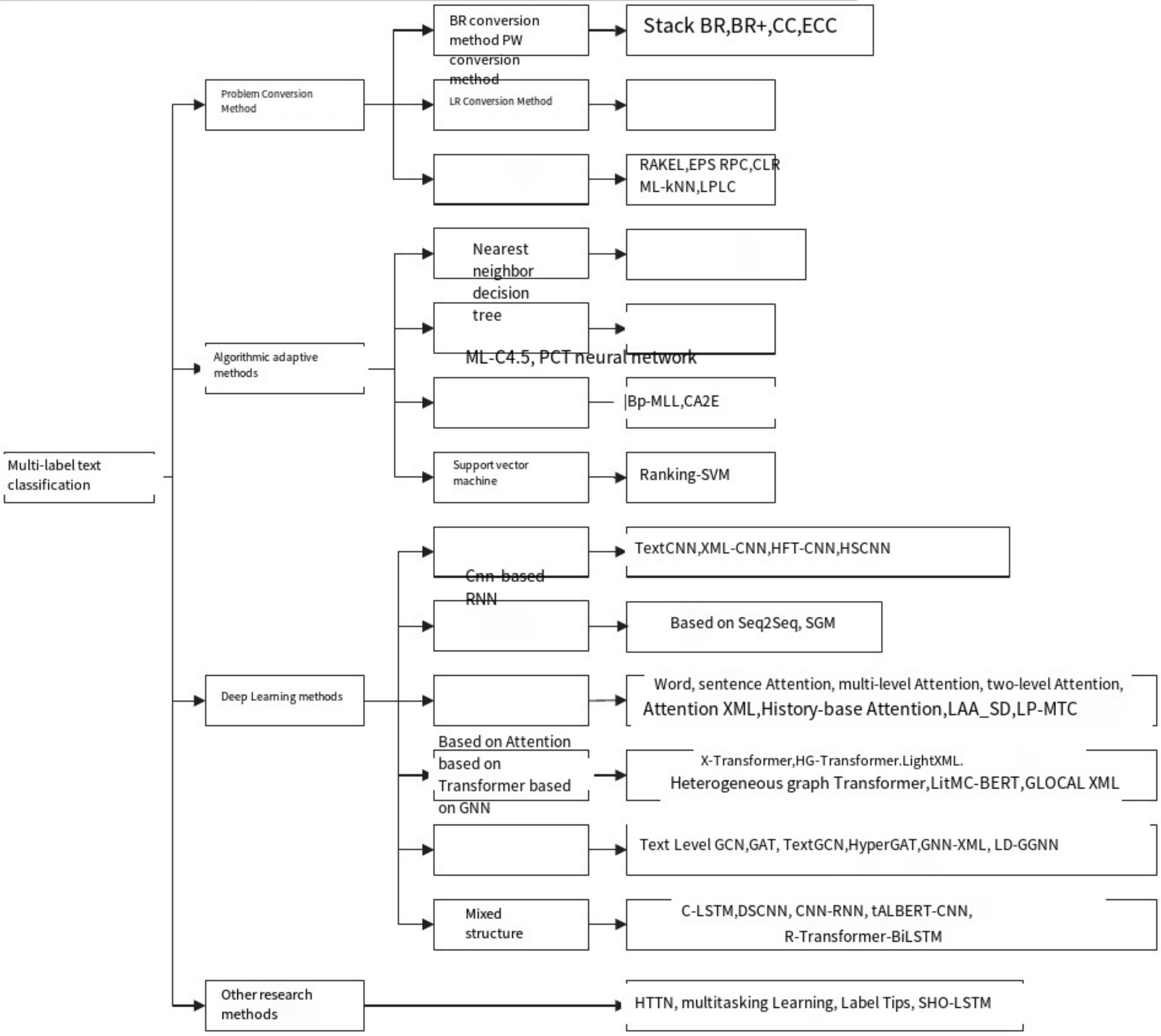

Figure 3 Multi-label Text Classification Method

Fig.3 Multi-label text classification method

(1) BR Model

In 2004, Boutell et al. [14] first proposed the basic BR model. It converts the multi-label problem into several binary classification sub-problems, and all sub-problems share the same feature space, but the label spaces are different. Although the BR algorithm is simple, intuitive and widely applied, its predictive performance is poor because it ignores the relationships between labels.

(2) Stack-based BR model

Godbole et al. [15] introduced the stack-based learning strategy in ensemble learning into the BR algorithm, considering the label correlation. During the training process, the stack-based BR model established a two-layer BR model: The first layer was the base layer, which was the same as the traditional BR learning process, and a corresponding binary classification model was assigned to each label. In the second layer at the meta-level, the predicted labels of all binary classification models in the base layer were added to the original feature space, and each label was learned again on these expanded features to obtain the corresponding binary classification model. This stack-based BR algorithm assumes that any label is related to all labels. However, this is ineffective in most cases. Relevant studies include the algorithm BR+ [16] and the algorithm BR based on pruning and stacking [17].

(3) Classifier Chain Model

Read et al. [18] first proposed the classifier chain (CC) model. Unlike the BR algorithm, the labels in the chain add the binary classifier outputs of all previous labels to the original feature space as new features for training. The CC model has two obvious drawbacks: First, the effect of the classifier is greatly affected by the label sequence, and different sequences bring significantly different classification effects; the other is that the current label may be irrelevant to the labels in the previous part of the sequence, so it may introduce noise by using the outputs of all previous labels. In the same literature, Read proposed the ensemble framework of CC (ECC). In ECC, the mean prediction results of CC models using multiple random label sequences can solve the influence of random label sequences on classification to a certain extent. However, this computational cost doubles. Cheng et al. proposed the probabilistic classifier chain (PCC) model and pointed out that classifier chains based on Hamming or rank loss functions can be obtained from the conditional joint distribution of labels. Besides improving the effect of the CC algorithm based on probabilistic derivation, there are some other methods to find the best or better label sequences. For example, GA-PratCC searches for the best label sequence using the genetic algorithm; OOCC finds the best label sequence for each sample from the *k* nearest neighbors.

2.1.2 LR conversion method

The LP transformation method [19] regards the combination of all labels in the training set as a class, and then transforms the multi-label problem into a single-label multi-class problem. After obtaining the classifier from the learning, the unseen instance is the input, and the class is the output. This class corresponds to a label set, which covers all the labels to which the instance belongs.

The LP transformation method can only predict the label sets that appear in the training set. Additionally, when there are many labels, there may be many label sets. Therefore, many sets may have some of the same instances, leading to class imbalance. These problems not only increase the time cost of learning but also reduce the model performance. To address these issues, two well-known algorithms, RAkEL [19] and EPS [20], have been proposed.

The Random *k* Label Sets (RAkEL) algorithm trains each label set through the LP transformation method. RAkEL can achieve more accurate performance than BR and LP. Additionally, RAkEL reduces the number of models that must be learned. During the prediction process, all learners obtained using the LP method are used to predict unseen instances, and the average of all prediction results is taken. Therefore, RAkEL is an ensemble learning method.

The EPS algorithm calculates the number of instances (Count*(Ri)*) related to the label set (*Ri*). If *Count(*Ri) is higher than the *specified threshold*, all instances related to Ri *are* added *to* the *training* set. If Count(Ri) is lower than the threshold, the counting of instances related to the subset of *Ri* continues. *If* the frequency of the subset is higher than the specified threshold, the samples corresponding to the subset are added to the training set corresponding to Ri. In this way, the LP *method* is trained on the corresponding training set of Ri. At the same time, *EPS also* reduces the overfitting problem through ensemble learning.

2.1.3 PW Conversion Method

In 2008, Hullermeier et al. [21] proposed the Pairwise Comparative Ranking (RPC) algorithm and applied it to multi-label classification. This algorithm converts a multi-label problem with q labels into q(q - *1*)/2 binary classification sub-problems, each corresponding to a pair of labels. The sub-problem for the label pair (yi, *yj)* contains *all* instances related to label yi or yj in the original problem, *but* texts simultaneously related to both labels are excluded. Thus, instances related to label yi are positive *examples*, while the rest are regarded as negative examples. Therefore, this sub-problem can be solved using traditional single-label algorithms.

Obviously, the scale of the RPC algorithm is greatly affected by the number of labels. When the number of labels is large, the RPC algorithm is impractical for high complexity. Moreover, the RPC algorithm cannot distinguish the labels related to the instance under test. In other words, it lacks a threshold or dividing point to distinguish which part of the labels belongs to the instance.

Furnkranz et al. [22] proposed the Calibrated Label Ranking (CLR) algorithm to solve the aforementioned threshold point problem. In the CLR algorithm, a calibrated label y0 is added as the boundary between relevant and irrelevant labels. Compared with the RPC algorithm, only one *sub-problem* (yj, y0) needs to be added for each label yj, where the data *covers* all instances related to label *yj* (considered as irrelevant to *y0*) and *instances* not related to label yj (considered as relevant to y0*)*.

2.2 Algorithm adaptation method

Algorithm adaptation is the expansion of existing single-label algorithms to easily apply them to multi-label text data. In this section, several representative and widely used algorithm adaptation methods are introduced.

2.2.1 Nearest Neighbor

ML-kNN[23] is the first multi-label algorithm to use the nearest neighbor. Its basic idea is to calculate the occurrence of labels in the k nearest neighbors, and then calculate the probability of each label in different occurrence times. The prediction result is given based on the principle of maximum *a* posteriori. To estimate the corresponding probability, *ML-kNN* must perform a large number of calculations and distance comparisons, and the time complexity is relatively high. If there is noise in the training set, the effect of the *ML-kNN* algorithm is prone to be affected, and *ML-kNN* does not consider label correlation. To solve this problem,

LPLC[24] utilizes the correlation within a pair of labels within the nearest neighbor range. The probability estimation of LPLC is very similar to that of *ML-kNN*. The key difference lies in that LPLC is committed to finding the relevant label set for the predicted label. LPLC assumes that strong correlations only exist among the labels related to the training instance. For a training *set* with n instances and q labels, an n × q matrix M needs to be defined to *record* the ***relevant*** labels for *each* instance. Then, the probability of each label is calculated based on M**.**

2.2.2 Decision Tree

Clare et al. [25] proposed a decision tree-based multi-label algorithm ML-C4.5. It builds the decision tree from top to bottom, and the root of the tree includes all the training samples. For the instances in the non-leaf nodes, each feature is investigated one by one to find the appropriate split point. This split point is used to split the instances of this node, thereby obtaining the maximum information gain.

Hierarchical multi-label classification learning is implemented based on the Prediction Clustering Tree (PCT) [26]. Similar to other decision trees, PCT also divides the current cluster into smaller clusters top-down based on the principle of minimizing the within-cluster variance to the greatest extent. For the instances in the current cluster, the variance degree *S* is defined as the sum of the squares of the distances between the label vector (c_i) and the average label vector (c☒).

2.2.3 Neural Network

Bp-MLL[27] is the first algorithm that converts traditional neural networks into multi-label classification. It constructs a simple three-layer network, with *d* input units in the input layer, each corresponding to a feature of the training set; the hidden layer contains *M* units; the output layer has *q* units, each corresponding to a label. In Bp-MLL, the global loss function is involved in differentiating the relevant labels and irrelevant labels of instances, and guides the learning system to output relatively large values for relevant labels and relatively small values for irrelevant labels. Compared with the traditional loss evaluation method that directly compares the predicted values and actual values of each label in the output layer, Bp-MLL considers the relationships between different labels and achieves better results. CA2E is a multi-label classification algorithm based on the Deep Neural Network (DNN) proposed. The objective function of the CA2E algorithm can be divided into two parts. The first part uses the DNN model to solve the objective function and obtain the embedding features and the label space. The second part aims to restore the label space in the output of the entire model, where a method similar to Bp-MLL is used to solve this problem.

2.2.4 Support Vector Machine

Elisseeff proposed the Ranking-SVM algorithm [28]. Firstly, the Ranking-SVM algorithm transforms the high-efficiency single-label method, the Support Vector Machine (SVM), into a method directly applicable to multi-label classification. Ranking-SVM first defines a linear classifier for each label $\{h_j(X) = \langle \omega_j, x \rangle + b_j = \omega_j^T + b_j | 1 \leqslant j \leqslant q\}$, and then maximizes the distance between all relevant and irrelevant label pairs.

2.3 Deep learning methods

Unlike traditional machine learning methods, deep learning methods are more complex, but they have greatly promoted the development of multi-label text classification. In this section, deep learning models are mainly classified according to their structures into those based on convolutional neural networks (CNN) and those based on recurrent neural networks. Recurrent neural network (RNN), attention-based, Transformer-based, graph neural network (GNN)-based, and hybrid structures.

2.3.1 Based on CNN

CNN consists of convolutional layers, pooling layers and fully connected layers. The typical CNN structure is shown in Figure 4.

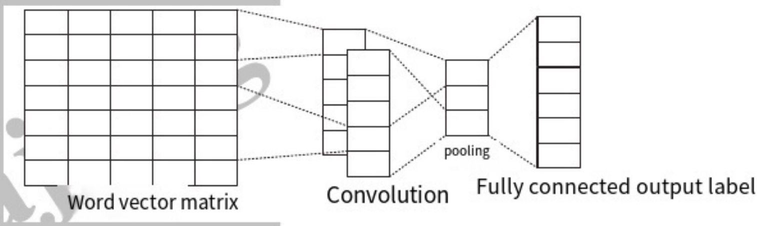

Figure 4 CNN Structure

Fig.4 CNN structure

CNN performs well when detecting local and position-invariant patterns is important. In 2014, Kim et al. [29] proposed the TextCNN model, training CNN on pre-trained word vectors for sentence-level classification tasks. A simple CNN with a small number of hyperparameter tunings and static vectors was tested, and task-specific vectors were learned through fine-tuning. However, due to the need to utilize a fixed window in CNN, it is not possible to model long text information. Liu et al. [30] improved the structure of TextCNN and proposed XML-CNN. The difference between this model and TextCNN is that it learns a large number of feature representations by passing the document through various convolutional filters, adopts dynamic max pooling to capture more fine-grained features from different regions of the document, and uses binary cross entropy loss on the output, inserting an additional hidden bottleneck layer between the pooling and output layers to learn a compact document representation. Shimura et al. [31] proposed a fine-tuning technique using CNN, a hierarchical convolutional neural network structure (HFT-CNN), which effectively utilizes the upper-layer data to contribute to the lower-layer classification. Yang et al. [32] proposed a dual hyperspectral CNN (HSCNN) to handle imbalanced data, which is a hybrid-Siamese convolutional neural network (HSCNN), that is, a multi-task structure based on a single network and a Siamese network, using a general network for the head part classification and a few-shot technique for the tail part classification.

Although multi-label text classification based on CNN does not require a large amount of computing cost, due to the drawback that CNN needs to utilize a fixed window and the pooling operation causes the loss of semantics, when the context text is too long, the CNN-based model is not conducive to capturing the label relationship between the context, and therefore is not conducive to multi-label text classification.

2.3.2 Based on RNN

RNN is a kind of network used to capture information from time series data. The structure of RNN is shown in Figure 5, where x_t is the input at time *t*; *St* is the hidden layer unit *at time* t; yt is the *output* at time *t*; O is the weight ***matrix***; U is the input transformation ***matrix***; V is the output transformation matrix, which is shared with U at different time ***points*** of the sequence and can be regarded as learning the fixed state transition matrix in the sequence.

The model based on RNN regards the text as a sequence of words, aiming to capture the word dependencies and text structure for TC. For multi-label texts

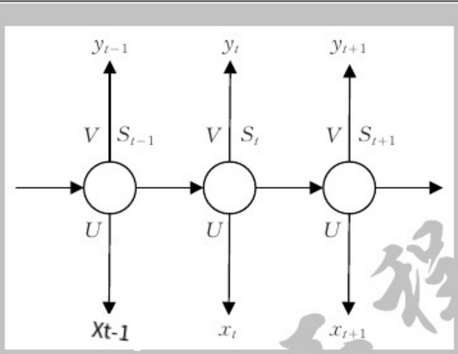

Figure 5 RNN Structure

Fig.5 RNN structure

Classification: Methods based on CNN usually cannot capture the complex correlations among multiple labels, resulting in a low recall rate. To address this issue, RNN is widely applied to explore the correlations of labels for multi-label text classification [33-34]. It is a recurrent neural network that predicts labels one by one. Nam et al. [33] used RNN instead of a classifier chain, which is a sequence-to-sequence prediction algorithm and has recently been successfully applied to sequence prediction tasks in many fields. The key advantage of this method lies in that it allows for the prediction to focus only on positive labels, and the set of them is much smaller than the complete set of possible labels. Moreover, parameter sharing among all classifiers can better utilize the information from previous decisions. In subsequent studies, Yang et al. [35] further incorporated deep reinforcement learning into the Seq2Seq model to reduce the impact of label arrangement on performance. Lin et al. [36] proposed a multi-level extended convolution based on the Seq2Seq model. The dilated convolution effectively reduces the dimension and supports the exponential expansion of the receptive field without losing local information.

However, these models ignore the correlation between labels or do not consider the key information of the text content. Therefore, they cannot obtain good prediction results. Yang et al. [37] regarded the multi-label text classification problem as sequence generation, considered the correlation between labels, transformed the decoding part, and automatically obtained the key information of the text to improve the effectiveness of label prediction. This improvement improved the model effect to a certain extent, but still needs to be enhanced.

2.3.3 Based on Attention

The Attention mechanism was proposed by the Bengio team in 2014 and applied to natural language processing. Yang et al. [38] incorporated word-level and sentence-level attention into the model to handle large-scale text classification. Hong et al. [39] proposed attention pooling, adding a 2D max pooling operation to maintain more important semantic information and reduce the impact of noise. In addition, to better highlight the important text information in different contexts, a multi-level attention mechanism was proposed. Li et al. [40] adopted two levels of attention to improve the performance of text classification. The first level of attention aims to capture both local and long-distance relevant features simultaneously, and the second level of attention pays attention to the generated features through a bidirectional recurrent attention network. You et al. [41] proposed Attention XML to capture the most relevant parts of the text to each label. The emergence of Attention XML surpasses all traditional machine learning methods and proves the superiority of the original text compared to sparse features. Unlike using a simple fully connected layer for label scoring in XML-CNN, Attention XML adopts a probabilistic label tree (PLT) that can handle millions of labels. By initializing the weights of the current layer model through its upper-layer model, it can help the model converge quickly. However, This still makes Attention XML very slow in terms of prediction and obtaining data from large-scale holistic model sizes. Du et al. [42] first used convolutional operations to capture attention signals, each of which represents the local information of a word in its context; then these attention signals were predicted. Xiao et al. [43] were the first to attempt to propose a history-based attention mechanism in the Seq2Seq model to enhance the predictive ability of labels in multi-label text classification. The history-based attention mechanism considers historical context information to avoid falling into label traps and also considers historical label information to alleviate the problem of error propagation. Liu et al. [44] proposed the LAA_SD method, which selects discriminative features from redundant content, considers semantic labels, and establishes the relationship between labels and text based on the attention mechanism. This method combines the enhanced text feature representation with the semantic dependence of labels to perform multi-label learning of text. Song et al. [45] proposed a label prompt multi-label text classification model (LP-MTC), which designed a template for multi-label text classification, integrated labels into the input of the pre-trained language model, and jointly optimized through the masked language model (MLM). Through this way, the correlation between labels and the semantic information between labels and text are captured under the self-attention mechanism, thereby effectively improving the model performance.

2.3.4 Based on Transformer

One of the computational bottlenecks encountered by RNN is the sequential processing of text. Although the sequentiality of CNN is not as good as that of RNN, the computational cost of capturing the relationships between words in a sentence also increases with the length of the sentence, similar to RNN. Transformer overcomes these problems. Its structure retains only Attention, as shown in Figure 6, and does not need to be combined with RNN or CNN. Self-attention is applied to calculate the "attention score " of each word in a sentence or document in parallel to simulate the influence of each word on the others. Due to this feature, Transformer allows for more parallelization than CNN and RNN, which makes it possible to efficiently train very large models on large amounts of data on GPUs.

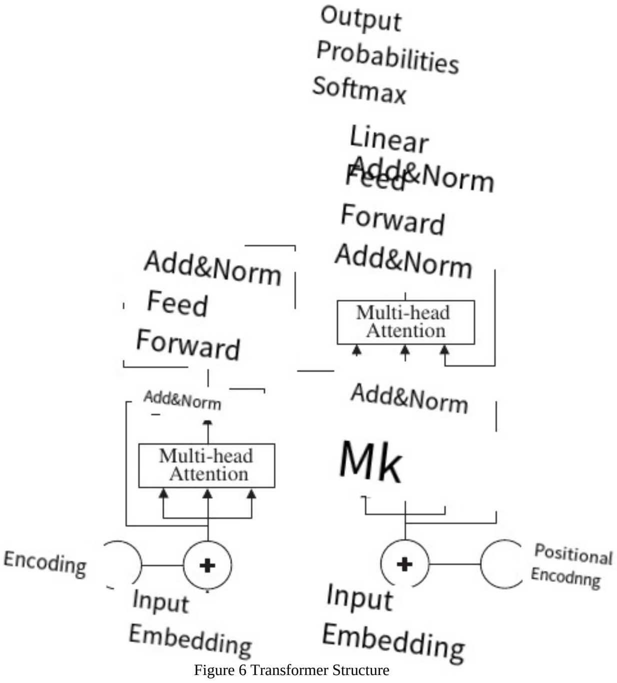

Figure 6 Transformer Structure

Fig.6 Transformer structure

Chang et al. [46] proposed the X-Transformer model, which only uses the deep learning model to match the label clusters of the given original text and sorts these labels through the high-dimensional linear classification of the sparse features and text representations with the deep learning model. However, due to the high computational complexity of the Transformer model, simply fine-tuning the Transformer model as the label cluster matcher cannot fully utilize the functions of the Transformer model. Although the X-Transformer can achieve higher accuracy than AttentionXML at the cost of higher computational complexity and model size, AttentionXML can achieve better accuracy under the same computational complexity of the X-Transformer because it uses more integrated models. Gong et al. [47] proposed the HG-transformer, which models the input text as a graph structure; then adopts a multi-layer transformation structure at the word, sentence, and graph levels to fully capture the text features; finally, generates *t* label representations using the hierarchical relationships between labels. Jiang et al. [48] proposed LightXML, which adopts end-to-end training and dynamic negative label sampling. In LightXML, the generative cooperative network is used to invoke and sort labels, where the label invocation part generates negative and positive labels, and the label sorting part distinguishes positive labels from these labels. Through these networks, during the training period of the label ranking part, negative labels are dynamically sampled by feeding the same text representation to improve the effect of the model. Ye et al. [49] proposed a novel neural network-based multi-label document classification method, in which the heterogeneous graph Transformer is used to construct and learn two heterogeneous graphs. One is the metadata heterogeneous graph, which models various types of metadata and their topological relationships; the other is the label heterogeneous graph, which is constructed based on the hierarchical structure of labels and their statistical dependencies. Chen et al. [50] proposed the LitMC-BERT model, which uses a shared Transformer backbone and also captures label-specific features and the correlations between label pairs for multi-label classification. Zhang et al. [51] believe that the global feature vector may not be sufficient to represent the different granularity levels of semantics in the document, so they combine the local and global features generated by the Transformer model to improve the predictive ability of the classifier.

2.3.5 Based on GNN

Although natural language texts exhibit a continuous sequence, they also contain internal graph structures, such as grammar and semantic analysis trees, which define the grammatical and semantic relationships between words in a sentence. One of the earliest graph-based models developed for NLP is TextRank. The authors proposed representing natural language texts as a graph (V, E), where V represents a set of nodes and E represents a set of edges between nodes. Depending on the specific application, nodes can represent various types of text units, such as words, collocations, entire sentences, etc. Similarly, edges can represent different types of relationships between any nodes, such as lexical or semantic relationships, context overlap relationships, etc.

Among various types of GNNs, Graph Convolutional Networks (GCNs) [52] and their variants are the most popular ones because they are effective and conveniently combined with other neural networks, and have achieved state-of-the-art results in many applications. GCNs extend the convolutional operation from grid data to graph data. The main idea is to generate the representation of a node by aggregating its own features and adjacent features. GCNs stack multiple graph convolutional layers to extract advanced node representations. The Text Level GCN model proposed by Liu et al. [53] for each

The input texts are constructed independently but have a globally parameterized graph instead of establishing a huge single graph for the entire training and test corpora, and the graph is constructed through a sliding window, in which the number of n-grams can be set to extract more local features and reduce a large amount of computing resources. This also enables the graph neural network to induce patterns from the existing data and apply them to new tasks. The Graph Attention Network (GAT) proposed by Velikovi et al. [54] is a variant of GCN that operates on graph-structured data and uses a hidden self-attention layer to address the shortcomings of previous graph convolution-based methods. Through layer stacking, nodes can participate in the features of their neighboring nodes, supporting (implicitly) the specification of different weights for different nodes in a neighborhood without the need for any expensive matrix operations (such as inversion) or reliance on prior knowledge of the graph structure. Yao et al. [3] proposed the TextGCN model, which uses GCN to establish a text and word-based heterogeneous graph for the entire dataset, which can be used to obtain the co-occurrence information of the global graph to enable GCN to perform semi-supervised classification of texts. Pal et al. [55] proposed a model based on the Graph Attention Network to capture the attention-dependent structure between labels. The Graph Attention Network uses feature matrices and correlation matrices to capture and explore the key dependencies between labels and generate classifiers for the task. The generated classifiers are applied to the sentence feature vectors obtained from the text feature extraction network (BiLSTM) to achieve end-to-end training. Ding et al. [56] proposed a principled model - the Hypergraph Attention Network (HyperGAT), which can achieve stronger expressive power with less computational consumption in text representation learning. Zong et al. [57] proposed GNN-XML, which is an extensible graph neural network framework. By mining their co-occurrence patterns to utilize label correlations and constructing a label graph based on the correlation matrix; then, by using a low-pass graph filter for graph convolution to jointly model the label dependencies and label features, thereby performing attribute graph clustering and inducing semantic label clustering. Zheng et al. [58] proposed a Label Division Gated Graph Neural Network (LD-GGNN), which can better distinguish labels at the same level, achieve adaptive interaction between texts and labels, optimize the Gated Graph Neural Network (GGNN) to accurately capture the structural features of the label hierarchy, and deeply explore label dependencies, leveraging the stronger nonlinear characteristics of GGNN to address the over-smoothing problem.

2.3.6 Hybrid model

Many hybrid models have been developed and applied to multi-label text classification.

Zhou et al. [59] proposed a convolutional LSTM (C-LSTM) network. C-LSTM utilizes CNN to extract sequences represented by higher-level phrases *(n-grams)*, which are fed into the LSTM network to obtain sentence representations. Similarly, Zhang et al. [60] proposed a Dependency Sensitive CNN (DSCNN) for document modeling. DSCNN is a hierarchical model in which LSTM learns sentence vectors, which are fed into convolutional and max-pooling layers to generate document representations. Chen et al. [34] proposed a CNN-RNN model to accurately obtain global and local text semantic information and model high-order label correlations. The proposal of Transformer has had a huge impact on the field of natural language processing, but the Transformer model often requires a large number of model parameters and has a complex network structure, which has

Certain limitations. Liu et al. [61] proposed a multi-label text classification method of tALBERT-CNN, using the LDA topic model and ALBERT model to obtain the topic vector and semantic context vector of each word (document), and adopting a certain fusion mechanism to obtain the deep topic and semantic representation of the document. The multi-label features of the text were extracted through the TextCNN model, and the multi-label classifier was trained, reducing the model parameters. Yan et al. [62] This paper proposes an R-Transformer_BiLSTM model based on label embedding and attention mechanism for multi-label text classification. For the first time, the entity recognition model is introduced into text classification, using the R-Transformer model combined with partial speech embedding to obtain the global and local information of the text sequence; at the same time, BiLSTM + CRF is used to obtain the entity information of the text, and the self-attention mechanism is used to obtain the keywords of the entity information. Then, the bidirectional attention and label embedding are used to further generate the text representation and label representation. Finally, the classifier classifies the text based on the label representation and text representation.

2.3.7 Other research methods

Xiao et al. [63] proposed a Head-to-Tail Network (HTTN), transferring meta-knowledge from the data-rich head labels to the data-poor tail labels. Zhang et al. [64] introduced a novel method with multi-task learning to enhance label correlation feedback. Firstly, the Joint Embedding (JE) mechanism was utilized to obtain the representations of both the text and the labels simultaneously. In the MLTC task, The document tag cross-attention mechanism (CA) is used to generate more discriminative document representations. Additionally, two auxiliary tag co-occurrence prediction tasks are proposed to enhance tag correlation learning: (1) Paired tag co-occurrence prediction (PLCP) and (2) Conditional tag co-occurrence prediction (CLCP). Khataei et al. [65] proposed a spotted hyena optimizer - long short-term memory (SHO-LSTM) model for MLTC based on the LSTM network and SHO algorithm. In the LSTM network, words are embedded into the vector space, and the SHO algorithm is used to optimize the initial weights of the LSTM network. Adjusting the weight matrices in the LSTM is a major challenge. If the weights of the neurons are accurate, the output accuracy will be higher. Table 1 enumerates some deep learning models or methods.

3 Multi-label text classification dataset

The mainstream datasets in 13 multi-label text classification fields were summarized, covering Chinese and English, long texts, short texts, extremely multi-label and ordinary multi-label. They were ranked according to the average number of labels per sample, and the relevant data were presented in Table 2.

(1) IMDB: It contains 117,196 movie introductions (in English), with a total of 27 movie categories. Each movie introduction has one or more possible categories. The dataset provides a multi-label binary mask for each movie based on whether the movie belongs to a specific category.

Table 1 Deep Learning Models and Methods

Table 1 Deep learning models and methods

Category	Model / Method	Literature	Brief Introduction	year
Based on CNN	TextCNN	[29]	The classification is carried out by using the basic CNN structure. This model takes the sentence matrix composed of word vectors as the input of CNN and uses the CNN convolutional layer to extract features.	2014
	XML-CNN	[30]	The document is passed through various convolutional filters to learn a large number of feature representations. Dynamic max pooling is adopted to obtain from different parts of the document. The document is passed through various convolutional filters to learn a large number of feature representations. Dynamic max pooling is adopted to capture more fine-grained features from different regions of the document. Binary cross entropy loss on the output is utilized. An additional hidden bottleneck layer is inserted between the pooling and output layers to learn a compact document representation.	
	HFT-CNN	[31]	A fine-tuning technique using CNN, a hierarchical convolutional neural network structure (HFT-CNN), effectively utilizes the upper-layer data to contribute to the classification of the lower layer.	2018
	HSCNN	[32]	A hybrid - Siamese convolutional neural network, that is, a multi-task based on a single network and a Siamese network, adopts a general network for the head classification and a few-shot technique for the tail classification.	2020
Based on RNN	Deep reinforcement learning	[35]	Integrate deep reinforcement learning into the Seq2Seq model to reduce the impact of label permutation on performance.	2018
	Multi-level extended convolution	[36]	The multi-level extended convolution based on the Seq2Seq model, the dilated convolution effectively reduces the dimension, supports the exponential expansion of the receptive field without losing local information.	2018
	SGM	[37]	The multi-label text classification problem is regarded as sequence generation, considering the correlation between labels, the decoding part is transformed, and the key information of the text is automatically obtained to improve the effectiveness of label prediction.	2018
Based on Attention	Words, sentences Attention	[38]	Introduce word-level and sentence-level attention into the model to handle large-scale text classification	2016
	Multi-level Attention	[39]	The 2Dmax pooling operation is added to maintain more important semantic information and reduce the influence of noise. Besides, in order to better highlight the important text information in different contexts, a multi-level attention mechanism is proposed.	2018
	Two-level Attention	[40]	The first-level attention aims to capture both local and long-distance relevant features simultaneously, and the second-level attention pays attention to the generated features through a bidirectional recurrent attention network.	2018
	Attention XML	[41]	Using the attention mechanism to handle the original texts with different models and initializing the weights of the current layer model through its upper-layer model can help the model converge rapidly.	2019
	History-base Attention	[43]	The historical context information was considered to avoid falling into the label trap, and the historical label information was also taken into account to alleviate the problem of error propagation in 2021.	
	LAA_SD	[44]	Select discriminative features from redundant content, consider semantic labels, and establish the relationship between labels and text based on the attention mechanism.	2022
	LP-MTC	[45]	Integrate the labels into the input of the pre-trained language model and jointly optimize through the masked language model (MLM). In this way, the correlation between labels and the semantic information between labels and the text can be captured with the help of self-attention.	2022

Table 1 (Continued)

Category	Model / Method	Literature	Brief Introduction	year
Based on Transformer	X-Transformer	[46]	The model consists of three parts, including the Semantic Label Sequence Component (SLI), the Deep Neural Matching Component and the Overall Ranking Component.	2020
	HG-Transfomer	[47]	The model models the text as a structure, introduces a multi-head attention mechanism-based multi-layer Transformer structure in words and sentences to capture features, and utilizes the hierarchical relationship of labels to generate the representation of labels. To capture the features, the representation of the labels is generated by using the hierarchical relationship of the labels. The generative cooperative network invokes and sorts the labels, where the label invocation part generates negative and positive labels, and the labels	2020
	LightXML	[48]	The ranking part distinguishes the positive labels from these labels. Through these networks, during the training period of the label ranking part, negative labels are dynamically sampled by feeding the same text representations.	
	Heterogeneous graph	[49]	The heterogeneous graph Transformer is used to construct and learn two heterogeneous graphs. One is the metadata heterogeneous graph, which models various types of metadata and their topological relationships. The other is the label heterogeneous graph, which is constructed based on the hierarchical structure of labels and their statistical dependencies.	
	LitMC-BERT	[50]	Multi-label classification is carried out by using the shared Transformer backbone while capturing the label-specific features and the correlations between label pairs.	2022
	GLOCAL XML	[51]	Combining the local and global features generated by the Transformer model to improve the predictive ability of the classifier. Construct an independent graph with global parameter sharing for each input text instead of establishing a huge single graph for the entire training and test corpus, and construct the graph through the sliding window to extract more local features and reduce a large amount of computing resources.	2022
Based on GNN	Text Level GCN	[53]		2016
	GAT		Operating on graphic structure data, the hidden self-attention layer is utilized to address the shortcomings of previous methods based on graph convolution or their approximations [54]. Through layer stacking, nodes can participate in the characteristics of their neighbors, supporting the specification of different weights for different nodes in a neighborhood in 2017, without the need for any expensive matrix operations (such as inversion) or reliance on prior knowledge of the graph structure.	
	TextGCN	[55]	Using GCN to establish a text and word-based heterogeneous graph for the entire dataset can be used to obtain the co-occurrence information of the global graph, enabling GCN to perform semi-supervised classification of the text.	2020
	HyperGAT	[56]	It is possible to obtain stronger expressive power with less computational consumption in text representation learning.	2020
	GNN-XML	[57]	The label correlations are exploited by mining their co-occurrence patterns and a label graph is constructed based on the correlation matrix. Then, the label dependencies and label features are jointly modeled by performing graph convolution using a low-pass graph filter for attribute graph clustering, thereby inducing semantic label clustering in 2020.	
	LD-GGNN	[58]	Better distinguish labels of the same level, achieve adaptive interaction between the text and the labels, optimize the Graph Neural Network with Gated Mechanism (GGNN) to accurately capture the structural features of the label hierarchy and deeply explore the label dependence. Utilize the stronger nonlinear characteristics of GGNN in 2022 to solve the over-smoothing problem.	
Hybrid model	C-LSTM	[59]	The CNN is utilized to extract the sequence represented by higher-level phrases *(n-grams)*, and this sequence is fed into the LSTM network to obtain the sentence representation.	2015
	DSCNN	[60]	A hierarchical model, in which LSTM learns sentence vectors, and these sentence vectors are fed into convolution and max pooling layers to generate document representations	2016
	CNN-RNN	[34]	This model integrates CNN and RNN to accurately obtain global and local text semantic information and model high-order label correlations.	2017
	tALBERT-CNN	[61]	It uses the LDA topic model and the ALBERT model to obtain the topic vector and semantic context vector of each word (document), and adopts a certain fusion mechanism to obtain the deep topic and semantic representation of the document. The multi-label characteristics of the text are extracted through the TextCNN model, and the multi-label classifier is trained.	
	R-Transformer_ BiLSTM	[62]	The entity recognition model is introduced into text classification for the first time. The R-Transformer model is used in combination with some speech embeddings to obtain the global and local information of the text sequence. At the same time, BiLSTM + CRF is used to obtain the entity information of the text, and the self-attention mechanism is used to obtain the keywords of the entity information. Then, bidirectional attention and label embedding are used to further generate the text representation and label representation. Finally, the classifier conducts text classification based on the label representation and text representation.	2022
Other research methods	HTTN	[63]	A head-to-tail network (HTTN) transfers meta-knowledge from the data-rich head labels to the data-poor tail labels.	2021
	Multi-task learning	[64]	The joint embedding (JE) mechanism is utilized to obtain the representations of both the text and the labels simultaneously. The document label cross attention (CA) mechanism is adopted to generate more discriminative document representations. Two auxiliary label co-occurrence prediction tasks are proposed to enhance the learning of label correlations: label co-occurrence prediction (PLCP) and (2) conditional label co-occurrence prediction (CLCP).	(1) Formed in 2021
	SHO-LSTM	[65]	The Skip-Gram method is used to embed words into the vector space. The SHO algorithm is adopted to optimize the initial weights of the LSTM network and adjust the weight matrices in the LSTM.	2022

(2) Ren-CECps1. 0: This dataset is a Chinese sentiment corpus, containing sentences from 37,678 Chinese blogs and 11 sentiment labels.

(3) Reuters-21578: The dataset contains 22 documents, totaling 10, 788 news articles from Reuters, with a total of 90 labels.

(4) AAPD: This dataset collects the abstracts of 55, 840 papers and the corresponding disciplinary categories. An academic paper belongs to one or more disciplines.

(5) RCV1: There are a total of 804,414 news reports, involving 103 categories. No. Each report may contain one or more categories. On average, each news report contains 3.2 category labels.

(6) RCV1-V2: This dataset consists of a total of 804,414 news articles, and each news story is assigned multiple topics, with a total of 103 topics.

(7) ToutiaoNews: This dataset is a Chinese dataset for statistics of news on Toutiao.

(8) Wiki-500K: The data also contains from Wikipedia, but is different from Wikil0-

Table 2 Multi-label Text Classification Dataset

Table 2 Multi-label text classification dataset

Dataset	The total number of samples	Number of labels	The average number of words in the sample	The average number of labels of the samples
IMDB	117 196	27	98.40	2.20
Ren-CECps1.0	37,678	11	24.71	2.40
Reuters-21578	10,788	90	—	—
AAPD	55,840	54	163.42	2.40
RCV1	804 414	103	268.90	3.20
RCV1-V2	804 414	103	123.94	3.20
ToutiaoNews	28 938	1070	—	—
Wiki-500K	2,549,302,501,008	501 008	808.66	4.75
AmazonCat-13K	1 493 021	13,330	448.57	5.00
EUR-Lex	19 314	3,956	1239.49	5.30
Amazon-670K	643 474	670,091	244.27	5.50
Wiki10-31K	20,762	30 938	2 484.30	18.64
Amazon-3M	2,460,406	2,812,281	104.13	36.00

Compared with the 31K dataset, the sample quantity is larger and the label quantity is also larger.

(9) AmazonCat-13K: This dataset is from Amazon and contains data such as user reviews and product information.

(10) EUR-Lex: The dataset is organized from documents of various EU laws, treaties, etc., and contains 15,449 training documents and 3,865 test documents. The entire dataset has a total of 3,956 labels.

(11) Amazon-670K: This dataset consists of reviews of Amazon products, with 643,474 sample data.

(12) Wiki10-31K: The dataset contains 20,762 articles from Wikipedia, but the number of labels reaches 30,938.

(13) Amazon-3M: This dataset consists of the product information, links and reviews of Amazon products, with the number of labels reaching 2,812,281.

4 Evaluation Metrics and Model Analysis for Multi-label Text Classification

4.1 Evaluation Index

The evaluation of multi-label text classification (MLTC) models is different from that of single-label classification models. Therefore, based on the literature [66], several multi-label text classification evaluation metrics have been proposed, which are divided into two main methods: instance-based metrics and label-based metrics. The first method is to test each instance and then calculate the average over all the tested instances. The second method is to calculate for each label and then take the average over all the labels.

4.1.1 Instance-based metrics

The following introduces the most common instance-based metrics used to evaluate multi-label text classification models. Hypothesis: m refers to the total number of instances in the dataset, i represents an instance in the dataset (where $1 \leqslant i \leqslant m$), n is the total number of labels, *Zi* *and* Yi *refer* to the predicted and actual labels *respectively*.

(1) Hamming loss: Calculate the average number of errors found in the instance label pairs and average over all instances. The expression of this metric is shown in Formula (1) as follows:

$$Hamming\ loss = \frac{1}{m}\sum_{i=1}^{m}\frac{1}{n}\left|Z_i \Delta Y_i\right| \tag{1}$$

Among them, Δ defines the symmetric difference between the predicted label and the actual label, and the factor $1/n$ is used to obtain the normalized value within [0, 1].

(2) Multi-label accuracy (ML-accuracy): Calculate the ratio of correctly predicted labels to the total number of labels. The calculation is shown in Formula (2) as follows:

$$ML\text{-}accuracy = \frac{1}{m}\sum_{i=1}^{m}\frac{\left|Z_i \cap Y_i\right|}{\left|Z_i \cup Y_i\right|} \tag{2}$$

(3) Subset-accuracy: Also known as the exact match ratio or classification accuracy. This is a very strict metric used to measure the rate of predicted labels that exactly match their corresponding actual label sets. The calculation is shown in Formula (3) as follows:

$$Subset\ accuracy = \frac{1}{m}\sum_{i=1}^{m} I(Z_i = Y_i) \tag{3}$$

(4) Precision: This indicator provides the ratio of correctly classified labels to predicted labels, and the calculation is shown in Formula (4) as follows:

$$Precision = \frac{1}{m}\sum_{i=1}^{m}\frac{\left|Z_i \cap Y_i\right|}{\left|Z_i\right|} \tag{4}$$

(5) Recall rate (Recall): Calculate the rate of correct predicted labels for the actual labels. The calculation is shown in Formula (5) as follows:

$$Recall = \frac{1}{m}\sum_{i=1}^{m}\frac{\left|Z_i \cap Y_i\right|}{\left|Y_i\right|} \tag{5}$$

(6) F-measure: The harmonic mean of precision and recall rate, calculated as shown in Formula (6):

$$F\text{-}measure = \frac{1}{m}\sum_{i=1}^{m}\frac{2\left|Z_i \cap Y_i\right|}{\left|Z_i\right| + \left|Y_i\right|} \tag{6}$$

Except for the Hamming loss metric, all the example-based metrics described in this subsection indicate that the metric with a higher value has better performance, and the lower the value of the Hamming loss, the better the performance.

4.1.2 Label-based metrics

Based on the two methods of calculating the average value, calculate the binary evaluation indicators (such as recall rate, precision, and *F-measure*) for all labels; macro or micro averaging methods. These indicators are widely used to measure the average values of recall rate, precision, and *F-measure*. Let *B* be a binary evaluation metric used to calculate these indicators, which is calculated based on the *quantities* of true positives *(tp)*, *false positives (fp)*, true negatives (tn), *and false* negatives (fn). As shown in Formula (7),

(8)

It explains the expressions of the *macroscopic* average and microscopic average indicators of *B*(*tp*, fp, *tn*, *fn*).

$$B_{\mathrm{macro}} = \frac{1}{n}\sum_{i=1}^{n} B(t_{p_i}, f_{p_i}, t_{n_i}, f_{n_i}) \tag{7}$$

$$B_{\mathrm{micro}} = B\left(\sum_{i=1}^{n} t_{p_i}, \sum_{i=1}^{n} f_{p_i}, \sum_{i=1}^{n} t_{n_i}, \sum_{i=1}^{n} f_{n_i}\right) \tag{8}$$

4.2 Model Analysis

Table 3 presents the result analysis of the relevant multi-label text classification methods on some datasets.

It can be seen from the model results that with the development of deep learning, the F-measure values of models based on deep learning methods on different datasets have significantly improved. On the APPD dataset, the F-measure value has increased from 0.6412 of the BR model to 0.7458 of the LP-MTC model, with an increase of

Table 3 Analysis of Model Method Results

Table 3 Analysis of model method results

Category	Model / Method	Literature	Dataset AAPD	Precision	Recall	F-measure	year
Traditional machine learning methods	BR	[18]	AAPD	0.66 41	0.6483	0.6461	2004
			RCV1-V2	0.9042	0.8161	0.8561	
	CC	[14]	AAPD	0.6574	0.6514	0.6542	2011
			RCV1-V2	0.8871	0.8281	0.8573	
Based on CNN	TextCNN	[29]	IMDB	0.7202	0.6152	0.6714	2014
			AAPD	0.6312	0.550 6	0.5732	
	XML-CNN	[30]	IMDB	0.728 7<br>0.728 7 0.651 8 0.887 0	0.6475	0.6905	2017
			AAPD		0.5884	0.6235	
Based on RNN	SGM	[37]	RCV1-V2		0.8501	0.869 1	2018
			AAPD	0.7461	0.659 2	0.6993	
	Multi-level extended convolution	[36]	RCV1-V2	0.891 0	0.8733	0.8821	2018
			Ren-CECps	0.593 1	0.5851	0.5902	
Based on Attention	Attention XML	[41]	AAPD	0.7571	0.6857	0.7155	2019
			RCV1-V2	0.8987	0.8665	0.881 7	
	LAA_SD	[44]	APPD	0.7641	0.693 2	0.7211	2022
Based on Transformer	X-Transformer	[46]	EURLex-4K	0.751 2	0.684 0	0.7179	2020
			Wiki10-31K	0.7871	0.714 2	0.7504	
			AmazonCat-13K	0.8385	0.7701	0.8041	
	LightXML	[48]	EURLex-4K	0.7589	0.6911	0.7251	2021
			Wiki10-31K	0.789 6	0.713 2	0.7514	
			AmazonCat-13K	0.8402	0.7824	0.8116	
	GLOCAL XML	[51]	EURLex-4K	0.789 0	0.7185	0.7538	2022
			Wiki10-31K	0.8095	0.7321	0.7710	
			AmazonCat-13K	0.839 7	0.7684	0.7623	
Based on GNN	GNN-XML	[57]	EURLex-4K	0.7614	0..691 2	0.7263	2020
			Wiki10-31K	0.8113	0.7485	0.7801	
	LD-GGNN	[58]	RCV1-V2	0.8924	0.8864	0.8894	2022
Hybrid model	CNN-RNN	[34]	AAPD	0.7181	0.6187	0.66 47	2017
			RCV1-V2	0.8898	0.8258	0.8564	
	tALBERT-CNN	[61]	IMDB	0.8431	0.7585	0.8023	2021
			AAPD	0.7519	0.6695	0.7317	
	R-Transformer_BiLSTM	[62]	AAPD	0.7621	0.6893	0.7181	2022
			RCV1-V2	0.9101	0.8904	0.893 2	
Other research models	SHO-LSTM	[65]	RCV1-V2	0.8696	0.8617	0.8803	2022
			Reuter-21578	0.6434	0.639 4	0.6414	
			Bookmarks	0.422 4	0.425 1	0.4233	

The effect is very obvious. On the RCV1-V2 dataset, the F-measure has increased from 0.8518 of the BR model to 0.8932 of the R-Transformer_BiLSTM model. On the EURLex-4K dataset, the effect of the model has increased from 0.7179 to 0.7538. On other datasets, with the development of deep learning, the models have all improved significantly. The performance of the R-Transformer_BiLSTM model on the RCV1-V2 dataset is completely superior to that on the AAPD dataset, indicating that this model still has good performance in text classification of large-scale data labels.

Traditional machine learning methods, such as BR and CC, etc., do not consider the correlation of labels, and the current label may be irrelevant to the labels in the previous part of the sequence. Thus, the use of the input of previous labels introduces noise, leading to a decrease in the performance of the classifier and a poor performance on some datasets.

Deep learning network models based on CNN, such as TextCNN and XML-CNN, etc., due to the drawbacks of the fixed window of CNN and the loss of semantics caused by its pooling operation, have affected the performance of their classifiers. Although the XML-CNN model has made certain improvements to this drawback to a certain extent, due to the fundamentally simple structure of CNN, its performance on different datasets is average.

The deep learning network models based on RNN, such as SGM, etc., have further improved the model and enhanced the performance of the classifier. However, when predicting labels, for sequence-based models, since the subsequent label often depends on the previous one, the influence of the wrong label of the previous one is often cumulative, resulting in the degradation of the classifier's performance. Although the models based on RNN have considered the correlation of labels, the improvement of the model's effect is not obvious.

Deep learning network models based on Attention, such as Attention

Such as XML and LAA_SD, etc., they apply dynamic max pooling to learn text representations and use multi-level attention to capture features. Attention XML uses a bidirectional long short-term memory (BiLSTM) network to extract embeddings from the original text input, which has a significant effect in improving the performance of the model. However, due to the insufficient consideration of local or global label correlations, it affects the model performance to a certain extent.

The deep learning network models based on Transformer, such as X-Transformer, etc., have overcome the problem of sequential processing of text and reduced the computational cost of capturing the relationships between words in a sentence, thereby improving the performance of the model. However, in actual application scenarios, the Transformer model often requires a large number of model parameters and has a complex network structure, which still affects the model performance to a certain extent.

Deep learning network models based on Graph Neural Networks, such as GNN-XML and LD-GGNN, etc., mine the internal graph structure of the text, extend from network data to graph data, extract more local features, reduce a large amount of computing resources, continuously explore the correlations between labels, improve the performance of the model, and perform significantly on different datasets.

The hybrid deep learning network models, such as the tALBERT-CNN and R-Transformer_BiLSTM models, utilize the attention mechanism to consider contextual information and the correlation between labels, extracting the key information of the text for multi-label text classification. Their classifier effects are remarkable, but there is still room for improvement in the classification results.

In recent years, the classification effects of other research models on different datasets have improved significantly. However, when dealing with large-scale labeled datasets and hierarchical multi-label datasets, the classification effects are not obvious and need to be further enhanced in future research.

5 Research Direction of Multi-label Text Classification

5.1 Label Relevance

5.1.1 Types of label relevance

In multi-label problems, labels are not independent but have some correlations. The use of label correlations is conducive to learning more effective and robust classification models. In multi-label text classification problems, some labels have very few positive samples. In such cases, the use of label correlations is extremely important. Making full use of label correlations has become one of the main research directions in multi-label classification at present, and it is an important component of many algorithms [67-68]. The existing label correlation usage strategies can be divided into three types [69]: first-order, second-order, and high-order.

(1) First-order algorithm

First-order algorithms such as BR, ML-C4.5, and *ML-kNN* [23]. BR builds binary classification methods for each label with the aim of learning the corresponding classifier hi: $X \rightarrow \{0, 1\}$. During the learning process, the input is the original feature space, and the output is the value of the label yi. Therefore, different labels have the same input but different outputs. ML-C4.5 divides the training data set layer by layer into several smaller subsets through decision trees, with the root covering all the training data. For non-leaf nodes, indicators such as information entropy and Gini index are applied to further divide the non-leaf nodes into child nodes, so that the "purity" of the data of the child nodes is higher than that of the parent node. *ML-kNN* is an inert learning method. When making predictions, the *ML-kNN* model decides whether a test instance is related to a certain label based on the principle of maximizing the posterior probability according to the distribution of each label among the nearest neighbors of the predicted data in the training data. However, these first-order algorithms completely ignore the label correlation.

(2) Second-order algorithm

Second-order algorithms such as Rank-SVM [28], CLR [22], MLPP [70], CPNL [71], PCT [72], and GBRAML [73] take into account the label correlations. Rank-SVM defines an optimization objective of maximizing the distance in the correlated and uncorrelated label pairs and uses SVM techniques to solve the multi-label classification problem. The CLR algorithm extends common learning to multi-label text classification through pairwise comparison methods, introducing an artificially calibrated label to separate the relevant labels from the irrelevant labels in each instance. MLPP trains the classifier for each pair of labels and combines the prediction results of various classifiers through voting to determine the sequence of label correlations. CPNL utilizes the positive and negative correlations of labels and extends the BR algorithm. PCT proposes a pairwise comparison transformation method of labels based on the definition of ranking loss, transforming each original multi-label sample into multiple samples with the same feature vector but different label vectors. GBRAML is a multi-label ranking activity model based on the particle batch processing mode. From a bottom-up perspective, three granulation operators are constructed in sequence to form three particle structures. In the lower-level granulation operators, auxiliary labels are introduced to enhance the informativeness and representativeness of each label. Table 4 enumerates the first-order and second-order algorithms for label correlation research.

(3) High-order algorithms

High-order algorithms such as RAkEL [19], CC [18], BNCC [74], and MLMF [75] consider the correlations among several or all labels. As an ensemble learning method, the *RAkEL* algorithm utilizes the relationships among multiple labels by considering a small random subset of labels for any label, and the labels at the beginning of the sequence will be added as new features to the original features. BNCC models the label correlations using Bayesian networks, describes the dependency relationships between labels with conditional entropy, with nodes as labels and the weights of edges as the dependency relationships, and introduces a heuristic algorithm to optimize the BN structure. By performing topological sorting on the optimized BN nodes, the label sequence for constructing the CC model is obtained. MLMF designs an effective multi-label classifier, automatically learns high-order asymmetric label correlations, reduces the dimension of the feature space, and handles both complete and missing labels. From the perspective of relationship extraction or usage, high-order algorithms can be classified as global relationship algorithms, local relationship algorithms, and global-local combination relationship algorithms.

① Global relationship algorithm

The global relation algorithm assumes that the label correlations are global. In other words, the correlations between labels exist in all the training data. Global relation algorithms include CC, MLLS [76], ML-LPC [77], CLSF [78], and A-GCN [79]. CC places all labels in a random sequence. The output of the binary classifier of the previous label is added as a new feature to the original feature space of the label. MLLS is a general framework for extracting the shared structure in multi-label classification. In this framework, a common subspace is shared by multiple labels, thereby clarifying their intrinsic relations. ML-LPC learns the relations between labels.

Table 4 First-order Algorithm and Second-order Algorithm

Table 4 First-order algorithms and second-order algorithms

Category	Literature	Frame	Brief Introduction	year
First-order algorithm	[18]	BR	Binary classification methods are constructed for each label, with the aim of learning the corresponding classifier *hi*: X → {0, 1}. During the learning process, the input is the original feature space, while the output is the value of the label yi. Therefore, different labels have the same input but different outputs.	2004
	[25]	ML-C4.5	The training data set is divided layer by layer into several smaller subsets through the decision tree, and the root node covers all the training data. For non-leaf nodes, indicators such as information entropy and Gini index are applied to further divide the non-leaf nodes into child nodes, so that the "purity" of the data of the child nodes is higher than that of the parent node.	2007
	[23]	ML-*k*NN	Based on the distribution of labels among the nearest neighbors of the training data in the predicted data, the principle of maximizing the posterior probability is adopted to determine whether the test instance is related to a certain label.	2016
Second-order algorithm	[28]		Rank-SVM defines the optimization objective of maximizing the distance in the relevant and irrelevant label pairs, and uses SVM technology to solve the multi-label classification problem.	2003
	[22]	CLR	Common learning is extended to the multi-label scenario through the pairwise comparison method, introducing an artificially calibrated label. In each example, the relevant label is separated from the irrelevant labels.	2008
	[70]	MLPP	The classifier is trained through each pair of labels, and the sequence of label correlations is determined by combining the prediction results of various classifiers through voting.	2008
	[72]	PCT	According to the definition of ranking loss, a pairwise comparison transformation method of labels is proposed, which converts each original multi-label sample into multiple samples with the same feature vector but different label vectors.	2017
	[71]	CPNL	The positive and negative correlations of the labels were utilized to extend the BR algorithm.	2018
	[73]	GBRAML	From a bottom-up perspective, three granulation operators were constructed in sequence to form three granular structures. In the lower-level granulation operators, auxiliary labels were introduced to enhance the informativeness and representativeness of each label.	2022

The correlation between intervals simultaneously trains the multi-label model. The low-rank structure is adopted to capture the complex correlation between labels, and the incomplete label matrix is obtained by using the label correlation. CLSF first defines the basic elements and features of each label, which reflect the internal characteristics and the connections between labels. In addition, the process of calculating the basic elements of a single label is provided. Secondly, by considering the overlap of the basic element sets determined by different labels, the correlation between labels and the correlation judgment matrix corresponding to the label set are described. Therefore, several labels with strong relationships are assigned to a related label group. At the same time, the local and global label correlations can be calculated. A-GCN uses the label graph to learn the global label correlation with word embeddings.

② **Local relation algorithm**

In the local relation algorithm, label correlations exist in a part of the training data. In this case, the dependencies of labels only exist in certain data. If such label correlations are extracted or used from a global perspective, unnecessary or even misleading constraints will be imposed on all instances, which will reduce the performance of the classification model. These algorithms that consider label correlations locally are LPLC[80]. LPLC considers label correlations locally and finds the positive and negative label correlations for each label for all training instances. Then, for each test instance, based on the local positive and negative label correlations of its *k* nearest neighbors, the maximum a posteriori probability is used for prediction. Ma et al.[81] proposed a new framework with local feature selection and local label correlations. In this framework, it is assumed that instances can be clustered into different groups, and the feature selection weights and label correlations can only be shared by instances in the same group. This framework includes a group-specific feature selection process and a group-specific label selection process. The former projects instances into different groups by extracting instance-group correlations, and the latter process selects the label set for each instance based on the relevant group by extracting group-label correlations and learns a single-label classifier to predict each element of the power set of this subset to construct each element of the set. The CC algorithm randomly places labels in a sequence and builds binary classifiers for each label.

③ **Global - Local Relationship Algorithm**

The global-local composition relationship algorithm takes into account both global and local label correlations to establish an efficient classification model. For example, GLOCAL[82] learns global and local label correlations through manifold regularization. *GLkEL* [83] selects the most relevant k-label set from the label space by approximating the joint mutual information to evaluate the global label correlation. Then, it clusters the training data into different groups and evaluates the local label correlation within each group. LFGLC [84] integrates global and local label correlations to extract label-specific features for each label. Liu et al. [85] calculate a global label correlation matrix throughout the label space and assign a local label correlation matrix to each instance set based on the cosine similarity of labels within the cluster. Based on the assumption that label correlations can be transferred from the original category space to the numerical label space, they add global and local label correlation regularization terms and integrate importance estimation and model training into a unified framework. Table 5 enumerates the high-order algorithms of label correlations.

5.1.2 Research on the Correlation of Labels

(1) Feature compression based on label correlation

To eliminate redundant and irrelevant features, researchers have proposed many methods to compress the features of multi-label data. Many of these methods select features or achieve feature transformation by using label correlations. In Reference [86], a filtering feature selection method is proposed; the minimum redundancy and maximum relevance (mRMR); mutual information is used to measure the importance of labels; and the relationship between features and labels is estimated through various weighting strategies.

(2) Feature expansion based on label correlation

The above method compresses the original feature space based on label correlation. Some algorithms expand features by using label correlation, based on the stacked BR algorithm[17], where the binary classifier in the second layer of BR selects the strongly correlated output from the first layer to expand the original feature space. In reference [87], a classifier chain model is proposed to handle multi-label problems. The main innovation lies in that it selects a directed acyclic graph to model the label correlation and measures the label correlation through conditional entropy, thereby maximizing the sum of the correlations between all labels represented in the graph; then

Table 5 High-order Algorithm

Table 5 High-order algorithms

Category	Literature	Model / Method	Brief Introduction	year
Global relation algorithm	[18]	CC	All labels are placed in a random sequence. The output of the binary classifier of the previous label is added as a new feature to the original feature space of the label, and the corresponding binary classifier is learned based on this sequence. The label correlation is constructed on all the training data.	2011
	[76]	MLLS	In this framework, a common subspace is shared by multiple labels, including several well-known algorithms as special cases, thereby clarifying their intrinsic relationships.	2008
	[77]	ML-LPC	The correlation between learning labels is studied simultaneously while training the multi-label model. The low-rank structure is adopted to capture the complex correlation between labels, and the incomplete label matrix is obtained by using the label correlation.	2014
	[78]	CLSF	Defining the basic elements and characteristics of each label reflects the internal characteristics and the connections between the labels. It also provides the calculation of the basis of a single label. The process of this element. By considering the overlap of the basic element sets determined by different labels, the correlation of the labels and the corresponding correlation judgment matrix corresponding to the label set of 2020 are described.	
	[79]	A-GCN	Use the label graph to learn the global label correlation with word embeddings	2020
	[80]	LPLC	Locally consider the label correlation to find the positive and negative label correlations of each label of the article title for all training instances. Then, for each test instance, based on the local positive and negative label correlations of its *k* nearest neighbors, the maximum a posteriori probability is used for prediction.	2017
	[81]	Local feature s, local labels	Suppose instances can be clustered into different groups, and the feature selection weights and label correlations can only be shared by instances within the same group. This framework includes a group-specific feature selection process and a label-specific group selection process. The former projects instances into different groups by extracting instance-group correlations. The latter process selects labels for each instance based on the relevant groups by extracting group-label correlations.	
Global - Local Group Correlation algorithm	[82]	GLOCAL	Learning global and local label correlations through manifold regularization	2018
	[83]	GLkEL	The most relevant k-label set is selected from the label space through the approximation of joint mutual information to evaluate the global label correlation. Then, it clusters the training data into different groups and evaluates the local label correlation in each group to calculate a global label correlation matrix in the entire label space. Secondly, based on the cosine similarity of the labels within the cluster, a local label correlation matrix is assigned to each instance label importance set. Based on the assumption that the label correlation can be transferred from the original category space to the numerical label space, the global and local label correlation regularization terms are added in 2022. Finally, the importance estimation and model training are integrated into a unified framework.	2018
	[84]			

Based on this directed acyclic graph and the predictions, the original feature space is extended by adding the results of the binary classifier corresponding to the original labels; finally, a binary classifier is trained based on the extended features. ML-LOC [88] is also a feature extension algorithm based on label correlation.

(3) Label embedding based on label correlation

Label embedding is an important multi-label classification algorithm, which can jointly extract the information of all labels to achieve better performance. Chen et al. [89] proposed to use the graph convolutional network to learn label embedding and the correlation between labels, and use the fusion layer to combine the label information with the context semantic information of the text. Wang et al. [90] proposed a new cross-view-based model, the Robust Cross-View Embedding with Multi-Label Classification Discrimination Structure (RCEDS). This method achieves a robust and discriminative embedding. In RCEDS, a new hypergraph fusion technique is designed to utilize the complementarities between the feature space and the label space, and simultaneously utilize the bilateral metric learning to mine the consistency between the feature space and the label space.

5.2 Specific label features

In 2014, Zhang et al. [91] proposed a boosting algorithm, which first introduced the concept of label-specific features. Most existing multi-label methods typically use the same instance expressions to build classification models for different labels. In other words, different labels use the same feature matrix during the learning process. However, the boosting algorithm argues that labels should have their own expressions, so different labels should use appropriate feature representations. Only by utilizing these features can the performance of the classification model be further improved. These functions are called "label-specific functions". The concept of label-specific features is significantly different from the concept of traditional feature compression. In the concept of traditional feature compression, features are generally compressed through feature extraction or feature selection.

Labels provide a unified feature representation. Label-specific functionality refers to the functionality related to a specific label rather than the functionality related to all labels.

At present, there are mainly two methods for constructing the specific features of labels: feature extraction and feature selection. The former is represented by LIFT[91], and the latter by LLSF[92]. Table 6 enumerates the research on specific label characteristics.

5.2.1 Feature extraction based on the characteristics of specific labels

LIFT extracts label-specific features for each label through feature extraction. Specifically, samples related to any label are regarded as positive samples, while the remaining samples are regarded as negative samples. k-means is used respectively to cluster the positive sample set and the negative sample set, and the distance from the samples to the cluster centers is calculated. These distances form the new sample features. Next, a binary classifier is learned on the space of these new label-specific functions. For different labels, the distribution of positive and negative samples is different, so the constructed label-specific features are different from each other. Based on a large number of experiments, its extraordinary performance is proved. Since then, label-specific functions have aroused widespread interest in the academic community, and a series of algorithms have been proposed successively.

Based on LIFT and LF-LPLCl[93], which integrate label-specific features and local pairwise label correlations, the specific features of each label are expanded by combining the relevant features from the related labels, which enriches the semantic information of the labels and solves the problem of class imbalance to a certain extent. LETTER[94] extracts label-specific features from the instance and feature levels. At the instance level, sparse and prototype constraints are used to find more discriminative instance centers; at the feature level, clusters are utilized to find feature centers from the original features of positive and negative instances. The final label-specific features are composed of the centers extracted from the above two levels. Fan et al. [95] proposed a new LFFS method based on label correlation and feature redundancy. Firstly, ridge

Table 6 Specific Label Characteristics

Table 6 Features of lable-specific

Category	Literature	Model / Method	Brief Introduction	year
Based on the spec ific characteristics of the label Extraction	[91]	LIFT	Extract label-specific features for each label through feature extraction	2015
	[92]	LF-LPLCl	It integrates the specific features of labels and the local pairwise label correlations. The specific features of each label are expanded by combining the relevant features in the related labels. This enriches the semantic information of the labels and solves the problem of class imbalance to a certain extent.	2018
	[94]	LETTER	Extract the label-specific features from the instance and feature levels. At the instance level, use sparsity and prototype constraints to find more discriminative instance centers. At the feature level, utilize clustering to find the feature centers from the original features of positive and negative instances. The final label-specific 2020 features are composed of the centers extracted from the above two levels.	
	[95]	LFFS	Feature selection matrix and low-dimensional embedding are established by using ridge regression. Then, the label correlation is mined by adopting low-dimensional embedding to maintain the global and local structure of the original label space. Finally, the feature redundancy is analyzed by using cosine similarity to generate a low-redundancy feature subset.	2022
	[96]	"LC domain set"	The LC is explored by calculating the similarity between labels and the relevant labels are divided into multiple label subsets. Then, a new neighborhood relationship is proposed, which utilizes the nearest neighbor information distribution of instances under the relevant labels to solve the problem of neighborhood granularity selection.	2022
Based on specific marks Sign the characteristic of the feature "Zheng Xuan"	[92]	LLSF	The objective function assumes that strongly correlated labels have more label-specific features than weakly correlated labels. Feature selection is achieved through linear regression, which can learn a binary classification model based on the selected features.	2015
	[97]	NSLSF	Convert logical labels to numerical labels to convey more semantic information and embed label correlations	2018
	[98]	Based on MFNRS and MRMR	For multi-label data with missing labels, the sample correlation coefficient, label complementary matrix and label-specific feature matrix are constructed, and the restoration of missing labels is achieved in the linear regression model. Secondly, the edge-based fuzzy neighborhood radius, fuzzy neighborhood similarity relation and modulo 2022 fuzzy neighborhood information granules are established. The multi-label neighborhood rough set is combined with the fuzzy neighborhood rough set to establish the multi-label neighborhood rough set model.	
	[99]	RWFS	Based on the change ratios of the two types, a more reliable feature ranking is provided by simultaneously considering the feature correlation assessment ratios of the two types.	2022

Regression establishes a feature selection matrix and low-dimensional embedding; then, it uses low-dimensional embedding to mine label correlations and maintain the global and local structure of the original label space; finally, it uses cosine similarity to analyze feature redundancy and generate a low-redundancy feature subset. Wu et al. [96] introduced a new domain set model considering LC. Firstly, they explored LC by calculating the similarity between labels and divided the relevant labels into multiple label subsets; then, they proposed a new neighborhood relationship and used the nearest neighbor information distribution of instances under the relevant labels to solve the problem of neighborhood granularity selection.

5.2.2 Feature selection based on the characteristics of specific labels

The above-mentioned algorithms all adopt feature transformation to extract the features specific to the labels. However, the LLSF algorithm proposed by Huang et al. [92] learns the label-specific features through feature selection techniques. LLSF assumes that each label is only related to some original features, and it expresses this sparsity in constrained linear regression. The nonzero regression parameters indicate that the corresponding features are specific to the label, while others are not.

The objective function of LLSF assumes that strongly correlated labels have more label-specific features than weakly correlated labels. Since LLSF achieves feature selection through linear regression, it can learn a binary classification model based on the selected features. NSLSF [97] considers that the sparsity assumption does not hold in some applications and proposes a feature selection method based on feature selection to select label-specific features. It converts logical labels to numerical labels to convey more semantic information and embed label correlations. MCUL also utilizes norm regularization on the coefficient matrix to learn sparse label-specific features, thereby handling missing and completely unobserved labels. Sun et al. [98] propose a feature selection method based on multi-label fuzzy neighborhood rough set (MFNRS) and maximum relevance minimum redundancy (MRMR), which can be used for multi-label data with missing labels. Firstly, for multi-label data with missing labels, the sample correlation coefficient, label complementary matrix, and label-specific feature matrix are constructed, and the restoration of missing labels is achieved in the linear regression model. Secondly, a method based on edge

The fuzzy neighborhood radius, fuzzy neighborhood similarity relation and fuzzy neighborhood information granules of the origin; The multi-label neighborhood rough set is combined with the fuzzy neighborhood rough set to establish the multi-label neighborhood rough set model. Based on the algebra and information view, the MFNRS uncertainty measure based on the entropy of the fuzzy neighborhood is proposed, and the label-related MRMR model based on the mutual information of the fuzzy neighborhood is improved to evaluate the performance of candidate features. Hu et al. [99] proposed a method based on weighted feature selection (RWFS), which provides a more reliable feature ranking by considering the evaluation ratios of feature correlations of both types simultaneously based on two types of change ratios.

5.3 Other research directions

5.3.1 Category imbalance

The problem of class imbalance has become an inherent feature of many labeled datasets, where the distribution of samples and their corresponding labels in the data space is uneven. The imbalance problem in multi-label text classification poses challenges to multi-label data analysis, which can be viewed from three perspectives: within labels, between labels, and between label sets.

The problem of class imbalance is also widespread in traditional binary and multi-class problems. Therefore, the solution ideas for this problem can provide some inspirations for dealing with multi-label imbalance problems. Zhang et al. [100] proposed a COCOA algorithm to solve the class imbalance problem in multi-label applications. Charte et al. [101] proposed several complex measurement standards for multi-label imbalance degree. At the same time, Charte et al. also proposed under-sampling and over-sampling algorithms (LP-RUS and LP-ROS) for preprocessing multi-label data imbalance. Pereira et al. [102] proposed MLTL, which is a similar heuristic-based method. This method uses the classical Tomek Link algorithm to solve the imbalance problem and can be used as an under-sampling or cleaning technique. The MLSMOTE algorithm [103] discusses an over-sampling technique for multi-label learning, which uses a similar strategy to SMOTE. First, the MLSMOTE algorithm identifies the minority labels through MeanIR and CVIR; then, it synthesizes new samples for these labels.

Another proposed method is MLSOL [104]. This method mainly analyzes imbalance by observing the local characteristics of minority group samples rather than the imbalance of the entire data set. In [105], an adaptation method was proposed to solve the imbalance problem in MLC, which dynamically adjusts the loss costs of positive and negative samples based on an asymmetric stage loss function. Rastogi et al. [106] dealt with the class imbalance problem by constructing a label weight matrix. The weight estimation was guided by the frequencies of label presence, absence, and non-observation. By utilizing the sensitive weights of class imbalance and the auxiliary label correlation, a weighted square loss function with discriminatory label weights was introduced to guide the completion of missing labels.

In conclusion, through sampling, the common intuitive processing of the imbalance of multi-label problem types has been achieved, and it is necessary to further study how to use label correlations in sampling. In addition, through ensemble learning and cost sensitivity, the adverse effects of class imbalance can be eliminated as much as possible.

5.3.2 Label lost

In many practical applications, obtaining all the true and relevant labels of all samples in the training set is impractical due to the following two main reasons [107-108]. On the one hand, many applications contain many that require a large number of label classes; on the other hand, the meanings of different labels may overlap, making it difficult to distinguish them completely. Therefore, learning models based on such partially labeled data may not accurately capture the label correlations and the relationships between labels and features. Taha et al. [109] proposed the missing label processing method based on the aggregation of features and label graphs (GB-AS) and the missing label propagation method based on the unified graph (UG-MLP). On the one hand, the GB-AS algorithm obtains the initial label matrix based on two document-level similarities, namely the feature-based weighted representation and the label-based weighted representation. On the other hand, it introduces the UG-MLP to construct a hybrid graph that combines GB-AS and label correlations into a single basis, obtaining high-order label correlations from incomplete training data and using them to supplement the missing label matrix and guide the establishment of multi-label classification models. Ai et al. [110] proposed an improved MLTSVM (LSFML-MLTSVM) that utilizes the label-specific features of the missing labels. LSFML-MLTSVM first extracts the label-specific features through semi-supervised clustering analysis and then obtains the structural information of the samples and the geometric information of the edge distribution. Sun et al. [111] proposed a multi-label classification method based on two-stage neighborhoods for incomplete data classification with missing labels in the neighborhood decision system. Firstly, to solve the problem of manually selecting the neighborhood radius and balancing samples within the neighborhood, the neighborhood radius based on the feature distribution function is defined, and the similarities and differences between samples are calculated respectively through the identifiable matrix and the non-identifiable matrix. On this basis, a recovery method for the missing feature values is proposed. Secondly, considering the nonlinear relationship between features, the fuzzy similarity relationship between samples based on neighborhoods is studied based on the Gaussian kernel function. Based on the comprehensive foundation of the fuzzy similarity relationship matrix, the label-specific feature matrix, and the label correlation matrix, an objective function based on the regression model is proposed, and the optimal solutions of the label-specific feature matrix and the label correlation matrix based on the gradient descent strategy are given. In the second stage, a new multi-label classification method for the missing labels is proposed. Finally, a two-stage multi-label classification algorithm is designed.

5.3.3 Label compression

Since the number of labels in many practical multi-label problems can reach to tens of thousands, therefore many studies have shifted their attention to multi-label classification involving a large number of labels, as excessive labels may bring considerable time and space costs to the algorithms. Moreover, many mature and common algorithms, such as BR[14] and ECC[18], are not applicable to handle these excessive labels. To solve this problem, researchers have proposed spatial dimension compression techniques for labels, compressing high-dimensional labels into low-dimensional label spaces and training classification models in the low-dimensional label spaces to reduce the computational burden. Of course, the prediction results in the low-dimensional label spaces must be restored to the original feature space. According to existing studies, label compression can not only shorten the running time of the algorithms but also improve the classification effect. Cao et al. [112] proposed a new label compression coding method that considers both feature and label information simultaneously. The label compression algorithm based on label transformation has the advantages of strong theoretical basis and easy implementation. However, the transformed labels lack the meaning of the original labels, so they are difficult to be interconnected. Label compression algorithms based on label subsets often use group sparse learning, random sampling, Boolean matrix factorization, etc. These algorithms can obtain low-dimensional labels, either directly from the original labels or completely restoring the original feature space. Yu et al. [113] introduced a PML method (PML-LCom) that uses label compression to effectively learn from some multi-label data. PML-LCom first decomposes the observed label data matrix into latent relevant label matrices and irrelevant label matrices, and then decomposes the relevant label matrices into two low-rank matrices, one encoding the compressed labels of the samples and the other exploring the latent label correlations; then, the coefficient matrix of the multi-label predictor is optimized for the compressed label matrix. Yang et al. [114] proposed a multi-synchronous compression transformation method (MSST) to transform the processed data. Based on synchronous compression transformation, iterative redistribution is used instead of the original data to complete pattern recognition. In addition, for the feature similarity of the samples, the compressed label matrix is regularized, and consistency optimization is performed on the label matrix and the predictor. Therefore, the label compression algorithm has strong interpretability. Table 7 enumerates some model analyses in other research directions.

6 Summary and Outlook

The research on multi-label text classification has achieved remarkable results with the advent of deep learning, especially after the arrival of BERT, which has greatly improved the accuracy of related research. Although there are already quite mature and practical technologies in this field, there are still some intractable problems worthy of exploration by researchers together:

The problem of the lack of datasets and low-quality datasets. Multi-label text classification is much more complex than single-label text classification. Therefore, the lack of dataset resources greatly limits the development of models by researchers, and datasets in specific fields such as healthcare, law, finance, and architecture are very scarce. Secondly, due to the widespread problem of uneven data distribution in the current datasets in this field, it is mainly manifested as the long-tail problem, that is, most of the documents in the same dataset are only related to one or very few labels. Therefore, creating more high-quality datasets is a long-discussed issue.

The dynamic division problem of text-related labels. Currently, multi-label texts

Table 7 Other Research Directions

Table 7 Other research directions

Category	Literature	Model/Method	Brief Introduction	year
Category imbalance	[103]	MLSMOTE	Identify a few labels through MeanIR and CVIR, and then synthesize new samples for these labels.	2015
	[105]	Adaptive method	Dynamically adjust the loss cost of positive and negative samples based on the asymmetric stage loss function	2019
	[104]	MLSOL	Analyze the imbalance by observing the local characteristics of the samples of minority groups instead of the imbalance of the entire data set.	2019
	[102]	MLTL	The classic Tomek Link algorithm is adopted to solve the imbalance problem and can be used as an undersampling or cleaning technique.	2020
	[106]	Label weight matrix	Weight estimation is guided by the frequencies of label presence, absence, and unobservation. By leveraging the sensitive weights of class imbalance and the correlation of auxiliary labels, a weighted square loss function with discriminative label weights is introduced to guide the completion of missing labels.	2022
The label is lost.	[109]	GB-AS GB-AS	The GB-AS algorithm is adopted. Based on the two document-level similarities of feature-based weighted representation and label-based weighted representation, the initial label matrix is obtained. The UG-MLP is introduced to construct a hybrid graph, combining GB-AS and label correlation into a single basis, obtaining high-order label correlations from incomplete training data, and using them to supplement the missing label matrix and guide the establishment of the multi-label classification model.	2022
			[110] LSFML-MLTSVM first extracts label-specific features through semi-supervised clustering analysis; then obtains the structural information of the samples and the geometric information of the edge distribution 2022	
	[111]	Two-stage neighborhood	Used for the classification of incomplete data with missing labels in neighborhood decision systems	2022
Label compression	[113]	PML-LCom	Use label compression to effectively learn from partially multi-label data	2015
	[112]	Label compression coding	The Hilbert-Schmidt independence criterion is utilized to maximize the dependence between features and labels, thereby considering both feature and label information simultaneously.	2020
	[114]	MSST	The processed data is transformed. On the basis of synchronous compression transformation, the iterative redistribution is adopted instead of the original data to complete pattern recognition.	2021

Classification mainly relies on supervised learning. When the labels change, the model needs to be re-trained to adapt to the changes. However, re-labeling the data set or training the model both require high costs. Therefore, how to adapt the trained model to the changes of labels at low cost and quickly is a problem worthy of consideration.

The problem of extreme multi-label text classification. Extreme multi-label text classification (XMC) aims to find relevant labels for a given text from an extremely large set of labels. The main difficulty of XMC is that the number of text labels is very large. Currently, the memory usage of the proposed models increases as the label space becomes larger. Therefore, how to reduce the size of extreme multi-label text classification models is one of the main research directions in the future.

The problem of hierarchical multi-label text classification. Many real-world text classification tasks usually handle a large number of closely related categories organized in a hierarchical structure or taxonomy. When dealing with a large number of closely related categories, hierarchical multi-label text classification (HMTC) becomes very challenging. The concept of hierarchical labels: The first-level labels contain the second-level labels, and the second-level labels contain the third-level labels. The difficulty of HMTC lies in considering the planar multi-label that considers the vertical category correlation and the horizontal correlation between categories at the same level; fully modeling the hierarchical dependencies, improving the prediction performance of labels at all levels, especially the lower-level long-tail labels. In addition, the structural features of all categories in the entire hierarchy and the word semantics of their category labels are very helpful for improving the accuracy of text classification of a large number of closely related categories. Therefore, how to design such a model to solve these problems is a difficult point that needs to be urgently solved in the future.

Small sample multi-label text classification is also a current and future research hotspot. In actual application scenarios, obtaining text data may face problems such as multiple classification categories, small sample data, and short texts. The construction of small sample datasets is also more conducive to the model being applied in different fields.

7 Conclusion

This article discusses the concepts, processes, and methods of multi-label text classification in recent years. The literatures of the research direction were reviewed. The multi-label text classification methods were divided into traditional machine learning methods and deep learning methods; the research directions were classified as label correlation, specific label characteristics, class imbalance, label loss and label compression; finally, the challenges and future directions of multi-label text classification were discussed.

References:

[1] TSOUMAKAS G， KATAKIS I.Multi-label classification： an overview[J].International Journal of Data Warehousing and Mining(IJDWM)， 2007， 3： 113.

[2] ZENG Q， ZHAO X， HU X， et al.Learning emotional word embeddings for sentiment analysis[J]. Journal of Intelli-gent & Fuzzy Systems， 2021， 40： 9515-9527.

[3] YAO L， MAO C， LUO Y.Graph convolutional networks for text classification[C]//Proceedings of the AAAI Con-ference on Artificial Intelligence， 2019， 33： 7370-7377.

[4] KALCHBRENNER N， GREFENSTETTE E， BLUNSOM P. A convolutional neural network for modelling sentences[J]. arXiv： 1404.2188， 2014.

[5] LEE J Y， DERNONCOURT F.Sequential short-text clas-sification with recurrent and convolutional neural net-works[J].arXiv： 1603.03827， 2016.

[6] XIAO L， HUANG X， CHEN B， et al.Label-specific docu-ment representation for multi-label text classification[C]// Proceedings of the 2019 Conference on Empirical Meth-ods in Natural Language Processing and the 9th Interna-tional Joint Conference on Natural Language Processing (EMNLP-IJCNLP)， 2019： 466-475.

[7] YANG Y， REN G.HanLP-based technology function matrix construction on Chinese process patents[J]. International Jour-nal of Mobile Computing and Multimedia Communica-

tions(IJMCMC)，2020，11：48-64.

[8] MIKOLOV T，CHEN K，CORRADO G，et al. Efficient esti-mation of word representations in vector space[J].arXiv：1301.3781，2013.

[9] PENNINGTON J，SOCHER R，MANNING C D. Glove：global vectors for word representation[C]//Proceedings of the 2014 Conference on Empirical Methods in Natural Language Processing(EMNLP)，2014：1532-1543.

[10] RADFORD A，NARASIMHAN K，SALIMANS T，et al. Improving language understanding by generative pre-training[EB/OL].(2018) [2020-11-30]. https：//s3-us-west-2. amazonaws.com/openaiassets/researchcovers/languageunsu-pervised/languageunderstandingpaper.pdf.

[11] DEVLIN J，CHANG M W，LEE K，et al.Bert：pre-training of deep bidirectional transformers for language under-standing[J].arXiv：1810.04805，2018.

[12] QIU X，SUN T，XU Y，et al.Pre-trained models for nat-ural language processing：a survey[J].Science China Tech-nological Sciences，2020，63：1872-1897.

[13] GHOSH S，DESARKAR M S.Class specific TF-IDF boost-ing for short-text classification：application to short-texts generated during disasters[C]//Companion Proceedings of the The Web Conference 2018，2018：1629-1637.

[14] BOUTELL M R，LUO J，SHEN X，et al.Learning multi-label scene classification[J].Pattern Recognition，2004，37：1757-1771.

[15] GODBOLE S，SARAWAGI S.Discriminative methods for multi-labeled classification[C]//Pacific-Asia Conference on Knowledge Discovery and Data Mining，2004：22-30.

[16] ALVARES-CHERMAN E，METZ J，MONARD M C.Incor-porating label dependency into the binary relevance frame-work for multi-label classification[J]. Expert Systems with Applications，2012，39：1647-1655.

[17] TSOUMAKAS G，DIMOU A，SPYROMITROS E，et al. Correlation-based pruning of stacked binary relevance models for multi-label learning[C]//Proceedings of the 1st International Workshop on Learning from Multi-Label Data，2009：101-116.

[18] READ J，PFAHRINGER B，HOLMES G，et al. Classifier chains for multi-label classification[C]//Joint European Conference on Machine Learning and Knowledge Dis-covery in Databases，2009：254 269.

[19] TSOUMAKAS G，VLAHAVAS I.Random k-labelsets：an ensemble method for multilabel classification[C]//Euro-pean Conference on Machine Learning，2007：406-417.

[20] READ J，PFAHRINGER B，HOLMES G.Multi-label clas-sification using ensembles of pruned sets[C]//2008 Eighth IEEE International Conference on Data Mining，2008：995-1000.

[21] HüLLERMEIER E，FüRNKRANZ J，CHENG W，et al. Label ranking by learning pairwise preferences[J].Artifi-cial Intelligence，2008，172：1897-1916.

[22] FüRNKRANZ J，HüLLERMEIER E，LOZA MENCíA E，et al.Multilabel classification via calibrated label rank-ing[J].Machine Learning，2008，73：133-153.

[23] ZHANG M L，ZHOU Z H.ML-KNN：a lazy learning approach to multi-label learning[J].Pattern Recognition，2007，40：2038-2048.

[24] HUANG J，LI G，WANG S，et al.Categorizing social multimedia by neighborhood decision using local pair-wise label correlation[C]//2014 IEEE International Con-ference on Data Mining Workshop，2014：913-920.

[25] CLARE A，KING R D.Knowledge discovery in multi-label phenotype data[C]//European Conference on Principles of Data Mining and Knowledge Discovery，2001：42-53.

[26] BLOCKEEL H，DE RAEDT L，RAMON J.Top-down induction of clustering trees[J].arXiv：cs/0011032，2000.

[27] ZHANG M L，ZHOU Z H.Multilabel neural networks with applications to functional genomics and text cate-gorization[J].IEEE Transactions on Knowledge and Data Engineering，2006，18：1338-1351.

[28] ELISSEEFF A，WESTON J.A kernel method for multi-labelled classification[C]//Advances in Neural Informa-tion Processing Systems，2001.

[29] KIM Y.Convolutional neural networks for sentence clas-sification[J].arXiv：1408.5882，2014.

[30] LIU J，CHANG W C，WU Y，et al.Deep learning for extreme multi- label text classification[C]//Proceedings of the 40th International ACM SIGIR Conference on Research and Development in Information Retrieval，2017：115-124.

[31] SHIMURA K，LI J，FUKUMOTO F.HFT-CNN：learning hierarchical category structure for multi-label short text categorization[C]//Proceedings of the 2018 Conference on Empirical Methods in Natural Language Processing，2018：811-816.

[32] YANG W，LI J，FUKUMOTO F，et al.HSCNN：a hybrid-siamese convolutional neural network for extremely imbal-anced multi-label text classification C]//Proceedings of the 2020 Conference on Empirical Methods in Natural Lan-guage Processing(EMNLP)，2020：6716-6722.

[33] NAM J，LOZA MENCíA E，KIM H J，et al. Maximizing subset accuracy with recurrent neural networks in multi-label classification[C]//Advances in Neural Information Processing Systems，2017.

[34] CHEN G，YE D，XING Z，et al.Ensemble application of convolutional and recurrent neural networks for multi-label text categorization[C]//2017 International Joint Con-ference on Neural Networks (IJCNN)，2017：2377-2383.

[35] YANG P，LUO F，MA S，et al.A deep reinforced sequence-to-set model for multi-label classification[C]//Proceedings

of the 57th Annual Meeting of the Association for Computational Linguistics，2019：5252-5258.

[36] LIN J，SU Q，YANG P，et al.Semantic-unit-based dilated convolution for multi-label text classification[J].arXiv：1808.08561，2018.

[37] YANG P，SUN X，LI W，et al.SGM：sequence generation model for multi-label classification[J].arXiv：1806.04822，2018.

[38] YANG Z，YANG D，DYER C，et al.Hierarchical atten-tion networks for document classification[C]//Proceedings of the 2016 Conference of the North American Chap-ter of the Association for Computational Linguistics：Human Language Technologies，2016：1480-1489.

[39] HONG M，WANG M，LUO L，et al.Combining gated recur-rent unit and attention pooling for sentimental classifi-cation[C]//Proceedings of the 2018 2nd International Con-ference on Computer Science and Artificial Intelligence，2018：99-104.

[40] LI Y，CAI Y，LEUNG H F，et al.Improving short text modeling by two-level attention networks for sentiment classification[C]//International Conference on Database Sys-tems for Advanced Applications，2018：878-890.

[41] YOU R，ZHANG Z，WANG Z，et al.Attentionxml：label tree-based attention-aware deep model for high-performance extreme multi-label text classification[C]//Advances in Neural Information Processing Systems，2019.

[42] YAO C，CAI M.A novel optimized convolutional neural network based on attention pooling for text classifica-tion[C]//Journal of Physics：Conference Series，2021.

[43] XIAO Y，LI Y，YUAN J，et al.History-based attention in Seq2Seq model for multi-label text classification[J]. Knowledge-Based Systems，2021，224：107094.

[44] LIU B，LIU X，REN H，et al.Text multi-label learning method based on label- aware attention and semantic dependency[J].Multimedia Tools and Applications，2022，81：7219-7237.

[45] SONG R，LIU Z，CHEN X，et al.Label prompt for multi-label text classification[J].Applied Intelligence，2022，53：8761-8775.

[46] CHANG W C，YU H F，ZHONG K，et al.Taming pre-trained transformers for extreme multi-label text classi-fication[C]//Proceedings of the 26th ACM SIGKDD Inter-national Conference on Knowledge Discovery & Data Mining，2020：3163-3171.

[47] GONG J，TENG Z，TENG Q，et al.Hierarchical graph transformer-based deep learning model for large-scale multi-label text classification[J].IEEE Access，2020，8：30885-30896.

[48] JIANG T，WANG D，SUN L，et al.Lightxml：transformer with dynamic negative sampling for high-performance extreme multi-label text classification[C]//Proceedings of the AAAI Conference on Artificial Intelligence，2021：7987-7994.

[49] YE C，ZHANG L，HE Y，et al.Beyond text：incorporating metadata and label structure for multi-label document clas-sification using heterogeneous graphs[C]//Proceedings of the 2021 Conference on Empirical Methods in Natural Language Processing，2021：3162-3171.

[50] CHEN Q，DU J，ALLOT A，et al.LitMC BERT：transformer-based multi-label classification of biomedical literature with an application on COVID-19 literature curation[J]. IEEE/ACM Transactions on Computational Biology and Bioinformatics，2022，19(5)：2584-2595.

[51] ZHANG R，WANG Y S，YANG Y，et al. Exploiting local and global features in transformer-based extreme multi-label text classification[J] arXiv：2204.00933，2022.

[52] KIPF T N，WELLING M.Semi-supervised classification with graph convolutional networks[J]. arXiv：1609.02907，2016.

[53] LIU P，QIU X，HUANG X.Recurrent neural network for text classification with multi-task learning [J].arXiv：1605.05101，2016.

[54] VELIKOVI P，CUCURULL G，CASANOVA A，et al.Graph attention networks[J].arXiv：1710 10903，2017.

[55] PAL A，SANKARASUBBU M，SELVAKUMAR M.Multi-label text classification using attention-based graph neu-ral network[J].arXiv：2003.11644，2020.

[56] DING K，WANG J，LI J，et al.Be more with less：hyper-graph attention networks for inductive text classifica-tion[C]//Proceedings of the 2020 Conference on Empir-ical Methods in Natural Language Processing(EMNLP)，2020：4927-4936.

[57] ZONG D，SUN S.GNN-XML：graph neural networks for extreme multi-label text classification[J]. arXiv：2012.05860，2020.

[58] ZHENG S，ZHOU J，MENG K，et al.Label-dividing gated graph neural network for hierarchical text classification[C]// 2022 International Joint Conference on Neural Networks (IJCNN)，2022：1-8.

[59] ZHOU C，SUN C，LIU Z，et al.A C-LSTM neural net-work for text classification[J].arXiv：1511. 08630，2015.

[60] ZHANG R，LEE H，RADEV D.Dependency sensitive con-volutional neural networks for modeling sentences and documents[C]//Proceedings of NAACL-HLT，2016：1512-1521.

[61] LIU W，PANG J，LI N，et al.Research on multi-label text classification method based on tALBERT-CNN[J].Inter-national Journal of Computational Intelligence Systems，2021，14：1-12.

[62] YAN Y，LIU F A，ZHUANG X，et al.An R-Transformer_ BiLSTM model based on attention for multi-label text classification[J].Neural Processing Letters，2022：1-24.

[63] XIAO L，ZHANG X，JING L，et al.Does head label help for long-tailed multi-label text classification [C]//Proceed-ings of the AAAI Conference on Artificial Intelligence，2021：14103-14111.

[64] ZHANG X，ZHANG Q W，YAN Z，et al. Enhancing label correlation feedback in multi-label text classification via multi-task learning[J].arXiv：2106.03103，2021.

[65] KHATAEI MARAGHEH H，GHAREHCHOPOGH F S，MAJIDZADEH K，et al.A new hybrid based on long short-term memory network with spotted hyena optimi-zation algorithm for multi-label text classification[J].Math-ematics，2022，10：488.

[66] GIBAJA E，VENTURA S.A tutorial on multilabel learn-ing[J].ACM Computing Surveys(CSUR)，2015，47：1-38.

[67] BAO J，WANG Y，CHENG Y.Asymmetry label correlation for multi-label learning[J].Applied Intelligence，2022，52：6093-6105.

[68] HUANG R，KANG L.Local positive and negative label correlation analysis with label awareness for multi-label classification[J].International Journal of Machine Learn-ing and Cybernetics，2021，12：2659-2672.

[69] LI Y K，ZHANG M L，GENG X.Leveraging implicit relative labeling- importance information for effective multi-label learning[C]//2015 IEEE International Confer-ence on Data Mining，2015：251-260.

[70] MENCíA E L，FURNKRANZ J.Pairwise learning of mul-tilabel classifications with perceptrons[C]//2008 IEEE Inter-national Joint Conference on Neural Networks(IEEE World Congress on Computational Intelligence)，2008：2899-2906.

[71] WU G，TIAN Y，LIU D.Cost-sensitive multi-label learning with positive and negative label pairwise correlations[J]. Neural Networks，2018，108：411-423.

[72] XU H，XU L.Multi-label feature selection algorithm based on label pairwise ranking comparison transformation[C]// 2017 International Joint Conference on Neural Networks (IJCNN)，2017：1210-1217.

[73] ZHANG Y，ZHAO T，MIAO D，et al.Granular multila-bel batch active learning with pairwise label correlation[J]. IEEE Transactions on Systems, Man，and Cybernetics：Systems，2021，52：3079-3091.

[74] WANG R，YE S，LI K，et al.Bayesian network based label correlation analysis for multi-label classifier chain[J]. Information Sciences，2021，554：256-275.

[75] HE Z F，YANG M，GAO Y，et al.Joint multi-label clas-sification and label correlations with missing labels and feature selection[J].Knowledge-Based Systems，2019，163：145-158.

[76] JI S，TANG L，YU S，et al.Extracting shared subspace for multi-label classification[C]//Proceedings of the 14th ACM SIGKDD International Conference on Knowledge Dis-covery and Data Mining，2008：381-389.

[77] XU L，WANG Z，SHEN Z，et al.Learning low-rank label correlations for multi-label classification with missing labels[C]//2014 IEEE International Conference on Data Mining，2014：1067-1072.

[78] CHE X，CHEN D，MI J.A novel approach for learning label correlation with application to feature selection of multi-label data[J].Information Sciences，2020，512：795-812.

[79] LI Q，PENG X，QIAO Y，et al.Learning label correla-tions for multi-label image recognition with graph net-works[J].Pattern Recognition Letters，2020，138：378-384.

[80] HUANG J，LI G，WANG S，et al.Multi-label classifica-tion by exploiting local positive and negative pairwise label correlation[J].Neurocomputing，2017，257：164-174.

[81] MA J，CHIU B C Y，CHOW T W.Multilabel classifica-tion with group-based mapping：a framework with local feature selection and local label correlation[J].IEEE Trans-actions on Cybernetics，2020，52(6)：4596-4610.

[82] ZHU Y，KWOK J T，ZHOU Z H.Multi-label learning with global and local label correlation[J].IEEE Transactions on Knowledge and Data Engineering，2017，30：1081-1094.

[83] YAN Y，LI S，ZHANG X，et al.k-Labelsets for Multi-media classification with global and local label correla-tion[C]//International Conference on Multimedia Model-ing，2018：177-188.

[84] WENG W，WEI B，KE W，et al.Learning label-specific features with global and local label correlation for multi-label classification[J].Applied Intelligence，2022：1-17.

[85] LIU Y，CAO F.A relative labeling importance estimation algorithm based on global-local label correlations for multi-label learning[J].Applied Intelligence，2022：1-19.

[86] LIU L，ZHANG J，LI P，et al.A label correlation based weighting feature selection approach for multi-label data[C]// International Conference on Web-Age Information Man-agement，2016：369-379.

[87] LEE J，KIM H，KIM N R，et al.An approach for multi-label classification by directed acyclic graph with label correlation maximization[J].Information Sciences，2016，351：101-114.

[88] HU Q，PEDRYCZ W，YU D，et al.Selecting discrete and continuous features based on neighborhood decision error minimization[J].IEEE Transactions on Systems，Man，and Cybernetics，Part B(Cybernetics)，2009，40：137-150.

[89] CHEN Z，LI S，YE L，et al.Multi-label classification of legal text based on label embedding and capsule net-work[J].Applied Intelligence，2022，53：6873-6886.

[90] WANG K.Robust cross-view embedding with discrimi-nant structure for multi-label classification[J].IEEE Access，2021，9：117596-117607.

[91] ZHANG M L, WU L.Lift: multi-label learning with label-specific features[J].IEEE Transactions on Pattern Analysis and Machine Intelligence, 2014, 37: 107-120.

[92] HUANG J, LI G, HUANG Q, et al.Learning label specific features for multi-label classification[C]//2015 IEEE Inter-national Conference on Data Mining, 2015: 181-190.

[93] WENG W, LIN Y, WU S, et al.Multi-label learning based on label-specific features and local pairwise label cor-relation[J].Neurocomputing, 2018, 273: 385-394.

[94] GUAN Y, LI W, ZHANG B, et al.Multi-label classifica-tion by formulating label-specific features from simulta-neous instance level and feature level[J]. Applied Intelli-gence, 2021, 51: 3375-3390.

[95] FAN Y, CHEN B, HUANG W, et al.Multi-label feature selection based on label correlations and feature redun-dancy[J].Knowledge-Based Systems, 2022, 241: 108256.

[96] WU Y, LIU J, YU X, et al.Neighborhood rough set based multi-label feature selection with label correlation[J].Con-currency and Computation: Practice and Experience, 2022, 34: e7162.

[97] WENG W, CHEN Y N, CHEN C L, et al.Non-sparse label specific features selection for multi-label classifi-cation[J].Neurocomputing, 2020, 377: 85-94.

[98] SUN L, YIN T, DING W, et al.Feature selection with missing labels using multilabel fuzzy neighborhood rough sets and maximum relevance minimum redundancy[J]. IEEE Transactions on Fuzzy Systems, 2021, 30: 1197-1211.

[99] HU L, GAO L, LI Y, et al.Feature-specific mutual infor-mation variation for multi-label feature selection[J]. Infor-mation Sciences, 2022, 593: 449-471.

[100] ZHANG M L, LI Y K, YANG H, et al.Towards class-imbalance aware multi-label learning[J].IEEE Transac-tions on Cybernetics, 2020, 52(6): 4459-4471.

[101] CHARTE F, RIVERA A, JESUS M J D, et al.A first approach to deal with imbalance in multi-label datas-ets[C]//International Conference on Hybrid Artificial Intel-ligence Systems, 2013: 150-160.

[102] PEREIRA R M, COSTA Y M, SILLA JR C N. MLTL: a multi-label approach for the tomek link undersam-pling algorithm[J].Neurocomputing, 2020, 383: 95-105.

[103] CHARTE F, RIVERA A J, DEL JESUS M J, et al. MLSMOTE: approaching imbalanced multilabel learning through synthetic instance generation[J].Knowledge-Based Systems, 2015, 89: 385-397.

[104] LIU B, TSOUMAKAS G.Synthetic oversampling of multi-label data based on local label distribution[C]// Joint Euro-pean Conference on Machine Learning and Knowledge Discovery in Databases, 2019: 180-193.

[105] LUO F F, GUO W Z, CHEN G L.Addressing imbal-ance in weakly supervised multi-label learning[J].IEEE Access, 2019, 7: 37463-37472.

[106] RASTOGI R, KUMAR S.Discriminatory label-specific weights for multi-label learning with missing labels[J]. Neural Processing Letters, 2022, 55: 1397-1431.

[107] DENDAMRONGVIT S, VATEEKUL P, KUBAT M.Irrel-evant attributes and imbalanced classes in multi-label text-categorization domains[J].Intelligent Data Analysis, 2011, 15: 843-859.

[108] WU B, JIA F, LIU W, et al.Multi-label learning with missing labels using mixed dependency graphs[J]. Inter-national Journal of Computer Vision, 2018, 126: 875-896.

[109] TAHA A Y, TIUN S, RAHMAN A H A, et al. Unified graph-based missing label propagation method for mul-tilabel text classification[J].Symmetry, 2022, 14: 286.

[110] AI Q, LI F, LI X, et al.An improved MLTSVM using label-specific features with missing labels[J]. Applied Intel-ligence, 2022, 53: 8039-8060.

[111] SUN L, WANG T, DING W, et al.Two-stage-neighbor-hood-based multilabel classification for incompletedata with missing labels[J].International Journal of Intelligent Systems, 2022, 37(10): 6773-6810.

[112] CAO L, XU J.A label compression coding approach through maximizing dependence between features and labels for multi- label classification[C]//2015 International Joint Conference on Neural Networks (IJCNN),

2015: 1-8.

[113] YU T, YU G, WANG J, et al.Partial multi-label learn-ing using label compression[C]//2020 IEEE International Conference on Data Mining(ICDM), 2020: 761-770.

[114] YANG Y, ZHOU J, LIU J, et al.Epileptic seizure detec-tion based on multi-synchrosqueezing transform and multi-label classification[C]//Signal and Information Process-ing, Networking and Computers, 2023: 1017-1024.
