# Supplementary material for: An Intelligent System for Classifying Patient Complaints Using Machine Learning and Natural Language Processing: Development and Validation Study
Source: J Med Internet Res. 2025 Jan 8;27:e55721. doi: 10.2196/55721 (PMC11754990; doi:10.2196/55721)
Supplement: Multimedia Appendix 3 [file jmir_v27i1e55721_app3.pdf]

(19)国家知识产权局

(12)发明专利

(10)授权公告号 CN 113157918 B
(45)授权公告日 2022.07.22

(21)申请号 202110307421.9

(22)申请日 2021.03.23

(65)同一申请的已公布的文献号
申请公布号 CN 113157918 A

(43)申请公布日 2021.07.23

(73)专利权人 浙江工业大学
地址 310014 浙江省杭州市下城区潮王路18号

(72)发明人 高楠 陈国鑫 陈磊 杨归一 方添斌 俞果

(74)专利代理机构 杭州天正专利事务所有限公司 33201
专利代理师 王兵

(51)Int.Cl.
G06F 16/35(2019.01)
G06F 40/289(2020.01)
G06F 40/30(2020.01)
G06N 20/00(2019.01)

(56)对比文件
CN 107862046 A,2018.03.30
CN 109062893 A,2018.12.21
CN 108241677 A,2018.07.03
CN 110134786 A,2019.08.16
US 2017083983 A1,2017.03.23
傅裕等.基于自注意力机制的冗长商品名称精简方法.《华东师范大学学报(自然科学版)》.2019,(第5期),第113-122、167页.
Nan Gao.et.A Supervised Named Entity Recognition Method Based on Pattern Matching and Semantic Verification.《Journal of Internet Technology》.2020,第21卷(第7期),第1917-1928页.

审查员 葛晓倩

权利要求书5页 说明书11页 附图2页

(54)发明名称

一种基于注意力机制的商品名称短文本分类方法和系统

(57)摘要

一种基于注意力机制的商品名称短文本分类方法,包含:对商品名称进行预处理,去除非中文字段以及一些特殊字符;通过jieba分词,将预处理后的商品短文本分成若干个词,去除停用词,对得到的词进行短补长切,统一词的长度到事先设定好的词个数;将每个词利用Global Entity Linking算法进行实体消歧与链接,通过链接到百度百科的外部知识库,用其结果对短文本中的词扩充解释,并将实体链接的结果利用Bert进行word embedding编码,得到相应的特征向量;将得到的向量喂入Transformer网络,利用self-attention机制,挖掘不同词对于税码分类的共享程度,赋予不同词的不同权重,最后通过Softmax对其进行分类,将概率最高的税码类别作为商品名称所属类别。本发明还包括实施上述发明方法的系统。

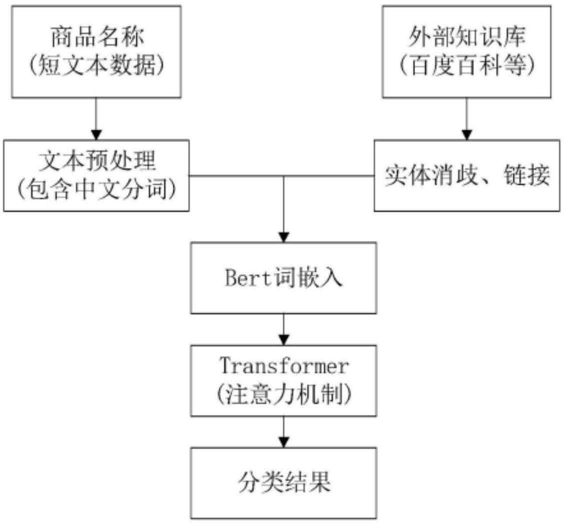

CN 113157918 B

1.一种基于注意力机制的商品名称短文本分类方法，包含以下步骤：

(1)对商品名称进行预处理，只保留中文字段；

(2)通过jieba分词，将预处理后的商品名称短文本分成若干个词，去除停用词，对得到的词进行短补长切，统一词的长度到事先设定好的词个数；

(3)将每个词利用Global Entity Linking算法进行实体消歧与链接，通过链接到百度百科的外部知识库，用其结果对短文本中的词扩充解释，丰富上下文语义信息，并将实体链接的结果利用Bert进行word embedding编码，得到相应的特征向量；具体包含以下步骤：

(3-1)将每个词利用Global Entity Linking算法进行实体消歧与链接，通过链接到百度百科的外部知识库，用其结果对短文本中的词扩充解释，丰富上下文语义信息，所述Global Entity Linking算法的公式如下：

$$T_g = argmax_{\Gamma}\left[\sum_{i=1}^{N}\phi(m_i,e_i) + \sum_{e_i\in\Gamma,e_j\epsilon\Gamma} coh(e_i,e_j)\right] \quad (1)$$

其中Γ表示待匹配确定的实体空间集；

•其中$\phi(m_i,e_i)$是定义的兼容性函数，定义如下：

$\phi(m_i,e_i)=f(m,e)\times g(m,e)$　(2)

m表示商品文本中待链接的mention；e表示外部知识库中的实体entity；

➢f(m,e)是上下文无关的分数，主要与mention的字面意思与知识库中候选实体上下文之间的不相关的程度有关，定义如下：

$$f(m,e) = p(e|m)\times sim(m,e) + \frac{\beta}{|E_m|} \quad (3)$$

其中，p(e|m)表示商品文本中mention与外部知识库中entity的先验概率，从外部知识库中获取，具体为从链接到百度百科中的锚文本中统计估计得到；E_m是指从外部知识库中，可能与mention产生链接的实体集合；β表示加权概率，对可靠的实体集合E_m通过β来平衡前后的权重；sim(m,e)表示mention和entity的文本相似性，用于约束可能包含噪声的先验概率p(e|m)，sim(m,e)采用余弦相似度来刻画；

➢g(m,e)是上下文相关的分数，主要与mention的字面意思与知识库中候选实体上下文之间的相关的紧密程度有关，定义如下：

$g(m,e)=sim_t(m,e)\times(1-\in+\in\times sim_c(m,e))$　(4)

其中$sim_c(m,e)$表示m与e之间的余弦相似度；参数∈用于平衡和控制相关性得分的影响；$sim_t(m,e)$定义如下：

$$\begin{cases} sim_t(m,e) = max_{w_c\in CT(m),w_d\in KP(e)}\cos(V_w(w_c),V_w(w_d)) \\ V_c(m) = \sum_{w\in CT(m)}\frac{v_c(w)}{D(w,m)} \end{cases} \quad (5)$$

CT(m)表示商品名称上下文分词后的关键词集合；KP(e)表示可能的实体集合；$v_c(w)$表示词w的向量化表示形式；D(w,m)表示上下文词w与待链接的词m之间的距离函数，利用词之间的绝对距离来定义；

•$coh(e_i,e_j)$函数定义为上下文mention所确定的实体集，两两之间的相关性度量，

定义如下：

$coh(e_i, e_j) = \gamma \times rel(e_1, e_2) + (1-\gamma) \times sim(e_1, e_2)$　(6)

➢ $sim(e_1, e_2)$ 表示归一化Google距离的否定形式，用于衡量相似性：

$$sim(e_1,e_2)=1-\frac{\log(\max(|E_1|,|E_2|))-\log(|E_1\cap E_2|)}{\log(|E|)-\log(\min(|E_1|,|E_2|))} \quad (7)$$

其中，E_1和E_2分别是实体e_1和e_2从百度百科中获取的内联实体集合，E表示整个实体集合；|·|表示集合的个数；利用归一化Google距离的否定形式来比较实体集合之间隐含的实体的相似性；

➢ $rel(e_1, e_2)$ 用来进一步表示实体之间的关联性，定义如下：

$$\begin{cases} rel(e_1,e_2)=\max(rel'(e_1,e_2),rel'(e_2,e_1)) \\ rel'(e_1,e_2)=\sum_{r\in R(e_1,e_2)}\frac{2}{|T(e_1,r)|+|H(r,e_2)|} \end{cases} \quad (8)$$

其中$R(e_1, e_2)$表示实体e_1和e_2之间的关系集合；$T(e_1, r)$表示头部实体e_1和关系r的尾部实体集合；$H(r, e_2)$表示关系r和尾部实体e_2的头部实体集合；参数$\gamma \in [0,1]$用于权衡相似性和相关性的权重；

(3-2)将实体链接的结果利用Bert进行word embedding编码，得到相应的特征向量；

(4)将得到的特征向量喂入Transformer网络，利用self-attention机制，挖掘不同词对于税码分类的共享程度，赋予不同词不同的权重，最后通过Softmax对其进行分类，将概率最高的税码类别作为商品名称所属类别，最终确定待分类的商品名称的税码类别标签。

2.如权利要求1所述的一种基于注意力机制的商品名称短文本分类方法，其特征在于：所述步骤(2)包含以下具体实现过程：

(2-1)对预处理后的商品名称，利用jieba库进行中文分词，得到初步的分词结果；

(2-2)去除其中的停用词，得到若干个商品名称特征词；

(2-3)对得到的特征词进行短补长切，统一词的长度至事先设定的个数。

3.如权利要求1所述的一种基于注意力机制的商品名称短文本分类方法，其特征在于：所述步骤(4)包含以下具体实现过程：

(4-1)将得到的特征向量喂入Transformer网络，利用self-attention机制，挖掘不同词对于税码分类的共享程度，赋予不同词不同的权重；所述注意力机制公式如下:

$$Attention(Q,K,V)=softmax\left(\frac{QK^T}{\sqrt{d_k}}\right)V \quad (9)$$

其中，$\frac{1}{\sqrt{d_k}}$表示缩放因子，用于优化点积注意力的缺陷，将值缩放到softmax函数变化最大的区域，放大差距；同时在实践中，点击注意力机制计算更快，空间效率也更高，可以使用高度优化矩阵乘法来实现；

(4-2)通过Softmax对其进行分类，将概率最高的税码类别作为商品名称所属类别，最终确定待分类的商品名称的税码类别标签。

4.如权利要求1所述的一种基于注意力机制的商品名称短文本分类方法，其特征在于：其特征在于：步骤(3-1)中取Γ<100，即在外部知识库中寻找最相关的100个实体集合。

5.实施权利要求1所述的一种基于注意力机制的商品名称短文本分类方法的系统，其特征在于：包括：依次连接商品名称预处理模块、商品名称分词模块、特征语义扩展模块和商品名称分类模块；其中商品名称预处理模块向商品名称分词模块输出经过预处理后的商品名称，商品名称分词模块向特征语义扩展模块输出分词后的初始特征集合，特征语义扩展模块向商品名称分类模块输出扩展后的特征向量，最后商品名称分类模块输出商品名称对应的税码分类标签；

商品名称预处理模块，用于对商品名称进行预处理，只保留中文字段；具体包括：

(1-1)通过正则表达式，只保留中文字段；

商品名称分词模块，用于针对预处理后的商品名称，通过jieba分词工具进行分词，去除其中的停用词并统一词的个数；具体包括：

(2-1)对预处理后的商品名称，利用jieba库进行中文分词，得到初步的分词结果；

(2-2)去除其中的停用词，得到若干个商品名称特征词；

(2-3)对得到的特征词进行短补长切，统一词的长度至事先设定的个数；

特征语义扩展模块，用于解决短文本中上下文语义缺失的问题，通过外部知识库补充特征集合中词的语义信息，得到更高质量表达的特征向量；具体包括：

(3-1)将每个词利用Global Entity Linking算法进行实体消歧与链接，通过链接到百度百科的外部知识库，用其结果对短文本中的词扩充解释，丰富上下文语义信息，所述Global Entity Linking算法的公式如下：

$$T_g = argmax_{\Gamma}\left[\sum_{i=1}^{N}\phi(m_i,e_i) + \sum_{e_i\in\Gamma,e_j\in\Gamma} coh(e_i,e_j)\right] \quad (1)$$

其中Γ表示待匹配确定的实体空间集；

•其中$\phi(m_i,e_i)$是定义的兼容性函数，定义如下：

$\phi(m_i,e_i)=f(m,e)\times g(m,e)$　(2)

m表示商品文本中待链接的mention；e表示外部知识库中的实体entity；

➢f(m,e)是上下文无关的分数，主要与mention的字面意思与知识库中候选实体上下文之间的不相关的程度有关，定义如下：

$$f(m,e) = p(e|m)\times sim(m,e) + \frac{\beta}{|E_m|} \quad (3)$$

其中，p(e|m)表示商品文本中ment ion与外部知识库中entity的先验概率，从外部知识库中获取，具体为从链接到百度百科中的锚文本中统计估计得到；E_m是指从外部知识库中，可能与mention产生链接的实体集合；β表示加权概率，对可靠的实体集合E_m通过β来平衡前后的权重；sim(m,e)表示mention和entity的文本相似性，用于约束可能包含噪声的先验概率p(e|m)，sim(m,e)采用余弦相似度来刻画；

➢g(m,e)是上下文相关的分数，主要与mention的字面意思与知识库中候选实体上下文之间的相关的紧密程度有关，定义如下：

$g(m,e)=sim_t(m,e)\times(1-\in+\in\times sim_c(m,e))$　(4)

其中$sim_c(m,e)$表示m与e之间的余弦相似度；参数∈用于平衡和控制相关性得分的影响；$sim_t(m,e)$定义如下：

$$\begin{cases} sim_t(m,e) = max_{w_c \in CT(m), w_d \in KP(e)} \cos\left(V_w(w_c), V_w(w_d)\right) \\ V_c(m) = \sum_{w \in CT(m)} \frac{v_c(w)}{D(w,m)} \end{cases} \quad (5)$$

CT(m)表示商品名称上下文分词后的关键词集合;KP(e)表示可能的实体集合;v_c(w)表示词w的向量化表示形式;D(w,m)表示上下文词w与待链接的词m之间的距离函数,利用词之间的绝对距离来定义;

• coh(e_i,e_j)函数定义为上下文mention所确定的实体集,两两之间的相关性度量,定义如下:

coh(e_i,e_j)=γ×rel(e_1,e_2)+(1-γ)×sim(e_1,e_2) (6)

➢sim(e_1,e_2)表示归一化Google距离的否定形式,用于衡量相似性:

$$sim(e_1,e_2) = 1 - \frac{\log(\max(|E_1|,|E_2|)) - \log(|E_1 \cap E_2|)}{\log(|E|) - \log(\min(|E_1|,|E_2|))} \quad (7)$$

其中,E_1和E_2分别是实体e_1和e_2从百度百科中获取的内联实体集合,E表示整个实体集合;|•|表示集合的个数;利用归一化Google距离的否定形式来比较实体集合之间隐含的实体的相似性;

➢rel(e_1,e_2)用来进一步表示实体之间的关联性,定义如下:

$$\begin{cases} rel(e_1,e_2) = \max(rel'(e_1,e_2), rel'(e_2,e_1)) \\ rel'(e_1,e_2) = \sum_{r \in R(e_1,e_2)} \frac{2}{|T(e_1,r)| + |H(r,e_2)|} \end{cases} \quad (8)$$

其中R(e_1,e_2)表示实体e_1和e_2之间的关系集合;T(e_1,r)表示头部实体e_1和关系r的尾部实体集合;H(r,e_2)表示关系r和尾部实体e_2的头部实体集合;参数γ∈[0,1]用于权衡相似性和相关性的权重;

(3-2)将实体链接的结果利用Bert进行word embedding编码,得到相应的特征向量;

商品名称分类模块,用于对商品名称通过Transformer网络架构进行最终的分类,将概率最高的税码类别作为商品名称所属类别,最终确定待分类的商品名称的税码类别标签;具体包括:

(4-1)将得到的特征向量喂入Transformer网络,利用self-attention机制,挖掘不同词对于税码分类的共享程度,赋予不同词不同的权重;所述注意力机制公式如下:

$$Attention(Q,K,V) = softmax\left(\frac{QK^T}{\sqrt{d_k}}\right)V \quad (9)$$

其中,$\frac{1}{\sqrt{d_k}}$表示缩放因子,用于优化点积注意力的缺陷,将值缩放到softmax函数变化最大的区域,放大差距;同时在实践中,点击注意力机制计算更快,空间效率也更高,可以使用高度优化矩阵乘法来实现;

(4-2)通过Softmax对其进行分类,将概率最高的税码类别作为商品名称所属类别,最终确定待分类的商品名称的税码类别标签。

6.如权利要求5所述的系统,其特征在于:其特征在于:取Γ<100,即在外部知识库中寻

找最相关的100个实体集合。

一种基于注意力机制的商品名称短文本分类方法和系统

技术领域

[0001] 本发明涉及一种基于注意力机制的商品名称短文本分类方法和系统，特别是针对商品名称对应税码的分类。利用中文文本分词工具对文本进行分词，将每个商品名称的词进行短补长切调整统一的词个数后，利用Bert对每个词进行word embedding得到对应的词向量，将词向量喂入Transformer后，利用注意力机制，得到各个词的权重信息，最后通过Softmax进行分类。本发明涉及概率模型，语音模型，深度学习等领域，具体涉及基于深度学习的建模领域。

背景技术

[0002] 随着社会的不断发展，税码分类体系越来越复杂，如何将海量的商品名称准确地划分到对应的税码分类是一种重要的技术需求。越来越多的企业需要开商品发票，由于交易量的剧增，人工进行税码分类的方法存在效率不高、成本巨大且分类结果受到的人偶然性错误的影响，同时对税码不熟悉的人也难以完成我国高达4000多种的税码分类。因此，利用程序解决繁重的分类问题才是当下的趋势。

[0003] 商品名称的税码分类存在以下几个问题：

[0004] (1)商品名称的记录大多由人工完成，导致了商品名称中大多包含很多噪声，需要滤除其中的噪声，以减少对税码分类的影响。

[0005] (2)商品名称大多是短文本类型，仅有几个词组成，无法有效的提取上下文信息，也导致目前主流的自然语言处理的方法在此问题上受限。

[0006] (3)在我国，五级税码分类高达4000余种，种类繁多，属于超分类难题，目前很难有有效的方法解决。

[0007] 目前税码分类的方法大致有两种：

[0008] 1)关键词匹配，即构造一个税码到关键词的1:N的映射关系，然后在给定的商品名称中匹配相应的关键词，即可完成税码分类任务。

[0009] 2)基于机器学习的分类方法，即将税码匹配的问题转化为一个分类问题，利用机器学习或者深度学习的模型予以解决。

[0010] 关键词匹配的方法，较为简单，但是严重依赖于关键词库的大小以及准确度，只能匹配到关键词库中已经存在的一些商品，而且关键词必须显式的存在与商品名称中。如果商品名称中不包含词库中的关键词，则匹配失效。虽然可以通过一些算法解决关键词分隔的问题，比如关键词是“小麦面粉”，而商品名称是“东北小麦优质面粉”的情况。但是无法解决若干个关键词同时出现在商品名称中的情况，目前一些做法是在匹配得到的关键词中取较长的作为最终的关键词，但是该策略无法解决全部等长的关键词，比如商品名称“钢笔，附赠小瓶墨水”，商品的主体是“钢笔”，但关键词库中也会同时匹配得到“墨水”，关键词匹配的算法无法区分两者的权重，也无法确定按哪个关键词匹配对应的税码。另外，该方法的泛化能力较差，因为不同公司在对商品名称进行税码匹配时，大都嵌入到专业的领域，比如电力行业，一些专有名词较多，普通人甚至从未听说过，此时就需要重新构造或添加关键词

库,费时费力。因此该方法在大型且覆盖面较广的数据集上表现并不好。

[0011] 基于机器学习的分类方法,按训练样本的特性可以分为有监督分类和无监督分类方法。基于无监督的分类方法,包括语言分析法、统计方法、主题方法和基于网络图的方法,主要是通过聚类来解决分类问题。文本分析中对无监督算法特征的刻画有以下几种:比较经典的TFIDF方法考虑了词的频率和逆文档频率;TextRank考虑了词的共现信息; Rake方法考虑了词的共现矩阵中词的度的信息和词频信息。尽管目前有很多学者基于此做了很多研究和改进,但是仍如无法避免特征工程的局限性:1、短文本特征刻画考虑不全面,有的考虑了词的频率忽略了词性、位置信息,有的考虑了词的共现信息忽略了文本结构信息等,影响了关键词提取的准确率。2、短本文评分机制过于主观化,以人的先验知识作为评分规则的解释标准或是根本就没有说明评分规则设定的依据。因此无监督的方法在税码分类问题上往往表现很差,因此需要考虑有监督的分类算法。

[0012] 基于有监督的分类方法的一般步骤是:首先,建立一个包含大量文本并标出商品名称对应的税码分类的训练集;然后,利用训练集合对分类模型进行训练;最后,应用训练好的模型对新的商品名称进行分类。该方法的实用性较强,如果采用合适的模型,设定合适的参数可以得到较好的结果。目前主流的自然语言问题上,大多都运用了深度学习的方法。深度学习具有自动挖掘文本中特征的性质,可以从数据中获取相应的文本表示,从而避免的繁重的特征工程。同时深度学习通过网络层数的设计与堆叠可以得到复杂的模型,满足复杂的任务需求。但是一般的机器学习或者深度学习的模型难以在税码分类问题上达到较好的效果,因为商品名称大多是有一些词构成的超短文本,严重缺乏上下文信息,存在稀疏性和噪声的问题,一般的深度学习网络比如LSTM在该问题上表现较差,因为这类模型都是基于对上下文信息的挖掘,从而完成特征的提取。因此如何有效的解决超短文本上下文语义缺乏的问题,显得格外重要。

[0013] 在目前,很多企业都需要对自己企业的商品买卖记录进行统一的管理,根据记录的商品开商品发票,或者获取商品相应的税率,确定相应的税收开支。而一些企业一年的商品记录可以达到上百万甚至上千万条,此时仅仅依靠人工进行相应的税码分类显得很不现实、主观性较强,难以保证效率以及准确率,同时税码分类需要一些专业的税务人员才能完成,导致成本的进一步增加,一般企业难以承担其成本。同时税码分类面临着很多难点:一方面商品名称的登记过程,可能记录了一些误导性的噪声,一些核心的关键词并不是我们所需要的有帮助的信息;另一方面,商品名称大多数仅有几个词组成,严重缺乏上下文语义,一般的分类算法根本难以解决短文本的问题,进一步增加了分类的难度。目前人工标记税码的方法既主观同时又是劳动密集型的方法,所以采用有效的分类算法解决该问题具有很好的应用前景。

[0014] 因此,如何解决商品名称的短文本上下文缺失的问题,快速、准确的完成税码的分类和匹配,成为技术人员急待解决的难题。

发明内容

[0015] 本发明要克服现有的商品名称的税码分类技术中的上述缺点与不足,提供了一种基于注意力机制的商品名称短文本分类方法和系统,实现对待不同商品名称所属税码分类的自动及精确判断。

[0016] 为了实现上述发明目的,本发明提供了以下技术方案:

[0017] 本发明提供了一种基于注意力机制的商品名称短文本分类方法,包含以下步骤:

[0018] (1)对商品名称进行预处理,只保留中文字段;

[0019] (2)通过jieba分词,将预处理后的商品短文本分成若干个词,去除停用词,对得到的词进行短补长切,统一词的长度到事先设定好的词个数;

[0020] (3)将每个词利用Global Entity Linking算法进行实体消歧与链接,通过链接到百度百科的外部知识库,用其结果对短文本中的词扩充解释,丰富上下文语义信息,并将实体链接的结果利用Bert进行word embedding编码,得到相应的特征向量;

[0021] (4)将得到的向量喂入Transformer网络,利用self-attention机制,挖掘不同词对于税码分类的共享程度,赋予不同词的不同权重,最后通过Softmax对其进行分类,将概率最高的税码类别作为商品名称所属类别,最终确定待分类的商品名称的税码类别标签。

[0022] 优选的,所述步骤(2)包含以下实现过程:

[0023] (2-1)对预处理后的商品名称,利用jieba库进行中文分词,得到初步的分词结果;

[0024] (2-2)去除其中的停用词,得到若干个商品名称特征词;

[0025] (2-3)对得到的特征词进行短补长切,统一词的长度至事先设定的个数;

[0026] 优选的,所述步骤(3)包含以下实现过程:

[0027] (3-1)将每个词利用Global Entity Linking算法进行实体消歧与链接,通过链接到百度百科的外部知识库,用其结果对短文本中的词扩充解释,丰富上下文语义信息,所述Global Entity Linking算法的公式如下:

[0028] $$T_g = argmax_{\Gamma}\left[\sum_{i=1}^{N}\phi(m_i,e_i) + \sum_{e_i\in\Gamma,e_j\in\Gamma} coh(e_i,e_j)\right] \quad (1)$$

[0029] 其中Γ表示待匹配确定的实体空间集。

[0030] •其中$\phi(m_i,e_i)$是定义的兼容性函数,定义如下:

[0031] $\phi(m_i,e_i)=f(m,e)\times g(m,e)$ (2)

[0032] m表示商品文本中待链接的mention;e表示外部知识库中的实体entity;

[0033] ➢f(m,e)是上下文无关的分数,主要与mention的字面意思与知识库中候选实体上下文之间的不相关的程度有关,定义如下:

[0034] $$f(m,e) = p(e|m)\times sim(m,e) + \frac{\beta}{|E_m|} \quad (3)$$

[0035] 其中,p(e|m)表示商品文本中mention与外部知识库中entity的先验概率,通常从外部知识库中获取,在本文中是从链接到百度百科中的锚文本中统计估计得到;E_m是指从外部知识库中,可能与mention产生链接的实体集合;β表示加权概率,对可靠的实体集合E_m通过β来平衡前后的权重;sim(m,e)表示mention和entity的文本相似性,用于约束可能包含噪声的先验概率p(e|m),在本文中sim(m,e)采用余弦相似度来刻画。

[0036] ➢g(m,e)是上下文相关的分数,主要与mention的字面意思与知识库中候选实体上下文之间的相关的紧密程度有关,定义如下:

[0037] $g(m,e)=sim_t(m,e)\times(1-\in+\in\times sim_c(m,e))$ (4)

[0038] 其中$sim_c(m,e)$表示m与e之间的余弦相似度;参数∈用于平衡和控制相关性得分

的影响；$sim_t(m,e)$定义如下：

[0039]
$$\begin{cases} sim_t(m,e) = max_{w_c \in CT(m), w_d \in KP(e)} \cos(V_w(w_c), V_w(w_d)) \\ V_c(m) = \sum_{w \in CT(m)} \frac{v_c(w)}{D(w,m)} \end{cases} \quad (5)$$

[0040] CT(m)表示商品名称上下文分词后的关键词集合；KP(e)表示可能的实体集合； $v_c(w)$表示词w的向量化表示形式；D(w,m)表示上下文词w与待链接的词m之间的距离函数，利用词之间的绝对距离来定义；

[0041] •$coh(e_i,e_j)$函数定义为上下文mention所确定的实体集，两两之间的相关性度量，

[0042] 定义如下：

[0043] $coh(e_i,e_j) = \gamma \times rel(e_1,e_2) + (1-\gamma) \times sim(e_1,e_2)$ (6)

[0044] ➢$sim(e_1,e_2)$表示归一化Google距离的否定形式，用于衡量相似性：

[0045]
$$sim(e_1,e_2) = 1 - \frac{\log(\max(|E_1|,|E_2|)) - \log(|E_1 \cap E_2|)}{\log(|E|) - \log(\min(|E_1|,|E_2|))} \quad (7)$$

[0046] 其中，E_1和E_2分别是实体e_1和e_2从百度百科中获取的内联实体集合，E表示整个实体集合；|•|表示集合的个数；利用归一化Google距离的否定形式来比较实体集合之间隐含的实体的相似性。

[0047] ➢$rel(e_1,e_2)$用来进一步表示实体之间的关联性，定义如下：

[0048]
$$\begin{cases} rel(e_1,e_2) = \max(rel'(e_1,e_2), rel'(e_2,e_1)) \\ rel'(e_1,e_2) = \sum_{r \in R(e_1,e_2)} \frac{2}{|T(e_1,r)| + |H(r,e_2)|} \end{cases} \quad (8)$$

[0049] 其中$R(e_1,e_2)$表示实体e_1和e_2之间的关系集合；$T(e_1,r)$表示头部实体e_1和关系r的尾部实体集合；$H(r,e_2)$表示关系r和尾部实体e_2的头部实体集合；参数$\gamma \in [0,1]$用于权衡相似性和相关性的权重；

[0050] (3-2)将实体链接的结果利用Bert进行word embedding编码，得到相应的特征向量；

[0051] 优选的，所述步骤(4)包含以下实现过程：

[0052] (4-1)将得到的特征向量喂入Transformer网络，利用self-attention机制，挖掘不同词对于税码分类的共享程度，赋予不同词的不同权重；所述注意力机制公式如下：

[0053]
$$Attention(Q,K,V) = softmax\left(\frac{QK^T}{\sqrt{d_k}}\right)V \quad (9)$$

[0054] 其中，$\frac{1}{\sqrt{d_k}}$表示缩放因子，用于优化点积注意力的缺陷，将值缩放到softmax函数变化最大的区域，放大差距。同时在实践中，点击注意力机制计算更快，空间效率也更高，可以使用高度优化矩阵乘法来实现；

[0055] (4-2)通过Softmax对其进行分类，将概率最高的税码类别作为商品名称所属类别，最终确定待分类的商品名称的税码类别标签。

[0056] 进一步,步骤(3-1)中取$\Gamma<100$,即在通过外部知识库中可能相关的100个实体集合。

[0057] 此外,本发明还提供了实施前述的一种基于注意力机制的商品名称短文本分类方法的系统,所述系统包括:

[0058] 商品名称预处理模块,用于对商品名称进行预处理,只保留中文字段;具体包括:

[0059] (1-1)通过正则表达式,只保留中文字段;

[0060] 商品名称分词模块,用于针对预处理后的商品名称,通过jieba分词工具进行分词,去除其中的停用词并统一词的个数;具体包括:

[0061] (2-1)对预处理后的商品名称,利用jieba库进行中文分词,得到初步的分词结果;

[0062] (2-2)去除其中的停用词,得到若干个商品名称特征词;

[0063] (2-3)对得到的特征词进行短补长切,统一词的长度至事先设定的个数;

[0064] 特征语义扩展模块,用于解决短文本中上下文语义缺失的问题,通过外部知识库补充特征集合中词的语义信息,得到更高质量表达的特征向量;具体包括:

[0065] (3-1)将每个词利用Global Entity Linking算法进行实体消歧与链接,通过链接到百度百科的外部知识库,用其结果对短文本中的词扩充解释,丰富上下文语义信息,所述Global Entity Linking算法的公式如下:

[0066] $$T_g = argmax_{\Gamma}\left[\sum_{i=1}^{N}\phi(m_i,e_i) + \sum_{e_i\in\Gamma,e_j\in\Gamma} coh(e_i,e_j)\right] \quad (1)$$

[0067] 其中Γ表示待匹配确定的实体空间集。

[0068] ·其中$\phi(m_i,e_i)$是定义的兼容性函数,定义如下:

[0069] $\phi(m_i,e_i)=f(m,e)\times g(m,e)$ (2)

[0070] m表示商品文本中待链接的mention;e表示外部知识库中的实体entity;

[0071] ➢f(m,e)是上下文无关的分数,主要与mention的字面意思与知识库中候选实体上下文之间的不相关的程度有关,定义如下:

[0072] $$f(m,e) = p(e|m)\times sim(m,e) + \frac{\beta}{|E_m|} \quad (3)$$

[0073] 其中,p(e|m)表示商品文本中mention与外部知识库中entity的先验概率,通常从外部知识库中获取,在本文中是从链接到百度百科中的锚文本中统计估计得到;E_m是指从外部知识库中,可能与mention产生链接的实体集合;β表示加权概率,对可靠的实体集合E_m通过β来平衡前后的权重;sim(m,e)表示mention和entity的文本相似性,用于约束可能包含噪声的先验概率p(e|m),在本文中sim(m,e)采用余弦相似度来刻画。

[0074] ➢g(m,e)是上下文相关的分数,主要与mention的字面意思与知识库中候选实体上下文之间的相关的紧密程度有关,定义如下:

[0075] $g(m,e)=sim_t(m,e)\times(1-\in+\in\times sim_c(m,e))$ (4)

[0076] 其中$sim_c(m,e)$表示m与e之间的余弦相似度;参数∈用于平衡和控制相关性得分的影响;$sim_t(m,e)$定义如下:

[0077] $$\begin{cases} sim_t(m,e) = max_{w_c \in CT(m), w_d \in KP(e)} \cos\left(V_w(w_c), V_w(w_d)\right) \\ V_c(m) = \sum_{w \in CT(m)} \frac{v_c(w)}{D(w,m)} \end{cases} \quad (5)$$

[0078] CT(m)表示商品名称上下文分词后的关键词集合;KP(e)表示可能的实体集合；$v_c(w)$表示词w的向量化表示形式;D(w,m)表示上下文词w与待链接的词m之间的距离函数,利用词之间的绝对距离来定义义;

[0079] •coh(e_i,e_j)函数定义为上下文mention所确定的实体集,两两之间的相关性度量,

[0080] 定义如下:

[0081] coh(e_i,e_j)＝γ×rel(e_1,e_2)+(1-γ)×sim(e_1,e_2) (6)

[0082] ➢sim(e_1,e_2)表示归一化Google距离的否定形式,用于衡量相似性:

[0083] $$sim(e_1,e_2) = 1 - \frac{\log(\max(|E_1|,|E_2|)) - \log(|E_1 \cap E_2|)}{\log(|E|) - \log(\min(|E_1|,|E_2|))} \quad (7)$$

[0084] 其中,E_1和E_2分别是实体e_1和e_2从百度百科中获取的内联实体集合,E表示整个实体集合;|•|表示集合的个数;利用归一化Google距离的否定形式来比较实体集合之间隐含的实体的相似性。

[0085] ➢rel(e_1,e_2)用来进一步表示实体之间的关联性,定义如下:

[0086] $$\begin{cases} rel(e_1,e_2) = \max\left(rel'(e_1,e_2), rel'(e_2,e_1)\right) \\ rel'(e_1,e_2) = \sum_{r \in R(e_1,e_2)} \frac{2}{|T(e_1,r)| + |H(r,e_2)|} \end{cases} \quad (8)$$

[0087] 其中R(e_1,e_2)表示实体e_1和e_2之间的关系集合;T(e_1,r)表示头部实体e_1和关系r的尾部实体集合;H(r,e_2)表示关系r和尾部实体e_2的头部实体集合;参数γ∈[0,1]用于权衡相似性和相关性的权重;

[0088] (3-2)将实体链接的结果利用Bert进行word embedding编码,得到相应的特征向量;

[0089] 商品名称分类模块,用于对商品名称通过Transformer网络架构进行最终的分类,将概率最高的税码类别作为商品名称所属类别,最终确定待分类的商品名称的税码类别标签;具体包括:

[0090] (4-1)将得到的特征向量喂入Transformer网络,利用self-attention机制,挖掘不同词对于税码分类的共享程度,赋予不同词的不同权重;所述注意力机制公式如下:

[0091] $$Attention(Q,K,V) = softmax\left(\frac{QK^T}{\sqrt{d_k}}\right)V \quad (9)$$

[0092] 其中,$\frac{1}{\sqrt{d_k}}$表示缩放因子,用于优化点积注意力的缺陷,将值缩放到softmax函数变化最大的区域,放大差距。同时在实践中,点击注意力机制计算更快,空间效率也更高,可以使用高度优化矩阵乘法来实现;

[0093] (4-2)通过Softmax对其进行分类,将概率最高的税码类别作为商品名称所属类

别,最终确定待分类的商品名称的税码类别标签;

[0094] 优选地,取Γ<100,即在通过外部知识库中可能相关的100个实体集合。

[0095] 上述商品名称分类系统,通过商品名称预处理模块只保留初始商品名称中的中文字段;然后通过商品名称分词模块,利用分词技术得到一系列特征词集合,统一集合中词的个数,构成初始特征集合;再通过特征语义扩展模块,用于解决短文本中上下文语义缺失的问题,通过外部知识库补充特征集合中词的语义信息,得到更高质量表达的特征向量;最后通过商品名称分类模块,用于对商品名称通过Transformer网络架构进行最终的分类,将概率最高的税码类别作为商品名称所属类别,最终确定待分类的商品名称的税码类别标签。本发明针对模拟专业人员在进行税码分类时,抓取核心词进行判断的行为特点,通过实体链接技术,引入外部知识库比如百度百科、维基百科中对一些专业性较强的关键词的解释,补充了短文本中上下文严重缺失的问题,并且结合深度学习中注意力机制,通过训练的方法得到不同词之间对于正确税码分类的重要程度,并从而完成相应的分类,可以有效的抓取商品名称中的关键信息,相较于现有的方法在准确度以及效率上有显著的提升。

[0096] 本发明与现有技术相比,具有如下优点和效益:

[0097] 1、上述商品名称税码分类方法,利用分词技术提取特征词集合,并利用实体链接技术补充短文本语义,并引入Transformer中的注意力机制,对商品名称进行税码类别划分,解决了人工分类效率低、成本高的问题。

[0098] 2、基于实体链接进行短文本语义补充的方法,可以很好的解决短文本分类过程中,由于短文本的语义严重缺失造成的问题,提高了分类的准确率。

[0099] 3、基于注意力机制的分类方法,可以很好的关注贡献率更高的核心特征词,对不同的特征词根据其贡献率赋予不同的权重,从而提高了神经网络分类过程中的准确率。

附图说明

[0100] 图1为本发明方法的实现过程示意图。

[0101] 图2为本发明方法的步骤(2)的具体实现过程示意图。

[0102] 图3为本发明方法的步骤(3)的具体实现过程示意图。

[0103] 图4为本发明方法的步骤(4)的具体实现过程示意图。

[0104] 图5为本发明的系统的结构示意图。

具体实施方式

[0105] 为了能够更清楚地理解本发明地上述目的、特征和优点,下面结合附图和具体实施方式对本发明进行进一步地详细说明。

[0106] 为了解决现有的商品名称的税码分类问题,针对现阶段税码分类算法存在的种种不足,本发明提出了一种基于注意力机制的商品名称超短文本分类方法,可以全面的考虑到专业人员在进行税码分类时,抓取核心词进行判断的行为,并且结合深度学习中注意力机制,通过训练的方法得到不同词之间对于正确税码分类的重要程度,避免了人在进行税码分类时存在的主观性,并结合entity linking方法,引入外部知识库比如百度百科、维基百科中对一些专业性较强的关键词的解释,补充了短文本中上下文严重缺失的问题,从而完成相应的分类,可以有效的抓取商品名称中的关键信息,相较于现有的方法在准确度以

及效率上有一定的提升。

[0107] 本发明提供了一种基于注意力机制的商品名称短文本分类方法，根据外部知识库补充短文本信息，引入注意力机制，对不同特征词赋予不同地权重，从而更好地实现商品名称的税码分类问题。

[0108] 为了实现上述发明目的，本发明提供了如图1所示的以下技术方案：

[0109] 步骤1：通过对商品名称短文本数据进行预处理，只保留中文字段；

[0110] 原始数据中包含大量的无效信息，如果直接进行特征提取会对分类正确率产生很大的干扰，因此在对数据进行特征提取前先进行数据预处理只保留其中的中文字段。

[0111] 步骤2：对预处理后的商品名称进行分词、去停用词和统一词个数；

[0112] 通过jieba分词技术，将预处理后的商品短文本分成若干个词，去除停用词，对得到的词进行短补长切，统一词的长度到事先设定好的词个数，得到代表商品名称的核心词集合；

[0113] 具体的，步骤2包含如图2所示的实现过程：

[0114] (2-1)对预处理后的短文本进行分词处理。利用jieba分词工具对短文本进行分词处理，例如，对“35kV及以下电缆终端150mm2/3芯/户内终端冷缩铜/AC35kV”进行预处理后得到：“及以下电缆终端芯户内终端冷缩铜”，再通过分词处理后得到：“及以下 /电缆终端/芯/户内/终端/冷缩铜”；

[0115] (2-2)去除停用词。去除那些对分类作用不大甚至负面作用的词语。通过停用词表剔除无用词后，得到以下特征词“电缆终端、户内、终端、冷缩铜”；

[0116] (2-3)对特征词进行短补长切，统一输入的长度；

[0117] 步骤3：利用外部知识库对处理后的数据进行实体消歧和链接，扩充上下文语义信息；

[0118] 由于商品名称大多是短文本，极度缺乏上下文信息，而现有的深度学习分类方法大多依靠上下文信息，因此如果不进行语义补充将极大的影响准确率。同时存在一些领域专有词汇，比如“绝缘穿刺接地环”、“锚定线夹”、“铜接线耳”等等词汇，将每个词利用Global Entity Linking算法进行实体消歧与链接，通过链接到百度百科的外部知识库，用其结果对短文本中的词扩充解释，丰富上下文语义信息，将实体链接得到的锚文本进行编码，替换在商品名称中词的编码，从而提高网络模型的表达能力，有效解决在短文本分类中的上下文缺乏的问题；

[0119] 具体的，步骤3包含如图3所示的实现过程：

[0120] (3-1)将每个词利用Global Entity Linking算法进行实体消歧与链接，通过链接到百度百科的外部知识库，用其结果对短文本中的词扩充解释，丰富上下文语义信息，所述Global Entity Linking算法的公式如下：

[0121] $$T_g = argmax_\Gamma\left[\sum_{i=1}^{N}\phi(m_i,e_i)+\sum_{e_i\in\Gamma,e_j\in\Gamma}coh(e_i,e_j)\right] \quad (1)$$

[0122] 其中Γ表示待匹配确定的实体空间集。

[0123] •其中$\phi(m_i,e_i)$是定义的兼容性函数，定义如下：

[0124] $\phi(m_i,e_i)=f(m,e)\times g(m,e)$ (2)

[0125] m表示商品文本中待链接的mention;e表示外部知识库中的实体entity;

[0126] ➢f(m,e)是上下文无关的分数,主要与mention的字面意思与知识库中候选实体上下文之间的不相关的程度有关,定义如下:

[0127] $$f(m,e)=p(e|m)\times sim(m,e)+\frac{\beta}{|E_m|} \quad (3)$$

[0128] 其中,p(e|m)表示商品文本中mention与外部知识库中entity的先验概率,通常从外部知识库中获取,在本文中是从链接到百度百科中的锚文本中统计估计得到;E_m是指从外部知识库中,可能与mention产生链接的实体集合;β表示加权概率,对可靠的实体集合E_m通过β来平衡前后的权重;sim(m,e)表示mention和entity的文本相似性,用于约束可能包含噪声的先验概率p(e|m),在本文中sim(m,e)采用余弦相似度来刻画。

[0129] ➢g(m,e)是上下文相关的分数,主要与mention的字面意思与知识库中候选实体上下文之间的相关的紧密程度有关,定义如下:

[0130] $g(m,e)=sim_t(m,e)\times(1-\in+\in\times sim_c(m,e))$ (4)

[0131] 其中$sim_c(m,e)$表示m与e之间的余弦相似度;参数∈用于平衡和控制相关性得分的影响;$sim_t(m,e)$定义如下:

[0132] $$\begin{cases} sim_t(m,e)=max_{w_c\in CT(m),w_d\in KP(e)}\cos\left(V_w(w_c),V_w(w_d)\right) \\ V_c(m)=\sum_{w\in CT(m)}\frac{v_c(w)}{D(w,m)} \end{cases} \quad (5)$$

[0133] CT(m)表示商品名称上下文分词后的关键词集合;KP(e)表示可能的实体集合;$v_c(w)$表示词w的向量化表示形式;D(w,m)表示上下文词w与待链接的词m之间的距离函数,利用词之间的绝对距离来定义;

[0134] •coh(e_i,e_j)函数定义为上下文mention所确定的实体集,两两之间的相关性度量,

[0135] 定义如下:

[0136] $coh(e_i,e_j)=\gamma\times rel(e_1,e_2)+(1-\gamma)\times sim(e_1,e_2)$ (6)

[0137] ➢sim(e_1,e_2)表示归一化Google距离的否定形式,用于衡量相似性:

[0138] $$sim(e_1,e_2)=1-\frac{\log(\max(|E_1|,|E_2|))-\log(|E_1\cap E_2|)}{\log(|E|)-\log(\min(|E_1|,|E_2|))} \quad (7)$$

[0139] 其中,E_1和E_2分别是实体e_1和e_2从百度百科中获取的内联实体集合,E表示整个实体集合;|•|表示集合的个数;利用归一化Google距离的否定形式来比较实体集合之间隐含的实体的相似性。

[0140] ➢rel(e_1,e_2)用来进一步表示实体之间的关联性,定义如下:

[0141] $$\begin{cases} rel(e_1,e_2)=\max\left(rel'(e_1,e_2),rel'(e_2,e_1)\right) \\ rel'(e_1,e_2)=\sum_{r\in R(e_1,e_2)}\frac{2}{|T(e_1,r)|+|H(r,e_2)|} \end{cases} \quad (8)$$

[0142] 其中R(e_1,e_2)表示实体e_1和e_2之间的关系集合;T(e_1,r)表示头部实体e_1和关系r的尾部实体集合;H(r,e_2)表示关系r和尾部实体e_2的头部实体集合;参数γ∈[0,1]用于权衡

相似性和相关性的权重。

[0143] (3-2)将实体链接的结果利用Bert进行word embedding编码,得到相应的特征向量;

[0144] 步骤4:将得到的向量喂入Transformer网络,利用self-attention机制,挖掘不同词对于税码分类的共享程度,赋予不同词的不同权重,最后通过Softmax对其进行分类,将概率最高的税码类别作为商品名称所属类别,最终确定待分类的商品名称的税码类别标签;

[0145] 得到补充语义信息的文本输入后,利用预训练模型Bert进行编码,然后利用self-attention机制,挖掘不同词对于税码分类的共享程度,赋予不同词的不同权重,最后通过Softmax对其进行分类,将概率最高的税码类别作为商品名称所属类别,最终确定待分类的商品名称的税码类别标签。

[0146] 具体的,步骤4包含如图4所示的实现过程:

[0147] (4-1)将得到的特征向量喂入Transformer网络,利用self-attention机制,挖掘不同词对于税码分类的共享程度,赋予不同词的不同权重;所述注意力机制公式如下:

[0148] $$Attention(Q,K,V)=softmax\left(\frac{QK^T}{\sqrt{d_k}}\right)V \quad (9)$$

[0149] 其中,$\frac{1}{\sqrt{d_k}}$表示缩放因子,用于优化点积注意力的缺陷,将值缩放到softmax函数变化最大的区域,放大差距。同时在实践中,点击注意力机制计算更快,空间效率也更高,可以使用高度优化矩阵乘法来实现;

[0150] (4-2)通过Softmax对其进行分类,将概率最高的税码类别作为商品名称所属类别,最终确定待分类的商品名称的税码类别标签。

[0151] 为了实施上述基于注意力机制的商品名称短文本分类方法,本发明还提供了一种基于注意力机制的商品名称短文本分类系统,如图5所示,主要包括:依次连接商品名称预处理模块、商品名称分词模块、特征语义扩展模块和商品名称分类模块;其中商品名称预处理模块向商品名称分词模块输出经过预处理后的商品名称,商品名称分词模块向特征语义扩展模块输出分词后的初始特征集合,特征语义扩展模块向商品名称分类模块输出扩展后的特征向量,最后商品名称分类模块输出商品名称对应的税码分类标签;

[0152] 商品名称预处理模块,用于对商品名称进行预处理,只保留中文字段;

[0153] 商品名称分词模块,用于针对预处理后的商品名称,通过jieba分词工具进行分词,去除其中的停用词并统一词的个数;

[0154] 特征语义扩展模块,用于解决短文本中上下文语义缺失的问题,通过外部知识库补充特征集合中词的语义信息,得到更高质量表达的特征向量;

[0155] 商品名称分类模块,用于对商品名称通过Transformer网络架构进行最终的分类,将概率最高的税码类别作为商品名称所属类别,最终确定待分类的商品名称的税码类别标签;

[0156] 上述商品名称短文本分类系统,通过商品名称预处理模块只保留初始商品名称中的中文字段;然后通过商品名称分词模块,利用分词技术得到一系列特征词集合,统一集合中词的个数,构成初始特征集合;再通过特征语义扩展模块,用于解决短文本中上下文语义

缺失的问题,通过外部知识库补充特征集合中词的语义信息,得到更高质量表达的特征向量;最后通过商品名称分类模块,用于对商品名称通过Transformer网络架构进行最终的分类,可以有效的抓取商品名称中的关键信息,将概率最高的税码类别作为商品名称所属类别,最终确定待分类的商品名称的税码类别标签,相较于现有的方法在准确度以及效率上有显著的提升,极大的降低了人工成本。

[0157] 本发明已经通过上述实例进行了说明,但应当注意的是实例只是解释说明的目的,而非将本发明局限于该实例范围内。尽管参照前述实例本发明进行了详尽的说明,本领域研究人员应当能够理解:其依然可以随前述各实例所记载的技术方案进行修改,或者对其部分技术特征进行同等替换;二、这些修改或替换,并不使相应的技术方案脱离本发明的保护范围。本发明的保护范围由附属的权利 要求书及其 等效范围所界定。

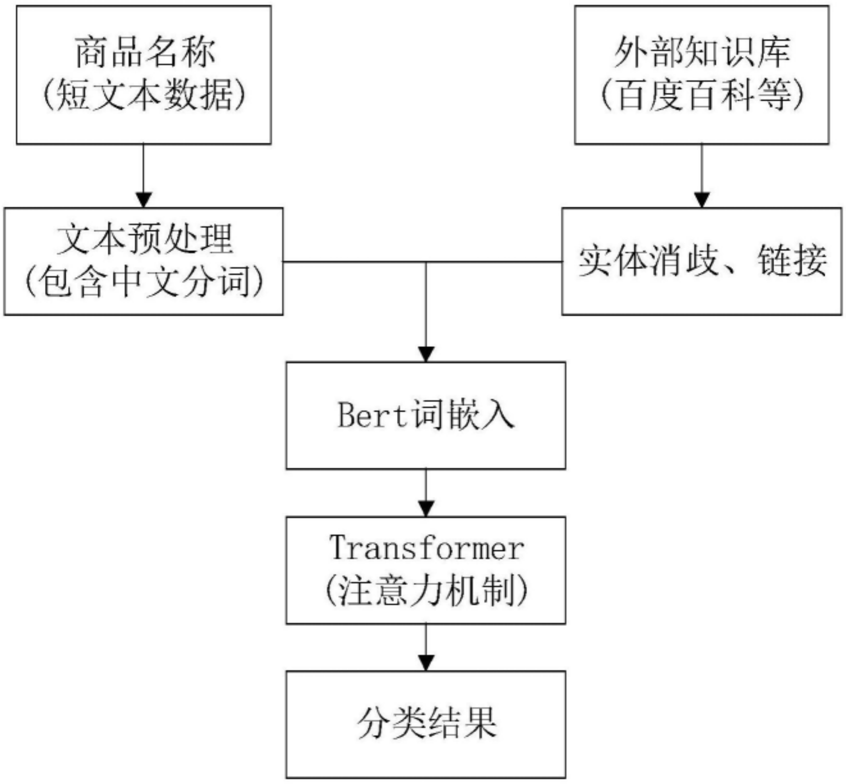
商品名称
(短文本数据)
外部知识库
(百度百科等)
文本预处理
(包含中文分词)
实体消歧、链接
Bert词嵌入
Transformer
(注意力机制)
分类结果

图1

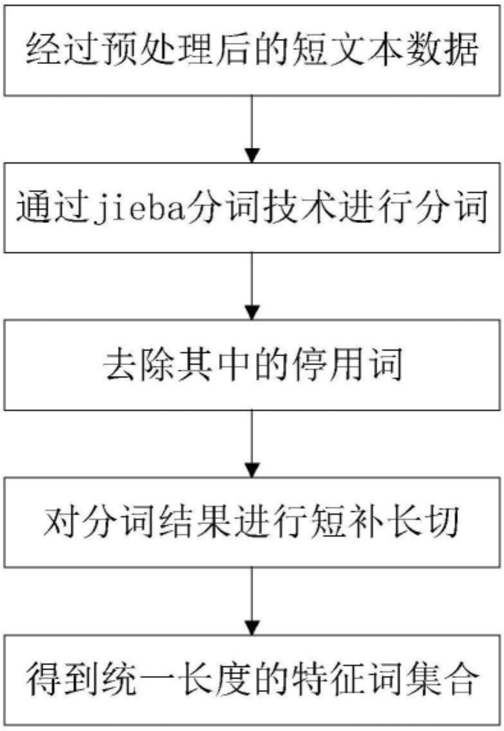
经过预处理后的短文本数据
通过jieba分词技术进行分词
去除其中的停用词
对分词结果进行短补长切
得到统一长度的特征词集合

图2

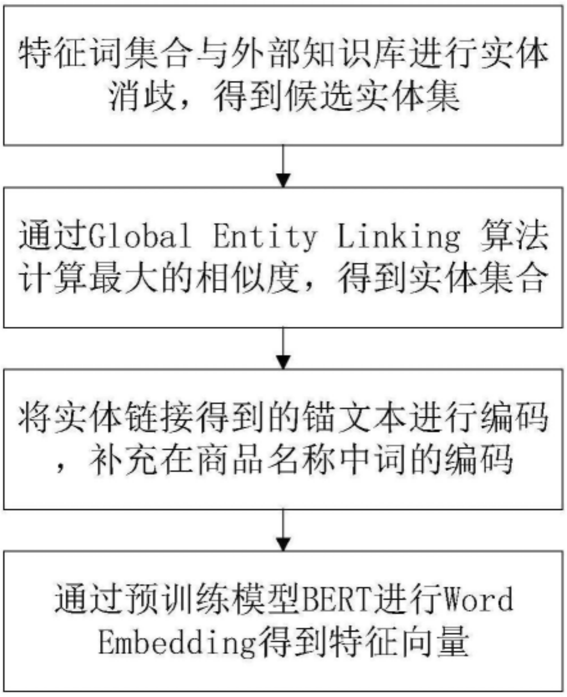
特征词集合与外部知识库进行实体消歧，得到候选实体集
通过Global Entity Linking 算法计算最大的相似度，得到实体集合
将实体链接得到的锚文本进行编码，补充在商品名称中词的编码
通过预训练模型BERT进行Word Embedding得到特征向量

图3

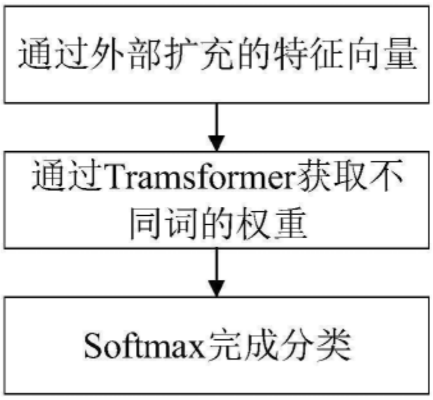
通过外部扩充的特征向量
通过Tramsformer获取不同词的权重
Softmax完成分类

图4

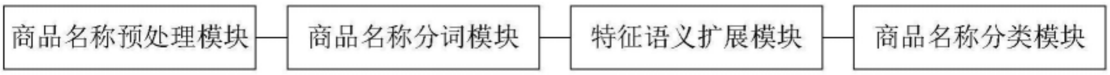
商品名称预处理模块
商品名称分词模块
特征语义扩展模块
商品名称分类模块

图5

(19)国家知识产权局

(12)发明专利

(10)授权公告号 CN 113157918 B

(45)授权公告日 2022.07.22

(21) Application No. 202110307421.9

(22) Application date: March 23, 2021

(65) The published literature number of the same application

Application Publication No. CN 113157918 A

(43) Application publication date: July 23, 2021

(73) Patentee: Zhejiang University of Technology

Address: 310014, Chao Wang Road, Xiacheng District, Hangzhou City, Zhejiang Province Number 18

(72) Inventors: Gao Nan, Chen Guoxin, Chen Lei, Yang Gui, Fang Tianbin, Yu Guo

(74) Patent Agency: Hangzhou Tianzheng Patent Agency Co., Ltd. 33201

Patent agent Wang Bing

(51)Int.Cl.

G06F 16/35 (January 2019)

G06F 40/289 (2020.01)

G06F 40/30 (2020.01)

G06N 20/00 (January 2019)

(56) Comparative documents

CN 107862046 A, March 30, 2018

CN 109062893 A, December 21, 2018

CN 108241677 A, July 3, 2018

CN 110134786 A, August 16, 2019

US 2017083983 A1, March 23, 2017

Fu Yu, et al. A Simplification Method for Long Commodity Names Based on the Self-Attention Mechanism. Journal of East China Normal University (Natural Science Edition). 2019, (Issue 5), pp. 113-122, 167.

Nan Gao, et al. A Supervised Named Entity Recognition Method Based on Pattern Matching and Semantic Verification. "Journal of Internet Technology". 2020, Vol. 21 (No. 7), pp. 1917-1928.

Reviewer Ge Xiaoxian

The claims are five pages.

The manual is 11 pages

Attached are two pages of pictures.

(54) Invention Name

A classification method and system for short commodity name texts based on the attention mechanism

(57) Abstract

A short text classification method for commodity names based on the attention mechanism, including: preprocessing the commodity names to remove non-Chinese characters and some special characters; through jieba word segmentation, dividing the preprocessed commodity short texts into several words, removing stop words, and performing short-to-long padding and cutting on the obtained words to uniform the word length to the pre-set number of words; using the Global Entity Linking algorithm to perform entity disambiguation and linking for each word, expanding the interpretation of the words in the short text by linking to the external knowledge base of Baidu Baike and using the result of the entity linkage to perform word embedding encoding with Bert to obtain the corresponding feature vector; feeding the obtained vector into the Transformer network, using the self-attention mechanism to explore the sharing degree of different words for tax code classification, assigning different weights to different words, and finally classifying them through Softmax. The tax code category with the highest probability is taken as the category to which the commodity name belongs. The present invention also includes a system for implementing the above-mentioned invention method.

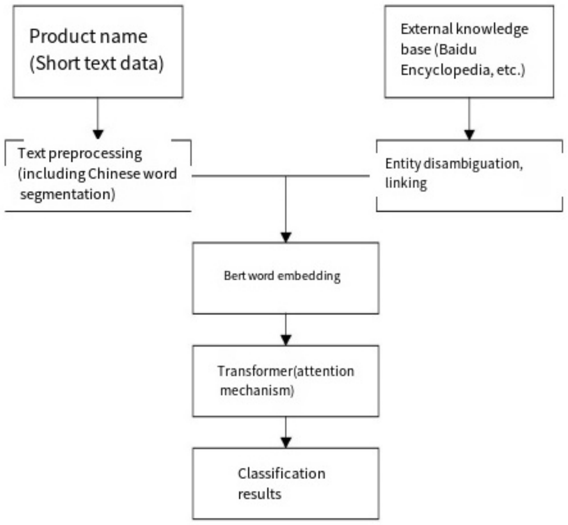

CN 113157918 B

A short text classification method for commodity names based on the attention mechanism, including the following steps:

(1) Preprocess the commodity names and retain only the Chinese character fields;

(2) Through jieba word segmentation, divide the preprocessed short text of commodity names into several words, remove stop words, perform short-to-long padding and long-to-short cutting on the obtained words, and uniform the length of the words to the pre-set number of words.

(3) Use the Global Entity Linking algorithm to perform entity disambiguation and linking for each word. By linking to the external knowledge base of Baidu Baike, expand the interpretation of the words in the short text with the results, enrich the context semantic information, and use the results of entity linking to perform word embedding encoding with Bert to obtain the corresponding feature vectors. This specifically includes the following steps:

(3 - 1) Perform entity disambiguation and linking for each word using the Global Entity Linking algorithm. By linking to the external knowledge base of Baidu Baike, expand the interpretation of the words in the short text with the results to enrich the context semantic information. The formula of the Global Entity Linking algorithm is as follows:

$$T_g = argmax_\Gamma \left[\sum_{i=1}^{N} \phi(m_i, e_i) + \sum_{e_i \in \Gamma, e_j \in \Gamma} coh(e_i, e_j) \right] \quad (1)$$

To= argmaxr ∑ ϕ(m, e) + con(ee) (1) Er,e,εr

Among them, Γ represents the set of entity spaces to be matched and determined;

Among them, $\phi(m_i, e_i)$ is the defined compatibility function.

The definition is as follows:

$$\phi(m_i, e_i) = f(m, e) \times g(m, e) \quad (2)$$

"m" represents the mention to be linked in the commodity text; "e" represents the entity in the external knowledge base.

f(m, e) is a context-free score, mainly related to the degree of irrelevance between the literal meaning of the mention and the context of the candidate entities in the knowledge base. It is defined as follows:

$$f(m, e) = p(e|m) \times sim(m, e) + \frac{\beta}{|E_m|} \quad (3)$$

f(m,e) = p(elm) x sim(m,e)+ (3)

Among them, p(e|m) represents the prior probability of the mention in the commodity text and the entity in the external knowledge base, which is obtained from the external knowledge base, specifically estimated statistically from the anchor text linked to Baidu Baike; Em refers to the set of entities that may be linked to the mention from the external knowledge base; β represents the weighted probability, which is used to balance the weights before and after through β for the reliable entity set E m; sim(m,e) represents the text similarity between the mention and the entity, which is used to constrain the prior probability p(e|m) that may contain noise. sim(m,e) is characterized by using cosine similarity.

g(m, e) is a context-dependent score, mainly related to the degree of relevance and closeness between the literal meaning of the mention and the context of the candidate entities in the knowledge base. It is defined as follows:

$$g(m, e) = sim_t(m, e) \times (1 - \in + \in \times sim_c(m, e)) \quad (4)$$

Among them, $sim_c(m, e)$ represents the cosine similarity between m and e; the parameter ∈ is used to balance and control the influence of the correlation score; $sim_t(m, e)$ is defined as follows:

$$\begin{cases} sim_t(m, e) = max_{w_c \in CT(m), w_d \in KP(e)} \cos(V_w(w_c), V_w(w_d)) \\ V_c(m) = \sum_{w \in CT(m)} \frac{v_c(w)}{D(w, m)} \end{cases} \quad (5)$$

V(m) = ∑ D(w,m) (5) WECT(m)

CT(m) represents the keyword set after context word segmentation of the commodity name; KP(e) represents the possible entity set; $v_c(w)$ represents the vectorized representation of word w; D(w, m) represents the distance function between the context word w and the word m to be linked, which is defined by the absolute distance between the words.

The function $coh(e_i, e_j)$ is defined as the set of entities determined by the context mention.

Correlation measurement between pairs

The definition is as follows:

$$coh(e_i, e_j) = \gamma \times rel(e_1, e_2) + (1 - \gamma) \times sim(e_1, e_2) \quad (6)$$

➢ sim(e1, e2) represents the negative form of the normalized Google distance and is used to measure similarity:

$$sim(e_1, e_2) = 1 - \frac{\log(\max(|E_1|, |E_2|)) - \log(|E_1 \cap E_2|)}{\log(|E|) - \log(\min(|E_1|, |E_2|))} \quad (7)$$

Among them, E1 and E2 are respectively the inline entity sets obtained from Baidu Baike for entities e1 and e2, and E represents the entire entity set. |β| represents the number of elements in the set;

The negative form of the normalized Google distance is utilized to compare the implicit between entity sets

The similarity of entities

➢ "rel(e1, e2) is used to further represent the correlation between entities and is defined as follows: "

$$\begin{cases} rel(e_1, e_2) = \max(rel'(e_1, e_2), rel'(e_2, e_1)) \\ rel'(e_1, e_2) = \sum_{r \in R(e_1, e_2)} \frac{2}{|T(e_1, r)| + |H(r, e_2)|} \end{cases} \quad (8)$$

Among them, R(e 1, e 2) represents the set of relationships between entities e 1 and e 2; T(e 1, r) represents the set of tail entities of the head entity e 1 and the relationship r; H(r, e 2) represents the set of head entities of the relationship r and the tail entity e 2; the parameter γ ∈ [0, 1] is used to balance the weights of similarity and relevance;

(3 - 2) Use Bert for word embedding encoding on the results of the entity link to obtain the corresponding feature vectors;

(4) Feed the obtained feature vectors into the Transformer network. Utilize the self-attention mechanism to explore the degree of sharing of different words for tax code classification, assign different weights to different words, and finally classify them through Softmax. Take the tax code category with the highest probability as the category to which the commodity name belongs, and finally determine the tax code category label of the commodity name to be classified.

2. A short text classification method for commodity names based on the attention mechanism as described in claim 1, characterized in that: the step (2) comprises the following specific implementation process:

(2 - 1) For the preprocessed commodity names, use the jieba library for Chinese word segmentation to obtain the preliminary segmentation results;

(2 - 2) Remove the stop words from it to obtain several feature words of commodity names;

(2 - 3) Perform short padding and long cutting on the obtained feature words, and uniform the length of the words to the pre-set number.

3. A short text classification method for commodity names based on the attention mechanism as described in claim 1, characterized in that: the step (4) comprises the following specific implementation process:

(4-1) Feed the obtained feature vectors into the Transformer network and utilize the self-attention mechanism to explore the degree of sharing of different words for tax code classification and assign different weights to different words; the formula of the said attention mechanism is as follows:

$$Attention(Q, K, V) = softmax\left(\frac{QK^T}{\sqrt{d_k}}\right)V \quad (9)$$

Among them, represents the scaling factor, which is used to optimize the defect of dot product attention by scaling the value to the change of the softmax function the most

$$\frac{1}{\sqrt{d_k}}$$

In large areas, the amplification gap is enlarged; meanwhile, in practice, the click attention mechanism is faster in calculation and has higher spatial efficiency, and can be implemented using highly optimized matrix multiplication.

(4 - 2) Classify it through Softmax, take the tax code category with the highest probability as the category to which the commodity name belongs, and finally determine the tax code category label of the commodity name to be classified.

4. A short text classification method for commodity names based on the attention mechanism as described in claim 1, characterized in that: In step (3-1), Γ < 100 is taken, that is, the most relevant set of 100 entities is sought in the external knowledge base.

5. A system for implementing the short text classification method of commodity names based on the attention mechanism as described in claim 1, characterized in that it comprises: a commodity name preprocessing module, a commodity name word segmentation module, a feature semantic extension module and a commodity name classification module connected in sequence; wherein the commodity name preprocessing module outputs the preprocessed commodity name to the commodity name word segmentation module, the commodity name word segmentation module outputs the initial feature set after word segmentation to the feature semantic extension module, the feature semantic extension module outputs the extended feature vector to the commodity name classification module, and finally the commodity name classification module outputs the tax code classification label corresponding to the commodity name.

Commodity name preprocessing module, used for preprocessing the commodity name and retaining only the Chinese character field; specifically including:

(1-1) Through regular expressions, only retain the Chinese character fields;

Commodity name word segmentation module, used for word segmentation of the preprocessed commodity name through the jieba word segmentation tool, removing stop words and unifying the number of words; specifically including:

(2 - 1) For the preprocessed commodity names, use the jieba library for Chinese word segmentation to obtain the preliminary segmentation results;

(2 - 2) Remove the stop words from it to obtain several feature words of commodity names;

(2 - 3) Perform short padding and long cutting on the obtained feature words, and uniform the length of the words to the pre-set number;

Feature semantic extension module, used to solve the problem of the lack of contextual semantics in short texts. By supplementing the semantic information of words in the feature set through the external knowledge base, a feature vector with higher quality expression is obtained; specifically including:

(3 - 1) Perform entity disambiguation and linking for each word using the Global Entity Linking algorithm. By linking to the external knowledge base of Baidu Baike, expand the interpretation of the words in the short text with the results to enrich the context semantic information. The formula of the Global Entity Linking algorithm is as follows:

$$T_g = argmax_\Gamma \left[\sum_{i=1}^{N} \phi(m_i, e_i) + \sum_{e_i \in \Gamma, e_j \epsilon \Gamma} coh(e_i, e_j) \right] \quad (1)$$

$$\text{To = argmax} \left[\sum_{i=1}^{N} \phi(\text{m, e\{}) + \sum_{\text{e\{Er,eer}} \text{coh(e,e;)} \right] \quad (1)$$

Among them, Γ represents the set of entity spaces to be matched and determined;

Among them, ϕ($_{mi}$, ei$_{)}$ is the defined compatibility function.

The definition is as follows:

$$\phi\ (m_i, e_i) = f\ (m, e) \times g\ (m, e) \quad (2)$$

"m" represents the mention to be linked in the commodity text; "e" represents the entity in the external knowledge base.

f(m, e) is a context-free score, mainly related to the degree of irrelevance between the literal meaning of the mention and the context of the candidate entities in the knowledge base. It is defined as follows:

$$f(m, e) = p(e|m) \times sim(m, e) + \frac{\beta}{|E_m|} \quad (3)$$

Among them, p(e|m) represents the prior probability of the entity in the external knowledge base corresponding to the mention in the commodity text, which is obtained from the external knowledge base, specifically estimated statistically from the anchor text linked to Baidu Baike; Em $_{refers}$ to the set of entities that may be linked to the mention from the external knowledge base; β represents the weighted probability, which is used to balance the weights before and after $_{through}$ β for the reliable entity set Em; sim(m, e) represents the text similarity between the mention and the entity, which is used to constrain the prior probability p(e|m) that may contain noise. sim(m,e) is characterized by using cosine similarity.

g(m, e) is a context-dependent score, mainly related to the literal meaning of the mention and the context of the candidate entities in the knowledge base.

It is related to the degree of closeness of the relevance between the texts, and the definition is as follows:

$$g\ (m, e) = sim_t\ (m, e) \times (1 - \in + \in \times sim_c\ (m, e)) \quad (4)$$

Among them, sim $_{c}$(m, e) represents the cosine similarity between m and e; the parameter ∈ is used to balance and control the influence of the correlation score; $_{simt}$(m, e) is defined as follows:

$$\begin{cases} sim_t(m,e) = max_{w_c \in CT(m), w_d \in KP(e)} \cos\left(V_w(w_c), V_w(w_d)\right) \\ V_c(m) = \sum_{w \in CT(m)} \frac{v_c(w)}{D(w,m)} \end{cases} \quad (5)$$

CT(m) represents the keyword set after context word segmentation of the commodity name; KP(e) represents the possible entity set; $v_c(w)$ represents the vectorized representation of word w; D(w, m) represents the distance function between the context word w and the word m to be linked, which is defined by the absolute distance between the words.

The function coh(ei, ej) is defined as the set of entities determined by the context mention.

Correlation measurement between pairs

The definition is as follows:

$$coh(e_i, e_j) = \gamma \times rel(e_1, e_2) + (1 - \gamma) \times sim(e_1, e_2) \quad (6)$$

- sim(e1, e2) represents the negative form of the normalized Google distance and is used to measure similarity:

$$sim(e_1, e_2) = 1 - \frac{\log(\max(|E_1|, |E_2|)) - \log(|E_1 \cap E_2|)}{\log(|E|) - \log(\min(|E_1|, |E_2|))} \quad (7)$$

Among them, E1 and E2 are respectively the inline entity sets obtained from Baidu Baike for entities e1 and e2, and E represents the entire entity set. |·| represents the number of elements in the set;

The negative form of the normalized Google distance is utilized to compare the implicit between entity sets

The similarity of entities

- "rel(e1, e2) is used to further represent the correlation between entities and is defined as follows: "

$$\begin{cases} rel(e_1, e_2) = \max(rel'(e_1, e_2), rel'(e_2, e_1)) \\ rel'(e_1, e_2) = \sum_{r \in R(e_1, e_2)} \frac{2}{|T(e_1, r)| + |H(r, e_2)|} \end{cases} \quad (8)$$

Among them, R(e 1, e 2) represents the set of relationships between entities e 1 and e 2; T(e 1, r) represents the set of tail entities of the head entity e 1 and the relationship r; H(r, e 2) represents the set of head entities of the relationship r and the tail entity e 2; the parameter $\gamma \in [0, 1]$ is used to balance the weights of similarity and relevance;

(3 - 2) Use Bert for word embedding encoding on the results of the entity link to obtain the corresponding feature vectors;

The commodity name classification module is used to conduct the final classification of commodity names through the Transformer network architecture. The tax code category with the highest probability is taken as the category to which the commodity name belongs, and the tax code category label of the commodity name to be classified is finally determined. This includes:

(4-1) Feed the obtained feature vectors into the Transformer network and utilize the self-attention mechanism to explore the degree of sharing of different words for tax code classification and assign different weights to different words; the formula of the said attention mechanism is as follows:

$$Attention(Q, K, V) = softmax\left(\frac{QK^T}{\sqrt{d_k}}\right)V \quad (9)$$

Among them, represents the scaling factor, which is used to optimize the defect of dot product attention by scaling the value to the change of the softmax function the most

$$\frac{1}{\sqrt{d_k}}$$

In large areas, the amplification gap is enlarged; meanwhile, in practice, the click attention mechanism is faster in calculation and has higher spatial efficiency, and can be implemented using highly optimized matrix multiplication.

(4 - 2) Classify it through Softmax, take the tax code category with the highest probability as the category to which the commodity name belongs, and finally determine the tax code category label of the commodity name to be classified.

6. The system as described in claim 5 is characterized in that: $\Gamma < 100$ is taken, that is, in the external knowledge base,

Find the collection of the 100 most relevant entities.

A classification method and system for short commodity name texts based on the attention mechanism

Technical field

[0001] The present invention relates to a classification method and system for short text of commodity names based on the attention mechanism, especially for the classification of tax codes corresponding to commodity names. The text is segmented by using a Chinese text segmentation tool. After adjusting the number of words for each commodity name to be uniform by shortening, supplementing and cutting the words, the corresponding word vectors are obtained by using Bert for word embedding of each word. After feeding the word vectors into the Transformer, the weight information of each word is obtained by using the attention mechanism, and finally classification is carried out through Softmax. The present invention involves fields such as probability models, speech models, deep learning, etc., specifically involving the modeling field based on deep learning.

Background Technology

[0002] With the continuous development of society, the tax code classification system has become increasingly complex. How to accurately classify the massive number of commodity names into the corresponding tax code classifications is an important technical requirement. More and more enterprises need to issue commodity invoices. Due to the sharp increase in transaction volume, the manual method of tax code classification has the problems of low efficiency, high cost, and the classification results being affected by human accidental errors. At the same time, it is difficult for those who are not familiar with tax codes to complete the tax code classification of more than 4, 000 types in China. Therefore, using programs to solve the heavy classification problem is the current trend.

[0003] There are the following several problems in the tax code classification of commodity names:

[0004] (1) The recording of commodity names is mostly done manually, resulting in most commodity names containing a lot of noise. The noise needs to be filtered out to reduce the impact on tax code classification.

[0005] (2) Most of the commodity names are short text types, consisting of only a few words. They cannot effectively extract context information, which also leads to the limitations of the current mainstream natural language processing methods on this issue.

[0006] (3) In China, there are over 4,000 types of tax codes classified at the fifth level, which is an extremely large number of categories and is an ultra-classification problem. Currently, it is very difficult to have an effective method to solve it.

[0007] Currently, there are approximately two methods for tax code classification:

[0008] 1) Keyword matching, that is, constructing a 1:N mapping relationship from tax codes to keywords, and then matching the corresponding keywords in the given commodity names can complete the tax code classification task.

[0009] 2) Classification method based on machine learning, that is, transforming the problem of tax code matching into a classification problem and solving it using machine learning or deep learning models.

[0010] The method of keyword matching is relatively simple, but it heavily relies on the size and accuracy of the keyword database. It can only match some goods that already exist in the keyword database, and the keywords must explicitly exist in the commodity name. If the commodity name does not contain the keywords in the keyword database, the matching will fail. Although some algorithms can solve the problem of keyword separation, such as when the keyword is "wheat flour" and the commodity name is "high-quality wheat flour from Northeast China", it cannot handle the situation where several keywords appear simultaneously in the commodity name. Currently, some practices take the longer ones among the matched keywords as the final keywords, but this strategy cannot handle all keywords of equal length. For example, for the commodity name "pen, with a small bottle of ink as a gift", the main body of the commodity is "pen", but the keyword database will also match "ink" at the same time. The keyword matching algorithm cannot distinguish the weights of the two, nor can it determine which keyword to match to the corresponding tax code. Additionally, the generalization ability of this method is poor because when different companies perform tax code matching on commodity names, most of them are embedded in professional fields, such as the power industry, where there are many technical terms that ordinary people have never even heard of. At this time, it is necessary to reconstruct or add keywords.

The repository is time-consuming and labor-intensive. Therefore, this method does not perform well on large and widely covered datasets.

[0011] The classification methods based on machine learning can be divided into supervised classification and unsupervised classification methods according to the characteristics of the training samples. Unsupervised-based classification methods, including language analysis methods, statistical methods, topic methods, and web graph-based methods, mainly solve classification problems through clustering. There are several ways to characterize the features of unsupervised algorithms in text analysis: The classic TFIDF method considers the frequency and inverse document frequency of words; TextRank considers the co-occurrence information of words; the Rake method considers the degree information of words and word frequency information in the co-occurrence matrix of words. Although many scholars have conducted a lot of research and improvements based on this, the limitations of feature engineering still cannot be avoided: 1. The feature characterization of short texts is not comprehensive. Some consider the frequency of words but ignore the part-of-speech and position information, while others consider the co-occurrence information of words but ignore the text structure information, etc., which affects the accuracy of keyword extraction. 2. The scoring mechanism of short texts is too subjective. Taking human prior knowledge as the interpretation standard of the scoring rule or not explaining the basis for setting the scoring rule at all. Therefore, unsupervised methods often perform poorly in tax code classification problems, and thus supervised classification algorithms need to be considered.

[0012] The general steps of the supervised classification method are as follows: Firstly, establish a training set that contains a large number of texts and labels the tax code classification corresponding to the commodity names; then, train the classification model using the training set; finally, apply the trained model to classify new commodity names. This method is highly practical. If an appropriate model is adopted and the appropriate parameters are set, good results can be obtained. Currently, in most mainstream natural language problems, deep learning methods are mostly used. Deep learning has the property of automatically mining features in the text and can obtain the corresponding text representation from the data, thereby avoiding heavy feature engineering. At the same time, deep learning can obtain complex models through the design and stacking of network layers to meet the requirements of complex tasks. However, general machine learning or deep learning models are difficult to achieve good results in tax code classification problems because commodity names are mostly short texts composed of some words, severely lacking context information, with problems of sparsity and noise. Common deep learning networks such as LSTM perform poorly on this problem because such models are based on the mining of context information to complete feature extraction. Therefore, how to effectively solve the problem of the lack of context semantics in short texts is particularly important.

[0013] Currently, many enterprises need to uniformly manage the records of commodity trading in their enterprises. They issue commodity invoices based on the recorded commodities, or obtain the corresponding tax rates of the commodities to determine the corresponding tax expenditures. Some enterprises can have millions or even tens of millions of commodity records in a year. At this time, relying solely on manual tax code classification is very unrealistic and subjective, making it difficult to guarantee efficiency and accuracy. At the same time, tax code classification requires some professional tax personnel to complete, leading to a further increase in costs, which is difficult for ordinary enterprises to bear. At the same time, tax code classification faces many difficulties: On the one hand, the registration process of commodity names may record some misleading noise, and some core keywords are not the helpful information we need; on the other hand, most commodity names are composed of only a few words, severely lacking context semantics. General classification algorithms are simply unable to solve the problem of short texts, further increasing the difficulty of classification. Currently, the method of manual tax code marking is both subjective and labor-intensive. Therefore, adopting effective classification algorithms to solve this problem has a very good application prospect.

[0014] Therefore, how to solve the problem of the lack of context in the short text of commodity names and complete the classification and matching of tax codes quickly and accurately has become an urgent problem to be solved by technicians.

Invention Content

[0015] The present invention aims to overcome the abovementioned shortcomings and deficiencies in the tax code classification technology of existing commodity names, and provides a commodity name short text classification method and system based on the attention mechanism to achieve automatic and accurate judgment of the tax code classification to which different commodity names belong.

[0016] To achieve the above-mentioned invention purpose, the present invention provides the following technical solution:

[0017] The present invention provides a short text classification method for commodity names based on the attention mechanism, including the following steps:

[0018] (1) Preprocess the commodity name and retain only the Chinese character fields;

[0019] (2) Through jieba word segmentation, divide the preprocessed short commodity texts into several words, remove stop words, perform short-to-long padding and long-to-short cutting on the obtained words, and uniform the length of the words to the pre-set number of words;

[0020] (3) Use the Global Entity Linking algorithm to perform entity disambiguation and linking for each word. By linking to the external knowledge base of Baidu Baike, expand the interpretation of the words in the short text with the results, enrich the context semantic information, and use the results of entity linking to perform word embedding encoding with Bert to obtain the corresponding feature vectors;

[0021] (4) Feed the obtained vectors into the Transformer network. Utilize the self-attention mechanism to explore the degree of sharing of different words for tax code classification, assign different weights to different words, and finally classify them through Softmax. Take the tax code category with the highest probability as the category to which the commodity name belongs, and finally determine the tax code category label of the commodity name to be classified.

[0022] Preferably, the step (2) comprises the following implementation process:

[0023] (2 - 1) For the preprocessed commodity names, the jieba library is used for Chinese word segmentation to obtain the preliminary segmentation results;

[0024] (2 - 2) Remove the stop words from them to obtain several feature words of commodity names;

[0025] (2 - 3) Perform short padding and long cutting on the obtained feature words to uniform the length of the words to the pre-set number;

[0026] Preferably, the step (3) comprises the following implementation process:

[0027] (3-1) Perform entity disambiguation and linking for each word using the Global Entity Linking algorithm. By linking to the external knowledge base of Baidu Baike, expand the interpretation of the words in the short text with the results to enrich the context semantic information. The formula of the Global Entity Linking algorithm is as follows:

[0028] $$T_g = argmax_{\Gamma}\left[\sum_{i=1}^{N}\phi(m_i,e_i) + \sum_{e_i\in\Gamma,e_j\in\Gamma} coh(e_i,e_j)\right] \quad (1)$$

coh(e,e) eEΓ,eεr

[0029] Among them, Γ represents the set of entity spaces to be matched and determined.

[0030]

Among them, $\phi(m_i, e_i)$ is the defined compatibility function.

The definition is as follows:

[0031] $$\phi(m_i,e_i) = f(m,e) \times g(m,e) \quad (2)$$

[0032] m represents the mention to be linked in the commodity text; e represents the entity in the external knowledge base;

[0033] f(m, e) is a context-free score, mainly related to the degree of irrelevance between the literal meaning of the mention and the context of the candidate entities in the knowledge base. It is defined as follows:

[0034] $$f(m,e) = p(e|m) \times sim(m,e) + \frac{\beta}{|E_m|} \quad (3)$$

[0035] Among them, p(e|m) represents the prior probability of the mention in the commodity text and the entity in the external knowledge base, which is usually obtained from the external knowledge base and is statistically estimated from the anchor text linked to Baidu Baike in this paper; Em refers to the set of entities that may be linked to the mention from the external knowledge base; β represents the weighted probability, which is used to balance the weights before and after through β for the reliable entity set Em; sim(m,e) represents the text similarity between the mention and the entity, which is used to constrain the prior probability p(e|m) that may contain noise. In this paper, sim(m, e) is characterized by cosine similarity.

[0036] g(m, e) is a context-dependent score, mainly related to the closeness of the correlation between the literal meaning of the mention and the context of the candidate entities in the knowledge base. It is defined as follows:

[0037] $g(m, e) = sim_t(m, e) \times (1 - \in + \in \times sim_c(m, e))$ (4)

[0038] Among them, $sim_c(m, e)$ represents the cosine similarity between m and e; the parameter ∈ is used to balance and control the correlation score

The influence; $_{simt}$(m, e) is defined as follows:

[0039]
$$\begin{cases} sim_t(m,e) = max_{w_c \in CT(m), w_d \in KP(e)} \cos\left(V_w(w_c), V_w(w_d)\right) \\ V_c(m) = \sum_{w \in CT(m)} \frac{v_c(w)}{D(w,m)} \end{cases} \quad (5)$$

[0040] CT(m) represents the keyword set after context word segmentation of the commodity name; KP(e) represents the possible entity set; vc $_($w) represents the vectorized representation of word w; D(w, m) represents the distance function between the context word w and the word m to be linked, which is defined by the absolute distance between the words.

[0041] · The function coh(ei, ej$_)$ is defined as the correlation measurement between each pair of entities in the entity set determined by the context mention.

[0042] The definition is as follows:

[0043] $$\mathrm{coh}(e_i, e_j) = \gamma \times \mathrm{rel}(e_1, e_2) + (1 - \gamma) \times \mathrm{sim}(e_1, e_2) \quad (6)$$

[0044] sim(e1, e2$_)$ represents the negative form of the normalized Google distance and is used to measure similarity:

[0045] $$sim(e_1, e_2) = 1 - \frac{\log(\max(|E_1|, |E_2|)) - \log(|E_1 \cap E_2|)}{\log(|E|) - \log(\min(|E_1|, |E_2|))} \quad (7)$$

[0046] Among them, E1 $_{and}$ E2 are $_{respectively}$ the inline entity sets obtained from $_{Baidu}$ Baike by entities e1 and e2, and E represents the entire entity set; |·| represents the number of elements in the set;

The similarity of the contained entities. The negative form of the normalized Google distance is utilized to compare the latent between entity sets.

[0047] rel(e1, e2$_)$ is used to further represent the correlation between entities and is defined as follows:

[0048]
$$\begin{cases} rel(e1, e2) = \max(rel'(e1, e2), rel'(ez, e1)) \\ rel'(e1, e2) = \frac{2\,\Sigma}{()\ rER(e1,e2)\ IT(e1,r)| + |H(r,ez)I} \end{cases}$$

[0049] Among them, R(e1, e2$_)$ represents the set of relationships $_{between}$ entities e1 $_{and}$ e2; T(e1, $_r$) represents the set of tail entities of the head entity e1 and the $_{relationship}$ r; H(r, e2) represents the set of head entities of the $_{relationship}$ r and the tail entity e2; the parameter γ ∈ [0, 1] is used to balance the weights of similarity and relevance;

[0050] (3 - 2) The result of the entity link is encoded by word embedding using Bert to obtain the corresponding feature vector;

[0051] Preferably, the step (4) comprises the following implementation process:

[0052] (4-1) Feed the obtained feature vectors into the Transformer network and utilize the self-attention mechanism to explore the degree of sharing of different words for tax code classification and assign different weights to different words; the formula of the said attention mechanism is as follows:

[0053] $$Attention(Q, K, V) = softmax\left(\frac{QK^T}{\sqrt{d_k}}\right)V \quad (9)$$

[0054] Among them, $\frac{1}{\sqrt{d_k}}$ represents the scaling factor, which is used to optimize the defect of dot product attention by scaling the value to the softmax function.

Maximize the area to amplify the gap. Meanwhile, in practice, the click attention mechanism is faster in calculation and has higher spatial efficiency, and can be implemented using highly optimized matrix multiplication.

[0055] (4 - 2) Classify it through Softmax, take the tax code category with the highest probability as the category to which the commodity name belongs, and finally determine the tax code category label of the commodity name to be classified.

[0056] Further, in step (3-1), take Γ < 100, that is, the set of 100 entities that may be relevant in the external knowledge base is collected.

[0057] Furthermore, the present invention also provides a system for implementing the aforementioned classification method of short commodity name texts based on the attention mechanism, the system comprises:

[0058] Commodity Name Preprocessing Module, used for preprocessing the commodity name and retaining only the Chinese character field; specifically including:

[0059] (1-1) Through regular expressions, only retain the Chinese character fields;

[0060] Commodity name word segmentation module, used for word segmentation of the preprocessed commodity name through the jieba word segmentation tool, removing stop words and unifying the number of words; specifically including:

[0061] (2-1) For the preprocessed commodity names, the jieba library is used for Chinese word segmentation to obtain the preliminary segmentation results;

[0062] (2 - 2) Remove the stop words from them to obtain several feature words of commodity names;

[0063] (2 - 3) Perform short padding and long cutting on the obtained feature words to uniform the length of the words to the pre-set number;

[0064] Feature semantic extension module, used to solve the problem of the lack of contextual semantics in short texts. By supplementing the semantic information of words in the feature set through the external knowledge base, a feature vector with higher quality expression is obtained; specifically including:

[0065] (3-1) Perform entity disambiguation and linking for each word using the Global Entity Linking algorithm. By linking to the external knowledge base of Baidu Baike, expand the interpretation of the words in the short text with the results to enrich the context semantic information. The formula of the Global Entity Linking algorithm is as follows:

[0066] $$T_g = argmax_\Gamma\left[\sum_{i=1}^{N} \phi(m_i, e_i) + \sum_{e_i\in\Gamma, e_j\in\Gamma} coh(e_i, e_j)\right] \quad (1)$$

I o = argmaxr [∑ φ(mi,e) + ∑ con(e,e)] (1) i=1 eEr,eer

[0067] Among them, Γ represents the set of entity spaces to be matched and determined.

[0068]

Among them, $\phi(m_i, e_i)$ is the defined compatibility function.

The definition is as follows:

[0069] $\phi(m_i, e_i) = f(m, e) \times g(m, e)$ (2)

[0070] m represents the mention to be linked in the commodity text; e represents the entity in the external knowledge base;

[0071] f(m, e) is ➢ a context-free score, mainly related to the literal meaning of the mention and the candidate entities in the knowledge base

The degree of irrelevance between contexts is related and is defined as follows:

[0072] $$f(m,e) = p(e|m) \times sim(m,e) + \frac{\beta}{|E_m|} \quad (3)$$

[0073] Among them, p(e|m) represents the prior probability of the mention in the commodity text and the entity in the external knowledge base, which is usually obtained from the external knowledge base and is statistically estimated from the anchor text linked to Baidu Baike in this paper; Em refers to the set of entities that may have links with the mention from the external knowledge base; β represents the weighted probability, which is used to balance the weights before and after through β for the reliable entity set Em; sim(m,e) represents the text similarity between the mention and the entity, which is used to constrain the prior probability p(e|m) that may contain noise. In this paper, sim(m,e) is characterized by cosine similarity.

[0074] g(m, e) is a context-dependent score, mainly related to the literal meaning of the mention and the candidate entities in the knowledge base

➢

The degree of relevance and closeness between the context is related and is defined as follows:

[0075] $$g(m,e) = sim_t(m,e) \times (1 - \in + \in \times sim_c(m,e)) \quad (4)$$

[0076] Among them, $sim_c(m, e)$ represents the cosine similarity between m and e; the parameter ∈ is used to balance and control the influence of the correlation score; $sim_t(m, e)$ is defined as follows:

[0077]
$$\begin{cases} sim_t(m,e) = max_{w_c \in CT(m), w_d \in KP(e)} \cos\left(V_w(w_c), V_w(w_d)\right) \\ V_c(m) = \sum_{w \in CT(m)} \frac{v_c(w)}{D(w,m)} \end{cases} \quad (5)$$

[0078] CT(m) represents the keyword set after context word segmentation of the commodity name; KP(e) represents the possible entity set; vc(w) represents the vectorized representation of word w; D(w, m) represents the distance function between the context word w and the word m to be linked, which is defined by the absolute distance between the words.

[0079] · The function coh(ei, ej) is defined as the correlation measurement between each pair of entities in the entity set determined by the context mention.

[0080] The definition is as follows:

[0081]
$$\mathrm{coh}(e_i, e_j) = \gamma \times \mathrm{rel}(e_1, e_2) + (1 - \gamma) \times \mathrm{sim}(e_1, e_2) \quad (6)$$

[0082] sim(e1, e2) represents the negative form of the normalized Google distance and is used to measure similarity:

[0083]
$$sim(e_1, e_2) = 1 - \frac{\log(\max(|E_1|, |E_2|)) - \log(|E_1 \cap E_2|)}{\log(|E|) - \log(\min(|E_1|, |E_2|))} \quad (7)$$

sim(e1,e2) = 1 log(IEI) - log(min(E1l,E2D)

[0084] Among them, E1 and E2 are respectively the inline entity sets obtained from Baidu Baike by entities e1 and e2, and E represents the entire entity set; | | represents the number of elements in the set;

The negative form of the normalized Google distance is utilized to compare the latent between entity sets.

The similarity of the contained entities.

[0085] rel(e1, e2) is used to further represent the correlation between entities and is defined as follows:

[0086]
$$\begin{cases} rel(e_1, e_2) = \max\left(rel'(e_1, e_2), rel'(e_2, e_1)\right) \\ rel'(e_1, e_2) = \sum_{r \in R(e_1, e_2)} \frac{2}{|T(e_1, r)| + |H(r, e_2)|} \end{cases} \quad (8)$$

[0087] Among them, R(e1, e2) represents the set of relationships between entities e1 and e2; T(e1, r) represents the set of tail entities of the head entity e1 and the relationship r; H(r, e2) represents the set of head entities of the relationship r and the tail entity e2; the parameter γ ∈ [0, 1] is used to balance the weights of similarity and relevance;

[0088] (3 - 2) The result of the entity link is encoded by word embedding using Bert to obtain the corresponding feature vector;

[0089] Commodity Name Classification Module, used to conduct the final classification of commodity names through the Transformer network architecture. The tax code category with the highest probability is taken as the category to which the commodity name belongs, and the tax code category label of the commodity name to be classified is finally determined. This includes:

[0090] (4-1) Feed the obtained feature vectors into the Transformer network and utilize the self-attention mechanism to explore the degree of sharing of different words for tax code classification and assign different weights to different words; the formula of the said attention mechanism is as follows:

[0091]
$$Attention(Q, K, V) = softmax\left(\frac{QK^T}{\sqrt{d_k}}\right)V \quad (9)$$

[0092] Among them, represents the scaling factor, which is used to optimize the defect of dot product attention by scaling the value to the softmax function.

$$\frac{1}{\sqrt{d_k}}$$

Maximize the area to amplify the gap. Meanwhile, in practice, the click attention mechanism is faster in calculation and has higher spatial efficiency, and can be implemented using highly optimized matrix multiplication.

[0093] (4 - 2) Classify it through Softmax, and take the tax code category with the highest probability as the category to which the commodity name belongs.

No. Finally determine the tax code category label of the goods names to be classified;

[0094] Preferably, take Γ < 100, that is, the set of 100 entities that may be relevant by passing through the external knowledge base.

[0095] The above-mentioned commodity name classification system retains only the Chinese character fields in the initial commodity name through the commodity name preprocessing module; then, through the commodity name word segmentation module, a series of feature word sets are obtained by using word segmentation technology, the number of words in the unified set is unified to form the initial feature set; then, through the feature semantic expansion module, which is used to solve the problem of the lack of context semantics in short texts, the semantic information of the words in the feature set is supplemented through the external knowledge base such as Baidu Baike and Wikipedia to obtain a feature vector with higher-quality expression; finally, through the commodity name classification module, the commodity name is classified finally through the Transformer network architecture, and the tax code category with the highest probability is taken as the category to which the commodity name belongs, and the tax code category label of the commodity name to be classified is finally determined. The present invention, aiming at the behavioral characteristics of simulation professionals grasping core words for judgment when conducting tax code classification, introduces the explanations of some highly technical keywords in external knowledge bases such as Baidu Baike and Wikipedia through entity link technology, supplements the problem of severe lack of context in short texts, and combines the attention mechanism in deep learning. Through the training method, the importance of different words for correct tax code classification is obtained, and the corresponding classification is completed. It can effectively capture the key information in the commodity name, and has a significant improvement in accuracy and efficiency compared with the existing methods.

[0096] Compared with the existing technologies, the present invention has the following advantages and benefits:

[0097] 1. The tax code classification method of the above-mentioned commodity names utilizes the word segmentation technology to extract the feature word set, and uses the entity linking technology to supplement the short text semantics. It also introduces the attention mechanism in Transformer to classify the tax code categories of commodity names, which solves the problems of low efficiency and high cost of manual classification.

[0098] 2. The method of supplementing the semantics of short texts based on entity links can well solve the problems caused by the severe lack of semantics of short texts during the classification process of short texts, and improve the accuracy of classification.

[0099] 3. The classification method based on the attention mechanism can well focus on the core feature words with higher contribution rates, and assign different weights to different feature words according to their contribution rates, thereby improving the accuracy of the neural network classification process.

Illustration of the attached picture

[0100] Figure 1 is a schematic diagram of the implementation process of the method of the present invention.

[0101] Figure 2 is a schematic diagram of the specific implementation process of Step (2) of the method of the present invention.

[0102] Figure 3 is a schematic diagram of the specific implementation process of Step (3) of the method of the present invention.

[0103] Figure 4 is a schematic diagram of the specific implementation process of Step (4) of the method of the present invention.

[0104] Figure 5 is a schematic diagram of the structure of the system of the present invention.

Specific implementation methods

[0105] In order to understand the above-mentioned purposes, features and advantages of the present invention more clearly, the present invention is further elaborated in detail below in combination with the accompanying drawings and specific embodiments.

[0106] To solve the problem of tax code classification of existing commodity names, in view of the various deficiencies of the tax code classification algorithms at the present stage, the present invention proposes a commodity name ultra-short text classification method based on the attention mechanism, which can comprehensively consider the behavior of professionals seizing core words for judgment when conducting tax code classification, and combine the attention mechanism in deep learning to obtain the importance of different words for correct tax code classification through the training method, avoiding the subjectivity existing in human tax code classification. Combined with the entity linking method, it introduces the explanations of some highly technical keywords in external knowledge bases such as Baidu Baike and Wikipedia to supplement the problem of severely lacking context in short texts, thereby completing the corresponding classification. It can effectively capture the key information in commodity names. Compared with the existing methods, it can effectively improve the accuracy.

There has been a certain improvement in terms of efficiency.

[0107] The present invention provides a short text classification method for commodity names based on the attention mechanism. By supplementing the short text information according to the external knowledge base and introducing the attention mechanism, different weights are assigned to different feature words, thereby better achieving the tax code classification problem of commodity names.

[0108] To achieve the above-mentioned invention purpose, the present invention provides the following technical solution as shown in Figure 1:

[0109] Step 1: Preprocess the short text data of commodity names to retain only the Chinese character fields;

[0110] The original data contains a large amount of invalid information. If feature extraction is carried out directly, it will cause significant interference to the classification accuracy. Therefore, data preprocessing is conducted before feature extraction to retain only the Chinese character fields.

[0111] Step 2: Perform word segmentation, stop word removal and uniform the number of words for the preprocessed commodity names;

[0112] Through the jieba word segmentation technology, the preprocessed short commodity texts are divided into several words, stop words are removed, and the obtained words are shortened, supplemented, and truncated to uniform the length of the words to the pre-set number of words, obtaining the core word set representing the commodity names;

[0113] Specifically, Step 2 contains the implementation process as shown in Figure 2:

[0114] (2-1) Perform word segmentation on the preprocessed short text. Use the jieba word segmentation tool to perform word segmentation on the short text. For example, after preprocessing "35kV and below cable terminals 150mm2/3 cores/household terminals cold shrink copper/AC35kV", we obtain: "and below cable terminals cores household terminals cold shrink copper", and then after word segmentation processing, we get: "and below/cable terminals/cores/household/terminals/cold shrink copper".

[0115] (2-2) Remove stop words. Remove those words that have little or even negative effect on classification. After eliminating useless words through the stop word list, the following characteristic words are obtained: "cable terminal, household interior, terminal, cold shrink copper".

[0116] (2 - 3) Perform short padding and long cutting on the feature words to unify the input length;

[0117] Step 3: Use the external knowledge base to perform entity disambiguation and linking on the processed data to expand the context semantic information;

[0118] Since most of the commodity names are short texts with an extreme lack of context information, and most of the existing deep learning classification methods rely on context information, the accuracy will be greatly affected if semantic supplementation is not carried out. At the same time, there are some domain-specific terms, such as "insulated piercing grounding ring", "anchoring line clamp", "copper connection ear", and so on. Each word is disambiguated and linked using the Global Entity Linking algorithm. By linking to the external knowledge base of Baidu Baike, the results are used to expand and interpret the words in the short text, enrich the context semantic information, encode the anchor text obtained from the entity linkage, and replace the encoding of the words in the commodity name, thereby improving the expressive power of the network model and effectively solving the problem of the lack of context in short text classification.

[0119] Specifically, Step 3 contains the implementation process as shown in Figure 3:

[0120] (3-1) Perform entity disambiguation and linking for each word using the Global Entity Linking algorithm. By linking to the external knowledge base of Baidu Baike, expand the interpretation of the words in the short text with the results to enrich the context semantic information. The formula of the Global Entity Linking algorithm is as follows:

[0121]
$$T_g = argmax_{\Gamma}\left[\sum_{i=1}^{N} \phi(m_i, e_i) + \sum_{e_i \in \Gamma, e_j \in \Gamma} coh(e_i, e_j)\right] \quad (1)$$

[0122] Among them, Γ represents the set of entity spaces to be matched and determined.

[0123]

$$Tg= argmaxr \left[\sum_{i=1}^{N} \phi(m, et) + \begin{matrix}\Sigma\ eE \\ r,eEr\end{matrix}\ coh(e,e;)\right] \quad (1)$$

Among them, $\phi(_{mi}, ei_{)}$ is the defined compatibility function.

The definition is as follows:

[0124]
$$\phi\ (m_i, e_i) = f\ (m, e) \times g\ (m, e) \quad (2)$$

[0125] m represents the mention to be linked in the commodity text; e represents the entity in the external knowledge base;

[0126] f(m, e) is a context-free score, mainly related to the degree of irrelevance between the literal meaning of the mention and the context of the candidate entities in the knowledge base. It is defined as follows:

[0127] $$f(m,e) = p(e|m) \times sim(m,e) + \frac{\beta}{|E_m|} \quad (3)$$

[0128] Among them, p(e|m) represents the prior probability of the mention in the commodity text and the entity in the external knowledge base, which is usually obtained from the external knowledge base and is statistically estimated from the anchor text linked to Baidu Baike in this paper; Em refers to the set of entities that may be linked to the mention from the external knowledge base; β represents the weighted probability, which is used to balance the weights before and after through β for the reliable entity set Em; sim(m,e) represents the text similarity between the mention and the entity, which is used to constrain the prior probability p(e|m) that may contain noise. In this paper, sim(m,e) is characterized by cosine similarity.

[0129] g(m, e) is a context-dependent score, mainly related to the closeness of the correlation between the literal meaning of the mention and the context of the candidate entities in the knowledge base. It is defined as follows:

[0130] $$g(m,e) = sim_t(m,e) \times (1 - \in + \in \times sim_c(m,e)) \quad (4)$$

[0131] Among them, $sim_c(m, e)$ represents the cosine similarity between m and e; the parameter ∈ is used to balance and control the influence of the correlation score; $sim_t(m, e)$ is defined as follows:

[0132] $$\begin{cases} sim_t(m,e) = max_{w_c \in CT(m), w_d \in KP(e)} \cos(V_w(w_c), V_w(w_d)) \\ V_c(m) = \sum_{w \in CT(m)} \frac{v_c(w)}{D(w,m)} \end{cases} \quad (5)$$

$$V(m) = \sum_{WECT(m)} \frac{ve(w)}{D(w,m)} \quad (5)$$

[0133] CT(m) represents the keyword set after context word segmentation of the commodity name; KP(e) represents the possible entity set; $v_c(w)$ represents the vectorized representation of word w; D(w, m) represents the distance function between the context word w and the word m to be linked, which is defined by the absolute distance between the words.

[0134] · The function coh(ei, ej) is defined as the correlation measurement between each pair of entities in the entity set determined by the context mention.

[0135] The definition is as follows:

[0136] $$coh(e_i, e_j) = \gamma \times rel(e_1, e_2) + (1 - \gamma) \times sim(e_1, e_2) \quad (6)$$

[0137] sim(e1, e2) represents the negative form of the normalized Google distance and is used to measure similarity:

[0138] $$sim(e_1,e_2) = 1 - \frac{\log(\max(|E_1|, |E_2|)) - \log(|E_1 \cap E_2|)}{\log(|E|) - \log(\min(|E_1|, |E_2|))} \quad (7)$$

[0139] Among them, E1 and E2 are respectively the inline entity sets obtained from Baidu Baike by entities e1 and e2, and E represents the entire entity set; | | represents the number of elements in the set;

The negative form of the normalized Google distance is utilized to compare the latent between entity sets.

$$sim(e1,e2) = 1 \frac{\log(\max(E1l.E2l)) - \log(lE1 \cap E2)}{\log(lEl) - \log(\min(E1l,E2l))} \quad (7)$$

The similarity of the contained entities.

[0140] rel(e1, e2) is used to further represent the correlation between entities and is defined as follows:

[0141] $$\begin{cases} rel(e_1,e_2) = \max(rel'(e_1,e_2), rel'(e_2,e_1)) \\ rel'(e_1,e_2) = \sum_{r \in R(e_1,e_2)} \frac{2}{|T(e_1,r)| + |H(r,e_2)|} \end{cases} \quad (8)$$

[0142] Among them, R(e 1, e 2) represents the set of relationships between entities e 1 and e 2; T(e 1, r) represents the set of tail entities of the head entity e 1 and the relationship r; H(r, e 2) represents the set of head entities of the relationship r and the tail entity e 2; the parameter γ ∈ [0, 1] is used for balancing.

The weights of similarity and correlation.

[0143] (3 - 2) The result of the entity link is encoded by word embedding using Bert to obtain the corresponding feature vector;

[0144] Step 4: Feed the obtained vectors into the Transformer network. Utilize the self-attention mechanism to explore the degree of sharing of different words for tax code classification, assign different weights to different words, and finally classify them through Softmax. Take the tax code category with the highest probability as the category to which the commodity name belongs, and finally determine the tax code category label of the commodity name to be classified.

[0145] After obtaining the text input with supplementary semantic information, the pre-trained model Bert is used for encoding. Then, the self-attention mechanism is utilized to explore the sharing degree of different words for tax code classification, assign different weights to different words, and finally classify them through Softmax. The tax code category with the highest probability is taken as the category to which the commodity name belongs, and finally the tax code category label of the commodity name to be classified is determined.

[0146] Specifically, Step 4 contains the implementation process as shown in Figure 4:

[0147] (4-1) Feed the obtained feature vectors into the Transformer network and utilize the self-attention mechanism to explore the degree of sharing of different words for tax code classification and assign different weights to different words; the formula of the said attention mechanism is as follows:

[0148] $$Attention(Q,K,V) = softmax\left(\frac{QK^T}{\sqrt{d_k}}\right)V \quad (9)$$

[0149] Among them, represents the scaling factor, which is used to optimize the defect of dot-product attention by scaling the value to the softmax function.

Maximize the area to amplify the gap. Meanwhile, in practice, the click attention mechanism is faster in calculation and has higher spatial efficiency, and can be implemented using highly optimized matrix multiplication.

$$\frac{1}{\sqrt{d_k}}$$

[0150] (4-2) Classify it through Softmax, take the tax code category with the highest probability as the category to which the commodity name belongs, and finally determine the tax code category label of the commodity name to be classified.

[0151] In order to implement the above-mentioned short text classification method of commodity names based on the attention mechanism, the present invention also provides a short text classification system of commodity names based on the attention mechanism, as shown in Figure 5, which mainly includes: the commodity name preprocessing module, the commodity name word segmentation module, the feature semantic expansion module and the commodity name classification module connected in sequence; among them, the commodity name preprocessing module outputs the preprocessed commodity name to the commodity name word segmentation module, the commodity name word segmentation module outputs the initial feature set after word segmentation to the feature semantic expansion module, the feature semantic expansion module outputs the expanded feature vector to the commodity name classification module, and finally the commodity name classification module outputs the tax code classification label corresponding to the commodity name.

[0152] Commodity name preprocessing module, used for preprocessing the commodity name and retaining only the Chinese character field;

[0153] Commodity name word segmentation module, used for word segmentation of the preprocessed commodity name through the jieba word segmentation tool, removing stop words and unifying the number of words;

[0154] Feature semantic extension module, used to solve the problem of the lack of contextual semantics in short texts. By supplementing the semantic information of words in the feature set through the external knowledge base, a feature vector with higher quality expression is obtained;

[0155] Commodity Name Classification Module, used to conduct the final classification of commodity names through the Transformer network architecture. The tax code category with the highest probability is taken as the category to which the commodity name belongs, and the tax code category label of the commodity name to be classified is finally determined.

[0156] The above-mentioned short text classification system for commodity names retains only the Chinese character fields in the initial commodity names through the commodity name preprocessing module; then, through the commodity name word segmentation module, a series of feature word sets are obtained by using word segmentation technology, the number of words in the unified set is unified to form the initial feature set; and then through the feature semantic expansion module, which is used to solve the context semantics in the short text

The missing problem is solved by supplementing the semantic information of the words in the feature set through the external knowledge base to obtain a feature vector with higher-quality expression. Finally, through the commodity name classification module, the Transformer network architecture is used for the final classification of commodity names, which can effectively capture the key information in the commodity names and take the tax code category with the highest probability as the category to which the commodity name belongs. The tax code category label of the commodity name to be classified is finally determined. Compared with the existing methods, there is a significant improvement in accuracy and efficiency, greatly reducing labor costs.

[0157] The present invention has been described through the above examples, but it should be noted that the examples are only for illustrative purposes and do not limit the invention to the scope of the examples. Although the invention has been described in detail with reference to the aforementioned examples, researchers in the field should be able to understand that it can still be modified or some of its technical features can be replaced equally as described in the technical solutions recorded in the aforementioned examples; second, these modifications or replacements do not cause the corresponding technical solutions to fall outside the scope of protection of the present invention. The scope of protection of the present invention is defined by the attached claims and their equivalent scopes.

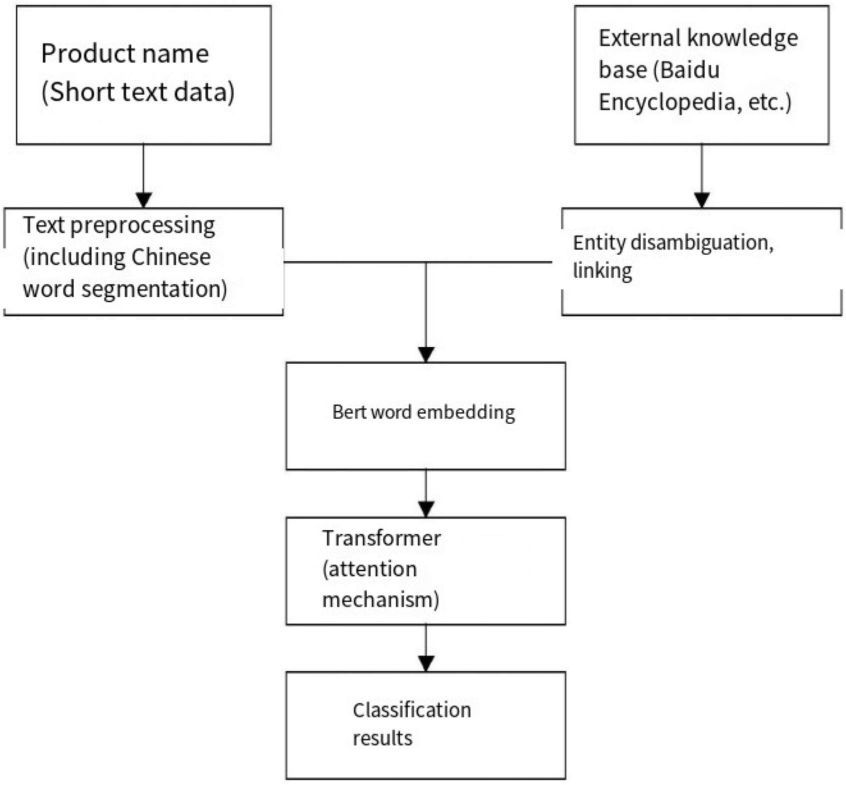

Figure 1

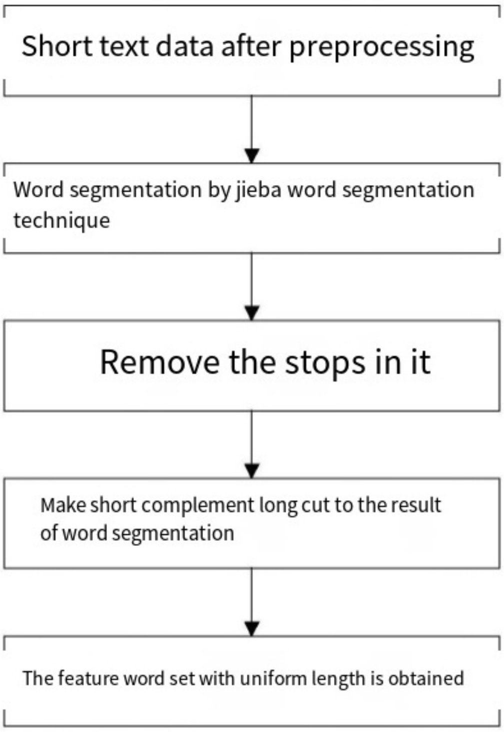

Figure 2

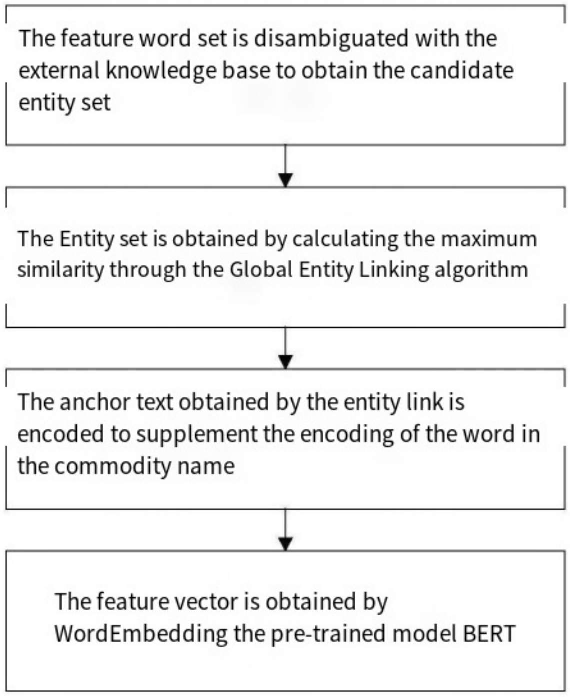

Figure 3

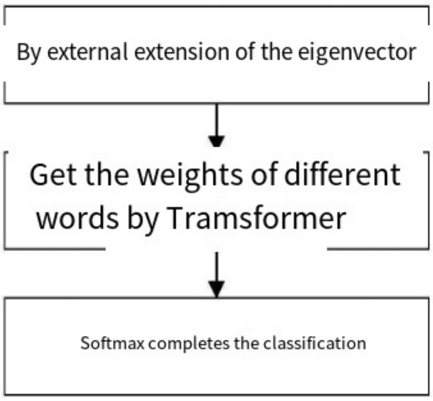

Figure 4

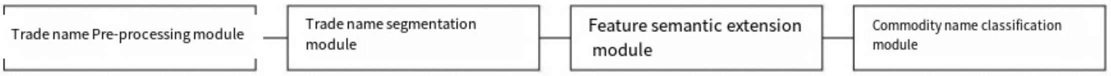

Figure 5
