# Supplementary material for: An Intelligent System for Classifying Patient Complaints Using Machine Learning and Natural Language Processing: Development and Validation Study
Source: J Med Internet Res. 2025 Jan 8;27:e55721. doi: 10.2196/55721 (PMC11754990; doi:10.2196/55721)
Supplement: Multimedia Appendix 4 [file jmir_v27i1e55721_app4.docx]

| Complaints related stop words | Reason to add into stop words |
| --- | --- |
| Doctor | This is a common word in the field of medical complaints, but when extracting high-frequency words, for specific analysis purposes, we may want to include doctor as a regular word instead of high-frequency words into stop words. |
| Nurse | Similar to doctors, nurses are also one of the common words that may appear in medical complaints, but they may not be the key words we pay attention to. |
| Department | In a hospital setting, departments are medical departments with different divisions of labor. Although this is a commonly used word, in high-frequency word extraction, we usually pay more attention to specific medical complaint words rather than department names. |
| Lead to | This is a common verb phrase, but when analyzing a medical complaint, we may be more concerned with the specific cause of the complaint than with the verb phrase itself. |
| Do | This is a general verb, but in the field of medical complaints, there is not enough critical information to describe the specific content of the complaint. |
| Reception | In a hospital, the reception desk is the location for patient auxiliary services, but in high-frequency word extraction, we may pay more attention to words directly related to complaints. |
| Examine | Although this is a behavior that may be involved in medical complaints, in high-frequency word extraction, more attention is usually paid to the reasons for the complaints, specific demands, etc. |
| Need | This is a common verb, but in high-frequency word extraction, it may not provide enough key information to describe the specific medical complaint content. |
| See a doctor | Although this is a common way for patients to seek medical treatment, in high-frequency word extraction, we may pay more attention to specific complaints, demands, etc. |
| Prevent | This is a common verb, but in medical complaint analysis, there is usually more focus on the reasons why the complaint occurred rather than on specific actions to prevent it. |
| Related | Although this is a common adjective, in high-frequency word extraction, more attention is paid to the specific complaint details and reasons rather than the specific description related to it. |
| Complaint | Although this is the core key word in the field of medical complaints, when extracting high-frequency words, we may want to focus on other key words besides the complaint itself. |
| Personnel | This is a general term, but a description directly related to a medical complaint may be more helpful in understanding the nature of the problem. |
| Dissatisfied | Although this is an emotional expression related to complaints, in high-frequency word extraction, more attention is paid to the specific details and appeals of complaints. |
| Discover | Although this is a common verb, when analyzing a medical complaint, there may be more focus on the specific circumstances and causes found. |
| Require | Although this is a common verb, in high-frequency word extraction, more attention may be paid to the specific details and requirements of the complaint. |
| Propose | Although this is a related verb, in high-frequency word extraction, we may pay more attention to the specific complaint content and related issues rather than the raising behavior itself. |
