# Supplementary material for: An Intelligent System for Classifying Patient Complaints Using Machine Learning and Natural Language Processing: Development and Validation Study
Source: J Med Internet Res. 2025 Jan 8;27:e55721. doi: 10.2196/55721 (PMC11754990; doi:10.2196/55721)
Supplement: Multimedia Appendix 5 [file jmir_v27i1e55721_app5.docx]

**Appendix Table 5. Comparison of the Performance of Three Machine Learning Algorithms: Point Estimate and 95% Confidence Intervals.**

|  |  | **Communication Problem** | | **Diagnosis and Treatment** | | **Management Problem** | | **Sense of responsibility** | |
| --- | --- | --- | --- | --- | --- | --- | --- | --- | --- |
|  |  | Training  PE(95% CI*) | Validation  PE(95% CI*) | Training  PE(95% CI*) | Validation  PE(95% CI*) | Training  PE(95% CI*) | Validation  PE(95% CI*) | Training  PE(95% CI*) | Validation  PE(95% CI*) |
| **MLR** | precision | 0.83 (0.83, 0.87) | 0.71 (0.69, 0.73) | 0.89 (0.88, 0.91) | 0.83 (0.82, 0.85) | 0.99 (0.99, 1.00) | 0.97 (0.96, 0.99) | 1.00 (0.99, 1.00) | 0.99 (0.98, 1.00) |
|  | recall | 0.95 (0.95, 0.97) | 0.91 (0.90, 0.92) | 0.88 (0.85, 0.90) | 0.81 (0.79, 0.83) | 0.94 (0.93, 0.96) | 0.88 (0.86, 0.89) | 0.96 (0.95, 0.97) | 0.89 (0.88, 0.92) |
|  | f1-score | 0.89 (0.88, 0.91) | 0.80 (0.79, 0.81) | 0.88 (0.86, 0.91) | 0.82 (0.82, 0.84) | 0.96 (0.95, 0.99) | 0.92 (0.90, 0.96) | 0.98 (0.97, 0.99) | 0.94 (0.93, 0.96) |
|  | AUC^*^ | 0.89 (0.89, 0.92) | 0.87 (0.86, 0.89) | 0.92 (0.91, 0.94) | 0.89 (0.87, 0.91) | 0.92 (0.92, 0.94) | 0.89 (0.87, 0.93) | 0.97 (0.96, 0.99) | 0.94 (0.93, 0.95) |
|  | Accuracy | 0.95 (0.95, 0.97) | 0.91 (0.90, 0.92) | 0.88 (0.88, 0.90) | 0.81 (0.79, 0.83) | 0.94 (0.93, 0.95) | 0.92 (0.90, 0.93) | 0.96 (0.95, 0.97) | 0.89 (0.87, 0.90) |
| **SVM** | precision | 0.75 (0.73, 0.76) | 0.67 (0.67, 0.69) | 1.00 (0.97, 1.00) | 0.95 (0.95, 0.96) | 1.00 (0.99, 1.00) | 0.99 (0.98, 1.00) | 1.00 (0.99, 1.00) | 1.00 (0.99, 1.00) |
|  | recall | 1.00 (0.98, 1.00) | 0.98 (0.97, 0.99) | 0.96 (0.96, 0.97) | 0.81 (0.81, 0.83) | 0.98 (0.97, 0.99) | 0.92 (0.90, 0.95) | 0.96 (0.94, 0.97) | 0.92 (0.90, 0.94) |
|  | f1-score | 0.86 (0.84, 0.88) | 0.80 (0.78, 0.82) | 0.98 (0.94, 0.99) | 0.87 (0.86, 0.89) | 0.99 (0.98, 1.00) | 0.95 (0.95, 0.97) | 0.98 (0.95, 0.99) | 0.96 (0.94, 0.98) |
|  | AUC^*^ | 0.98 (0.98, 0.99) | 0.92 (0.89, 0.95) | 0.94 (0.92, 0.95) | 0.91 (0.91, 0.93) | 0.98 (0.97, 0.99) | 0.96 (0.96, 0.98) | 0.99 (0.98, 1.00) | 0.95 (0.93, 0.97) |
|  | Accuracy | 0.98 (0.98, 0.99) | 0.98 (0.97, 0.99) | 0.96 (0.96, 0.97) | 0.81 (0.79, 0.85) | 0.98 (0.97, 0.99) | 0.92 (0.91, 0.94) | 0.96 (0.95, 0.98) | 0.92 (0.91, 0.94) |
| **MNB** | precision | 0.73 (0.71, 0.75) | 0.67 (0.66, 0.69) | 0.65 (0.65, 0.69) | 0.95 (0.94, 0.96) | 1.00 (0.99, 1.00) | 0.99 (0.98, 1.00) | 1.00 (0.99, 1.00) | 1.00 (0.99, 1.00) |
|  | recall | 1.00 (0.99, 1.00) | 0.96 (0.93, 0.97) | 0.94 (0.91, 0.95) | 0.81 (0.81, 0.85) | 0.96 (0.96, 0.98) | 0.92 (0.91, 0.94) | 0.97 (0.95, 0.99) | 0.92 (0.91, 0.94) |
|  | f1-score | 0.84 (0.83, 8.87) | 0.80 (0.77, 0.81) | 0.96 (0.94, 0.97) | 0.87 (0.83, 0.89) | 0.98 (0.98, 0.99) | 0.95 (0.94, 0.97) | 0.98 (0.97, 0.99) | 0.96 (0.95, 0.97) |
|  | AUC^*^ | 0.94 (0.94, 0.96) | 0.91 (0.88, 0.93) | 0.87 (0.85, 0.90) | 0.85 (0.85, 0.87) | 0.98 (0.97, 0.99) | 0.92 (0.90, 0.94) | 0.97 (0.96, 0.98) | 0.93 (0.91, 0.94) |
|  | Accuracy | 1.00 (0.99, 1.00) | 0.96 (0.94, 0.98) | 0.94 (0.92, 0.95) | 0.81 (0.79, 0.84) | 0.96 (0.95, 0.97) | 0.92 (0.90, 0.95) | 0.97 (0.96, 0.99) | 0.92 (0.91, 0.94) |
