# Supplementary material for: An Intelligent System for Classifying Patient Complaints Using Machine Learning and Natural Language Processing: Development and Validation Study
Source: J Med Internet Res. 2025 Jan 8;27:e55721. doi: 10.2196/55721 (PMC11754990; doi:10.2196/55721)
Supplement: Multimedia Appendix 6 [file jmir_v27i1e55721_app6.docx]

**Appendix Table 6: Point Estimate and 95% confidence intervals of each model’s performance metrics in distinguishing the four categories**

|  | **Logistic Regression** | | **MultinomialNB** | | **SVM** | |
| --- | --- | --- | --- | --- | --- | --- |
|  | **training Point Estimate(95% CI^*^)** | **test Point Estimate(95% CI)** | **training Point Estimate(95% CI)** | **test Point Estimate(95% CI)** | **training Point Estimate(95% CI)** | **test Point Estimate(95% CI)** |
| **Precision_macro^Δ^** | 0.87 (0.83, 0.90) | 0.76 (0.70, 0.82) | 0.83 (0.81, 0.86) | 0.76 (0.69, 0.84) | 0.91 (0.87, 0.96) | 0.79 (0.72, 0.87) |
| **Precision_micro^★^** | 0.86 (0.83, 0.90) | 0.76 (0.69, 0.82) | 0.83 (0.80, 0.86) | 0.75 (0.68, 0.83) | 0.91 (0.86, 0.95) | 0.78 (0.70, 0.86) |
| **Precision_weighted^⭘^** | 0.87 (0.83, 0.90) | 0.76 (0.70, 0.82) | 0.83 (0.81, 0.86) | 0.76 (0.69, 0.84) | 0.91 (0.87, 0.96) | 0.79 (0.72, 0.87) |
| **Recall_macro** | 0.86 (0.83, 0.90) | 0.76 (0.69, 0.82) | 0.83 (0.80, 0.86) | 0.75 (0.68, 0.83) | 0.91 (0.86, 0.95) | 0.78 (0.70, 0.86) |
| **Recall_micro** | 0.86 (0.83, 0.90) | 0.76 (0.69, 0.82) | 0.83 (0.80, 0.86) | 0.75 (0.68, 0.83) | 0.91 (0.86, 0.95) | 0.78 (0.70, 0.86) |
| **Recall_weighted** | 0.86 (0.83, 0.90) | 0.76 (0.69, 0.82) | 0.83 (0.80, 0.86) | 0.75 (0.68, 0.83) | 0.91 (0.86, 0.95) | 0.78 (0.70, 0.86) |
| **F1-Score_macro** | 0.86 (0.82, 0.90) | 0.75 (0.68, 0.82) | 0.83 (0.79, 0.86) | 0.75 (0.67, 0.83) | 0.90 (0.85, 0.96) | 0.78 (0.69, 0.86) |
| **F1-Score_micro** | 0.86 (0.83, 0.90) | 0.76 (0.69, 0.82) | 0.83 (0.80, 0.86) | 0.75 (0.68, 0.83) | 0.91 (0.86, 0.95) | 0.78 (0.70, 0.86) |
| **F1-Score_weighted** | 0.86 (0.82, 0.90) | 0.75 (0.68, 0.82) | 0.83 (0.79, 0.86) | 0.75 (0.67, 0.83) | 0.90 (0.85, 0.96) | 0.78 (0.69, 0.86) |
| **roc_auc_ovo^§^** | 0.97 (0.96, 0.98) | 0.92 (0.87, 0.98) | 0.96 (0.95, 0.97) | 0.92 (0.86, 0.97) | 0.98 (0.97, 1.00) | 0.94 (0.90, 0.98) |
| **roc_auc_ovr^※^** | 0.97 (0.96, 0.98) | 0.92 (0.87, 0.98) | 0.96 (0.95, 0.97) | 0.92 (0.86, 0.97) | 0.98 (0.97, 1.00) | 0.94 (0.90, 0.98) |
| **roc_auc_ovo_weighted** | 0.97 (0.96, 0.98) | 0.92 (0.87, 0.98) | 0.96 (0.95, 0.97) | 0.92 (0.86, 0.97) | 0.99 (0.97, 1.00) | 0.94 (0.90, 0.98) |
| **roc_auc_ovr_weighted** | 0.97 (0.96, 0.98) | 0.92 (0.87, 0.98) | 0.96 (0.95, 0.97) | 0.92 (0.86, 0.97) | 0.98 (0.97, 1.00) | 0.94 (0.90, 0.98) |

*: 95% Confidence Interval

macro^Δ^: Each class is equally important

micro^★^: Based on global count

weighted^⭘^: Weighted average based on the support (number of samples) of each class

ovo^§^: One-to-one strategy for multiple categories

ovr^※^: One-to-many strategy for multiple classifications
