# Supplementary figures and images for: An Intelligent System for Classifying Patient Complaints Using Machine Learning and Natural Language Processing: Development and Validation Study
Source: J Med Internet Res. 2025 Jan 8;27:e55721. doi: 10.2196/55721 (PMC11754990; doi:10.2196/55721)

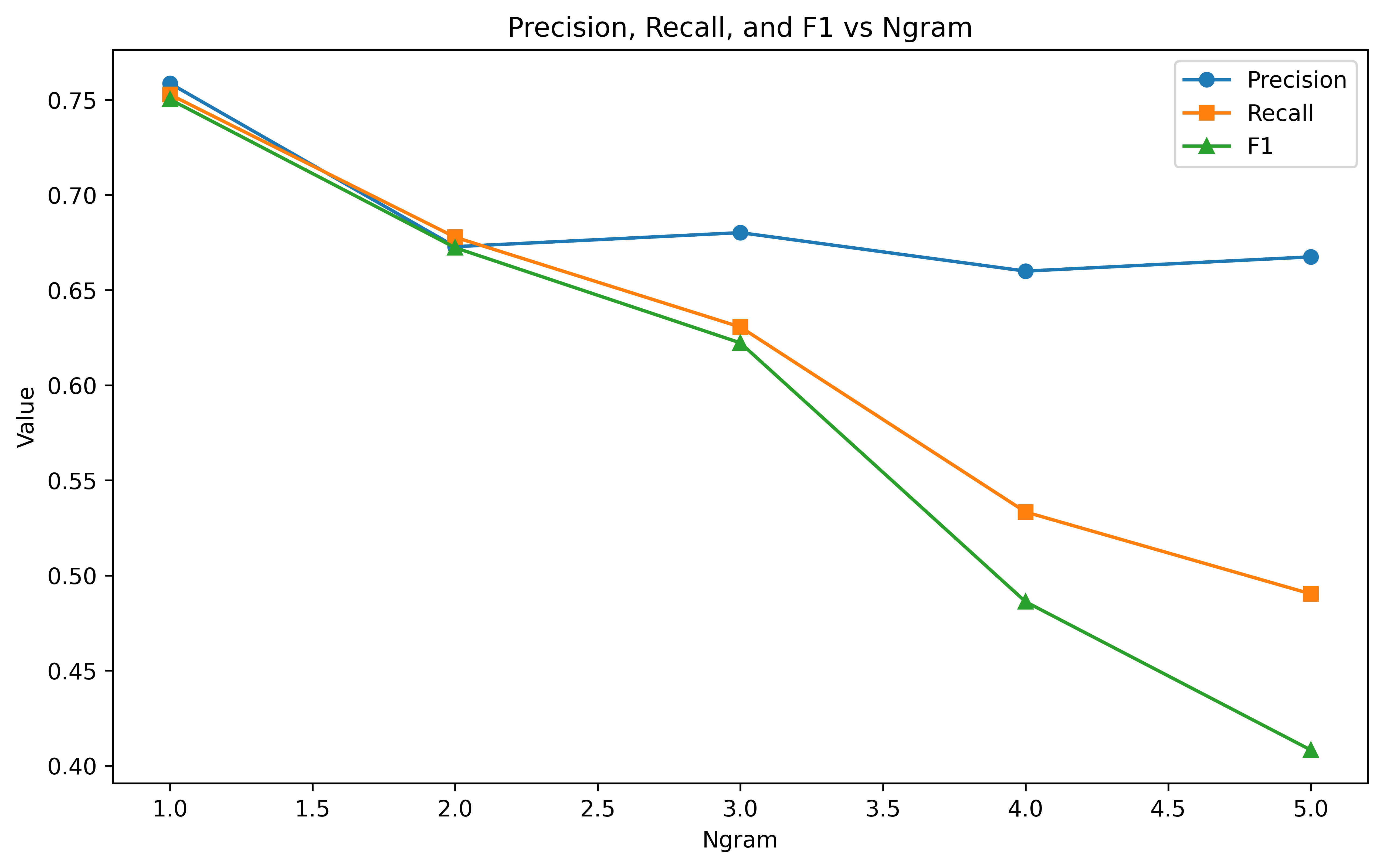

Supplement: Multimedia Appendix 7 [file jmir_v27i1e55721_app7.png]

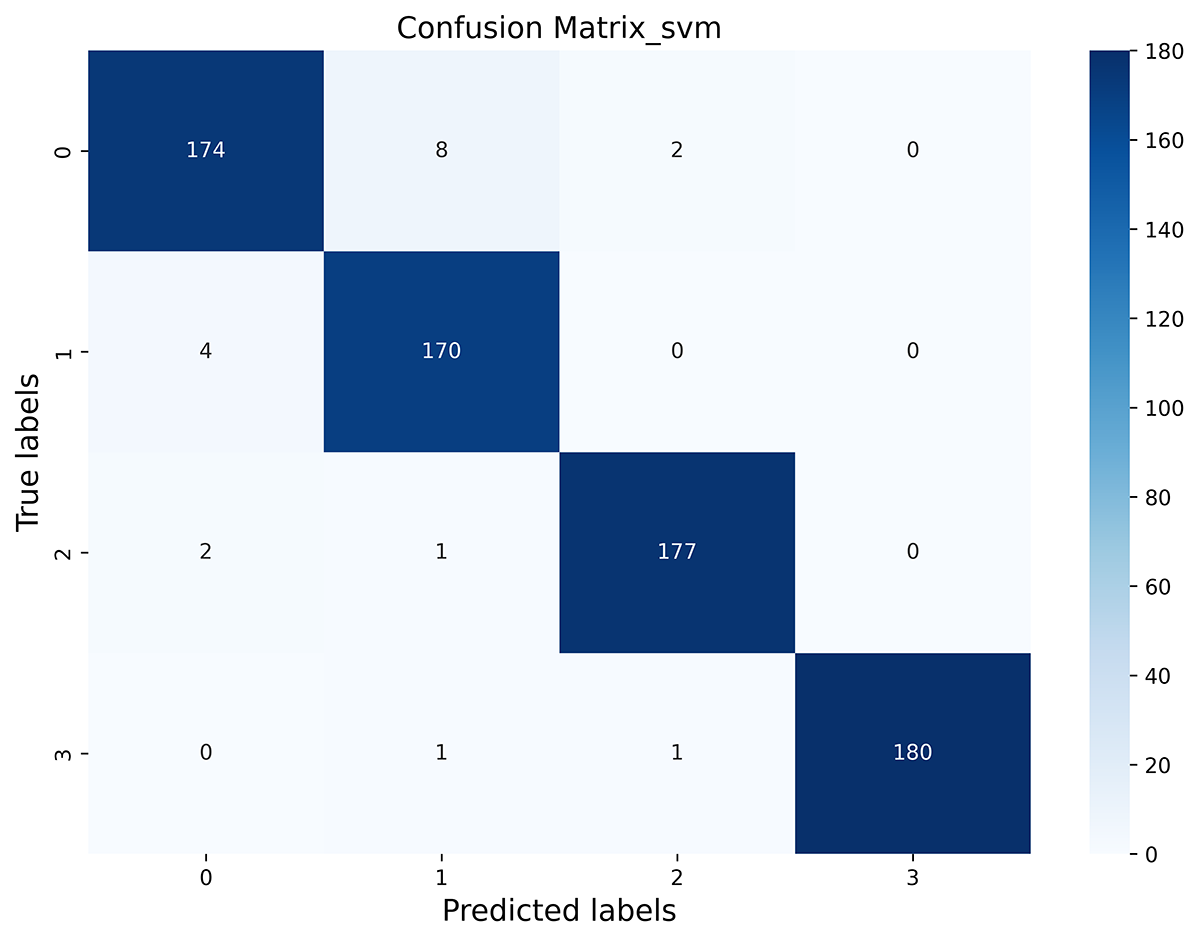

Supplement: Multimedia Appendix 9 [file jmir_v27i1e55721_app9.png]

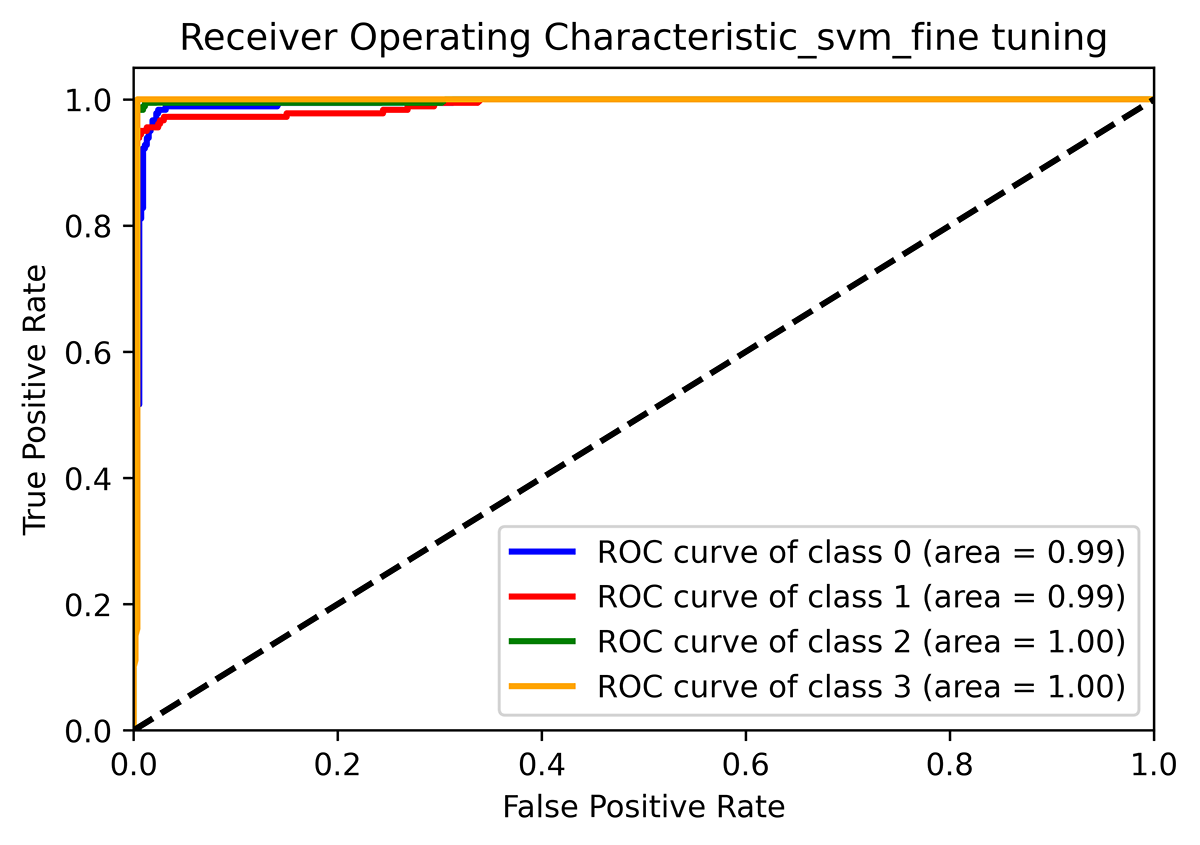

Supplement: Multimedia Appendix 10 [file jmir_v27i1e55721_app10.png]
