# Supplementary material for: An Intelligent System for Classifying Patient Complaints Using Machine Learning and Natural Language Processing: Development and Validation Study
Source: J Med Internet Res. 2025 Jan 8;27:e55721. doi: 10.2196/55721 (PMC11754990; doi:10.2196/55721)
Supplement: Multimedia Appendix 8 [file jmir_v27i1e55721_app8.docx]

| **Parameter** | **Descriptions** | **Initial value** |
| --- | --- | --- |
| C | Penalty parameter C, used to control the error tolerance of the classifier.   Smaller C values ​​result in higher error tolerance, which may lead to underfitting of the model;   larger C values ​​result in lower error tolerance, which may lead to overfitting of the model. | 1 |
| break_ties | Whether to break ties when predicting. The default value is False, which means no tie breaking. | FALSE |
| cache_size | Cache size, used to store kernel matrices. A larger cache size may increase training speed. | 200 |
| class_weight | Class weight, used to deal with class imbalance. | None |
| coef0 | Parameters in the kernel function that affect the degree of model fit. | 0 |
| decision_function_shape | The shape of the decision function, which can be 'ovo' (one-to-one) or 'ovr' (one-to-other). | ovr |
| degree | The degree of the polynomial kernel function. | 3 |
| gamma | The coefficient of the kernel function. Larger gamma values ​​will cause the model to fit the training data better, but may cause overfitting. | scale |
| kernel | Kernel type, which can be 'linear' (linear kernel), 'poly' (polynomial kernel), 'rbf' (Gaussian kernel), 'sigmoid' (sigmoid kernel), etc. | rbf |
| max_iter | Maximum number of iterations, used to control the number of iterations of training. | -1 |
| probability | Whether to enable the probability estimation function. | TRUE |
| random_state | Random seed, used to reproduce experimental results. | None |
| shrinking | Whether to enable the shrinking heuristic algorithm. | TRUE |
| tol | Precision, used to specify the tolerance for stopping model training. | 0.001 |
| verbose | Verbosity, controls the level of detail of output information. | FALSE |
